# SLC44A2 negatively regulates mitochondrial fatty acid oxidation to suppress colorectal progression by blocking the MUL1-CPT2 interaction

**DOI:** 10.1038/s41419-025-07781-z

**Published:** 2025-07-01

**Authors:** Ying Yang, Longlong Zheng, Jiaxing He, Tao Wu, Haicheng Yang, Bo Zhang, Shuai Zhou, Yueyue Lu, Xianli He, Jibin Li, Nan Wang

**Affiliations:** 1 Department of General Surgery, Tangdu Hospital, the Air Force Medical University, Xi’an, 710038 China; 2 State Key Laboratory of Holistic Integrative Management of Gastrointestinal Cancers and Department of Physiology and Pathophysiology, the Air Force Medical University, Xi’an, 710032 China

**Keywords:** Colon cancer, Rectal cancer

## Abstract

The dependence of cancer cells on mitochondrial metabolism has been revealed in various cancer types. However, the mechanisms underlying this metabolic remodeling remain largely unclear. Solute carrier family 44 member 4 (SLC44A2) is a mitochondrial membrane-localized transmembrane protein belonging to the choline transporter-like protein family. Recently, it was reported that deletion of SLC44A2 impairs adhesion and increases proliferation in cultured lung mesenchymal cells. This finding implies that SLC44A2 may play a role in the malignant phenotypes of human cancers. However, the effects of SLC44A2 on malignant phenotypes and mitochondrial metabolism in human cancers remain unexplored. In the present investigation, we observed a significant reduction in SLC44A2 expression in colorectal cancer (CRC), and low SLC44A2 expression was closely associated with poorer survival of CRC patients. Functional assays demonstrated that SLC44A2 suppressed CRC growth and metastasis both in vitro and in vivo. Mechanistically, SLC44A2 inhibits mitochondrial fatty acid oxidation, thereby reducing energy supply and increase ROS stress. This effect is achieved by promoting mitochondrial E3 ubiquitin ligase 1 (MUL1)-regulated degradation of carnitine palmitoyltransferase 2 (CPT2) via enhancing the interaction between MUL1 and CPT2, without increasing MUL1 expression, which ultimately contributes to the proliferation and metastasis of CRC. Together, SLC44A2 functions as a critical tumor suppressor in CRC and potential therapeutic target in the treatment of this malignancy.

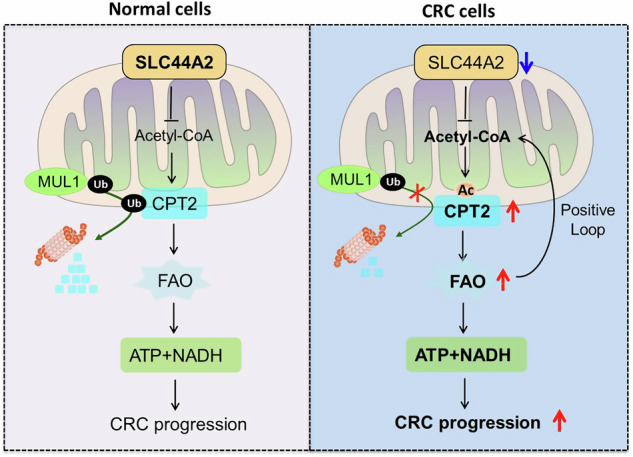

## Introduction

Colorectal cancer (CRC) is among the top five leading cancers for both incidence and mortality globally [1]. Despite significant progress in understanding and managing CRC, the long-term survival rate of patients remains largely unsatisfactory. This is primarily due to the lack of understanding of the molecular mechanisms that drive the uncontrolled proliferation and metastasis of CRC [2]. Therefore, there is an unmet need to provide further insights into the causative molecular factors of CRC progression, which could facilitate the identification of molecular markers for early detection and the development of more effective therapeutic strategies.

Accumulating evidence in various cancer types now suggests that, despite increased aerobic glycolysis, enhanced mitochondrial metabolism is also required for tumorigenesis [3, 4]. The co-existence of mitochondrial oxidative phosphorylation (OXPHOS) and enhanced glycolytic in cancer cells is due to the wider source of tricarboxylic acid (TCA) cycle substrates, mainly including fatty acids and amino acids, which are caused by extensive metabolism reprogramming [5]. Although mitochondrial metabolism has been acknowledged for its significant contribution to cancer progression and has emerged as a promising therapeutic target [6], the underlying molecular mechanisms leading to mitochondrial metabolism remodeling remain to be determined.

Solute carrier family 44 member 4 (SLC44A2) is a mitochondrial membrane-localized transmembrane protein that shares homology with choline transporters [7]. SLC44A2 knockout mice have been shown to exhibit progressive hearing loss [8], decreased venous thrombosis [9] and epilepsy [10]. SLC44A2 is also involved in regulating choline transport into mitochondria, leading to increased ATP production and mitochondrial oxygen consumption [11]. Notably, deletion of SLC44A2 impairs adhesion but increases proliferation in cultured mesenchymal lung cells [12], implying that SLC44A2 may play a role in the malignant phenotypes of human cancers. Nonetheless, the expression patterns of SLC44A2, as well as its impact on mitochondrial metabolism and malignancy progression in human cancers, including CRC, remain largely unexplored.

In the current study, we conducted a comprehensive analysis of the biological roles of SLC44A2 in mitochondrial metabolism and malignant progression in this malignancy. Our results indicate that SLC44A2 acts as a tumor suppressor in CRC by downregulating CPT2-mediated mitochondrial fatty acid oxidation via increasing the interaction between MUL1 and CPT2, supporting SLC44A2 as a potential therapeutic target in the treatment of this malignancy.

## Materials and methods

### Cell culture

In this study, seven cell lines were used, including the normal human colon epithelial cell line NCM460 and six colorectal cancer (CRC) cell lines: HCT116, HT29, SW480, LOVO, SW620, and LS174T. All cell lines underwent authentication through Short Tandem Repeat (STR) analysis through the Short Tandem Repeat (STR) analysis and were confirmed to be free from mycoplasma. These cell lines were purchased from the Cell bank of Chinese Academy of Sciences (Shanghai, China) maintained in either Dulbecco’s modified Eagle’s medium (HyClone) or RPMI 1640 medium supplemented with 10% fetal bovine serum (FBS)within a humidified incubator set at 37 °C and 5% CO_2_.

### Tissue collection

A collection of 303 pairs of CRC tissues and their corresponding adjacent normal colon tissue were collected at the Second Affiliated Hospital of the Air Force Medical University with informed consent from each participant involved in the study. Of these, 35 pairs were used for real-time quantitative PCR analysis, while the remaining 268 pairs were used for immunohistochemical analysis. This research protocol received ethical approval from the Ethics Committee of the First Affiliated Hospital of the Air Force Medical University (Approval No. KY20234028-1).

### Gene silencing and overexpression

To achieve the overexpression of SLC44A2 in CRC cells, the complete human SLC44A2 cDNA was amplified using PCR and cloned into a pcDNA3.1 vector. For the knockdown of SLC44A2, two distinct small interfering RNA targeting SLC44A2 (SLC44A2#1: 5’-AAGTATGATCCCACTTTCAAAGG-3’; SLC44A2#2: 5’-TCCTTGAAGTCATTATCATCTTG-3’) were used. The transfection of either the plasmid or siRNA was performed utilizing Lipofectamine 2000 Reagent (Invitrogen), in accordance with the manufacturer’s protocol. Twenty-four hours post-transfection, the success of gene silencing and overexpression was verified using qRT-PCR and Western blot analysis.

### Quantitative real-time PCR

Quantitative real-time polymerase chain reaction (qRT-PCR) was performed to assess gene expression at the mRNA level. Total RNA was isolated from CRC cell lines or tissues using Trizol reagent (Invitrogen). Subsequently, cDNA synthesis was conducted with the Superscript II kit (Invitrogen) in accordance with the manufacturer’s instructions. Quantitative RT-PCR analysis was conducted using the SYBR Green PCR Master Mix (Takara). The thermal cycling conditions were set as follows: an initial denaturation at 95 °C for 10 s, followed by 35 cycles of 95 °C for 5 s and 60 °C for 30 s. Relative expressions were calculated assessed by the 2^-ΔCt^ method, using β-actin as an internal control. The primer sequences utilized in this investigation are provided in Supplementary Table S1.

### Western blot

Proteins were isolated from CRC cells using a Protein Extraction Kit (KeyGen Biotech). Following extraction, the protein samples underwent separation through SDS/PAGE and were subsequently transferred onto a PVDF membrane (Millipore). This was followed by a blocking step using a 5% nonfat milk solution. The membranes were then incubated with a primary antibody specific to SLC44A2 (Aviva Systems Biology, #ARP44009_P050), CPT2 (Proteintech, #26555-1-AP), anti-MUL1 (Proteintech, #16133-1-AP), Ac-K (Proteintech, #66289-1-AP), Ub (Proteintech, 10201-2-AP), β-actin (Proteintech, #66009-1-ig) and secondary antibodies. The results were detected using a chemiluminescence detection system.

### Immunohistochemistry (IHC) analysis

Tissue sections underwent a rehydration process followed by boiling in citrate buffer at a pH of 6.0 to facilitate antigen retrieval. Subsequently, the sections were treated with 3% hydrogen peroxide to inhibit endogenous peroxidase activity. Following this, the sections were incubated with primary antibodies against SLC44A2, CPT2, MUL1 or Ki-67. The results were semi-quantitatively analyzed under a light microscope, where the staining intensity was assessed on a scale (0 indicating negative, 1 for weak, 2 for moderate, and 3 for intense) and the proportion of stained area was categorized ( <5% as 0; 5–25% as 1; 26–50% as 2; 51–75% as 3; and 76–100% as 4). The median score was established as the threshold for distinguishing between high and low expression levels of SLC44A2.

### Cell proliferation assays

Cell proliferation was evaluated by both MTS and 5-ethynyl-20-deoxyuridine (EdU) assays. In MTS assay, CRC cells were seeded in 96-well plates at a density of 1×10^3^ cells/well. Then, MTS-PMS solution (Promega) was introduced to the cells and incubated for 30 minutes at specified time intervals at 37 °C. The absorbance was recorded at a wavelength of 490 nm.

In EdU assay, 1×10^5^ cells were cultured in 24-well plates for 2 days. Following this, 250 mM EdU (RiboBio, China) was administered to the cells and incubated for 100 minutes at 37 °C. After fixation and permeabilization, Hoechst was added to stain the nuclei for 20 min. The staining was examined by a confocal microscopy.

### Colony formation assay

Colorectal cancer (CRC) cells were plated in 6-well plates at a density of 1×10^3^ cells/well and incubated 12 days. Following the incubation, the colonies were fixed using 4% formaldehyde for 5 minutes and subsequently stained with 1% crystal violet for 2 minutes. Finally, the number of colonies was quantified.

### Assessment of cell cycle and apoptosis

The assessment of cell cycle and apoptosis was conducted with the cell cycle detection kit (C6078) and the FITC apoptosis detection kit (Y6002) provided by US Everbright Biotech. The results were subsequently analyzed using a flow cytometry (Agilent) within 30 min period.

### Assessment of Cell migration and invasion

Transwell assays without or with matrigel were used for the evaluations of cell migration and invasion, respectively. Briefly, 3×10^4^ or 6×10^4^ CRC cells were plated onto the upper chamber. The culture medium in lower containing chamber contains 15% FBS. Following a culture period of 24 or 48 hours, the cells that had migrated or invaded to the lower surface were subsequently fixed, stained, and quantified.

### Tumor xenograft models

Male BALB/c nude mice, aged between 4 and 6 weeks were used in this study. Animals were randomly separated into groups (6 mice per group) and maintained under a controlled environment with 12-h light-dark cycles with unrestricted access to food and water 25 ± 1 °C. The animal experimentation was conducted in accordance with the Guidelines for the Care and Use of Laboratory Animals (NIH publication, revised 2011) and approved by the animal ethics committee of the Air Force medical university (Approval No. 20210353).

For tumor growth, 6×10^6^ stable SLC44A2 overexpression or control CRC cells were subcutaneously injected into the left or right flanks of the same nude mice. Tumor size was recorded weekly. Following a four-week period post cells injection, the mice were euthanized. Subcutaneous tumors were removed, weighed and subjected to immunohistochemistry analysis.

For the construction of in vivo lung metastasis model, 3×10^6^ stable SLC44A2 overexpression or control CRC cells were administered via the tail vein. Five weeks after the injection, the mice were euthanized and lung tissues were harvested for subsequent Hematoxylin and Eosin (H&E) staining.

### Untargeted metabolomics analysis

SLC44A2 overexpression or control HCT116 cells were washed by PBS and subsequently frozen swiftly in liquid nitrogen. Following this, cells were harvested into 1.5 mL Eppendorf tubes for metabolite analysis utilizing liquid chromatography-mass spectrometry (LC-MS). Data were preprocessed under the criteria of RSD. Different metabolites between SLC44A2 overexpression and control HCT116 cells were identified through the application of the orthogonal partial least squares discriminant analysis (OPLS-DA) method.

### Glucose uptake and lactate production

The levels of glucose and lactate in cell culture medium were evaluated by a commercial glucose detection (#A154-1-1) or lactate detection (#A019-2-1) kit from the Nanjing Jiancheng Bioengineering Institute.

For glucose uptake, standard glucose solution was prepared. Then, 5 µL of sample or standard and 250 µL of the working solution were added the 96-well plate and incubated at 37 °C for 10 min. Absorbance was measured at 505 nm using a microplate reader. The results were determined using the standard curve and were normalized to protein content.

For lactate production, standard lactate solution was prepared. Then, 5 µL of sample or standard, 250 µL of the working solution and chromogen reagent were added to the 96-well plate and incubated at 37 °C for 10 min. Absorbance was measured at 530 nm using a microplate reader. The results were determined using the standard curve and normalized to protein content.

### Evaluations of ECAR and OCR

The XF96 Extracellular Flux Analyzer (Seahorse Bioscience) employed for determinations of extracellular acidification rate (ECAR) and oxygen consumption rate (OCR). A total of 1.0 × 10^4^ cells were seeded onto the XF96 plate. Prior to the measurements, the cell culture medium was replaced with XF medium one hour in advance. Glycolytic ability was measured with an XF Glycolysis Stress Test Kit according to the manufacturer’s instruction. Mitochondrial OCR was quantified using the XF Cell Mito Stress Test Kit following the manufacturer’s protocol.

### Determination of mitochondrial OXPHOS complexes activity

A commercial kit from Abcam (ab110419) was used to detect the activities of mitochondrial oxidative phosphorylation (OXPHOS) complexes (I-V). The experiments were performed in accordance with the guidelines provided by the manufacturer.

### Detection of ATP content, ROS content and mitochondrial membrane potential

The content of cellular ATP and reactive oxygen species (ROS) were assessed using the ATP colorimetric/fluorometric assay kit (abcam, ab83355) and fluorescent probe DCFH-DA (Beyotime Biotechnology, S0033), following the protocols provided by the manufacturers. The obtained results were normalized to the corresponding protein concentrations. JC-1 dye (Beyotime Biotechnology, C2006) was used to evaluate the mitochondrial membrane potential.

### Assessment of mitochondrial mass and mtDNA content

Mitochondrial mass was evaluated using the MitoTracker green fluorescent dye (Molecular Probes, M7514) in accordance with the guidelines provided by the manufacturer. The staining results were examined using a confocal microscopy (Olympus). Relative mtDNA content was quantified employing a quantitative polymerase chain reaction (qPCR) technique, normalizing the MT-ND1 gene expression to that of the nuclear HGB gene.

### Determination of NADP^+^/NADPH ratio

The NADP^+^/NADPH ratio in lysates of CRC cells was determined with the NADP^+^/NADPH Quantitation Kit (Abcam), following the protocols provided by the manufacturer. Briefly, CRC cells were lysed and supernatants were collected by centrifugation. Then, add samples (or standards) and reaction mix to 96-well plate 28 °C for 5 min. Colorimetric measurements were performed at wavelength of 450 nm using a Hidex Sense 96-wells plate reader.

### Immunoprecipitation

CRC cells were lysed in Immunoprecipitation (IP) lysis buffer (Thermo) supplemented with protease and phosphatase inhibitors. The cell lysates were then centrifuged at 13,000 × g for 15 min at 4 °C. The supernatants were collected and protein concentrations were assessed using the BCA Protein Assay Kit (Thermo Fisher Scientific). For immunoprecipitation, 1 mg of protein sample was incubated with 5 µl of anti-CPT2 (Proteintech, #26555-1-AP) or anti-MUL1 (Proteintech, #16133-1-AP) or IgG (Proteintech, #11541-1-AP) overnight at 4 °C with gentle rotation. Subsequently, 50 µL of protein A/G magnetic beads (Millipore Sigma) was added and incubated for 2 h at 4 °C. After three times washes of the beads with ice-cold IP lysis buffer, the immunoprecipitated proteins were boiled in 2× SDS-PAGE loading buffer for 6 minutes at 95 °C and subjected to Western blot analysis.

### Statistical analysis

**Fig. 1:**
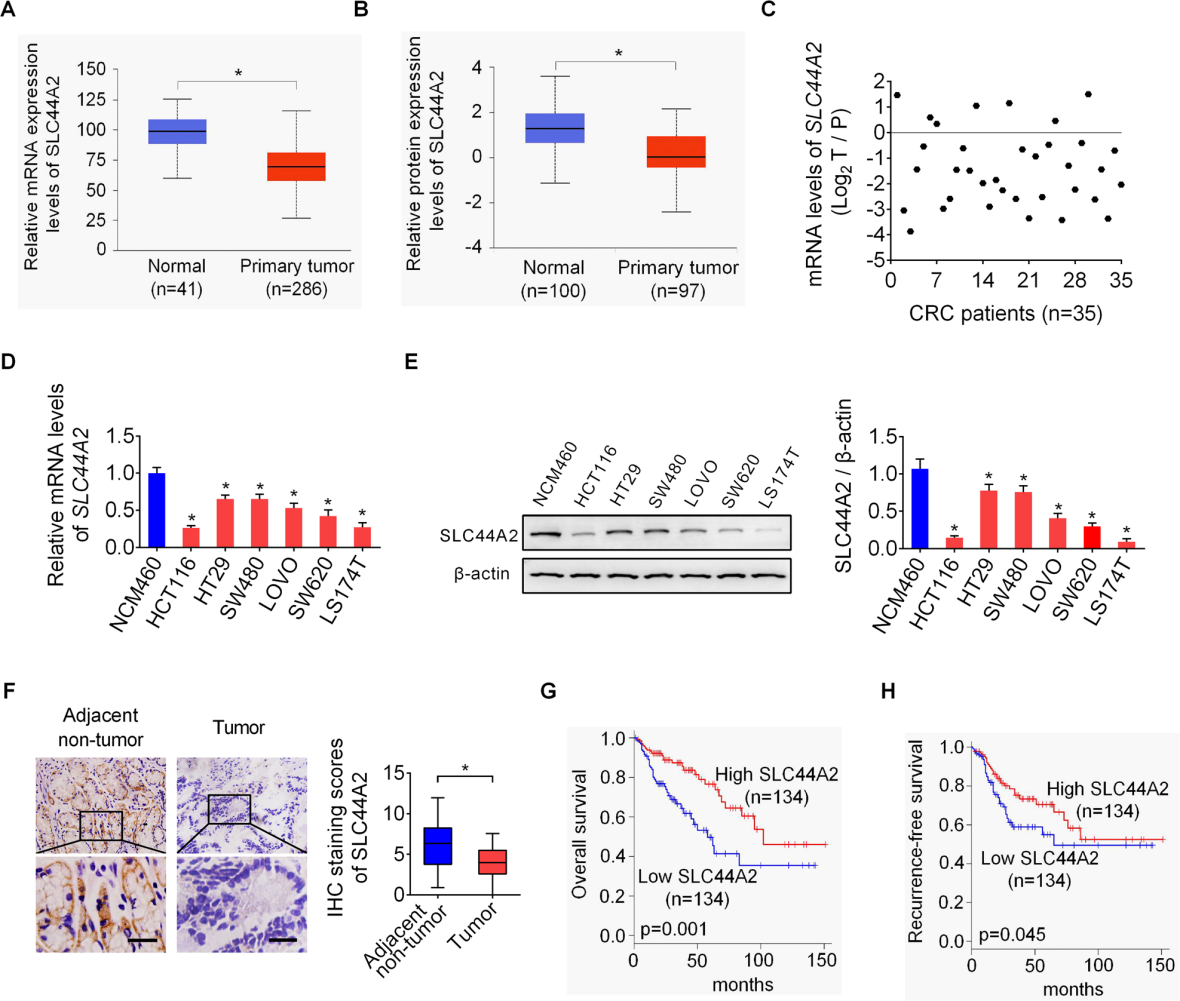
SLC44A2 is markedly downregulated in CRC and low SLC44A2 expression is closely associated with poorer patient survival. **A**, **B** The expression levels of SLC44A2 mRNA (**A**) and protein (**B**) in CRC specimens and paired non-tumor tissues were analyzed in the online UALCAN database. **C** qRT-PCR analysis of SLC44A2 expression in 35 paired CRC samples and their adjacent non-tumor counterparts. **D**, **E** Both qRT-PCR and western blot assays were used to detect SLC44A2 expression in CRC and normal colonic epithelial cell lines. **F** Immunohistochemistry (IHC) analysis of SLC44A2 in an expanded 268 pairs of CRC specimens and their matched adjacent non-tumor tissues. Scale bars, 10 μm. **G**, **H** Kaplan–Meier analysis of overall (**G**) and recurrence-free (**H**) survival rates among for CRC patients with high or low SLC44A2 expression (*n* = 268). The figure was generated using GraphPad Prism software version 7.0.

For the statistical evaluations, GraphPad Prism software version 7.0 (GraphPad Software, La Jolla, USA) was employed. Experiments were performed at least three times (*n* = 3) unless otherwise stated and the results were presented as mean ± standard deviation (SD). Comparisons between two or more groups were conducted using the two-tailed unpaired (two-tailed paired only used in Fig. 1C) Student’s t-test or one-way ANOVA with Tukey’s post-hoc tests. Correlation analysis was performed using the Pearson method. Patient survival was compared using the Kaplan–Meier method. Differences or correlations were defined as statistically significant at *P* < 0.05.

## Results

### SLC44A2 is markedly downregulated in CRC and low SLC44A2 expression is closely associated with poorer patient survival

We first examined the expression of SLC44A2 in colorectal cancer (CRC) using the UALCAN database. The results indicated that both mRNA and protein levels of SLC44A2 were significantly downregulated in CRC tumor tissues compared to normal tissues (Fig. 1A and B). Detection of SLC44A2 expression by qRT-PCR analysis in 35 pairs of human CRC tissues and adjacent non-tumor tissues also confirmed the down-regulation of SLC44A2 in CRC (Fig. 1C). To validate these findings, we tested SLC44A2 expression in CRC cell lines and normal colonic epithelial cells using qRT-PCR and Western blot analysis. As shown in Fig. 1D and E, SLC44A2 expression was significantly lower in CRC cell lines than in normal colonic epithelial cells. Additionally, immunohistochemical (IHC) staining of an expanded cohort of 268 CRC patients showed significantly reduced SLC44A2 expression in CRC tissues compared to adjacent normal colon tissues (Fig. 1F).

Next, we investigated the clinical significance of SLC44A2 in CRC. Correlation analysis revealed that SLC44A2 downregulation was significantly associated with larger tumor size and higher frequency of lymph node metastasis (Table S2). Kaplan-Meier survival analysis demonstrated that CRC patients with low SLC44A2 expression had significantly poorer survival rates and higher recurrence rates compared to those with high SLC44A2 expression (Fig. 1G and H).

To further elucidate the role of SLC44A2 in different human cancer types, we conducted a pan-cancer analysis using Sangerbox 3.0. Among the 26 TCGA cancer types, SLC44A2 was significantly downregulated in lung adenocarcinoma (LUAD), lung squamous cell carcinoma (LUSC), renal papillary cell carcinoma (KIRP), renal clear cell carcinoma (KIRC), prostate adenocarcinoma (PRAD), and head and neck squamous cell carcinoma (HNSC) (Fig. S1A). Conversely, SLC44A2 was significantly upregulated in glioblastoma multiforme (GBM), lower-grade glioma (LGG), cervical squamous cell carcinoma and endocervical adenocarcinoma (CESC), invasive breast carcinoma (BRCA), gastric and esophageal carcinoma (STES), gastric adenocarcinoma (STAD), uterine corpus endometrial carcinoma (UCEC), liver hepatocellular carcinoma (LIHC), thyroid carcinoma (THCA), pheochromocytoma and paraganglioma (PCPG), bladder urothelial carcinoma (BLCA), and cholangiocarcinoma (CHOL) (Fig. S1B). Kaplan-Meier survival analysis showed that patients with lower SLC44A2 expression had worse overall survival (OS) in renal clear cell carcinoma (KIRC), pan-kidney cohort (KIPAN), glioma (GBMLGG), head and neck squamous cell carcinoma (HNSC), and skin cutaneous melanoma (SKCM) (Fig. S1C). In contrast, patients with lower SLC44A2 expression in pancreatic adenocarcinoma (PAAD), invasive breast carcinoma (BRCA), and adrenocortical carcinoma (ACC) had better OS than those with higher SLC44A2 expression (Fig. S1D). These results suggest that SLC44A2 is closely associated with human cancers.

### SLC44A2 impairs proliferation and invasion of CRC cells both in vitro and in vivo

**Fig. 2:**
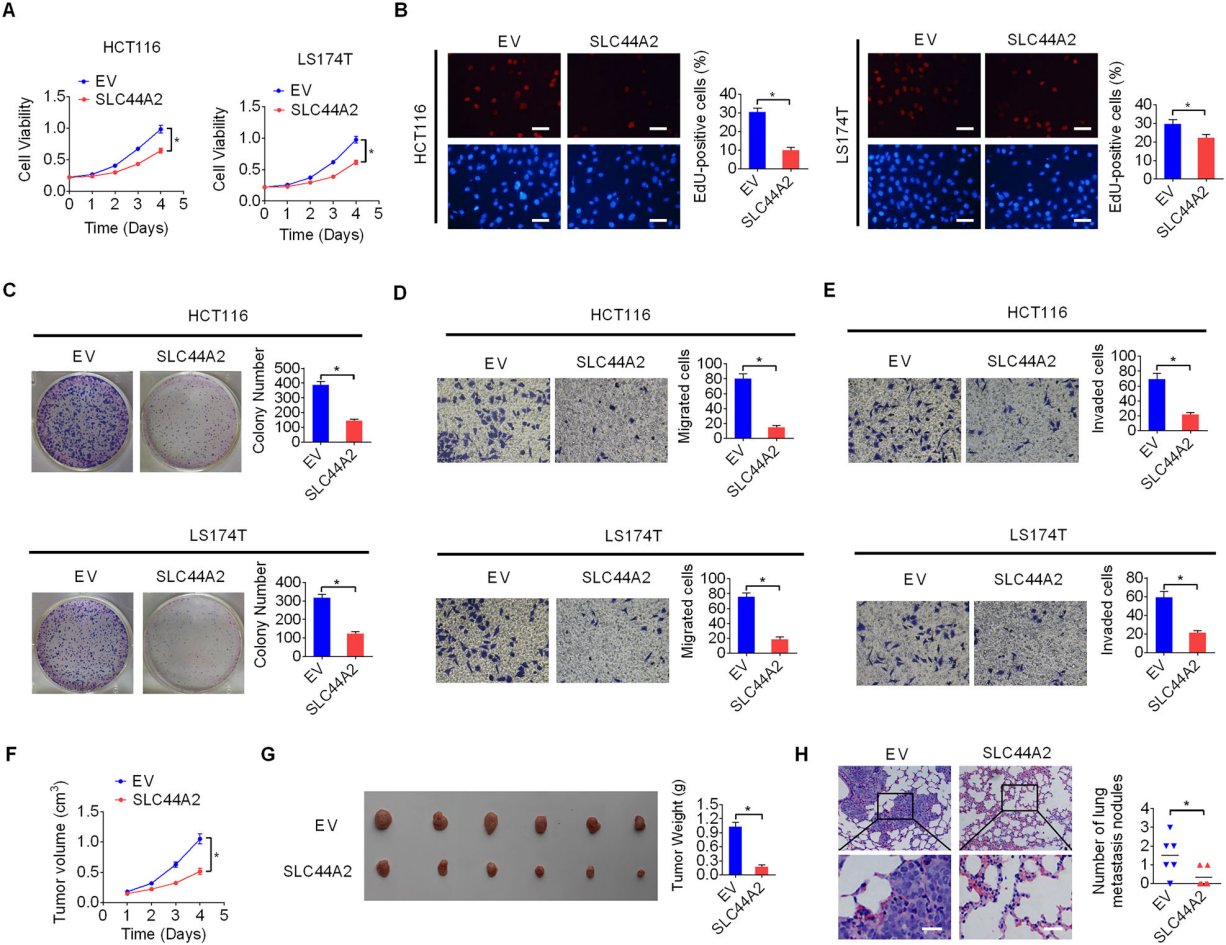
SLC44A2 impairs proliferation and invasion of CRC cells both in vitro *and* in vivo*.* **A**–**C** Short- and long-term cell proliferations were examined by MTS (**A**), EDU (**B**, Scale bars=50 μm) and colony formation (**C**) assays in HCT116 and LS174T cells. **D**, **E** The migratory (**D**) and invasive (**E**) capabilities of HCT116 and LS174T cells were examined by transwell assays. **F** Growth of xenografts developed from HCT116 cells with forced SLC44A2 expression (SLC44A2) or empty vector (EV). **G** The original tumors (left) and tumor weight (right) from the nude mice are shown. **H** H&E staining of the lungs from SLC44A2 and EV mice for the incidences of lung metastases. Scale bars, 10 μm. The figure was generated using GraphPad Prism software version 7.0.

Since low SLC44A2 expression was associated with poor survival in CRC patients, we subsequently examined the functional role of SLC44A2 in CRC progression. Based on the results shown in Fig. 1D and E, we overexpressed SLC44A2 in HCT116 and LS174T cells with low endogenous SLC44A2 expression (Fig. S2A and S2B). MTS cell viability and colony formation assays indicated that upregulation of SLC44A2 significantly suppressed the proliferation of HCT116 and LS174T cells (Fig. 2A–C). Since cell proliferation is mainly determined by both cell cycle progression and apoptosis, we examined the effects of SLC44A2 on these processes. The results showed that SLC44A2 overexpression in HCT116 and LS174T cells significantly slowed cell cycle progression from G1 to S phase and increased apoptosis (Fig. S2C and S2D). Additionally, forced SLC44A2 expression significantly impaired the migratory and invasive capabilities of HCT116 and LS174T cells, as assessed by transwell assays (Fig. 2D and E).

Next, we investigated the effects of SLC44A2 on CRC proliferation and invasion in an in vivo model. Stable overexpression of SLC44A2 in HCT116 cells led to a significant decrease in xenograft tumor growth and weights compared to the control group (Fig. 2F and G). Immunohistochemical (IHC) staining confirmed significant SLC44A2 upregulation in tumors from the SLC44A2-overexpressing group compared to the control group (Fig. S2E), indicating that SLC44A2 overexpression inhibited tumor growth. Consistent with the in vitro findings, a reduced number of proliferating cells and an increased percentage of apoptotic cells were observed in SLC44A2-overexpressing tumors compared to control tumors from nude mice (Fig. S2F and S2G). In addition, lung metastasis assay demonstrated that overexpression of SLC44A2 markedly decreased the number of metastatic nodules in the lungs of nude mice (Fig. 2H). Collectively, these findings indicate that SLC44A2 acts as a crucial tumor suppressor in CRC progression.

To provide more support for the tumor-suppressive function of SLC44A2 in CRC, SLC44A2 expression was knocked-down in HT29 and SW480 cells with higher endogenous SLC44A2 expression (as shown in Fig. 1D and E). Successful knockdown of SLC44A2 was confirmed by qRT-PCR and Western blot analysis (Fig. S3A and S3B). MTS, EDU and colony formation assays demonstrated that knockdown of SLC44A2 markedly enhanced the short- and long-term proliferation of HT29 and SW480 cells (Fig. S3C–S3E). Moreover, Knockdown of SLC44A2 significantly promoted the migratory and invasive capabilities of HT29 and SW480 cells (Fig. S3F and S3G).

### SLC44 A2 suppresses mitochondrial energy metabolism and increases ROS stress in CRC cells

**Fig. 3:**
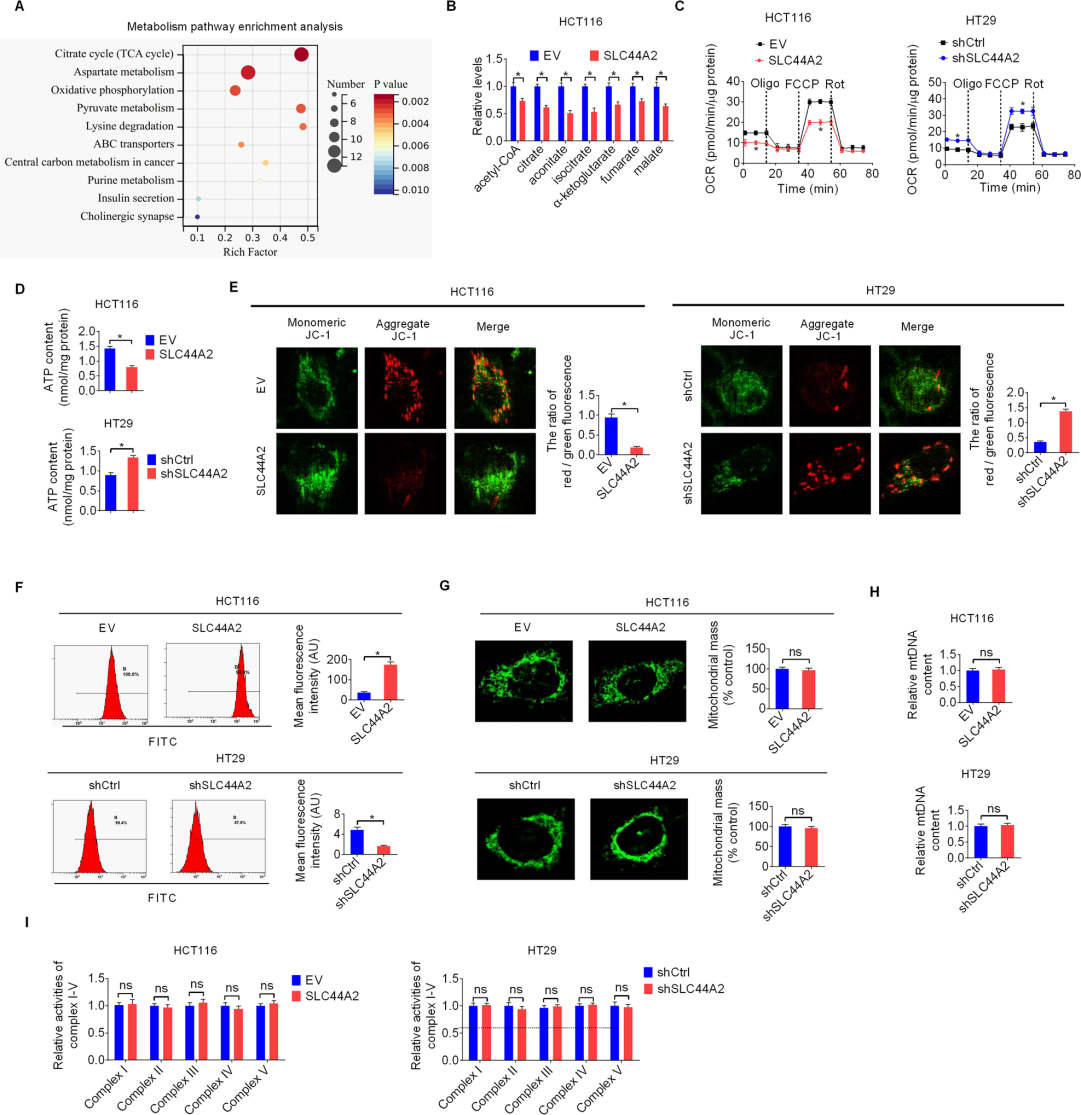
SLC44A2 suppresses mitochondrial energy metabolism and increases ROS stress in CRC cells. **A** Pathway enrichment of significant changed metabolites by SLC44A2 overexpression in HCT116 cells. **B** The concentrations of intermediates within the TCA cycle in SLC44A2-overexpressing and control HCT116 cells were compared. **C** The oxygen consumption rate (OCR) was assessed with a Seahorse XF24e Extracellular Flux Analyzer in SLC44A2 overexpression or knockdown CRC cells. **D** ATP levels were determined in SLC44A2 overexpression or knockdown CRC cells. **E** Confocal-based detection of mitochondrial membrane potential by JC-1 fluorescence staining assay in SLC44A2 overexpression or knockdown CRC cells. **F** Flow-cytometry-based measurement of ROS content in SLC44A2 overexpression or knockdown CRC cells. **G**–**I** Quantification of mitochondrial mass (**G**), mtDNA content (**H**) and activities of oxidative phosphorylation complexes (**I**) were evaluated in SLC44A2 overexpression or knockdown CRC cells. The figure was generated using GraphPad Prism software version 7.0.

We next explored the mechanisms by which downregulation of SLC44A2 promotes the proliferation and invasion of CRC cells. Given that SLC44A2 is a choline transporter involved in regulating mitochondrial function [11], the effect of SLC44A2 overexpression on cell metabolic changes were determined in HCT116 cells. Untargeted metabolome analysis revealed that that SLC44A2-regulated metabolites were primarily enriched in energy metabolism pathways, including the “TCA cycle,” “oxidative phosphorylation,” and “central carbon metabolism in cancer” (Fig. 3A). Specifically, intermediates within the TCA cycle were significantly increased upon SLC44A2 overexpression (Fig. 3B), highlighting its crucial role in mitochondrial metabolism in CRC cells.

To evaluate the impact of SLC44A2 on mitochondrial metabolism, we measured mitochondrial oxidative phosphorylation activity and ATP production in CRC cells with SLC44A2 overexpression or knockdown. In SLC44A2-overexpressing HCT116 cells, we observed a marked reduction in oxygen consumption rate (OCR) and ATP production (Fig. 3C). Conversely, these mitochondrial respiratory phenotypes were significantly enhanced in SLC44A2 knockdown HT29 cells (Fig. 3D). Consistent with these findings, mitochondrial membrane potential was significantly reduced or elevated upon SLC44A2 overexpression or knockdown, respectively (Fig. 3E).

Notably, reactive oxygen species (ROS) levels were significantly increased by SLC44A2 overexpression (Fig. 3F), contrary to our initial hypothesis that suppressing mitochondrial oxidative metabolism would decrease ROS production in CRC cells. Unlike the changes in mitochondrial respiration, no significant changes in mitochondrial mass, mtDNA content, and oxidative phosphorylation (OXPHOS) complex activities were observed after either SLC44A2 overexpression or knockdown (Fig. 3G–I). These results suggest that SLC44A2 regulates mitochondrial metabolism in CRC cells not by altering mitochondrial mass or OXPHOS complex activities.

### SLC44A2 suppresses mitochondrial energy metabolism and increases ROS stress by inhibiting fatty acid oxidation

**Fig. 4:**
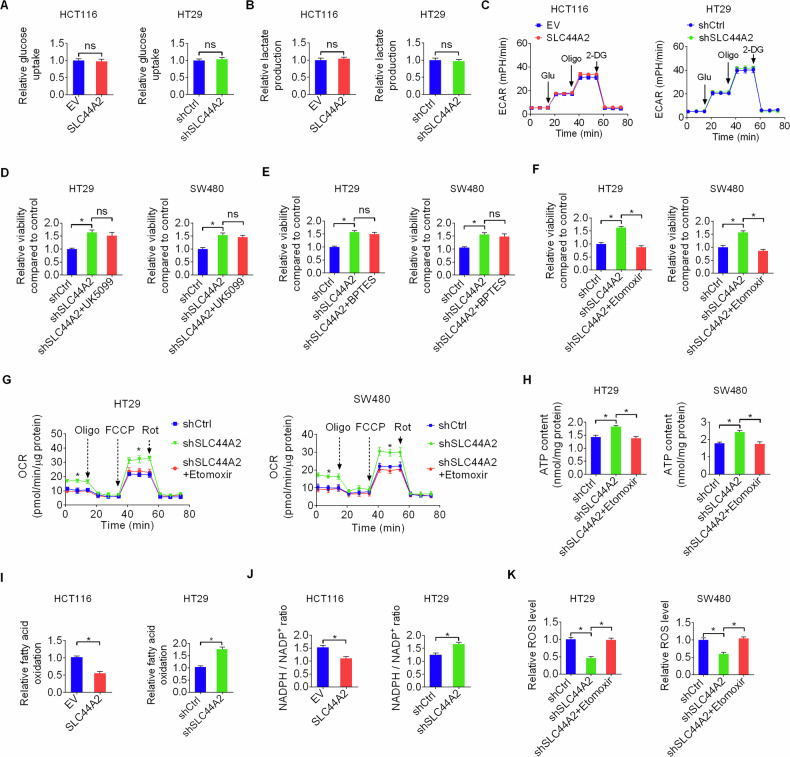
SLC44A2 suppresses mitochondrial energy metabolism and increases ROS stress by inhibiting fatty acid oxidation. **A** Glucose uptake was assessed in SLC44A2 overexpression or knockdown CRC cells. **B** Lactate production was assessed in SLC44A2 overexpression or knockdown CRC cells. **C** Assessment of extracellular acidification rate (ECAR) using a Seahorse XF24e Extracellular Flux Analyzer in SLC44A2 overexpression or knockdown CRC cells. **D**–**F** MTS assay was conducted in CRC cells after treatment with UK5009 (**D**, 20 nM), BPTES (**E**, 100 nM) and Etomoxir (**F**, 100 nM) for 72 h. **G**, **H** The levels of OCR (**G**) and ATP (**H**) were determined in CRC cells with indicated treatment. **I** Fatty acid oxidation (FAO) was evaluated in SLC44A2 overexpression or knockdown CRC cells. **J** The ratio of NADPH/NADP^+^ was determined in SLC44A2 overexpression or knockdown CRC cells. **K** The concentration of ROS was assessed in CRC cells with indicated treatment. The figure was generated using GraphPad Prism software version 7.0.

Since glucose is a major source for mitochondrial metabolism, we evaluated glucose uptake and lactate production to assess the impact of SLC44A2 on glucose metabolism in CRC cells. No significant changes in glucose uptake or lactate production were observed following SLC44A2 overexpression or knockdown (Fig. 4A and B). Consistent with this, extracellular acidification rate (ECAR) remained unchanged in CRC cells with SLC44A2 overexpression or knockdown (Fig. 4C).

To identify the energy source driving the enhanced mitochondrial metabolism caused by SLC44A2 knockdown, we treated control and SLC44A2-knockdown CRC cells with specific inhibitors targeting glycolysis, fatty acid oxidation (FAO), and glutaminolysis. Inhibition of glycolysis by UK5099 or glutaminolysis by BPTES did not significantly affect cell proliferation enhanced by SLC44A2 knockdown (Fig. 4D and E). In contrast, inhibition of FAO by etomoxir significantly impaired CRC cell proliferation promoted by SLC44A2 knockdown (Fig. 4F). Consistent with these findings, increased oxygen consumption rate (OCR) and ATP production caused by SLC44A2 knockdown were markedly attenuated by etomoxir treatment (Fig. 4G and H). These results suggest that SLC44A2 primarily suppresses mitochondrial metabolism by inhibiting FAO.

Further support for this conclusion came from measurements of FAO rates, which showed that FAO was significantly decreased by SLC44A2 overexpression and increased by SLC44A2 knockdown in CRC cells (Fig. 4I). Given that FAO is a key source of mitochondrial NADPH, which is required to quench ROS, we hypothesized that SLC44A2-regulated FAO might affect redox homeostasis in CRC cells. Indeed, the intracellular NADPH/NADP+ ratio was significantly decreased by SLC44A2 overexpression and increased by SLC44A2 knockdown (Fig. 4J). Moreover, the increased ROS levels caused by SLC44A2 knockdown could be rescued by inhibition of FAO with etomoxir treatment (Fig. 4K). This suggests that SLC44A2 downregulation facilitates redox homeostasis by enhancing FAO-mediated NADPH production in CRC cells.

### SLC44A2 downregulates CPT2 expression by enhancing ubiquitin-mediated proteasomal degradation of CPT2 via reducing its acetylation

**Fig. 5:**
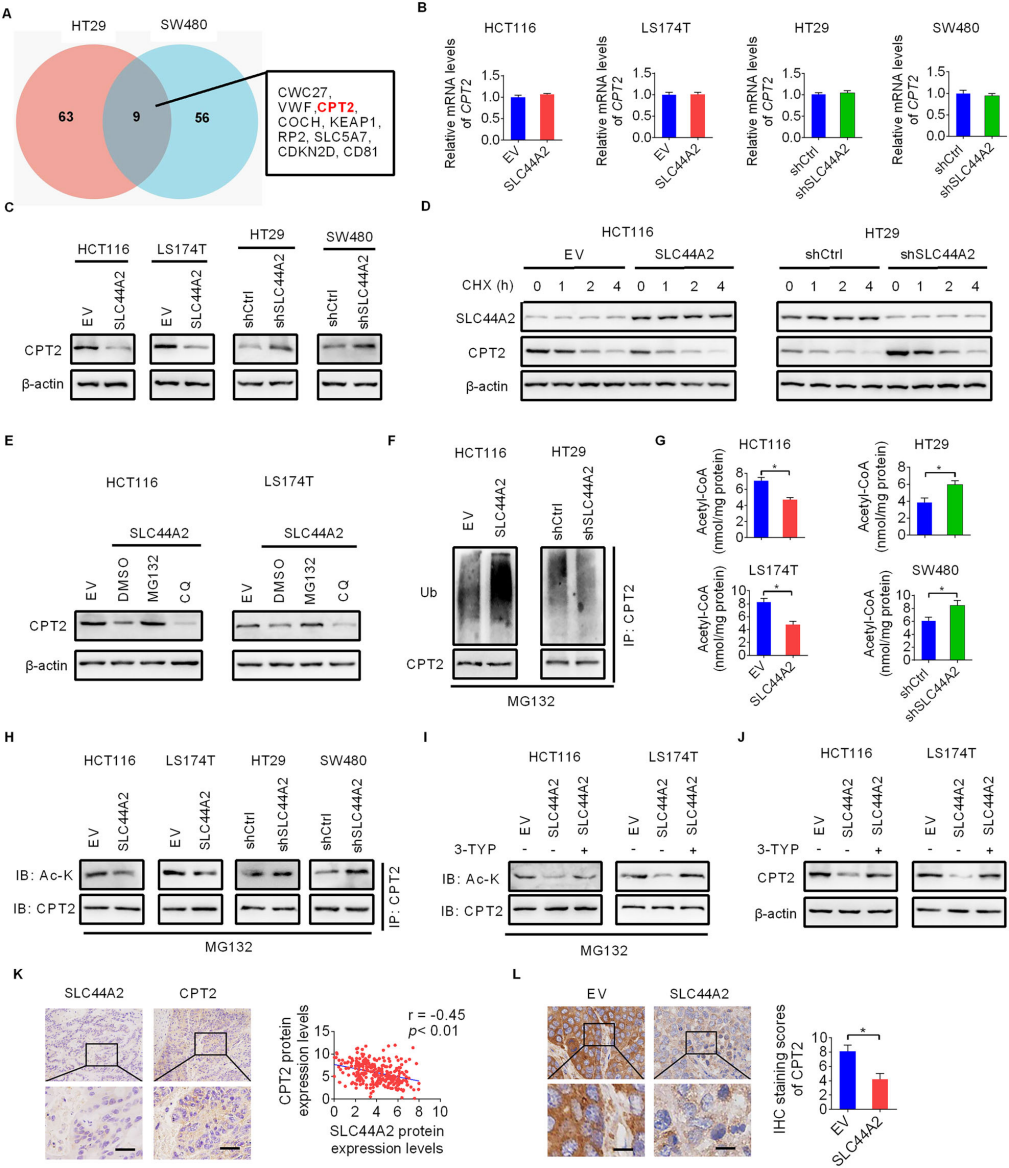
SLC44A2 downregulates CPT2 expression by enhancing ubiquitin-mediated proteasomal degradation of CPT2 via reducing its acetylation. **A** Venn diagram analyses for differently expressed proteins (more than 3-fold expression change and *p* < 0.01) revealed by mass spectrometry-based proteomic analysis in CRC cells with SLC44A2 overexpressed or knocked-down. **B**, **C** The expression levels of CPT2 were assessed using qRT-PCR (**B**) and Western blot (**C**) assays in CRC cells with either overexpressed or silenced SLC44A2. **D** The expressions of SLC44A2 and CPT2 were assessed by western blot analysis in HCT116 cells and HT29 cells with CHX (10 μM) treatment. **E** Western blot analysis for the effects of MG132 (20 μM for 8 hours) or CQ (20 μM for 12 hours) treatment on SLC44A2-regulated CPT2 expression in CRC cells. **F** Western blot analysis for the effects of SLC44A2 overexpression and knockdown on the ubiquitination of CPT2 in CRC cells. **G**, **H** The levels of acetyl-CoA (**G**) and acetylation of CPT2 (**H**) were determined in CRC cells with SCL44A2 overexpressing or knocking-down. **I**, **J** Western blot analysis for the acetylation (**I**) and expression (**J**) of CPT2 in SCL44A2 overexpressing CRC cells treated with 3-TYP (the inhibitor of SIRT3) with or without MG132. **K** Immunohistochemistry staining assay for correlation between the expressions of SLC44A2 and CPT2 in tumor tissue samples obtained from CRC patients. **L** IHC staining of CPT2 in tumors from the nude mice of SLC44A2 and EV groups from. Scale bars, 10 μm. The figure was generated using GraphPad Prism software version 7.0.

We next explored the detailed molecular mechanisms by which SLC44A2 suppresses fatty acid oxidation (FAO) using mass spectrometry-based proteomic analysis. We identified nine proteins whose expression levels changed markedly (more than 3-fold, *p* < 0.01) in CRC cells with SLC44A2 overexpression or knockdown (Fig. 5A). We focused on carnitine palmitoyltransferase 2 (CPT2), a key rate-limiting enzyme in FAO. qRT-PCR and Western blot analyses revealed that SLC44A2 overexpression significantly reduced CPT2 protein levels, while CPT2 mRNA levels remained unchanged. Conversely, SLC44A2 knockdown increased CPT2 protein expression in CRC cells (Fig. 5B and C), suggesting that SLC44A2 regulates CPT2 at the post-translational level. To further investigate whether SLC44A2 affects CPT2 stability, we treated CRC cells with cycloheximide (CHX) to inhibit protein synthesis. As shown in Fig. 5D, SLC44A2 overexpression significantly shortened the half-life of CPT2 in HCT116 cells, while SLC44A2 knockdown extended it in HT29 cells, indicating that SLC44A2 promotes CPT2 degradation in CRC cells. The ubiquitin-proteasome pathway and the autophagy-lysosomal pathway are the primary mechanisms responsible for intracellular protein degradation. Treatment with MG132 (a proteasome inhibitor) restored the decreased CPT2 levels caused by SLC44A2 overexpression, while treatment with chloroquine (CQ, an autophagy-lysosomal inhibitor) had no such effect (Fig. 5E). Consistent with this, CPT2 ubiquitination was increased by SLC44A2 overexpression and decreased by SLC44A2 knockdown (Fig. 5F). These results indicate that SLC44A2 downregulates CPT2 expression by enhancing its ubiquitin-proteasomal degradation.

CPT2 has been reported to be regulated by acetylation [13], which is crucial for modulating both protein activity and stability [14–16]. Given that SLC44A2 downregulates the key metabolite acetyl-CoA (as indicated in Fig. 3B), a major donor of acetyl groups for protein acetylation [17], we determined the effect of SLC44A2 on CPT2 acetylation. Using an ELISA assay, we confirmed the negative regulation of acetyl-CoA levels by SLC44A2 in CRC cells, as shown in the untargeted metabolome analysis (Fig. 5G). Additionally, we found that CPT2 acetylation was significantly decreased upon SLC44A2 overexpression and increased upon SLC44A2 knockdown (Fig. 5H).

To further investigate whether SLC44A2 downregulates CPT2 expression by decreasing its acetylation, we treated SLC44A2-overexpressing CRC cells with 3-TYP, a known inhibitor of SIRT3, which is a critical deacetylase of CPT2 [18, 19]. The results showed that 3-TYP treatment significantly restored the decreased acetylation and expression of CPT2 caused by SLC44A2 overexpression in HCT116 and LS174T cells (Fig. 5I and J). These findings suggest that SLC44A2 downregulates CPT2 expression by reducing its acetylation.

We also examined the association between CPT2 and SLC44A2 in tumor tissues from CRC patients using IHC staining-based histological analysis and found a notable inverse correlation between CPT2 and SLC44A2 protein levels (Fig. 5K). Consistent with this, IHC staining of CPT2 in tumor tissues from nude mice showed significantly lower CPT2 levels in the SLC44A2 overexpression group compared to the control group (Fig. 5L). Collectively, these data indicate that SLC44A2 downregulates CPT2 expression by reducing its acetylation, thereby promoting ubiquitin-proteasomal degradation in CRC cells.

### SLC44A2 promotes the degradation of CPT2 by enhancing the interaction between MUL1 and CPT2

**Fig. 6:**
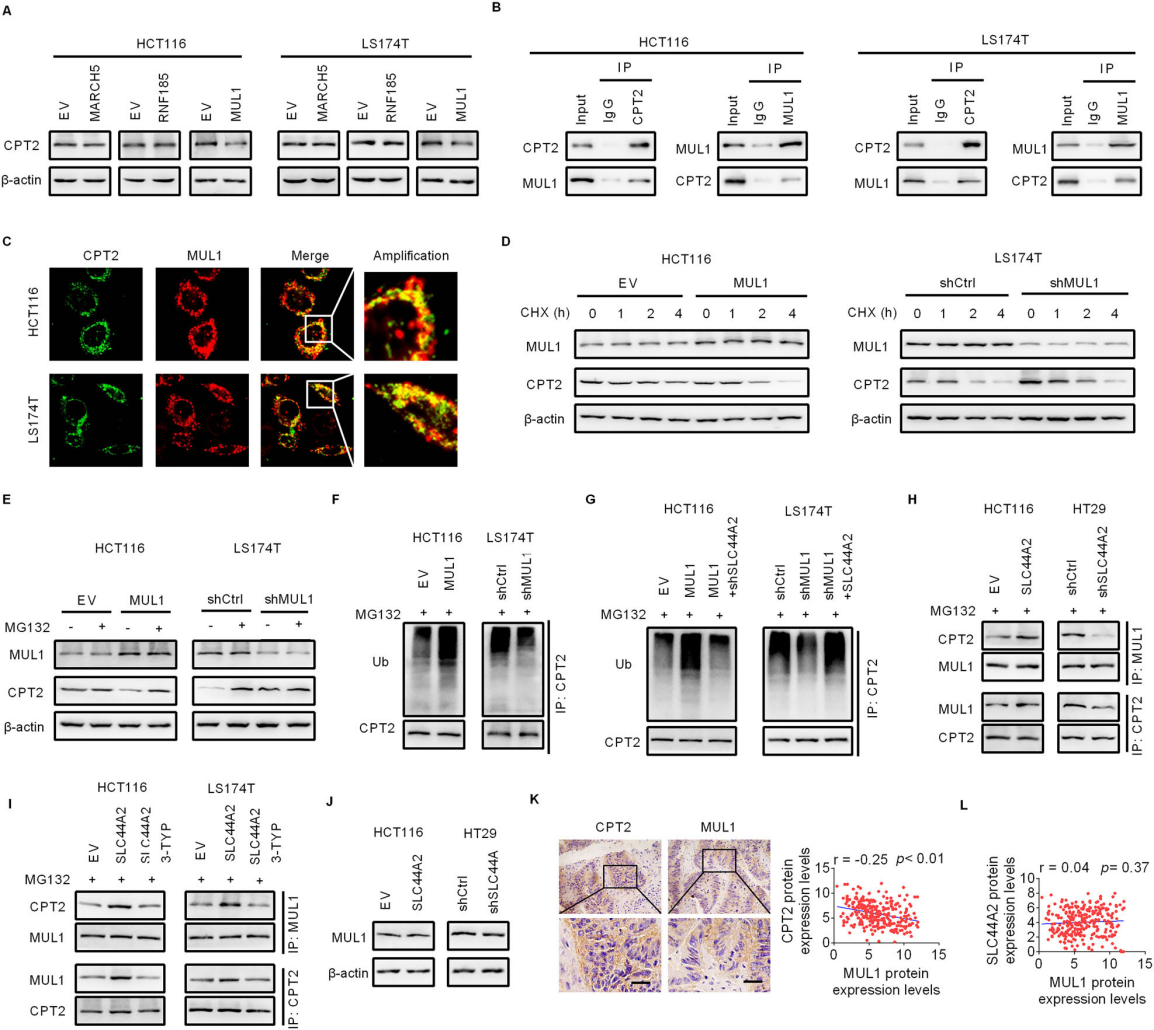
SLC44A2 promotes the degradation of CPT2 by enhancing the interaction between MUL1 and CPT2. **A** CPT2 expression was assessed by western blot analysis in CRC cells upon overexpression of MUL1, MARCH5 or RNF185. **B** Co-immunoprecipitation (Co-IP) assay was employed to assess the interaction between CPT2 and MUL1 in CRC cells. **C** Immunofluorescence (IF) assay was used to assess the localization of CPT2 and MUL1 in CRC cells. **D** The expressions of MUL1 and CPT2 were determined by western blot assay in CRC cells with CHX (10 μM) treatment. **E** The expressions of MUL1 and CPT2 were assessed by western blot assay in CRC cells with MG132 (20 μM) treatment. **F** Western blot analysis for the impact of MUL1 overexpression on the ubiquitination of CPT2 in CRC cells. **G** The ubiquitination of CPT2 was assessed by western blot assay in CRC cells with indicated treatment. **H** The effect of SLC44A2 on the interaction between MUL1 and CPT2 was assessed in CRC cells. **I** The effect of acetylation on SLC44A2-regulated interaction between MUL1 and CPT2 was determined in CRC cells. **J** The effect of SLC44A2 on the expression of MUL1 was assessed in CRC cells. **K** Immunohistochemistry staining assay for correlation between the expressions of MUL1 and CPT2 in CRC tissues. **L** Correlation between the expressions of SLC44A2 and MUL1 in CRC tissues was analyzed. The figure was generated using GraphPad Prism software version 7.0.

We next sought to identify the E3 ubiquitin ligase responsible for the ubiquitination and degradation of CPT2 in CRC cells. Given that CPT2 is localized in the mitochondrial membrane, we focused on three mitochondrial-specific E3 ligases: MARCH5, RNF185, and MUL1 [20]. Western blot analysis showed that CPT2 expression in CRC cells was downregulated by MUL1 overexpression but not by MARCH5 or RNF185 overexpression (Fig. 6A). Co-immunoprecipitation (Co-IP) and immunofluorescence (IF) assays indicated a direct interaction between CPT2 and MUL1 in CRC cells (Fig. 6B and C). As expected, the half-life of CPT2 was significantly shortened by MUL1 overexpression and extended by MUL1 knockdown (Fig. 6D). Additionally, the regulation of CPT2 expression by MUL1 was abolished by treatment with MG132 (Fig. 6E). Moreover, CPT2 ubiquitination was significantly enhanced by MUL1 overexpression and reduced by MUL1 silencing in CRC cells (Fig. 6F). These data indicate that MUL1 directly interacts with CPT2 to promote its ubiquitination and degradation in CRC cells.

We then explored whether MUL1-mediated ubiquitination of CPT2 could be regulated by SLC44A2 in CRC cells. The results showed that increased CPT2 ubiquitination by MUL1 overexpression was significantly reversed by SLC44A2 silencing (Fig. 6G). Conversely, decreased CPT2 ubiquitination by MUL1 knockdown was restored by SLC44A2 overexpression. Additionally, SLC44A2 overexpression increased the amount of CPT2-bound MUL1 and MUL1-bound CPT2, while SLC44A2 silencing decreased these interactions (Fig. 6H). Furthermore, the increased interaction between MUL1 and CPT2 caused by SLC44A2 overexpression was impaired by 3-TYP treatment, which elevates CPT2 acetylation (Fig. 6I). Notably, MUL1 expression levels remained unchanged regardless of SLC44A2 overexpression or knockdown (Fig. 6J). These findings suggest that SLC44A2 downregulates CPT2 expression by enhancing MUL1-mediated degradation through increased interaction between MUL1 and CPT2, rather than by upregulating MUL1 expression. Immunohistochemical (IHC) staining revealed a negative correlation between MUL1 and CPT2 expression levels (Fig. 6K). However, no significant correlation was observed between SLC44A2 and MUL1 expression levels (Fig. 6L). Consistent with this, analysis using the UALCAN database showed no significant change in MUL1 protein expression in CRC tumor tissues compared to normal tissues (Fig. S4).

### SLC44A2 suppresses CRC proliferation and invasion by inhibiting CPT2-regulated mitochondrial FAO

**Fig. 7:**
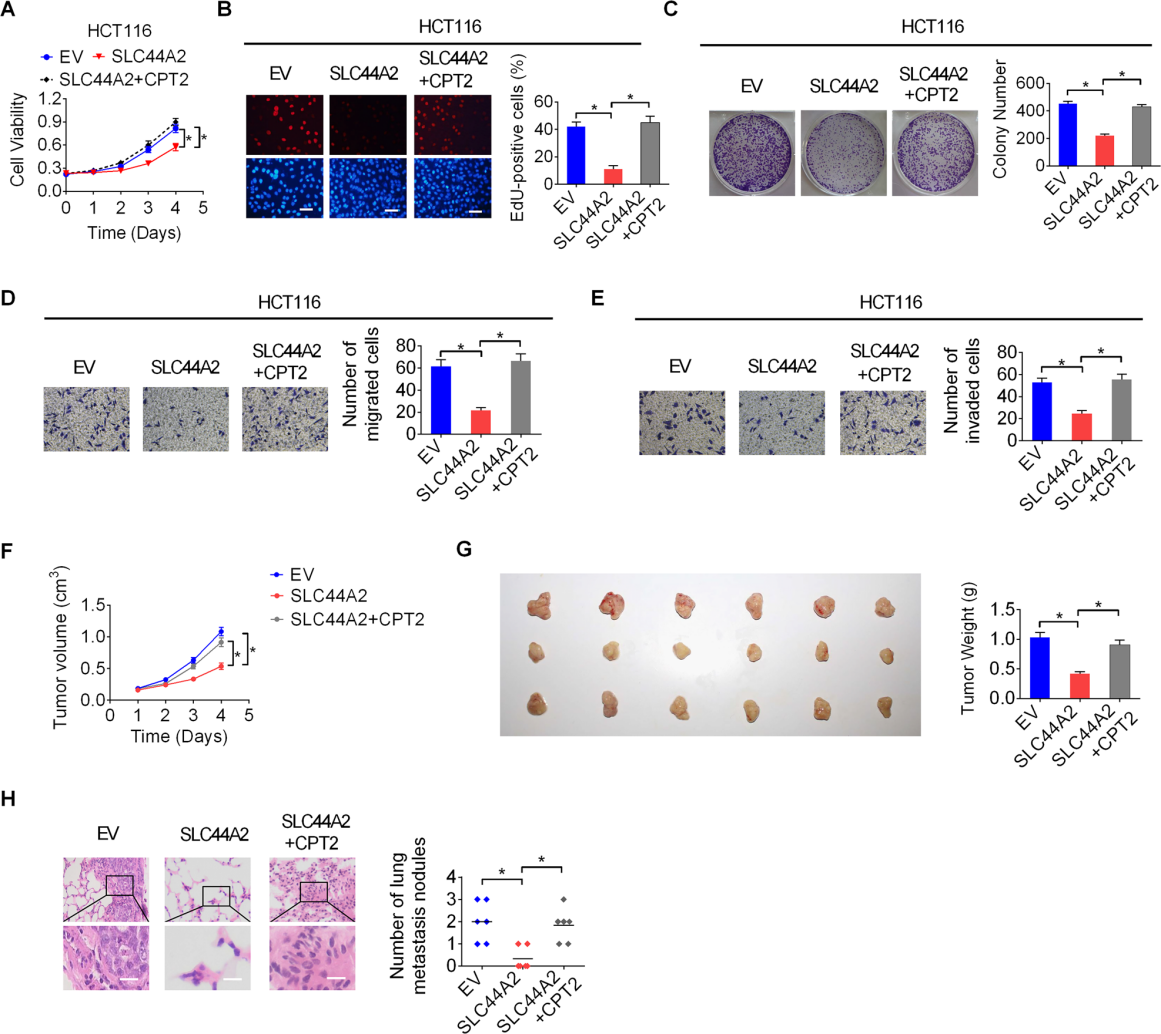
SLC44A2 suppresses CRC proliferation and invasion by inhibiting CPT2-regulated mitochondrial FAO. **A**–**C** Short- and long-term cell proliferations were examined by MTS (**A**), EdU (**B**) and colony formation (**C**) assays in CRC cells with indicated treatment. **D**, **E** Cell migration (**D**) and invasion (**E**) abilities were examined by transwell assays in CRC cells with indicated treatment. **F** Growth of xenografts developed from indicated CRC cells. **G** The original tumors (left) and tumor weight (right) from the nude mice developed from indicated CRC cells. **H** H&E staining for the incidence of lung metastases in nude mice injected with indicated CRC cells. Scale bars, 10 μm. The figure was generated using GraphPad Prism software version 7.0.

Fatty acid β-oxidation (FAO) has been shown to be essential in the tumorigenesis of numerous malignancies [21]. We therefore investigated whether SLC44A2 suppresses CRC proliferation and invasion by inhibiting CPT2-regulated fatty acid oxidation. Our findings indicated that forced expression of CPT2 significantly rescued the suppression of cell proliferation and invasion in vitro, as well as tumor growth and metastasis in vivo, which were caused by SLC44A2 overexpression (Fig. 7A–H). Conversely, inhibiting FAO by etomoxir significantly impaired the proliferation (Fig. S5A–S5C) and metastasis (Fig. S5D and S5E) of CRC cells promoted by SLC44A2 knockdown. These results suggest that SLC44A2 suppresses CRC proliferation and invasion by inhibiting CPT2-regulated mitochondrial FAO.

To explore the potential role of SLC44A2 in drug sensitivity, the online GSCA (Gene Set Cancer Analysis) [22] database was utilized to analyze the relationship between SLC44A2 expression and drug sensitivity. The results showed that SLC44A2 expression is positively correlated with resistance to 5-Fluorouracil (5-FU) but negatively correlated with resistance to Cetuximab (Fig. S6A), suggesting that SLC44A2 could serve as a biomarker for predicting drug sensitivity to 5-FU and Cetuximab in CRC treatment. Additionally, we used the online TIMER (Tumor IMmune Estimation Resource) [23] database to examine the relationship between SLC44A2 expression and tumor-infiltrating immune cells in CRC. SLC44A2 expression is positively correlated with the infiltration levels of B cells, CD4^+^ T cells, CD8^+^ T cells, Neutrophils, Macrophages, and Dendritic cells within CRC tumors (Fig. S6A), implying that SLC44A2 may enhance the effectiveness of immunotherapy in CRC. Together, these findings suggest that SLC44A2 may play an important role in drug sensitivity in CRC, which still needs further validation.

## Discussion

Although the dependence of cancer cells on mitochondrial metabolism has been recognized in various cancer types [21], the molecular mechanisms leading to this mitochondrial metabolism remodeling remain largely unclear. SLC44A2 is a mitochondrial membrane-localized ubiquitous transmembrane protein that belongs to the family of choline transporter-like proteins. However, the biological functions of SLC44A2 are not yet fully understood [7]. Previous studies have shown that SLC44A2 is involved in regulating choline transport into mitochondria, thereby promoting ATP production and mitochondrial oxygen consumption [11]. Notably, deletion of SLC44A2 impairs adhesion but increases proliferation in cultured mesenchymal lung cells [12], suggesting its potential involvement in cancer cell proliferation and invasion. However, the effects of SLC44A2 on mitochondrial metabolism and malignant progression in human cancers remain largely unexplored. In this study, we observed a marked reduction in SLC44A2 expression in colorectal cancer (CRC) and found that low SLC44A2 expression was significantly correlated with increased tumor size, higher incidence of tumor thrombus, and poorer survival in CRC patients. Pan-cancer analysis revealed that lower SLC44A2 expression was also associated with poorer survival in several other cancers, including clear cell carcinoma of the kidney (KIRC), glioma (GBMLGG), head and neck squamous cell carcinoma (HNSC), and skin cutaneous melanoma (SKCM). Conversely, lower SLC44A2 expression was associated with better survival in pancreatic adenocarcinoma (PAAD), invasive breast cancer (BRCA), and adrenocortical carcinoma (ACC). These findings suggest that SLC44A2 may play distinct roles in different tumors, possibly due to differences in cellular origins, genetic backgrounds, and microenvironments.

The significant downregulation of SLC44A2 and its close association with patient survival suggests that downregulation of SLC44A2 may play a role in aggressive biological behaviors in CRC. However, the influence of SLC44A2 on the aggressive characteristics of human cancers has not previously been studied. In this study, we demonstrate that SLC44A2 inhibited the short- and long-term proliferation of CRC cells, as evidenced by both in vitro MTS cell viability, Edu and colony formation assays, and in vivo xenograft tumor growth assay in nude mice. These results are consistent with previous findings in cultured mesenchymal lung cells, where SLC44A2 deletion led to faster proliferation [12]. Cell proliferation is mainly determined by cell cycle progression and apoptosis. Our results also showed that SLC44A2 caused a significant slowdown in cell cycle progression from G1 to S and an increase in apoptotic cells, suggesting that SLC44A2 may suppress CRC cell proliferation by promoting cell cycle arrest and apoptosis. Meanwhile, Ki-67 and tunnel staining assays in nude mouse xenograft tumors provided further support for SLC44A2 as a regulator of cellular proliferation by promoting cell cycle arrest and facilitating apoptosis. Our data from in vitro transwell migration and invasion assays and in vivo lung metastasis model in nude mice indicated that SLC44A2 also inhibited the migratory and invasive capacities of CRC cells. In contrast to our observations in CRC cells, an earlier study has indicated that deletion of SLC44A2 significantly suppressed cell adhesion in cultured mesenchymal lung cells [12], suggesting that SLC44A2 may play distinct roles in different cell types.

Cancer cells reprogram metabolic pathways to support their energy and biosynthetic demands. Although enhanced aerobic glycolysis, also known as Warburg effect, has been well established as the main metabolic reprogramming of malignant cells [24], abnormal lipid metabolism has been recently recognized as another metabolic hallmark of cancer [21, 25]. Here, we found that SLC44A2 downregulation in CRC cells promoted mitochondrial metabolism by enhancing fatty acid oxidation (FAO), which generates not only ATP for energy supply but also NADPH for the maintenance of redox homeostasis in CRC. This is consistent with previous studies linking FAO to cancer cell survival, proliferation, drug resistance, and metastasis [21]. Carnitine palmitoyltransferase 2 (CPT2), a rate-limiting enzyme in FAO, has been implicated in various diseases, including cancer [26]. However, its expression and function in cancer remain controversial. Downregulations and tumor suppressive functions of CPT2 have been observed in ovarian [27], liver [28] and renal [29] cancers. On the contrary, like our findings in CRC, it was also reported that CPT2 played an oncogenic function in triple-negative breast cancer (TNBC) cells [30, 31], suggesting that CPT2 may play distinct functions in different types of cancer. Despite the aberrant expression of CPT2 in multiple cancer types, the underlying molecular mechanisms, especially from mitochondrial, leading to the aberrant expression of CPT2 remain largely unclear. Our study shows that the mitochondrial membrane-localized choline transporter-like protein SLC44A2 promoted mitochondrial E3 ubiquitin ligase 1 (MUL1)-mediated proteasomal degradation of carnitine palmitoyl transferase 2 (CPT2) by decreasing acetyl-CoA-mediated acetylation of CPT2. This suggests that MUL1 is a specific E3 ubiquitin ligase for CPT2 and their interaction can be disrupted by CPT2 acetylation. Importantly, MUL1 expression remained unchanged regardless of SLC44A2 overexpression or knockdown, indicating that SLC44A2 inhibits mitochondrial FAO by promoting MUL1-mediated degradation of CPT2 through increased interaction between MUL1 and CPT2, rather than by upregulating MUL1 expression. MUL1 is a mitochondrial membrane- located RING E3 ligase that has been linked to several pathologies, such as cardiovascular, neurological diseases and cancer, and thus been proposed as a potential therapeutic target [32]. Similar to CPT2, MUL1 acts as an oncogene and as a tumor suppressor in different cancer types depending on the different targets [20]. However, the role of MUL1 in CRC remains unexplored. Here we show that MUL1-mediated downregulation of CPT2 play a crucial suppressive role in CRC progression, suggesting that MUL1 may function as a potential tumor suppressor in CRC, which still needs more investigations.

Protein acetylation is a common post-translational modification that regulates protein function and stability [33]. As a crucial mitochondrial metabolic intermediate, acetyl-CoA also serves as the acetyl group donor in protein acetylation [13]. Therefore, the protein acetylation modification is in tune with metabolic state. We identified an acetyl-CoA-MUL1-CPT2 positive feedback loop in CRC cells with SLC44A2 downregulation, highlighting the role of acetyl-CoA-mediated protein acetylation in regulating mitochondrial metabolism. Acetylation can stabilize proteins by competing with ubiquitination when both modifications occur on the same lysine residue. For example, p53 stabilization by acetylation prevents MDM2-mediated ubiquitination at the same site [34]. Similarly, we found that decreased acetylation and expression of CPT2 caused by SLC44A2 overexpression were restored by inhibiting SIRT3, a critical deacetylase of CPT2 [18, 19]. This suggests that SLC44A2 downregulates CPT2 expression by reducing its acetylation, likely through competition with MUL1-mediated ubiquitination at the same site in CPT2. However, the specific acetylation and ubiquitination sites in CPT2 were not investigated in this study and warrant further exploration. Moreover, we demonstrate that CPT2-mediated mitochondrial fatty acid oxidation (FAO) plays an essential role in CRC proliferation and invasion promoted by SLC44A2 downregulation. While SLC44A2 suppresses mitochondrial FAO-mediated energy metabolism and ATP production in CRC cells, it has been reported that SLC44A2 promotes mitochondrial oxygen consumption and ATP production in mouse platelets [11]. These contrasting observations suggest that SLC44A2 may exert distinct effects in different cell types or species, highlighting its potential dual roles in health and disease. Furthermore, our findings indicate that SLC44A2 suppresses CRC proliferation and invasion by inhibiting CPT2-mediated mitochondrial FAO.

In summary, our study provides a molecular basis for SLC44A2 as a potential tumor suppressor in CRC by downregulating CPT2-mediated mitochondrial FAO in a MUL1-dependent manner (Graphical abstract, generated using the Microsoft PowerPoint software). These findings support SLC44A2 as a promising therapeutic target for CRC treatment.

## Supplementary information

Supplementary figures and tables

Full uncropped Gels and Blots image

## Data Availability

All data supporting the findings of this study are available from the corresponding author on reasonable request.
