# Supplementary material for: SLC44A2 negatively regulates mitochondrial fatty acid oxidation to suppress colorectal progression by blocking the MUL1-CPT2 interaction
Source: Cell Death Dis. 2025 Jul 1;16(1):468. doi: 10.1038/s41419-025-07781-z (PMC12219296; doi:10.1038/s41419-025-07781-z)
Supplement: Supplementary file 1 — Supplementary figures and tables [file 41419_2025_7781_MOESM1_ESM.docx]

**Supplemental information**

**SLC44A2 negatively regulates mitochondrial fatty acid oxidation to suppress colorectal progression by blocking the MUL1-CPT2 interaction**

**Supplemental Figures**

**Figure S1. A pan-cancer analysis was conducted for the expression and clinical implication of SLC44A2 in CRC.** (A and B) Significant decreased (A) or increased (B) expressions of SLC44A2 were observed in different cancer types. (C and D) Favorable (C) and unfavorable (D) prognosis of SLC44A2 expression were revealed in different cancer types.


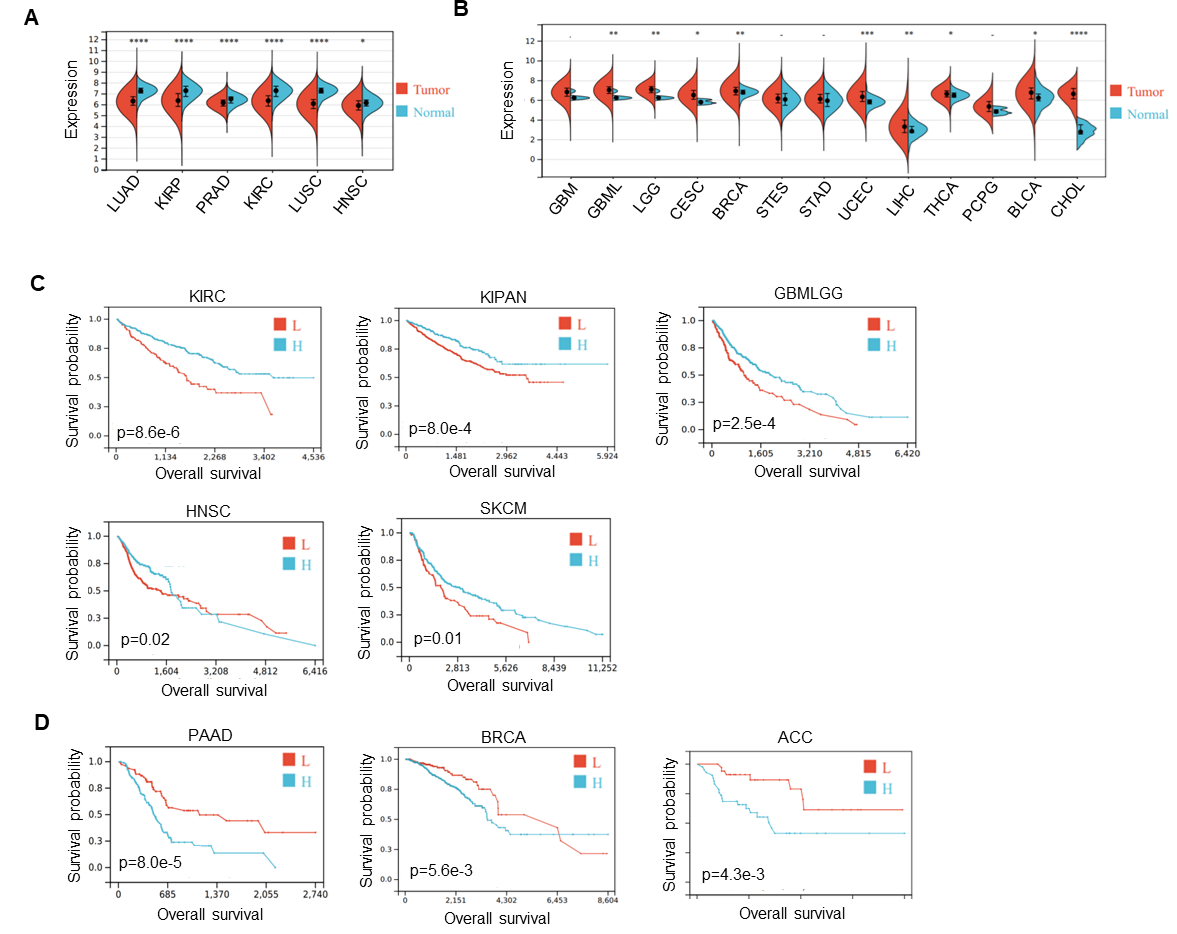


**Figure S2. Forced expression of SLC44A2 impaired the proliferation and invasion of CRC cells both *in vitro* and *in vivo*. (A and B)** The efficiencies of forced expression of SLC44A2 were verified using qRT-PCR (A) and Western blot (B) analysis in HCT116 and LS174T cells. **(C and D)** Cell cycle (C) and apoptosis (D) were examined by flow cytometry analysis in HCT116 and LS174T cells. **(E and F)** IHC staining of SLC44A2 (E) and Ki-67 (F) in tumors from SLC44A2 and EV groups. Scale bars, 10 μm. **(G)** TUNEL staining in tumors of SLC44A2 and EV groups. Scale bars, 5μm.


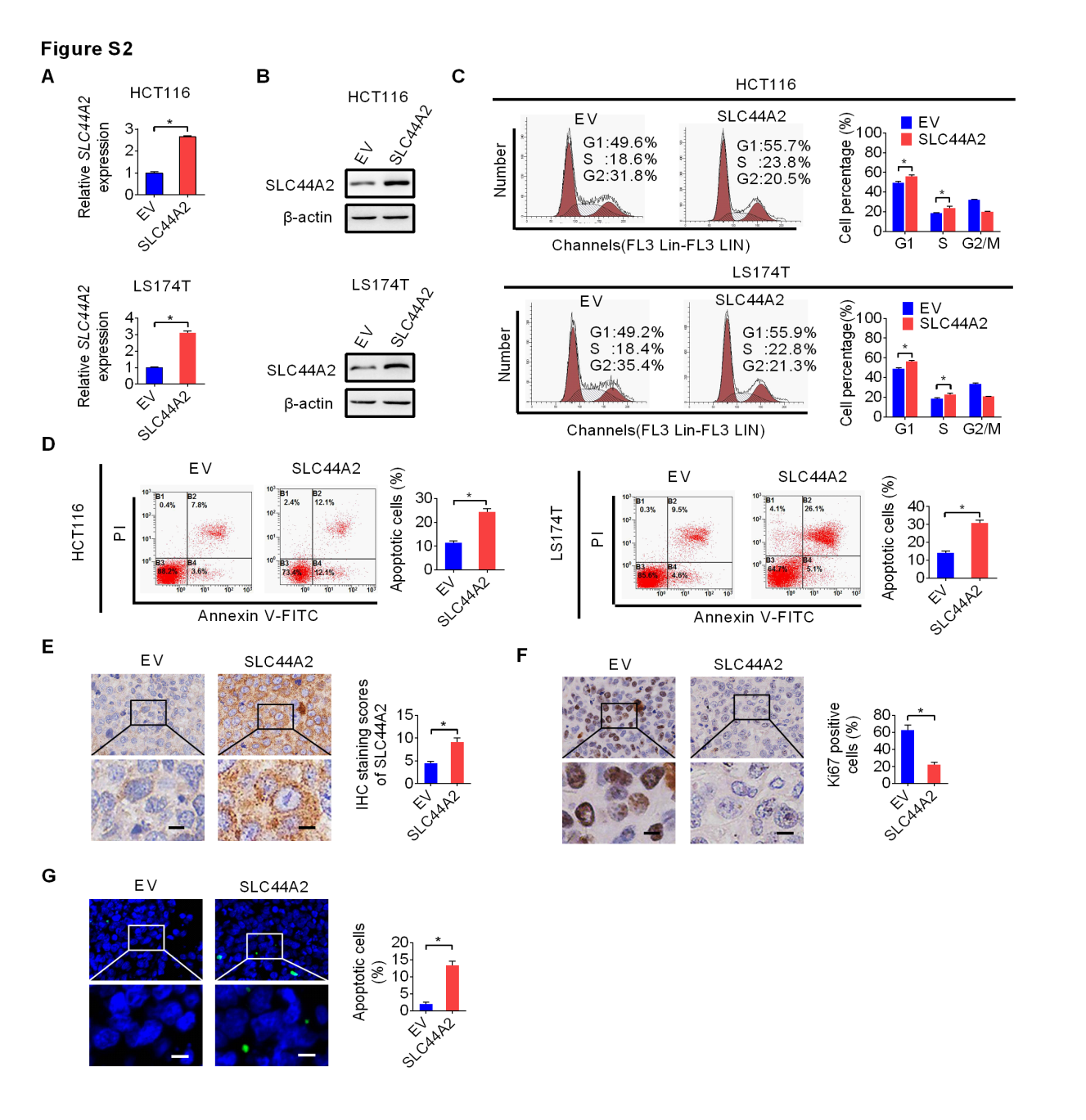


**Figure S3. Knockdown of SLC44A2 enhanced CRC cell proliferation and invasion. (A and B)** Knockdown efficiencies of SLC44A2 were verified using qRT-PCR (A) and Western blot (B) analysis in HT29 and SW480 cells. **(C-E)** Short- and long-term cell proliferations were examined by MTS (C), EdU (D, Scale bars=50 μm) and colony formation (E) assays in HT29 and SW480 cells. **(F and G)** Migration (F) and invasion (G) of HT29 and SW480 cells were examined by transwell migration and invasion assays.

**
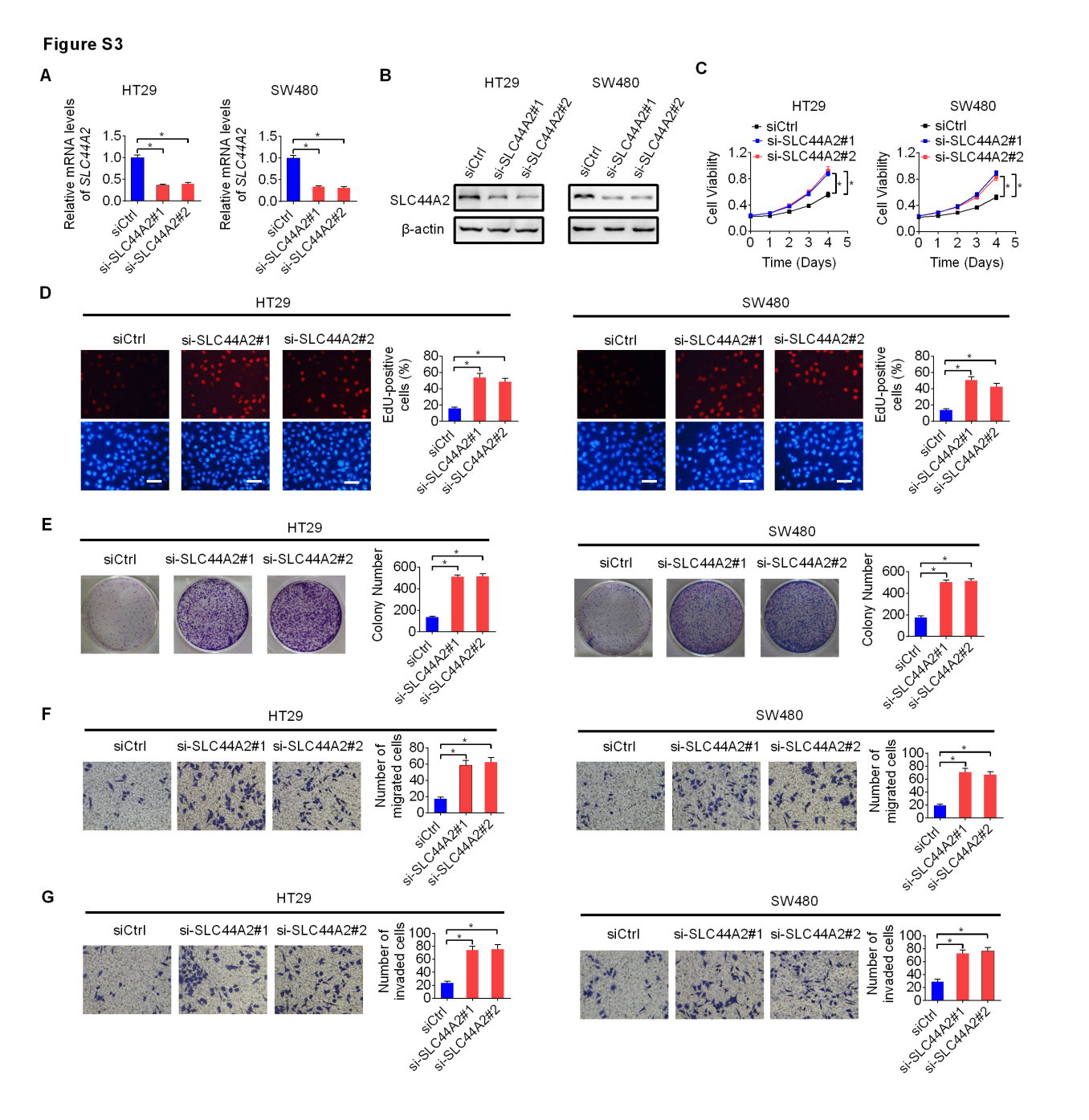
**

**Figure S4.** The protein expressions of MUL1 were compared in CRC and corresponding normal tissues using the online UALCAN database.


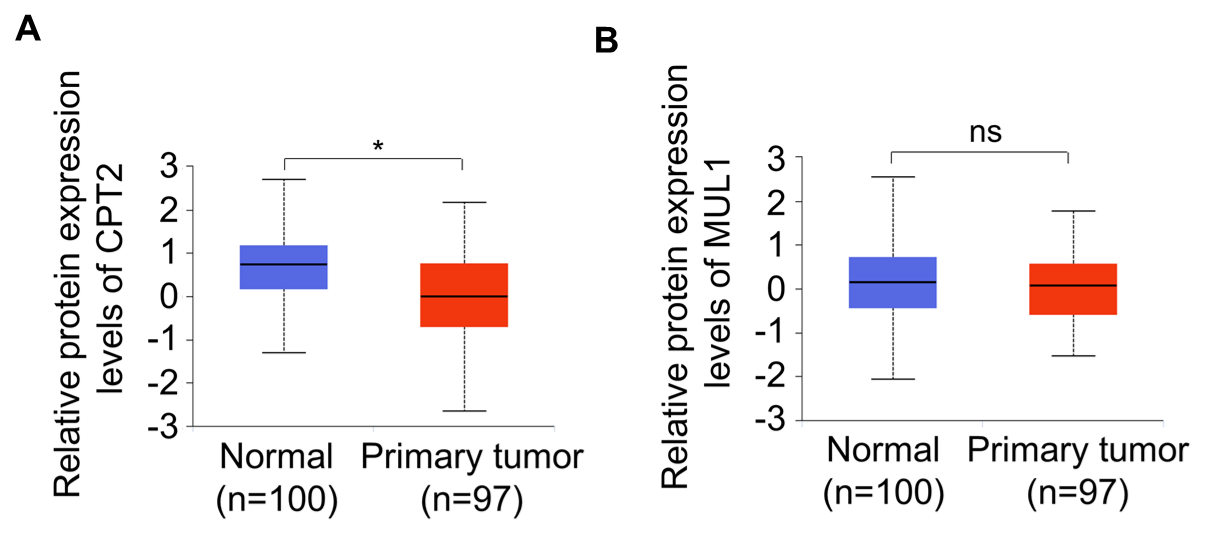


**Figure S5. SLC44A2 suppresses CRC proliferation and invasion by inhibiting FAO. (A-C)** Short- and long-term cell proliferations were examined by MTS (A), EdU (B) and colony formation (C) assays in CRC cells with indicated treatment. **(D and E)** Cell migration (D) and invasion (E) capabilities were examined using transwell assays in CRC cells with indicated treatment.

**
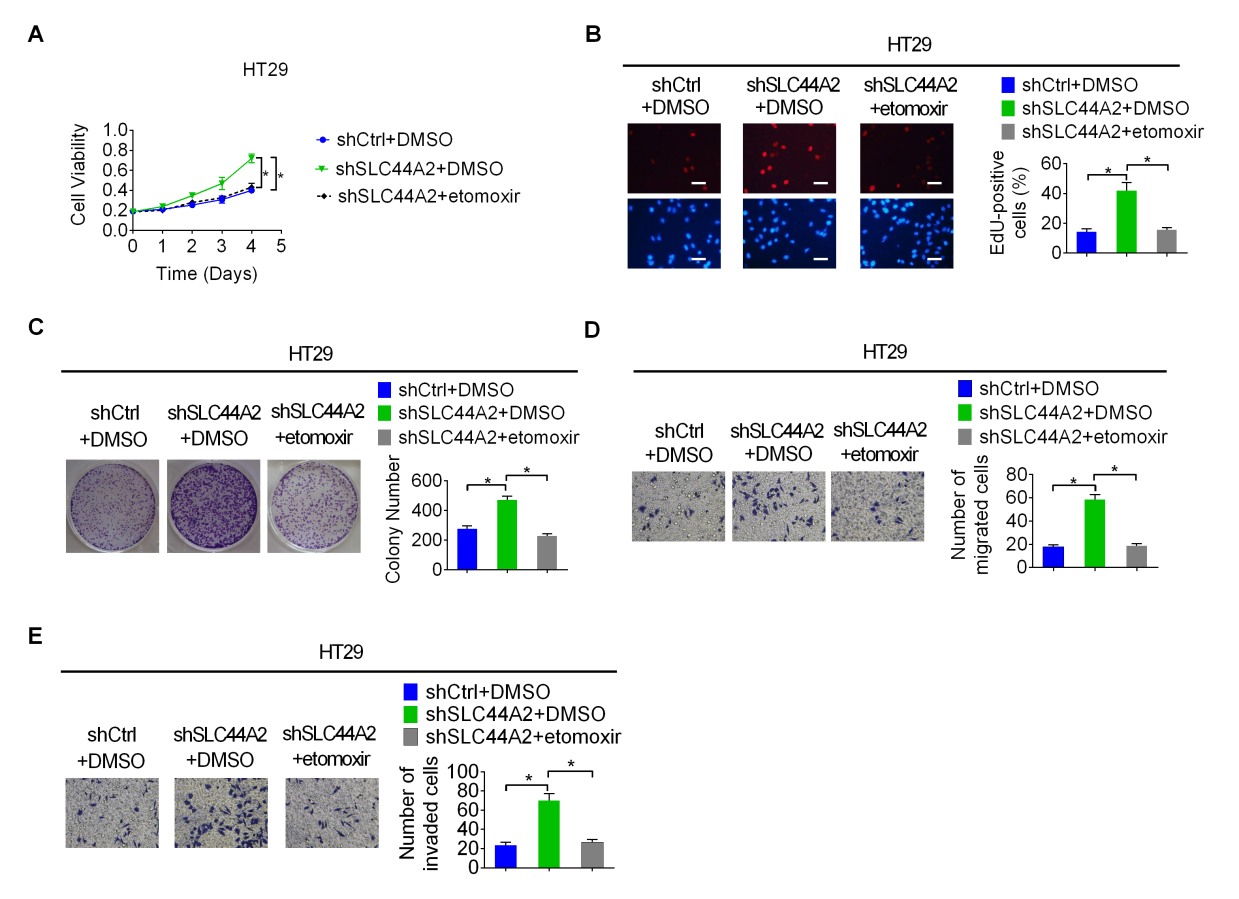
**

**Figure S6. SLC44A2 may play a significant role in drug sensitivity in CRC. (A)** The online GSCA (Gene Set Cancer Analysis) database was utilized to analyze the relationship between SLC44A2 expression and drug sensitivity. (B) The online TIMER (Tumor IMmune Estimation Resource) database was employed to examine the relationship between SLC44A2 expression and tumor-infiltrating immune cells in CRC.


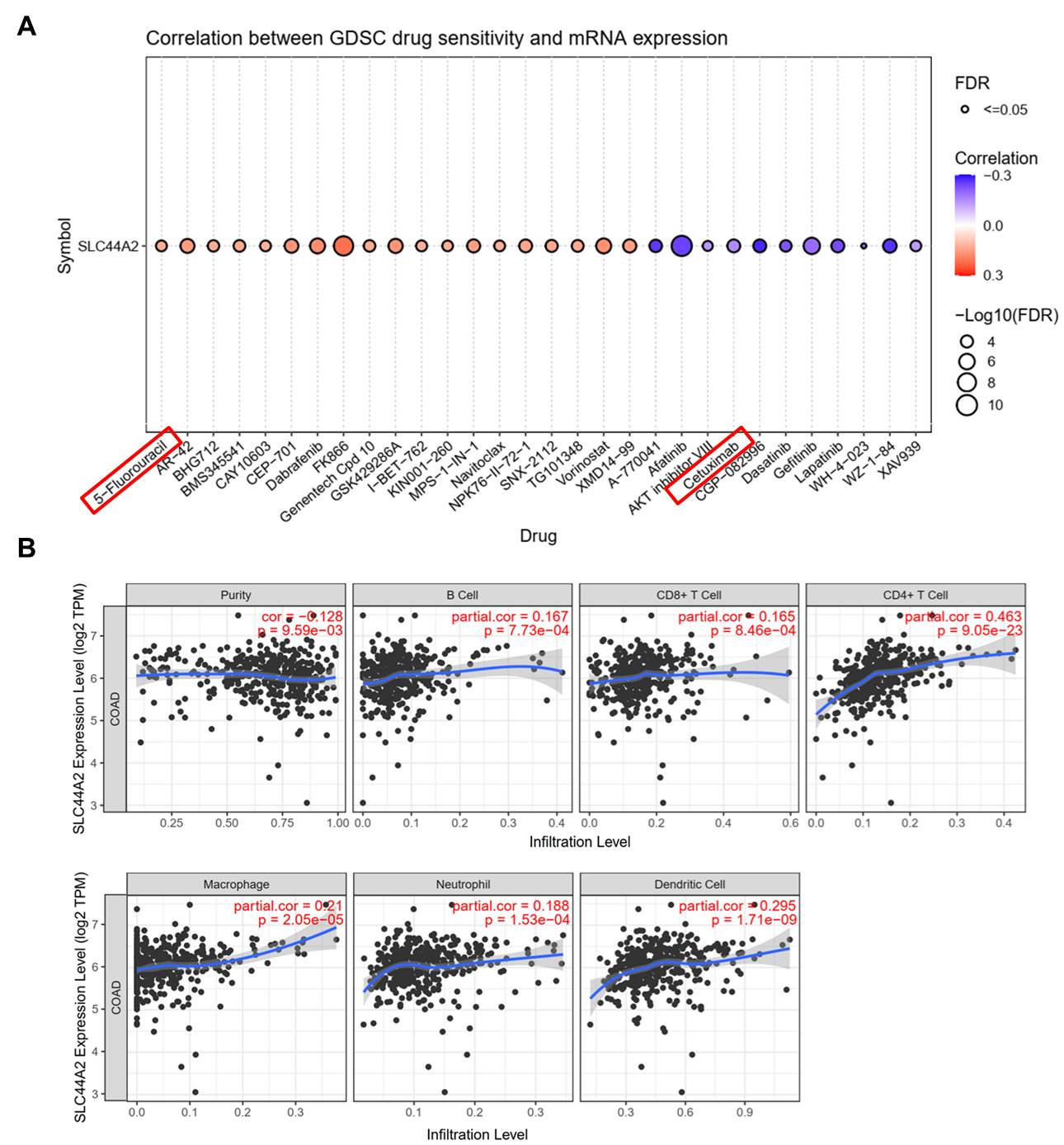


**Supplemental tables**

**Table S1.** Sequence of primers for qRT-PCR analysis

| **1. Primers used in q-PCR analysis** | | |
| --- | --- | --- |
| *SLC44A2* | forward primer | AGGCATCATAGCCTGGACTCA |
|  | reverse primer | TGGGGAGTGGGACATTGGAA |
| *CPT2* | forward primer | CATACAAGCTACATTTCGGGACC |
|  | reverse primer | AGCCCGGAGTGTCTTCAGAA |
| *ND-1* | forward primer | CCCTAAAACCCGCCACATCT |
|  | reverse primer | GAGCGATGGTGAGAGCTAAGGT |
| *HGB* | forward primer | GTGCACCTGACTCCTGAGGAGA |
|  | reverse primer | CCTTGATACCAACCTGCCCAG |
| *β-actin* | forward primer | TCGCCTTTGCGATCCG |
|  | reverse primer | ATGATCTGGGTCATCTTCTCG |

**Table S2. Correlation between SLC44A2 expression and clinicopathologic features in 268 CRC patients.**

| Variables | No. of cases (%) | SLC44A4 expression | | *P* value |
| --- | --- | --- | --- | --- |
|  |  | Low | High |  |
| All | 268 (100%) | 134 | 134 |  |
| Age |  |  |  |  |
| <60 | 147 (54.9%) | 78 | 69 | 0.326 |
| >=60 | 121 (45.1%) | 56 | 65 |  |
| Gender |  |  |  |  |
| Female | 155 (57.8%) | 79 | 76 | 0.805 |
| Male | 113 (42.2%) | 55 | 58 |  |
| Size |  |  |  |  |
| <5 cm | 142 (53.0%) | 80 | 62 | 0.037 |
| >=5 cm | 126 (47.0%) | 54 | 72 |  |
| Lymph node metastasis |  |  |  |  |
| No | 138 (51.5%) | 80 | 58 | 0.010 |
| Yes | 130 (48.5%) | 54 | 76 |  |
| Distant metastasis |  |  |  |  |
| No | 229 (85.4%) | 119 | 110 | 0.165 |
| Yes | 39 (14.6%) | 15 | 24 |  |
| Clinical stage |  |  |  |  |
| I+ II | 92 (34.3%) | 44 | 48 | 0.700 |
| III+ IV | 176 (65.7%) | 90 | 86 |  |
| Differentiation |  |  |  |  |
| Well+Moderate | 112 (41.8%) | 64 | 48 | 0.063 |
| Poor and others | 156 (58.2%) | 70 | 86 |  |
| CEA |  |  |  |  |
| <5 ng/ml | 175 (65.3%) | 94 | 81 | 0.123 |
| >=5 ng/ml | 93 (34.7%) | 40 | 53 |  |
