# Supplementary material for: SLC44A2 negatively regulates mitochondrial fatty acid oxidation to suppress colorectal progression by blocking the MUL1-CPT2 interaction
Source: Cell Death Dis. 2025 Jul 1;16(1):468. doi: 10.1038/s41419-025-07781-z (PMC12219296; doi:10.1038/s41419-025-07781-z)
Supplement: Supplementary file 2 — Full uncropped Gels and Blots image [file 41419_2025_7781_MOESM2_ESM.pdf]

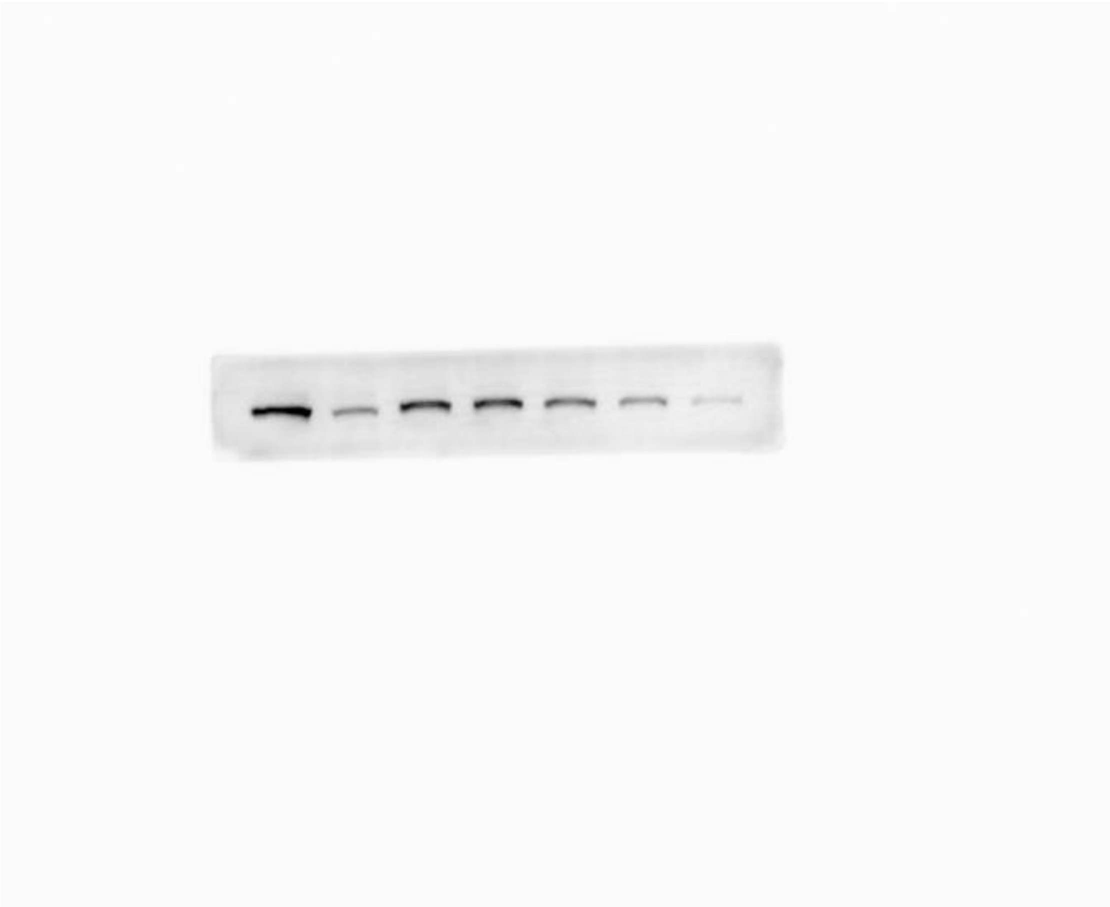

Fig 1E

IB: SLC44A2

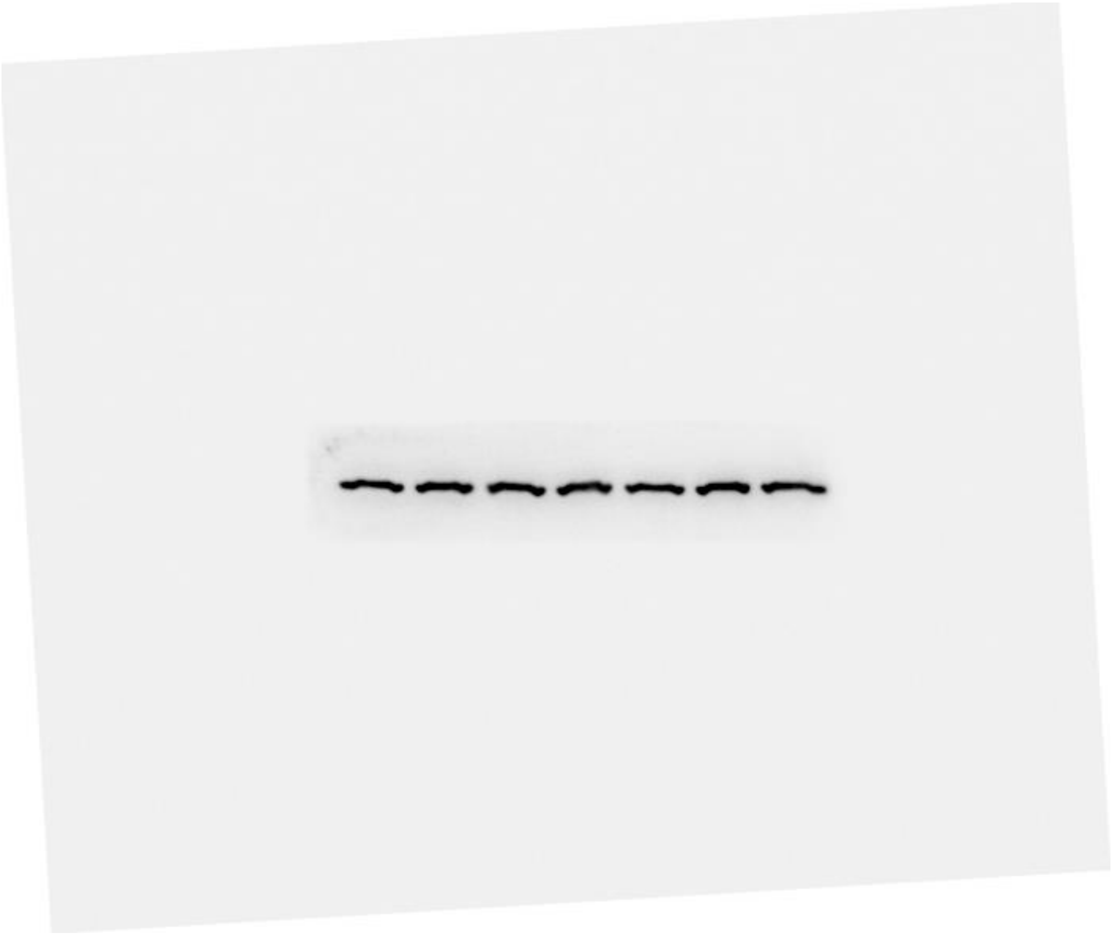

Fig 1E

IB: β-actin

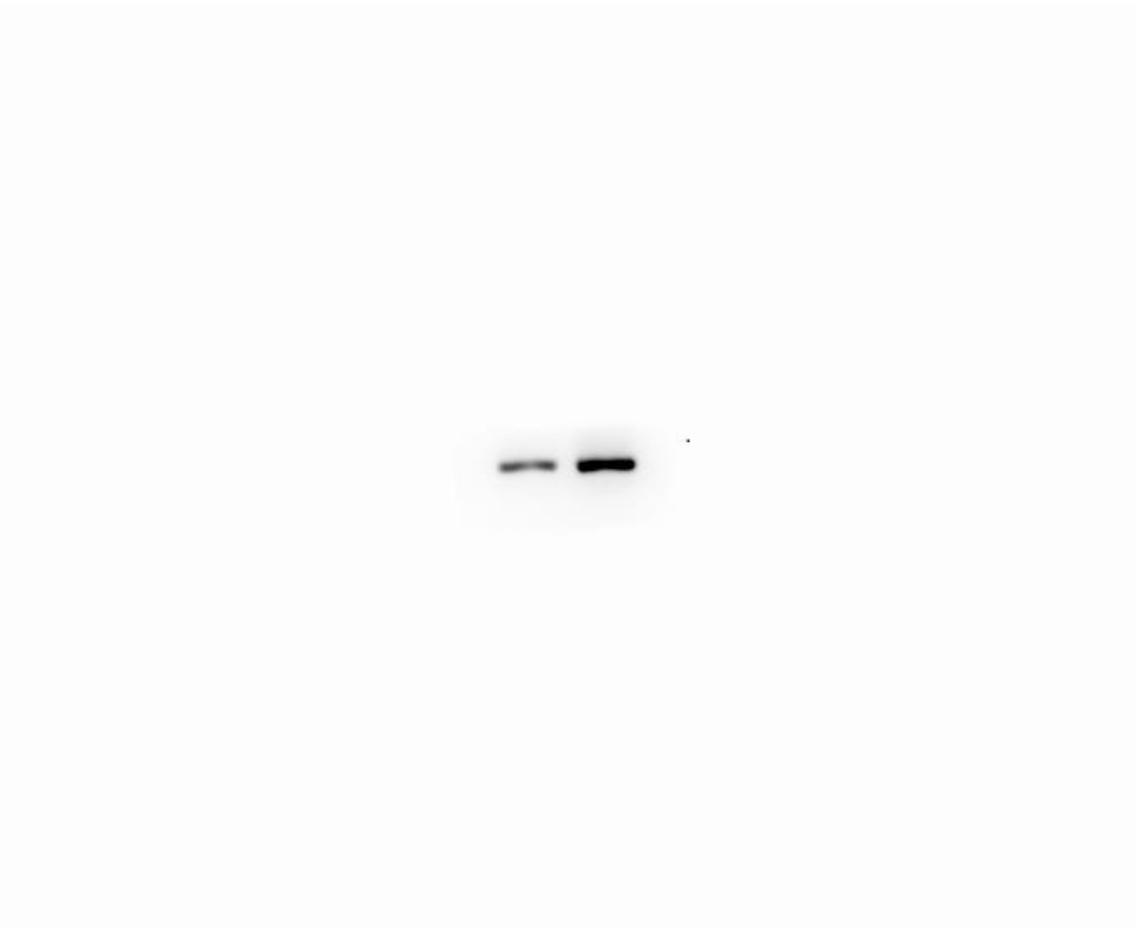

Fig S2B (HCT116 cells)

IB: SLC44A2

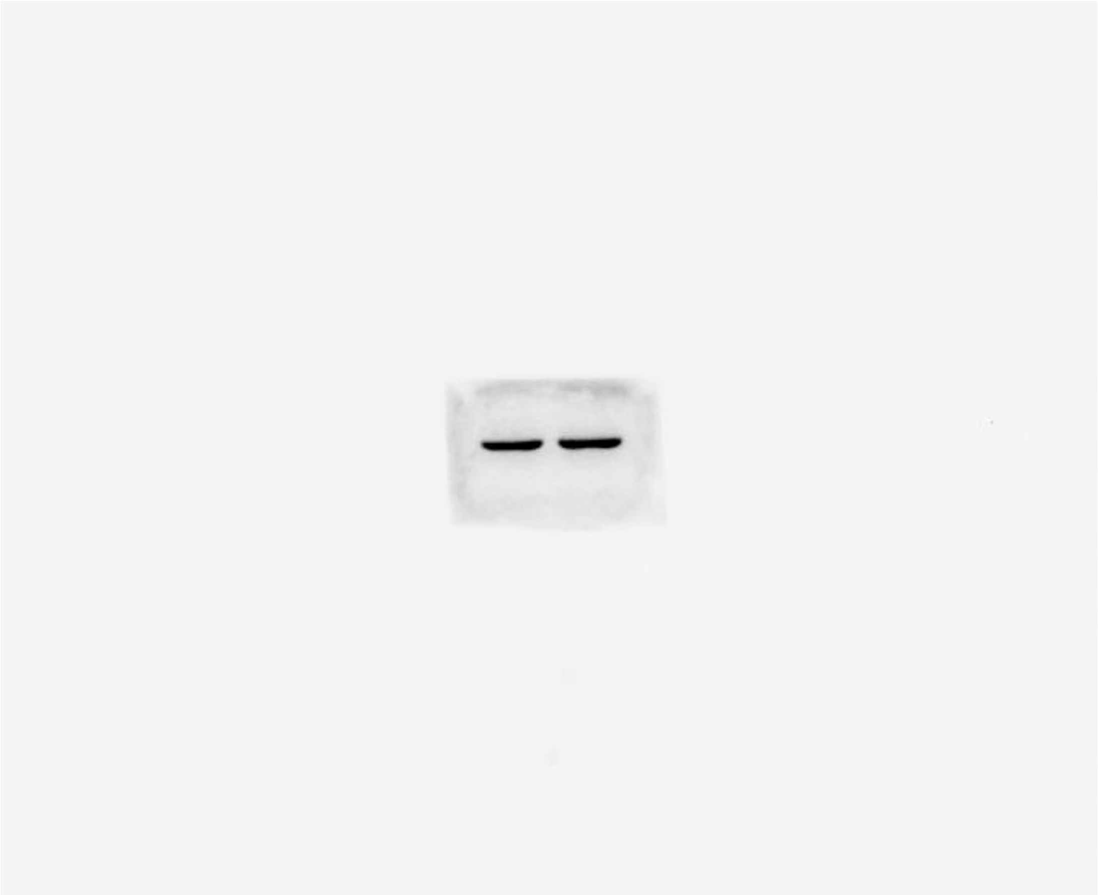

Fig S2B (HCT116 cells)

IB: β-actin

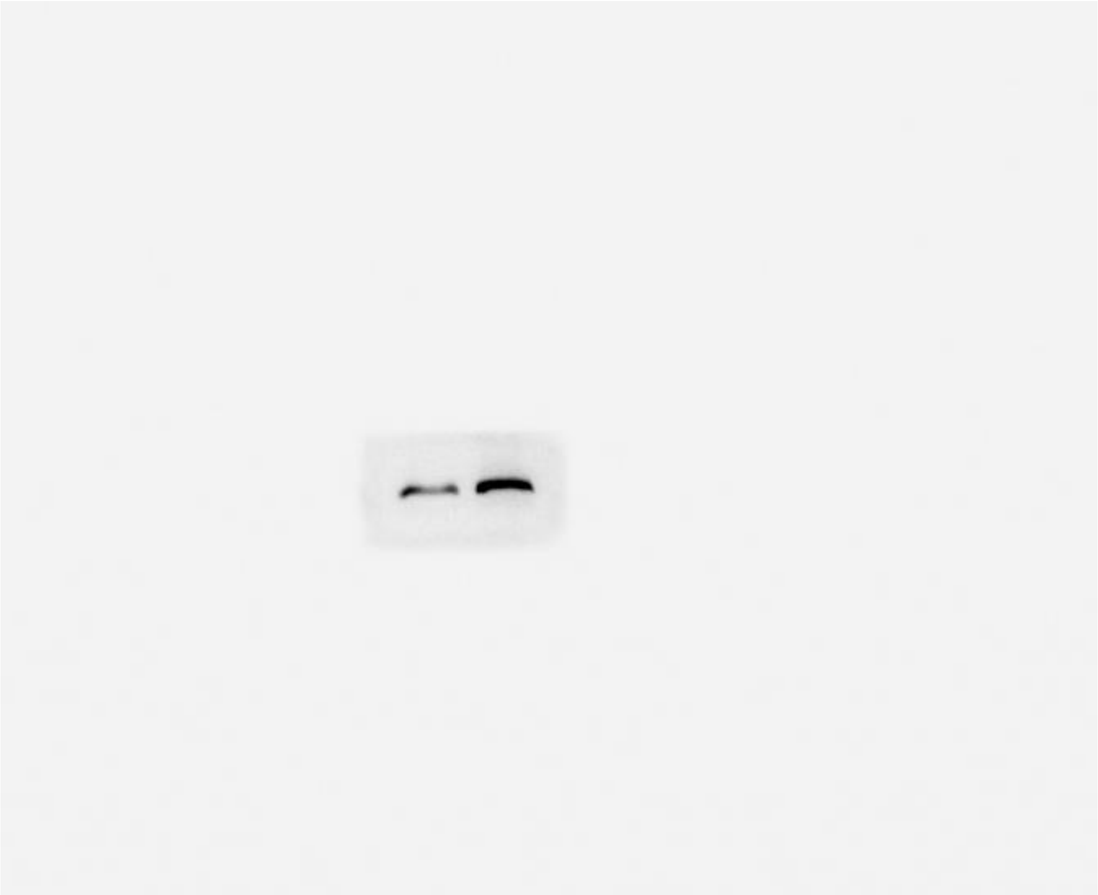

Fig S2B (LS174T cells)

IB: SLC44A2

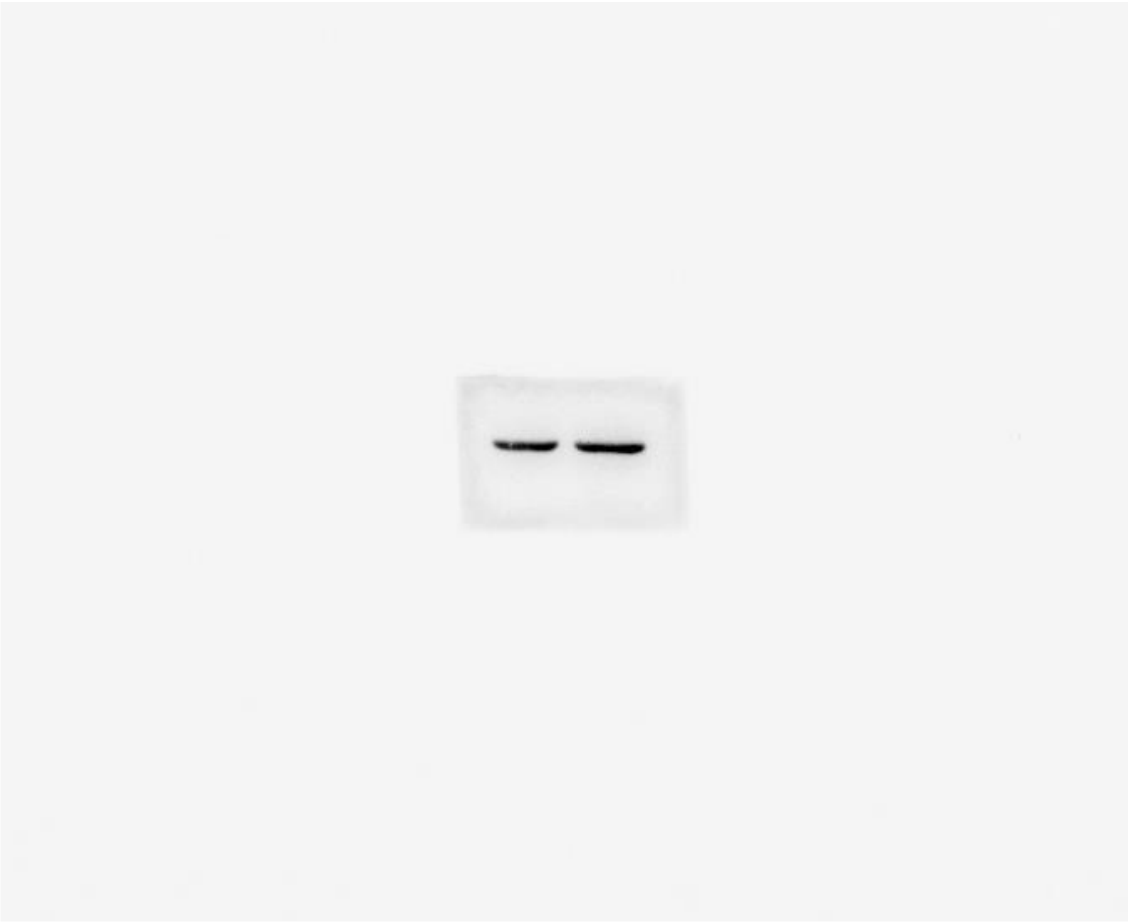

Fig S2B (LS174T cells)

IB: β-actin

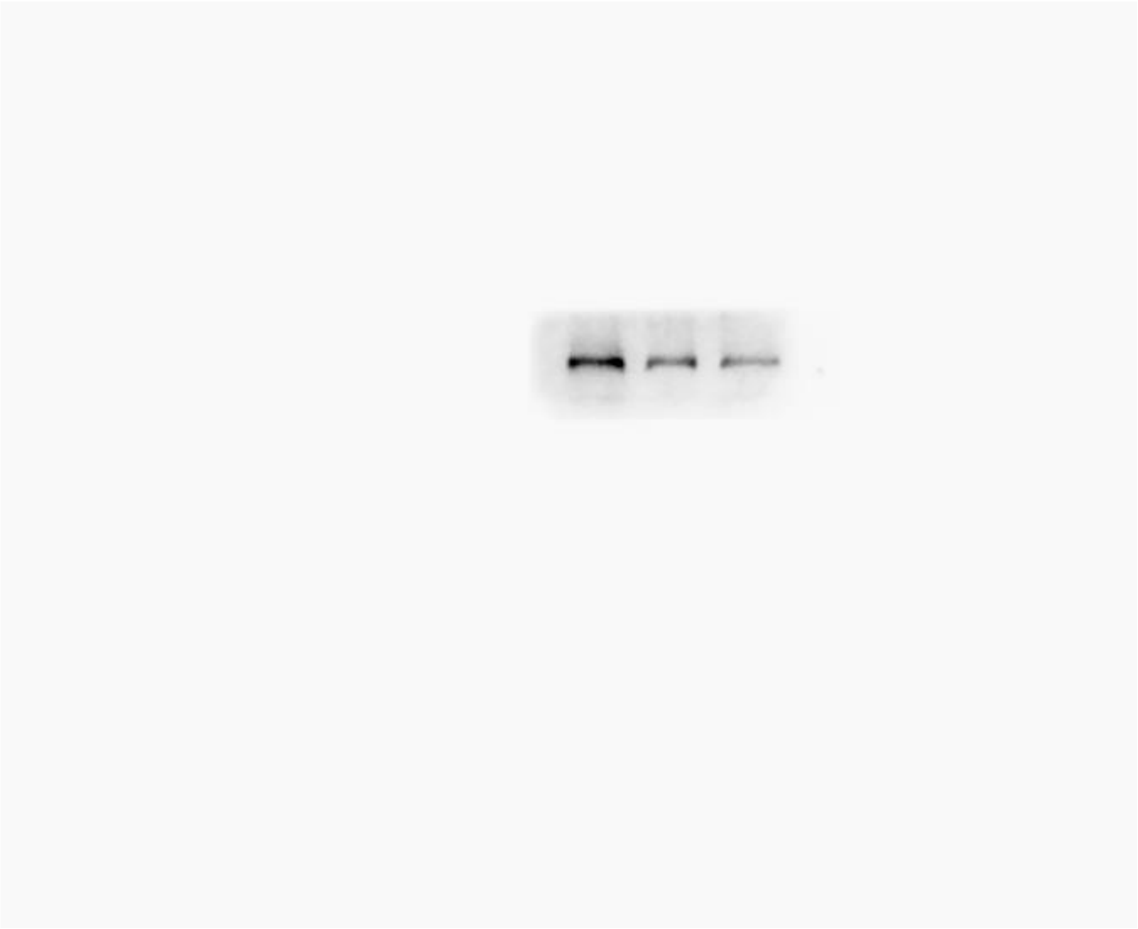

Fig S3B (HT29 cells)

IB: SLC44A2

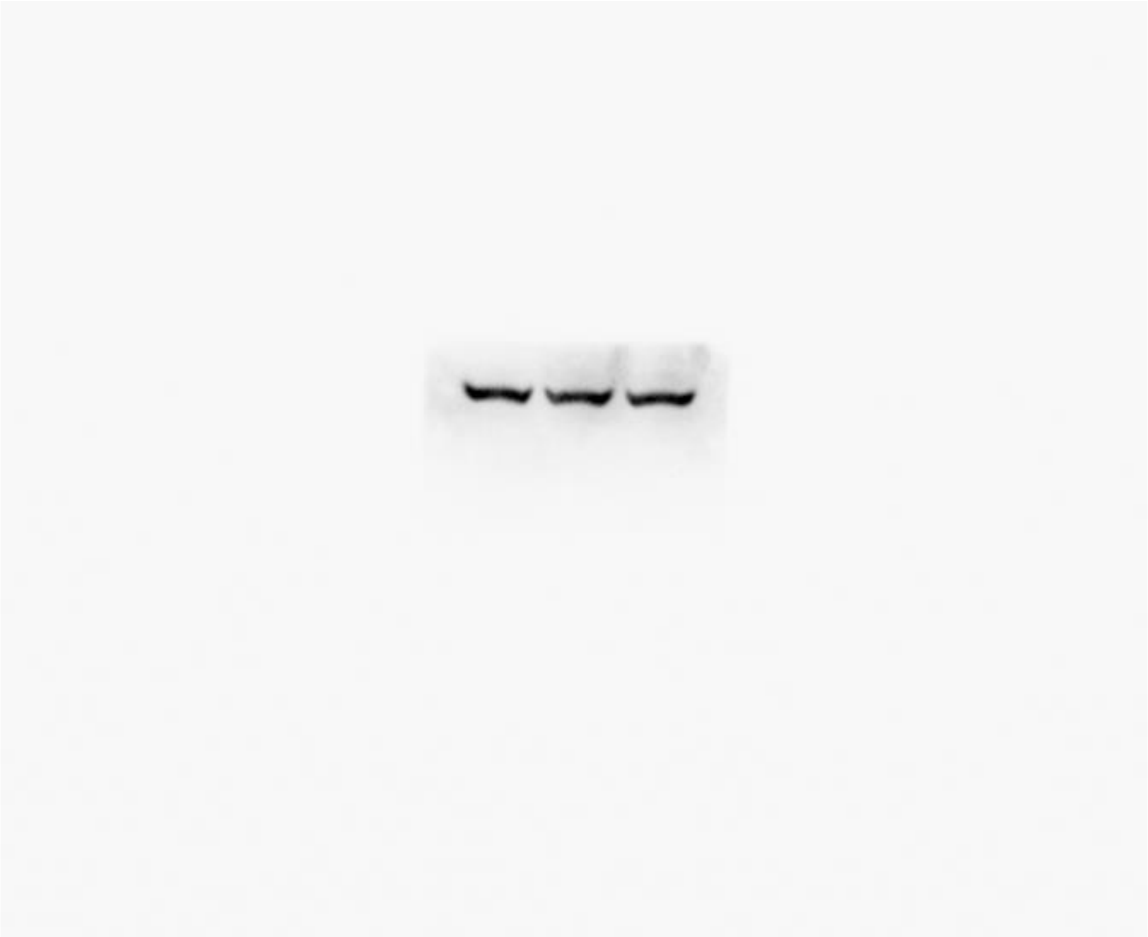

Fig S3B (HT29 cells)

IB: β-actin

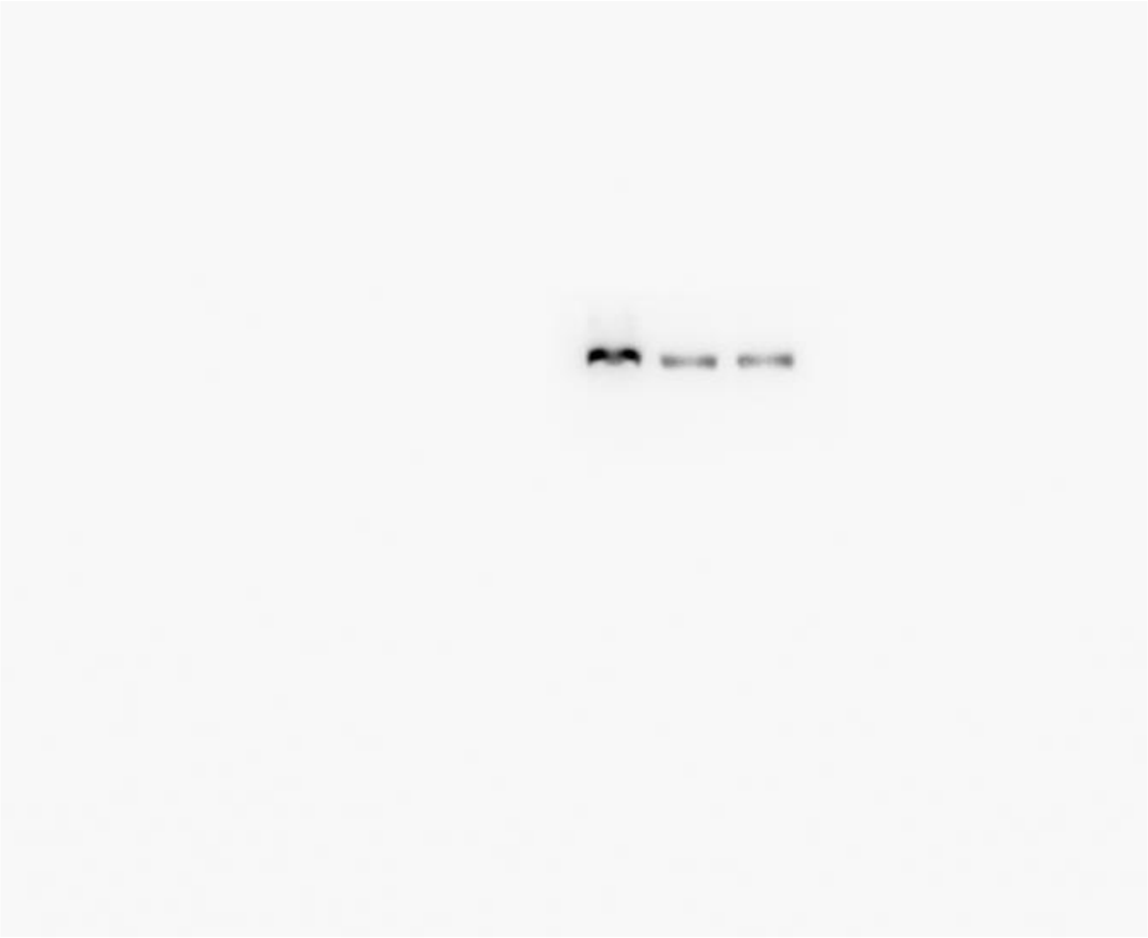

Fig S3B (SW480 cells)

IB: SLC44A2

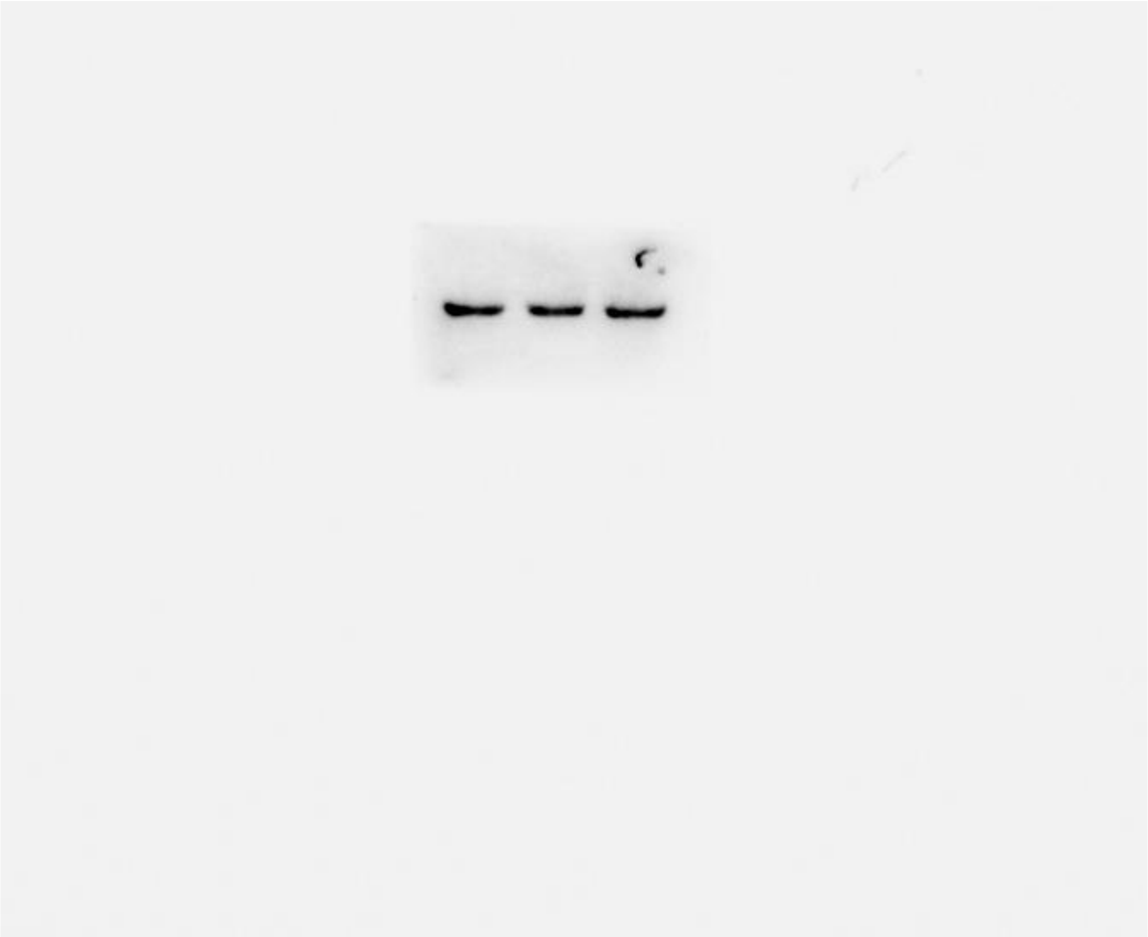

Fig S3B (SW480 cells)

IB: β-actin

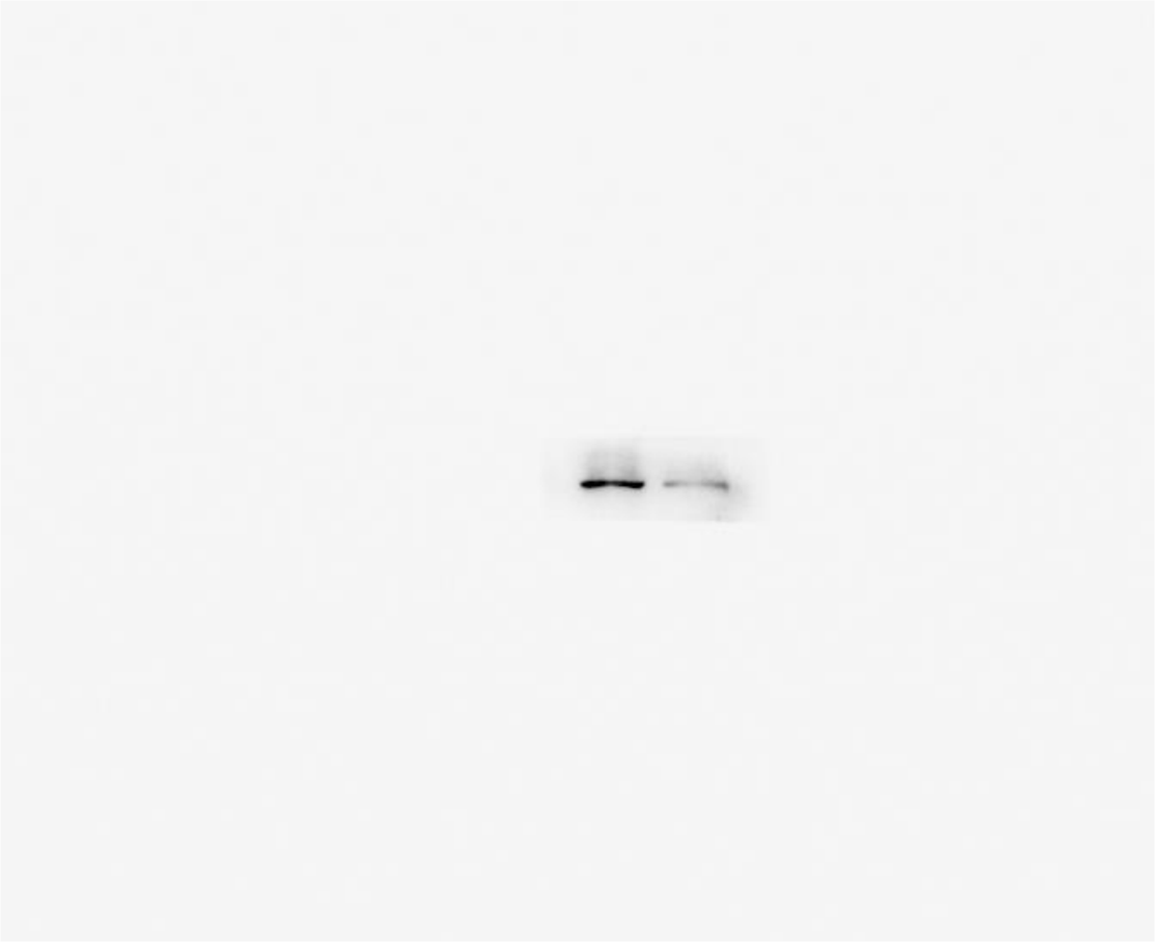

Fig 5C (HCT116 cells)

IB: CPT2

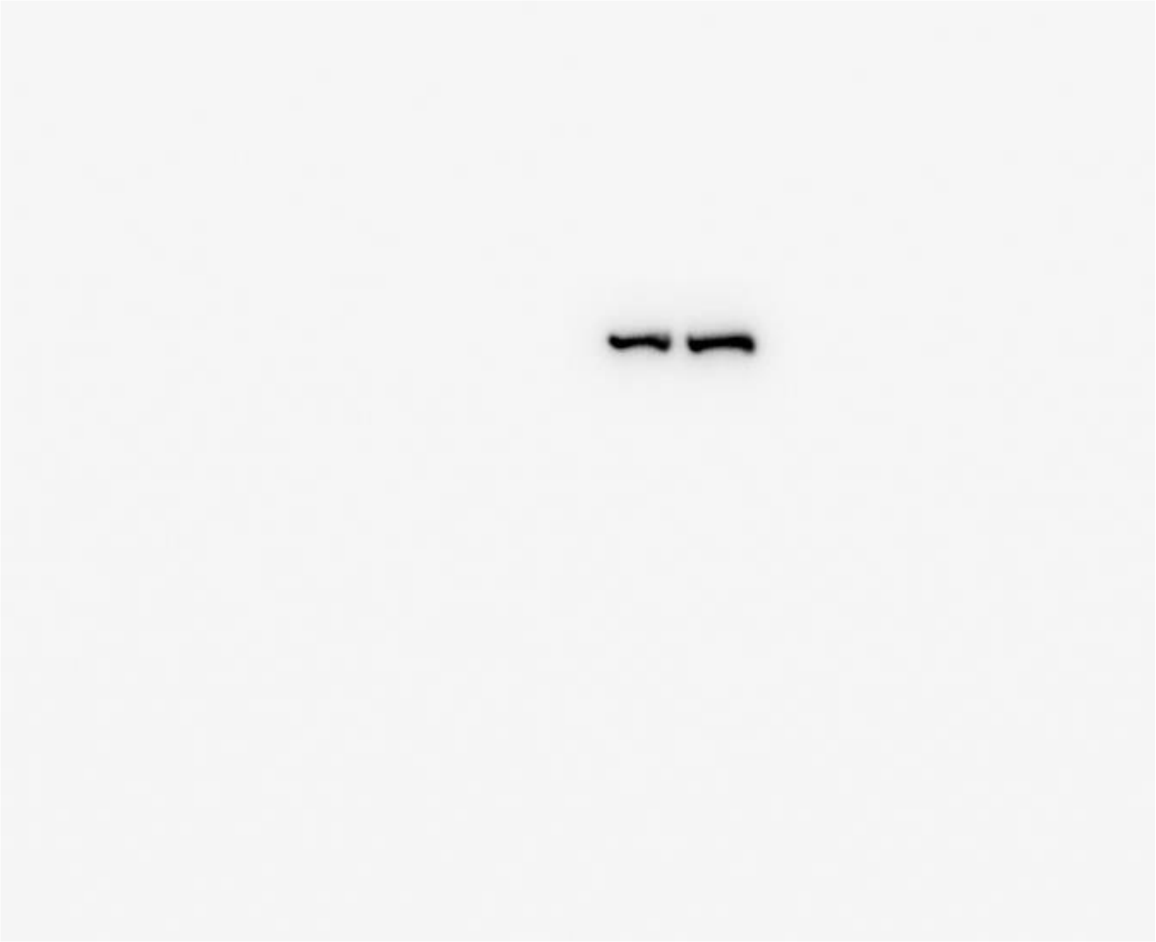

Fig 5C (HCT116 cells)

IB: β-actin

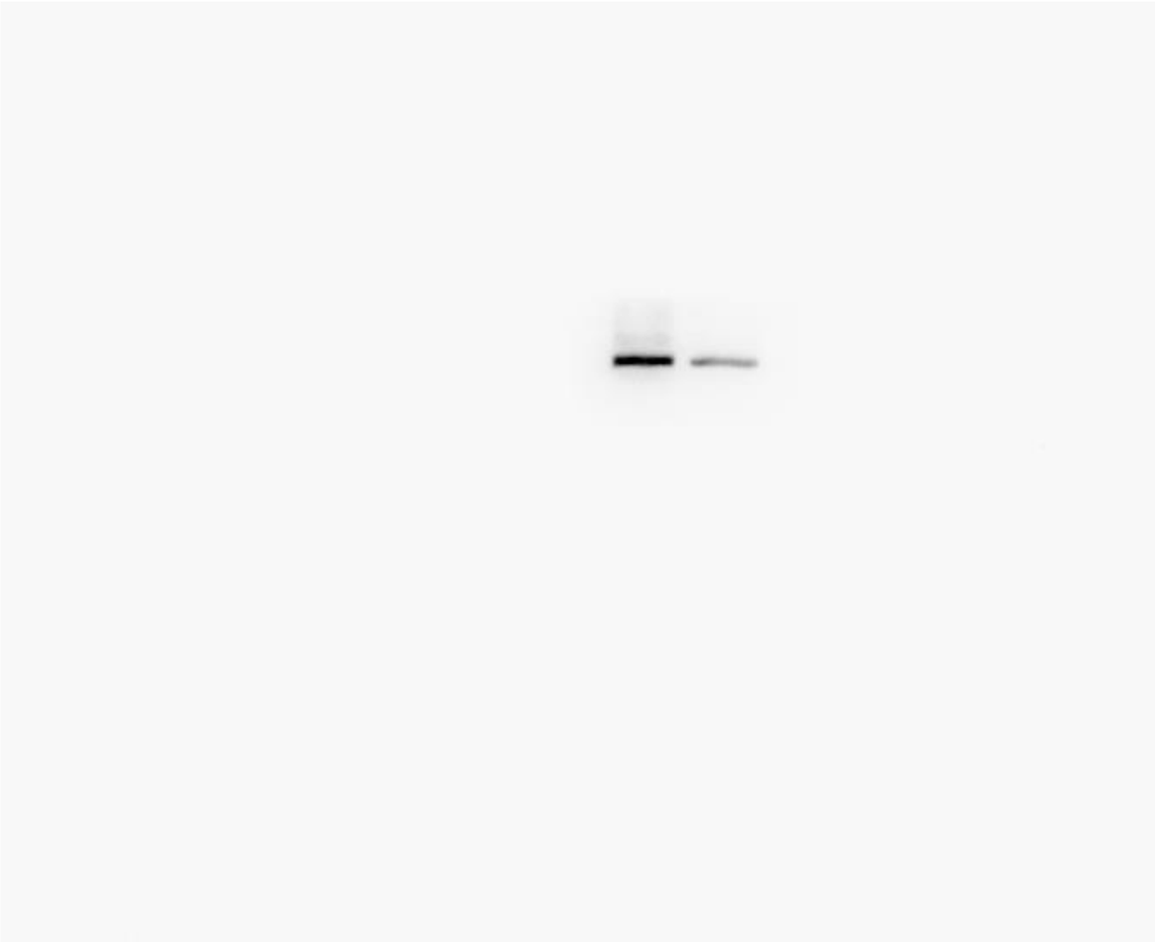

Fig 5C (LS174T cells)

IB: CPT2

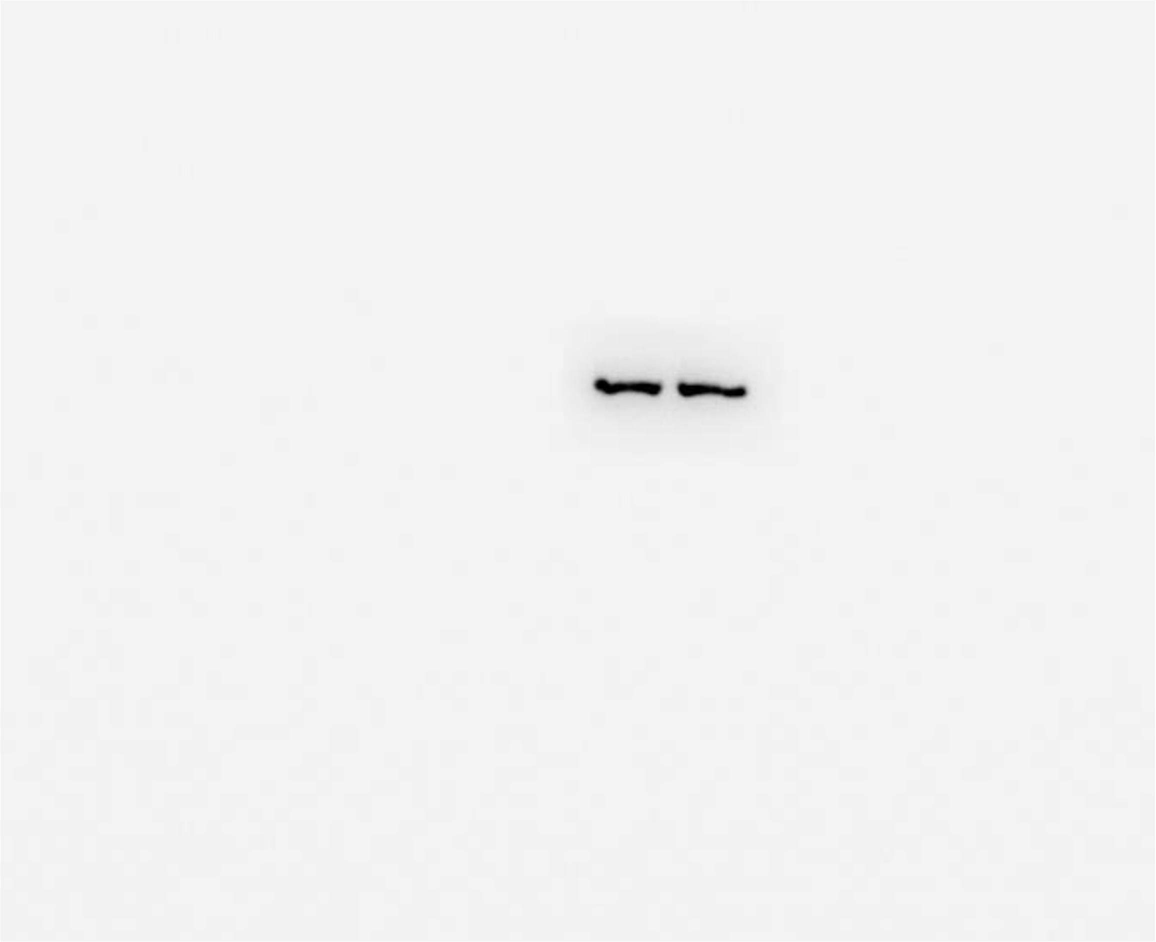

Fig 5C (LS174T cells)

IB: β-actin

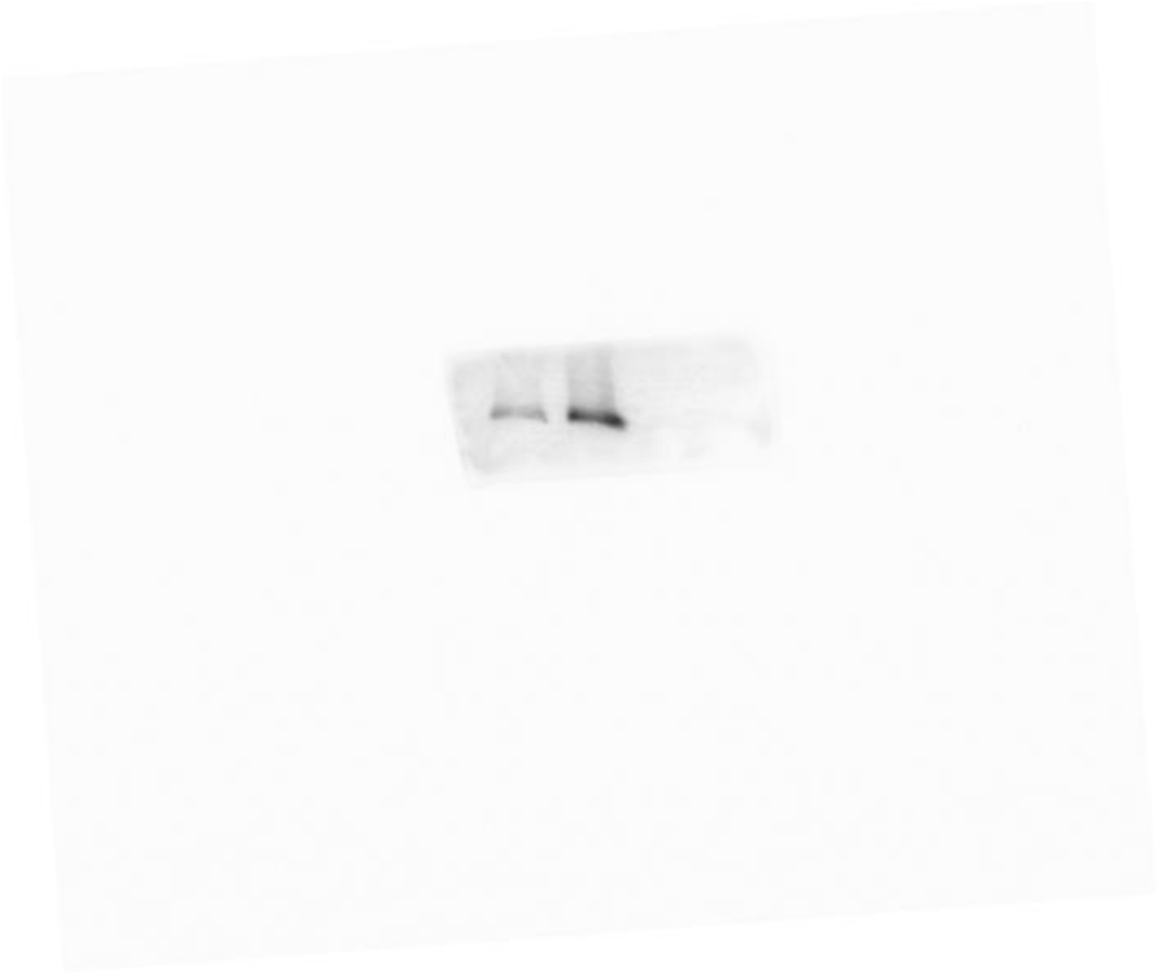

Fig 5C (HT29 cells)

IB: CPT2

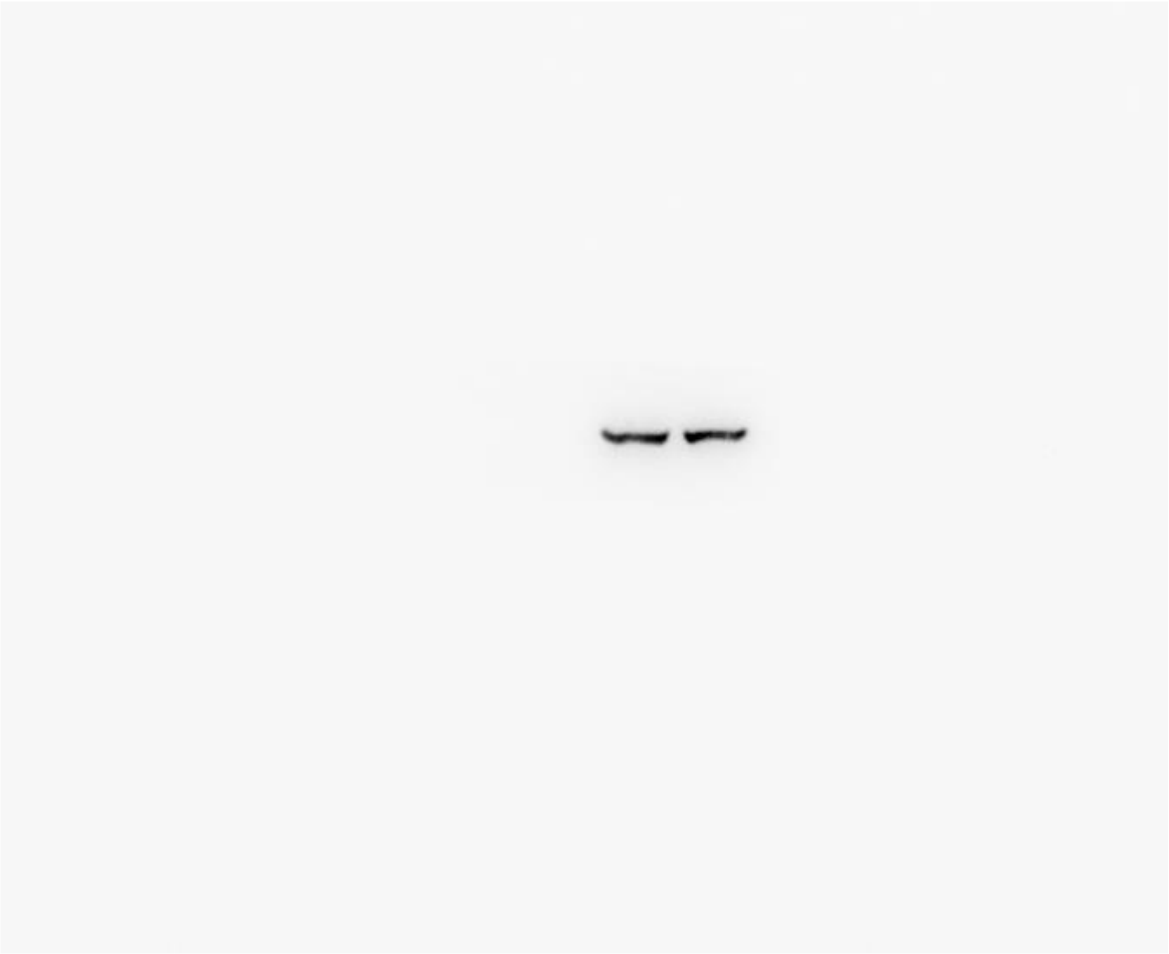

Fig 5C (HT29 cells)

IB: β-actin

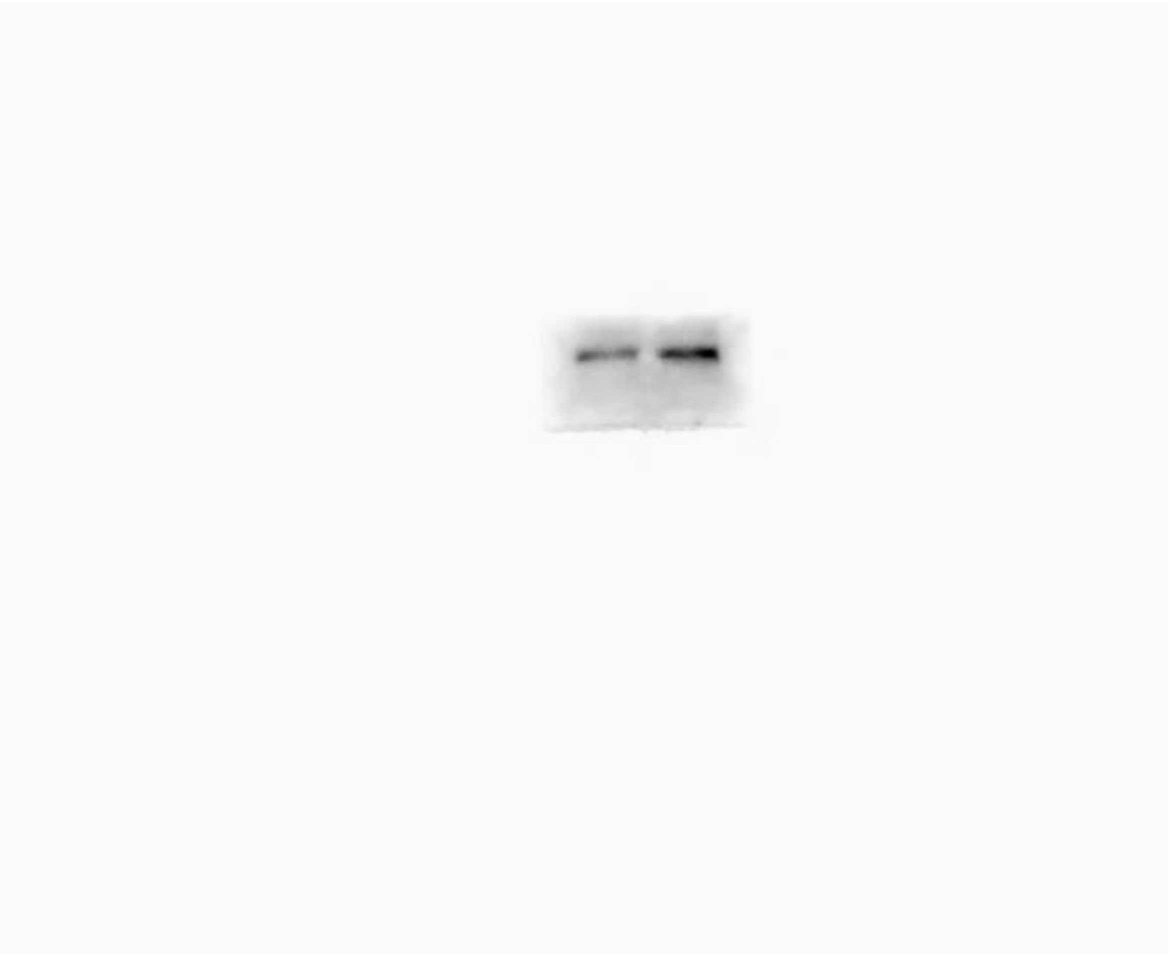

Fig 5C (SW480 cells)

IB: CPT2

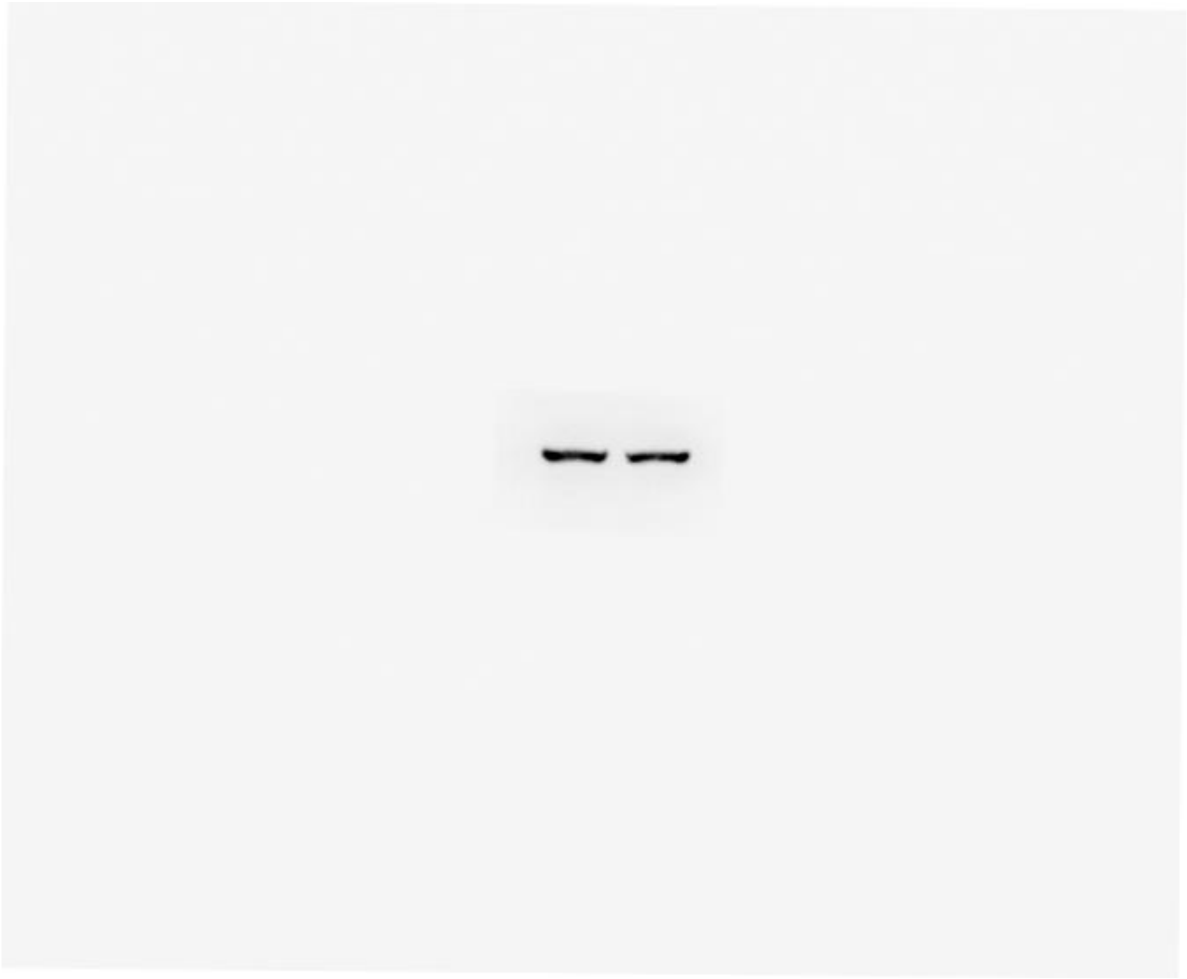

Fig 5C (SW480 cells)

IB: β-actin

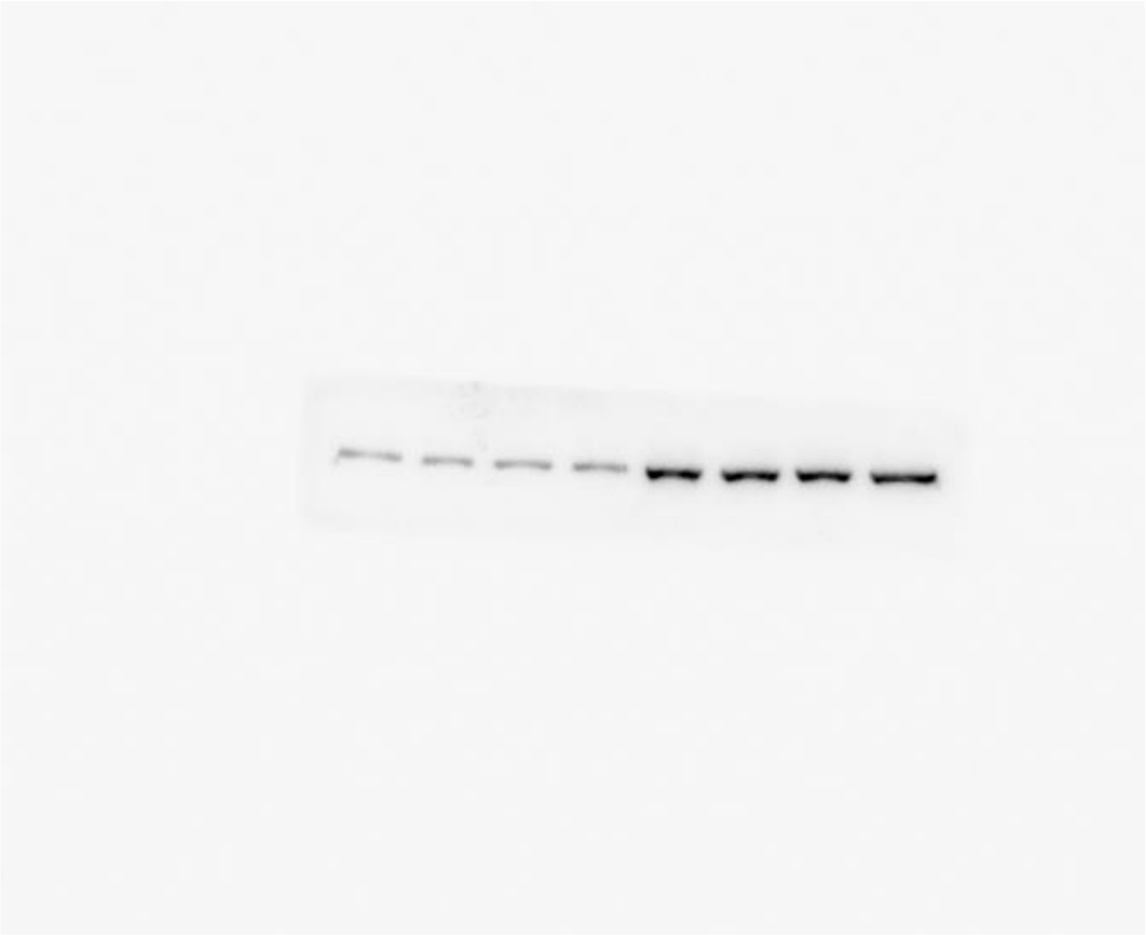

Fig 5D (HCT116 cells)

IB: SLC44A2

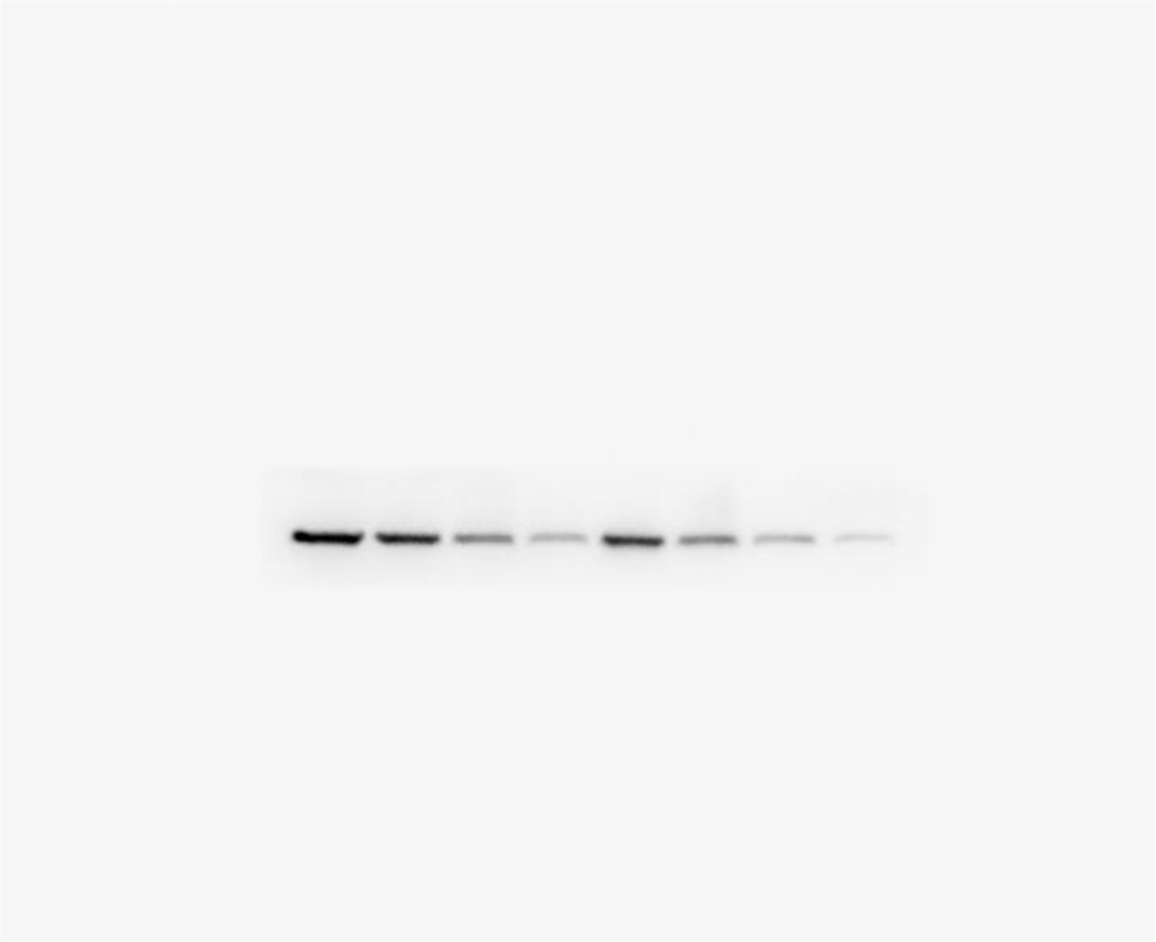

Fig 5D (HCT116 cells)

IB: CPT2

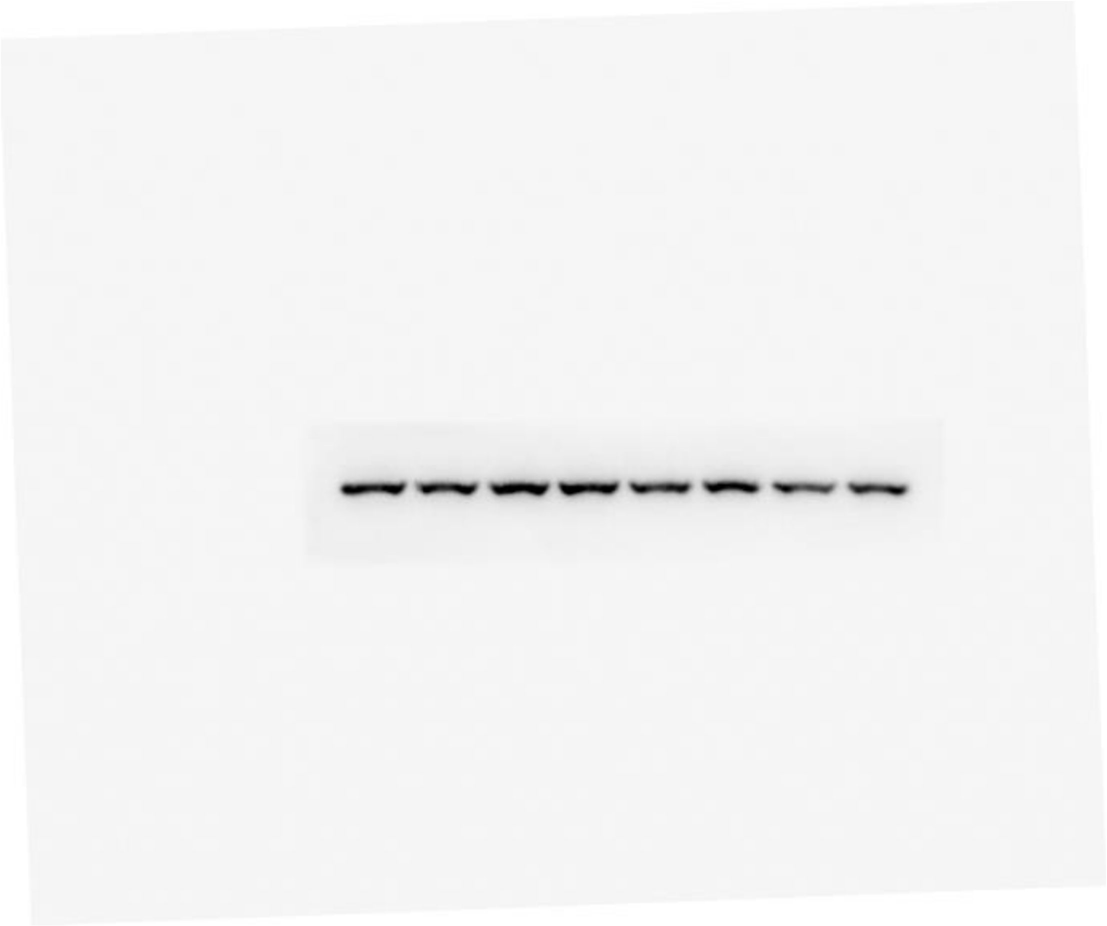

Fig 5D (HCT116 cells)

IB: β-actin

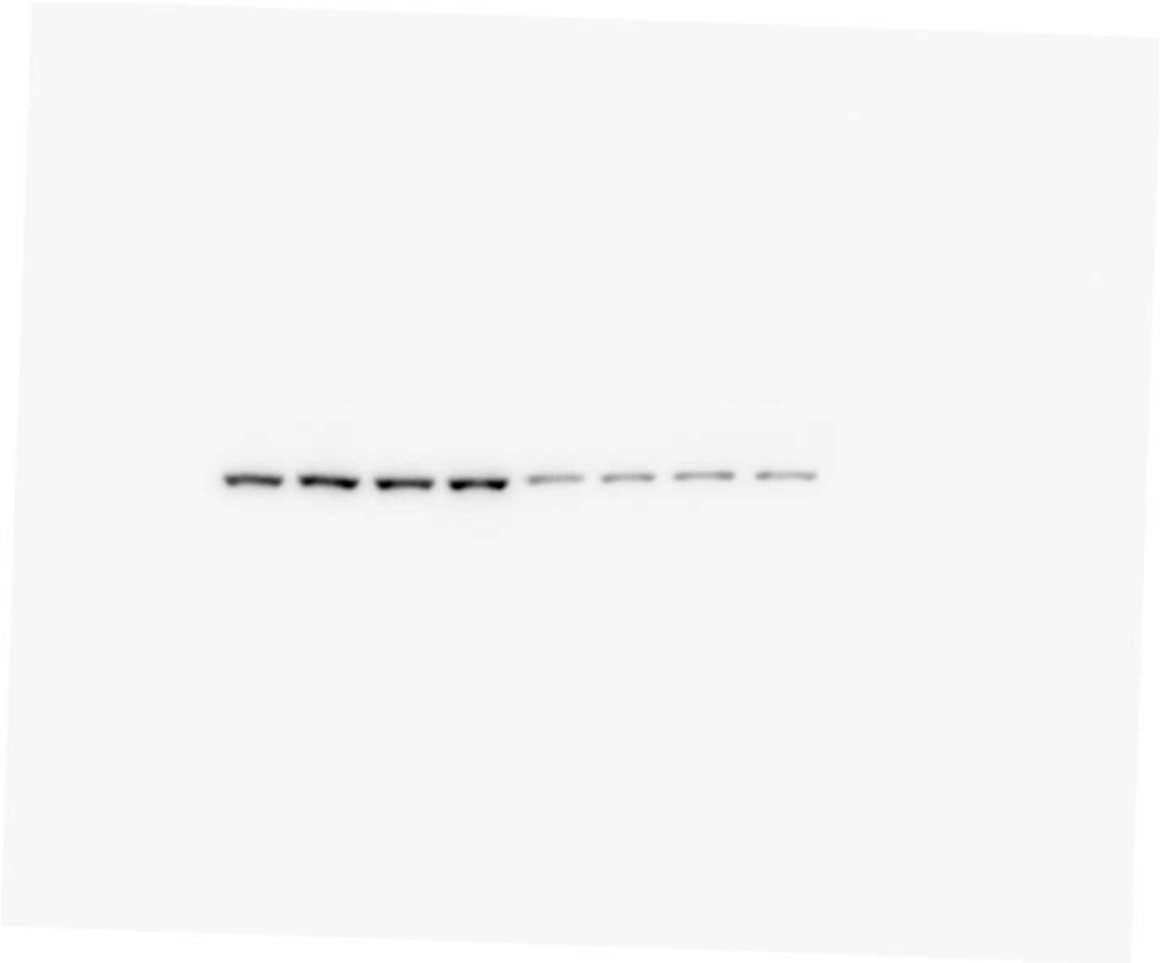

Fig 5D (HT29 cells)

IB: SLC44A2

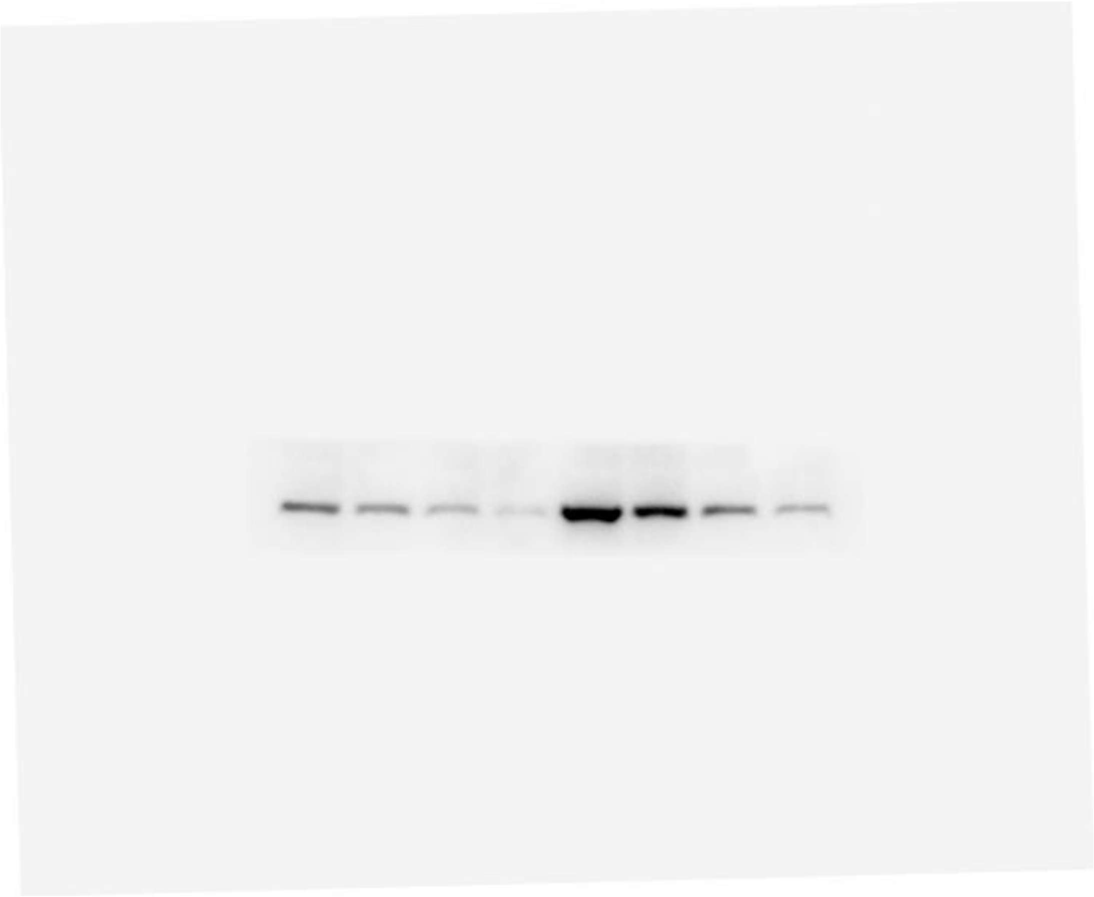

Fig 5D (HT29 cells)

IB: CPT2

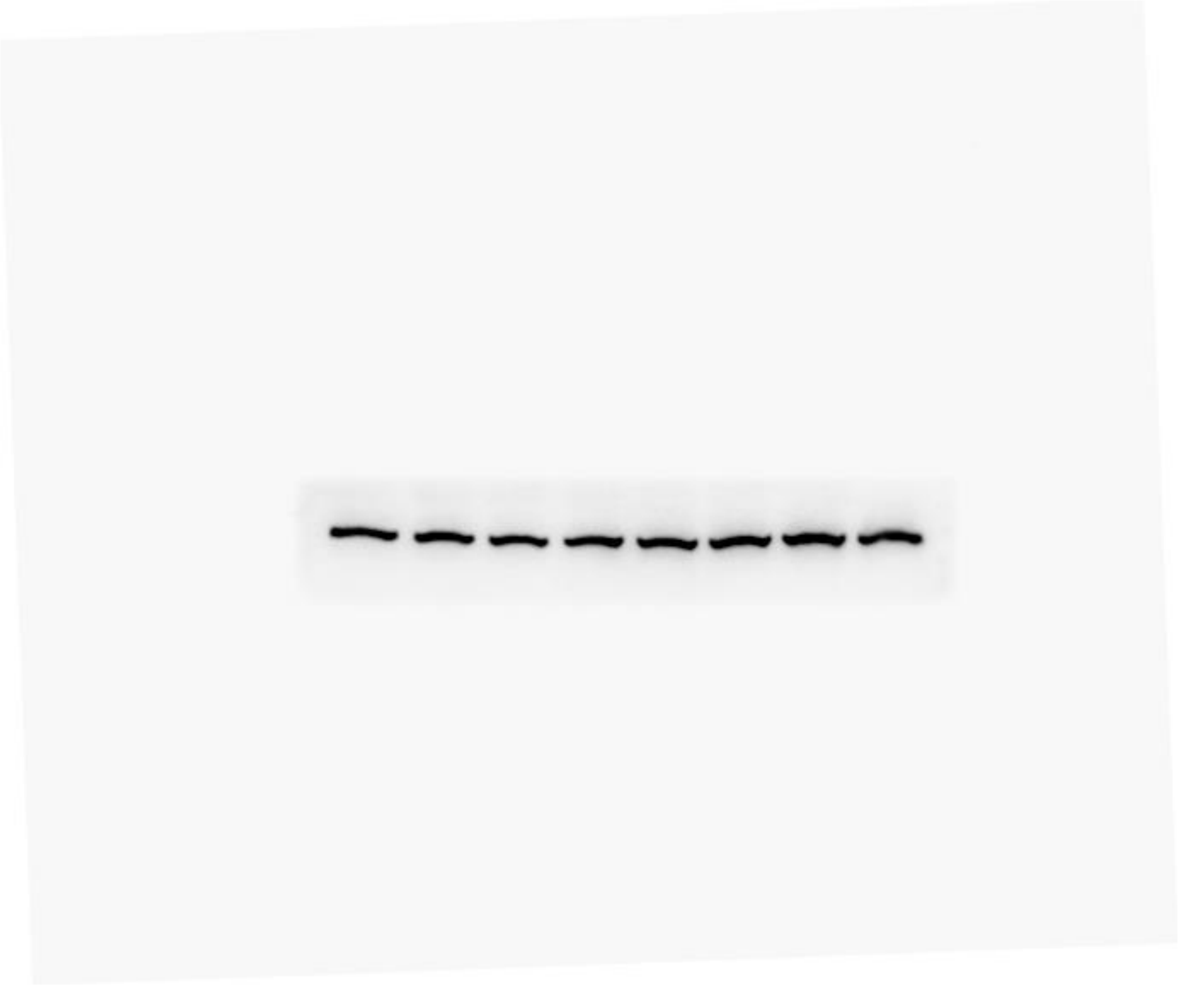

Fig 5D (HT29 cells)

IB: β-actin

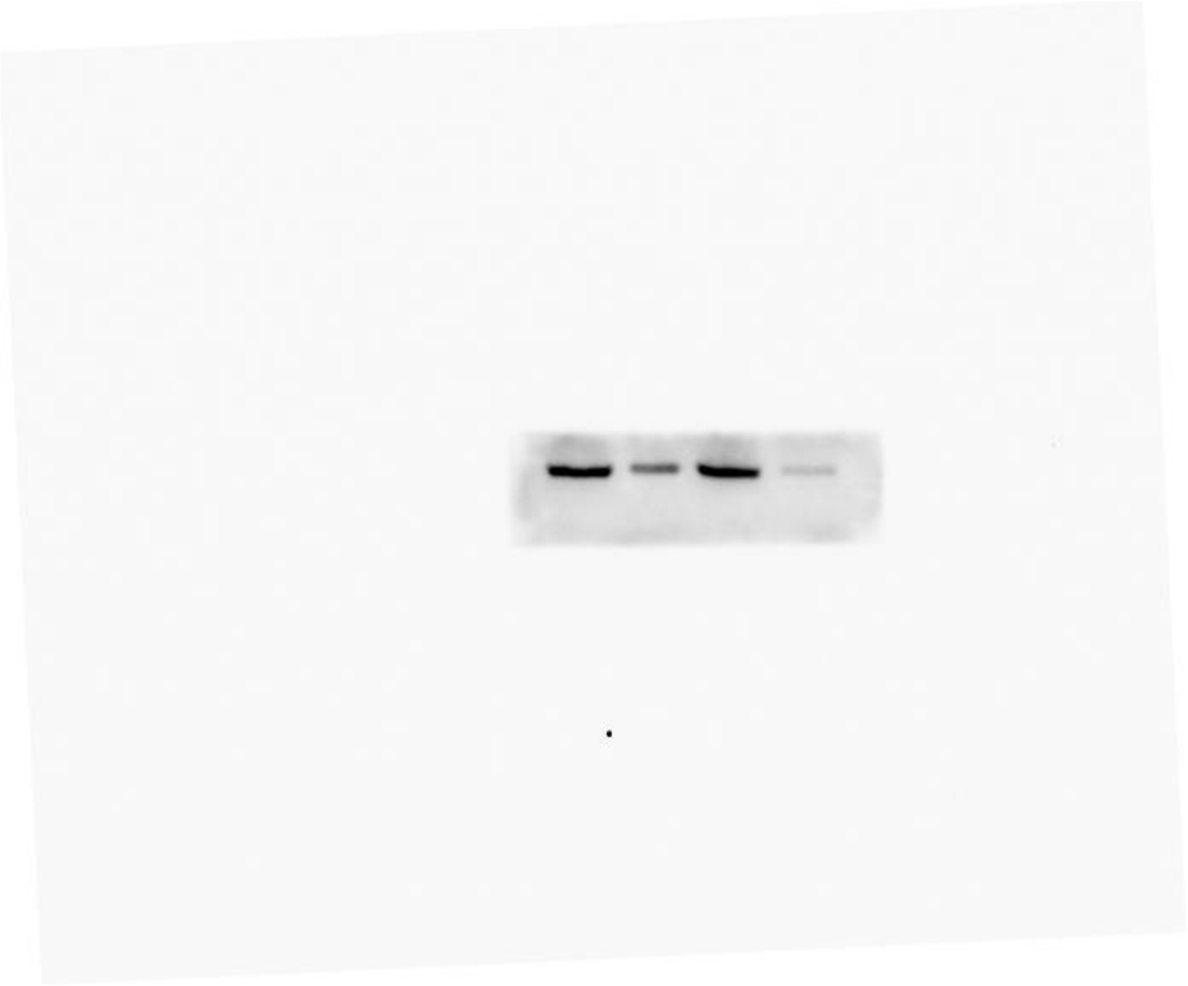

Fig 5E (HCT116 cells)

IB: CPT2

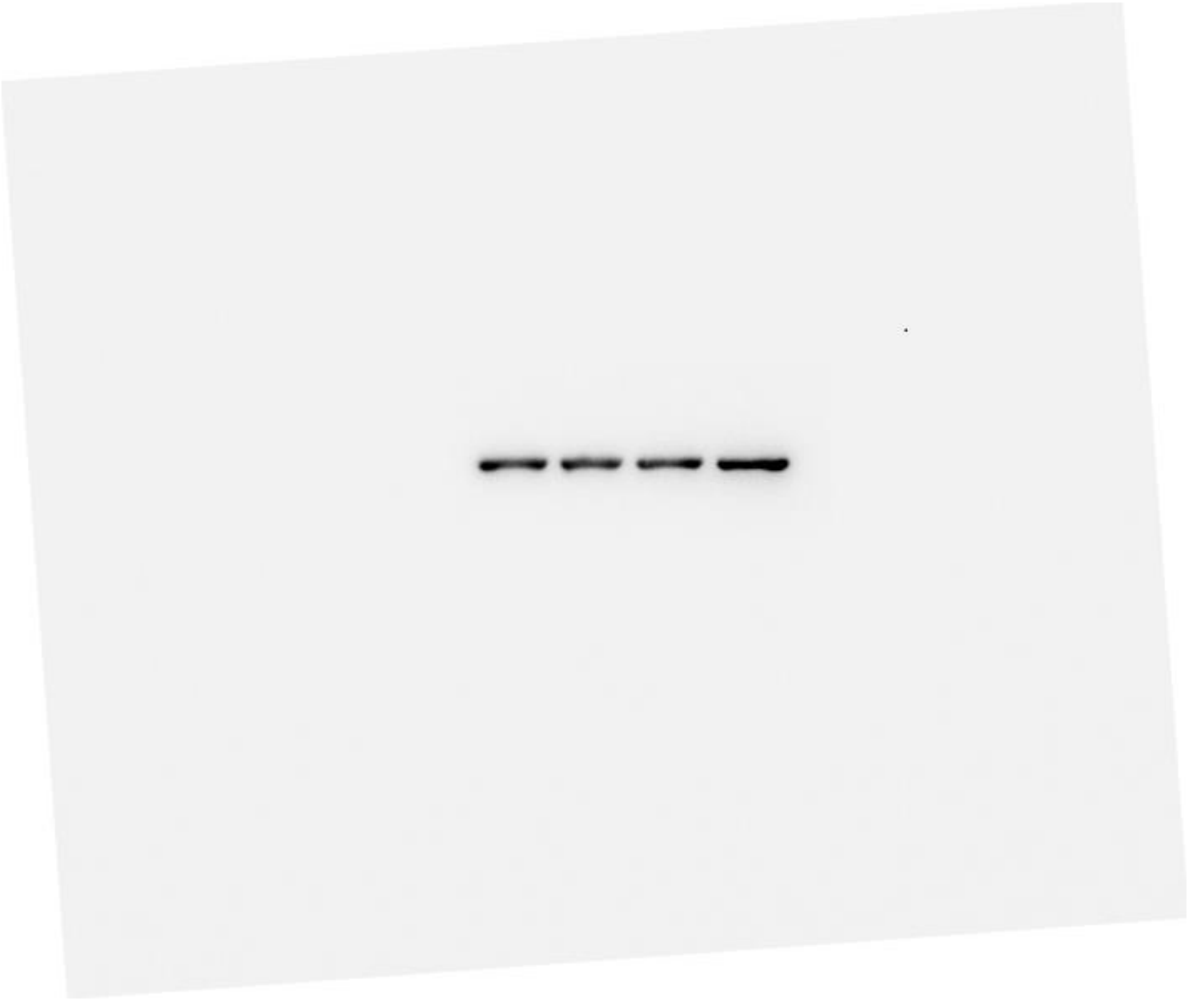

Fig 5E (HCT116 cells)

IB: β-actin

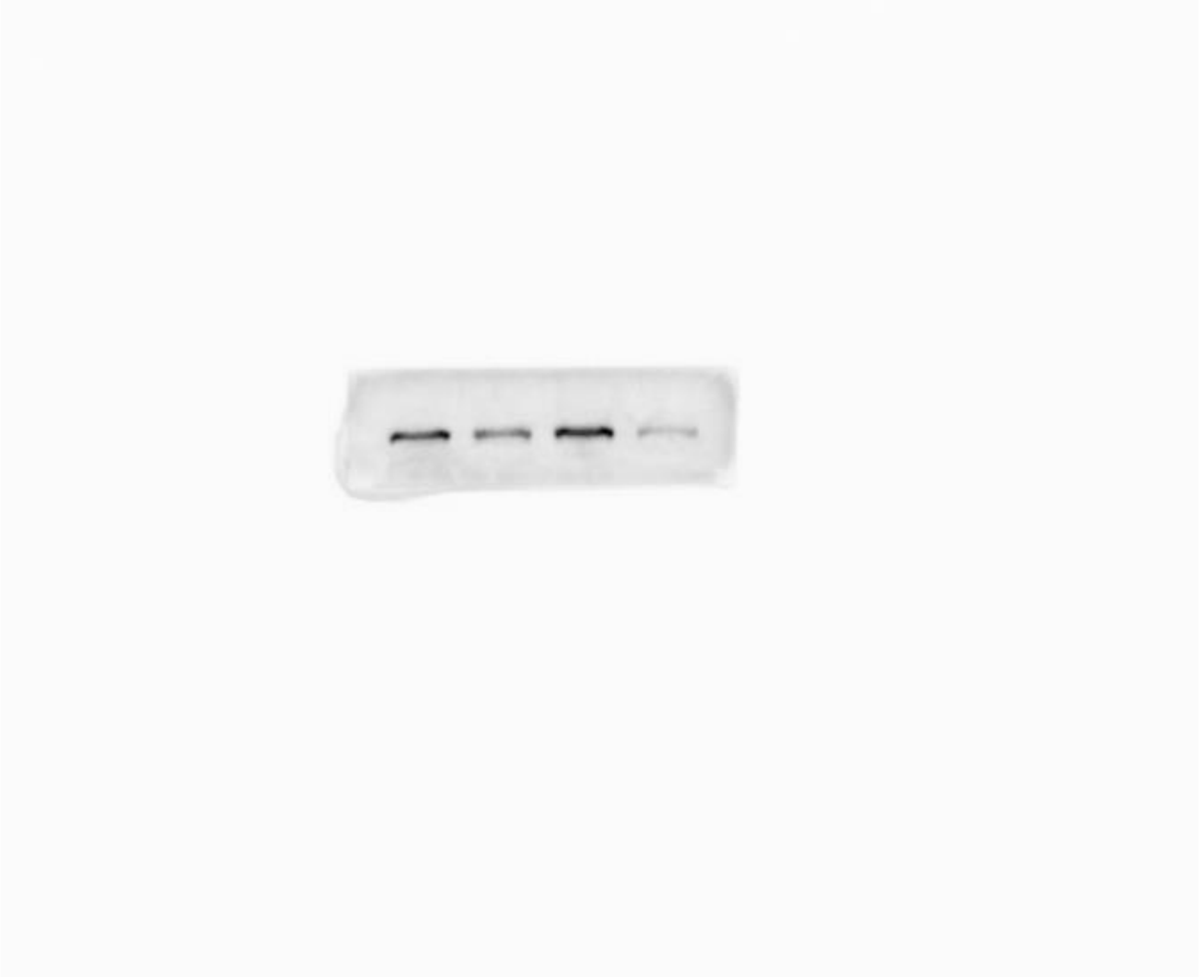

Fig 5E (LS174T cells)

IB: CPT2

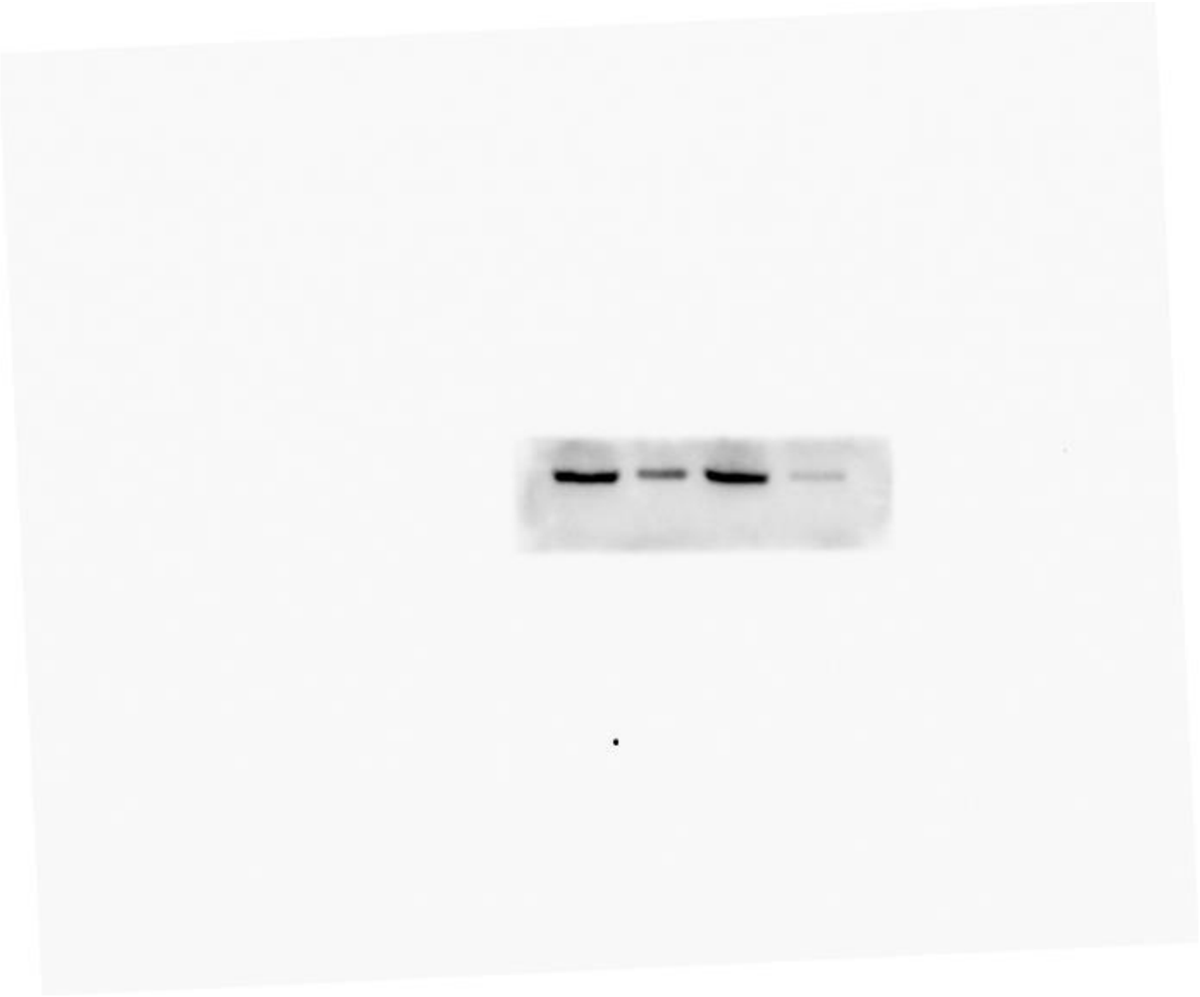

Fig 5E (LS174T cells)

IB: β-actin

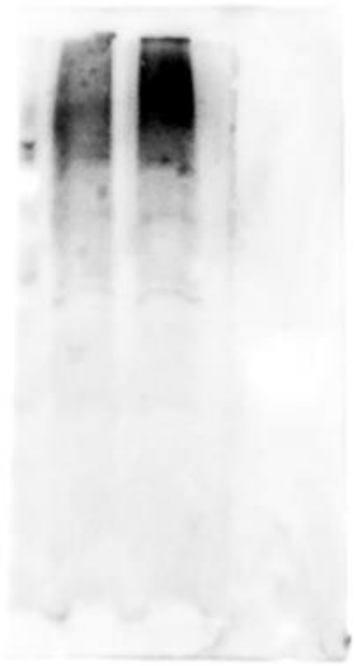

Fig 5F (HCT116 cells)

IB: UB

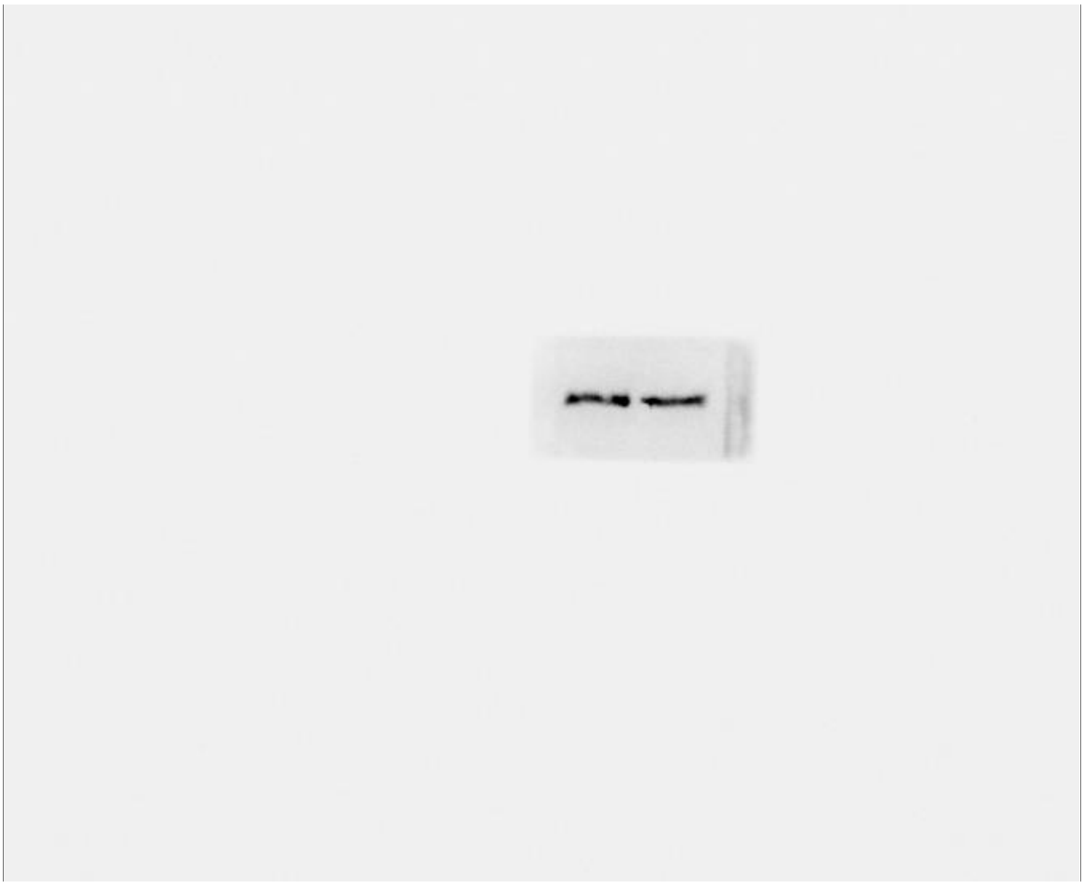

Fig 5F (HCT116 cells)

IB: CPT2

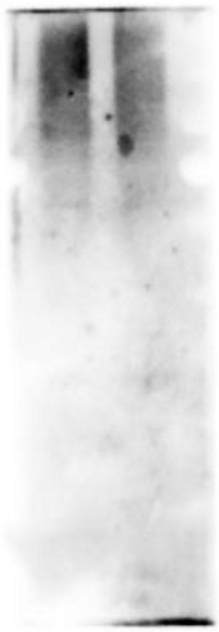

Fig 5F (HT29 cells)

IB: UB

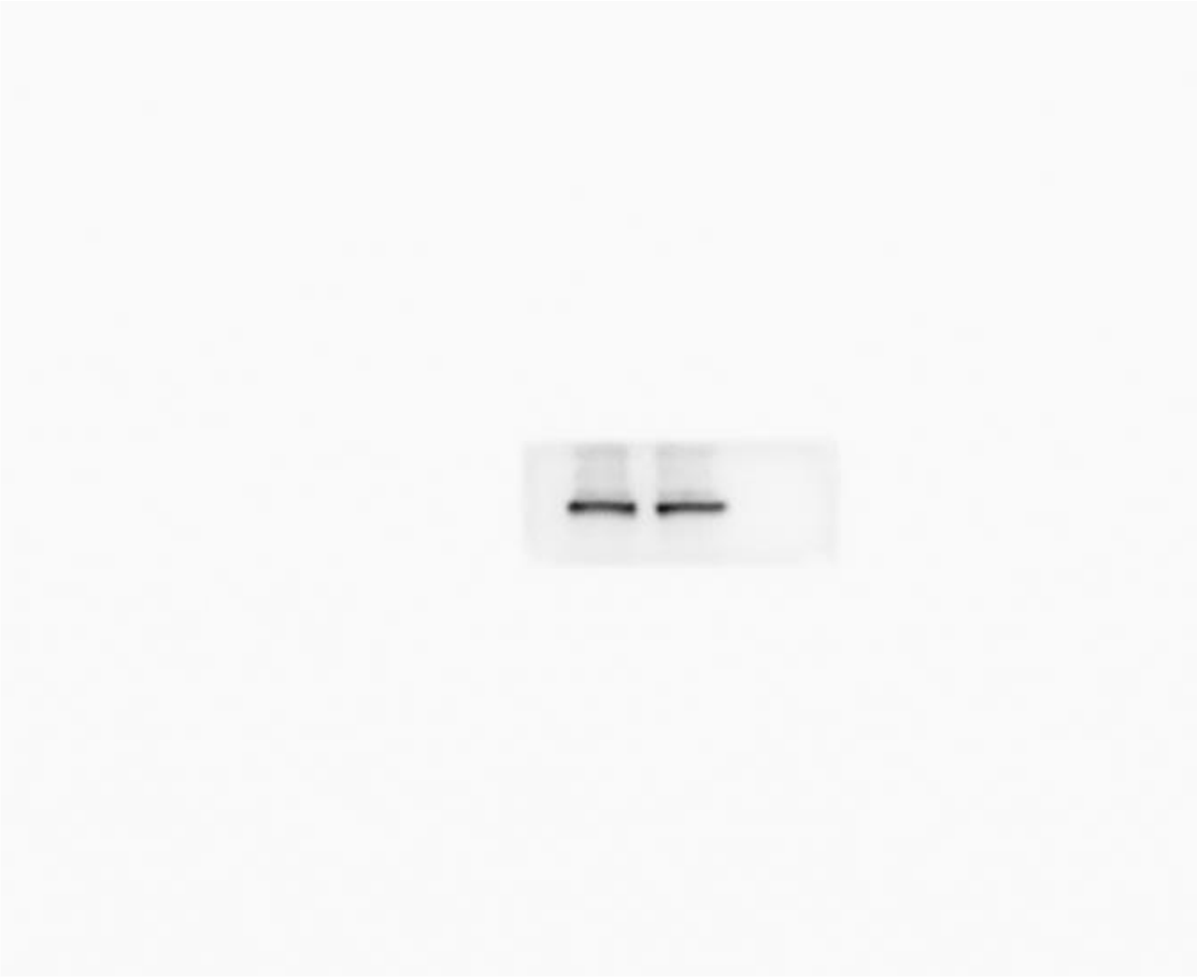

Fig 5F (HT29 cells)

IB: CPT2

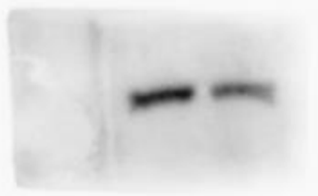

Fig 5H (HCT116 cells)

IB: Ac-K

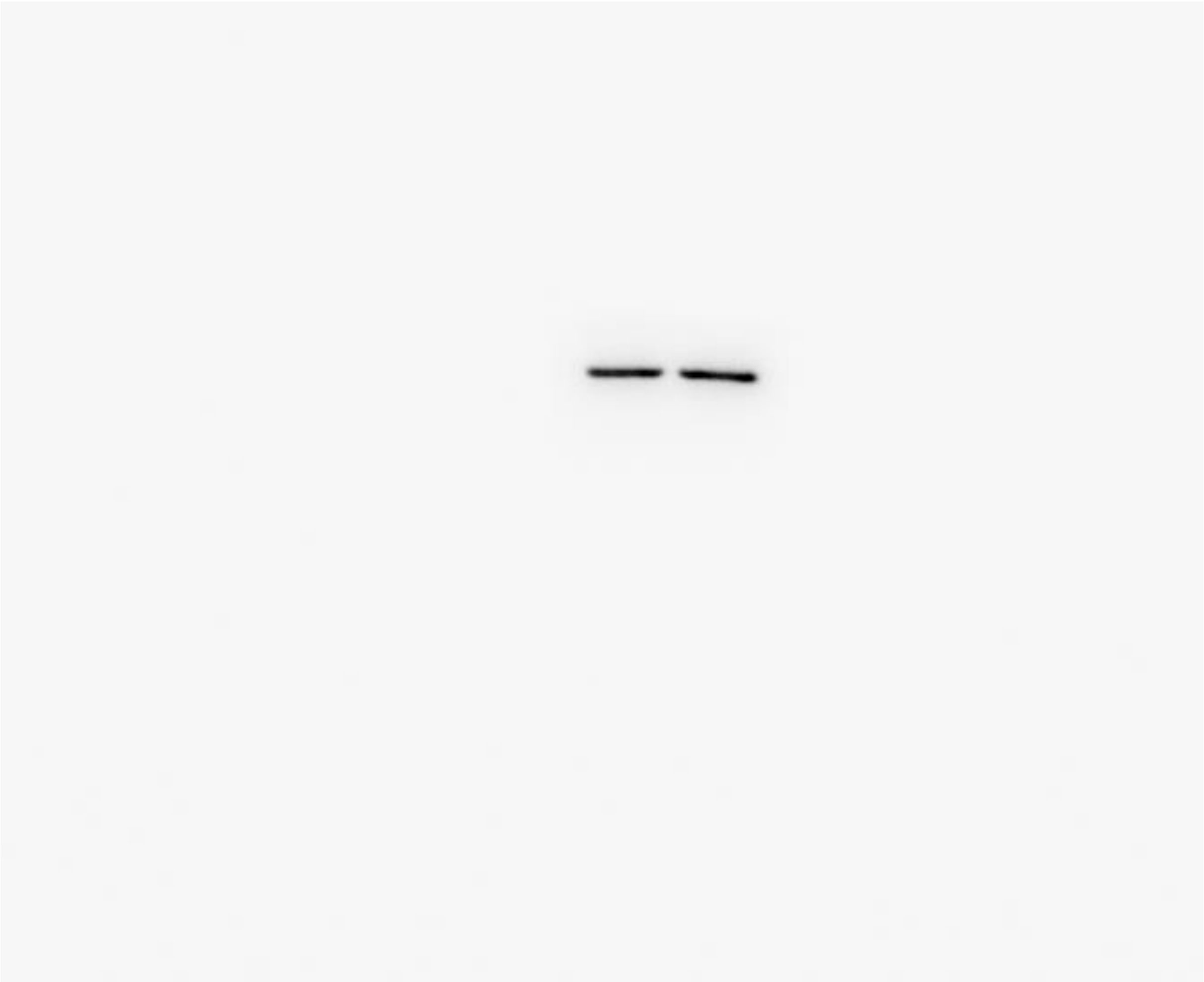

Fig 5H (HCT116 cells)

IB: CPT2

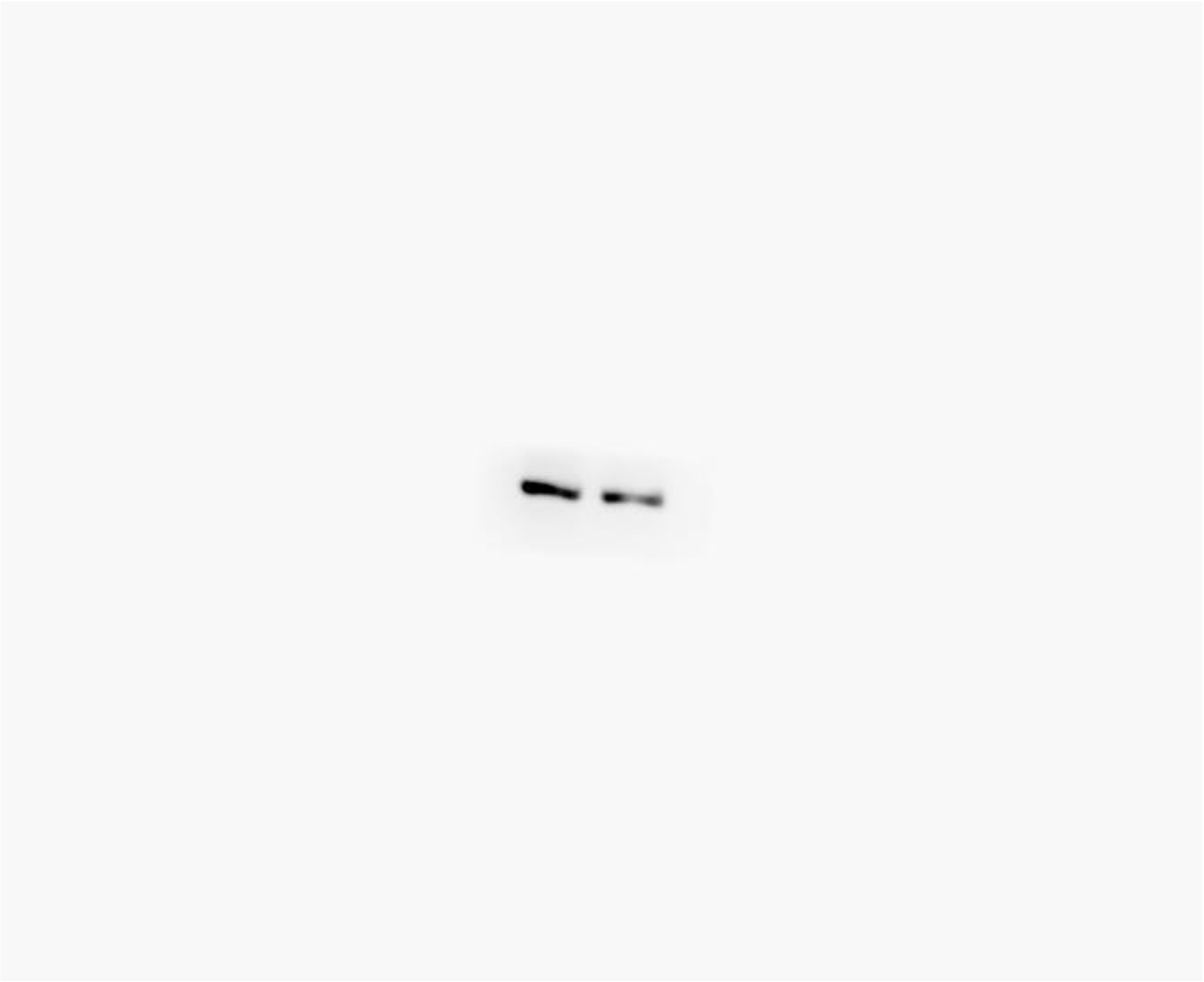

Fig 5H (LS174T cells)

IB: Ac-K

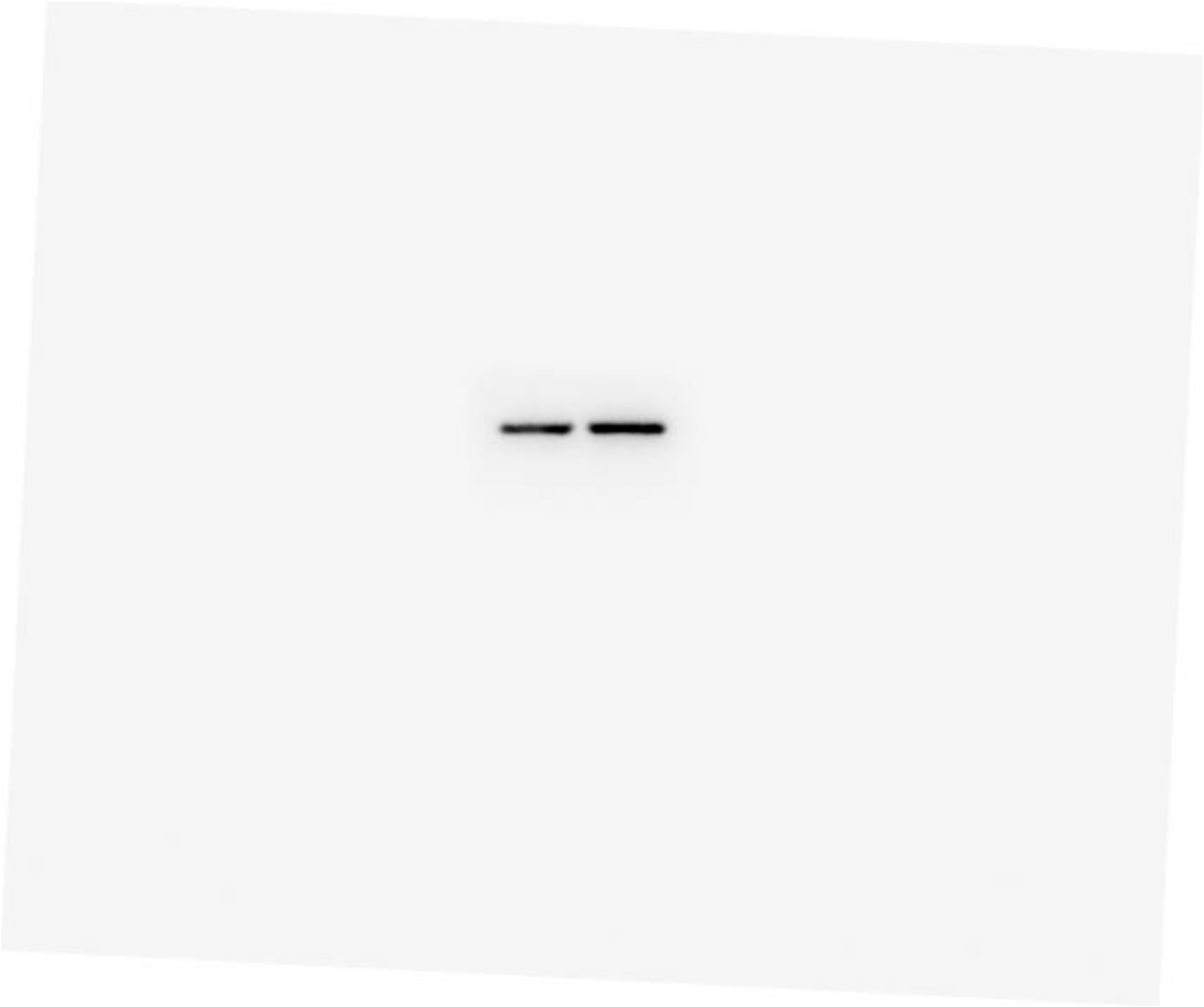

Fig 5H (LS174T cells)

IB: CPT2

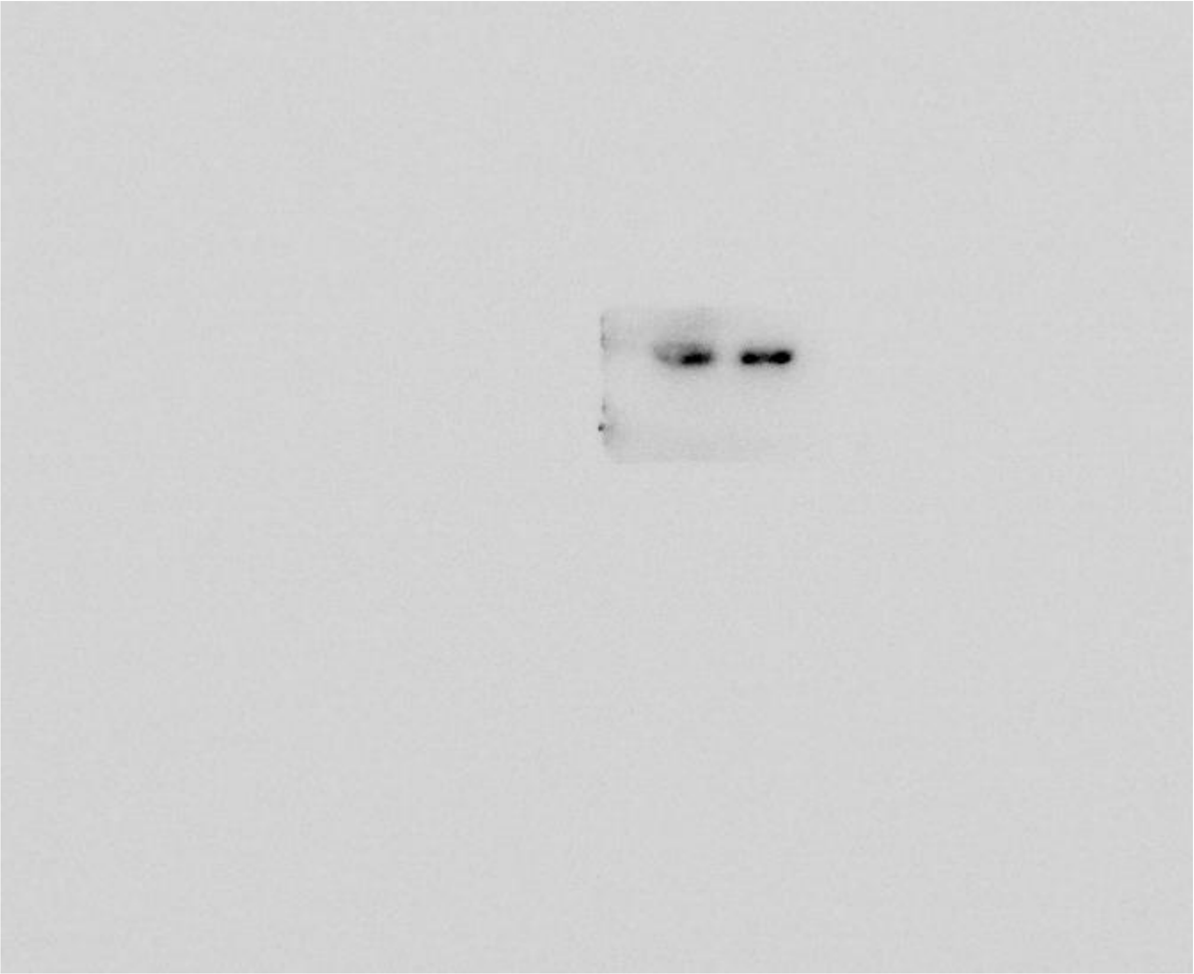

Fig 5H (HT29 cells)

IB: Ac-K

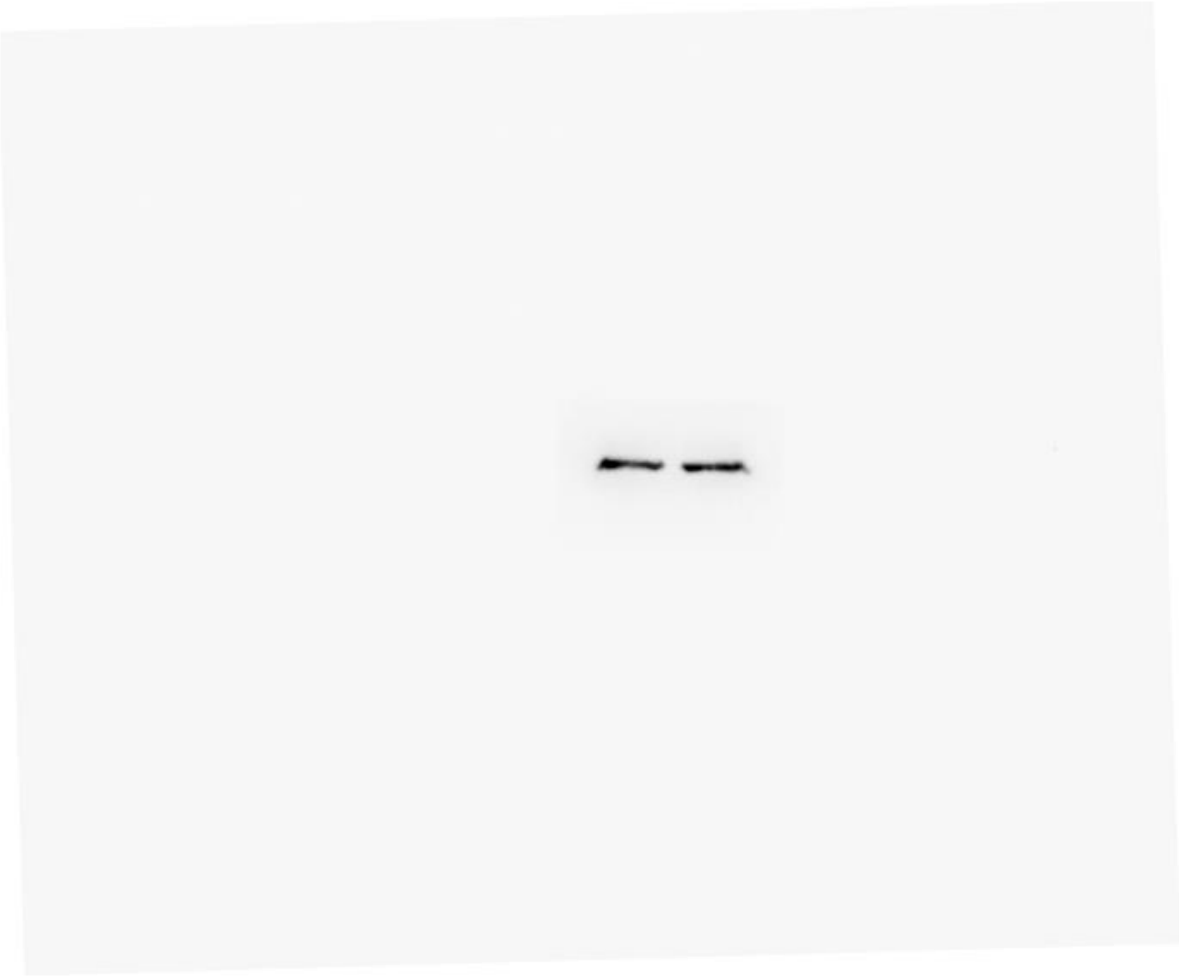

Fig 5H (HT29 cells)

IB: CPT2

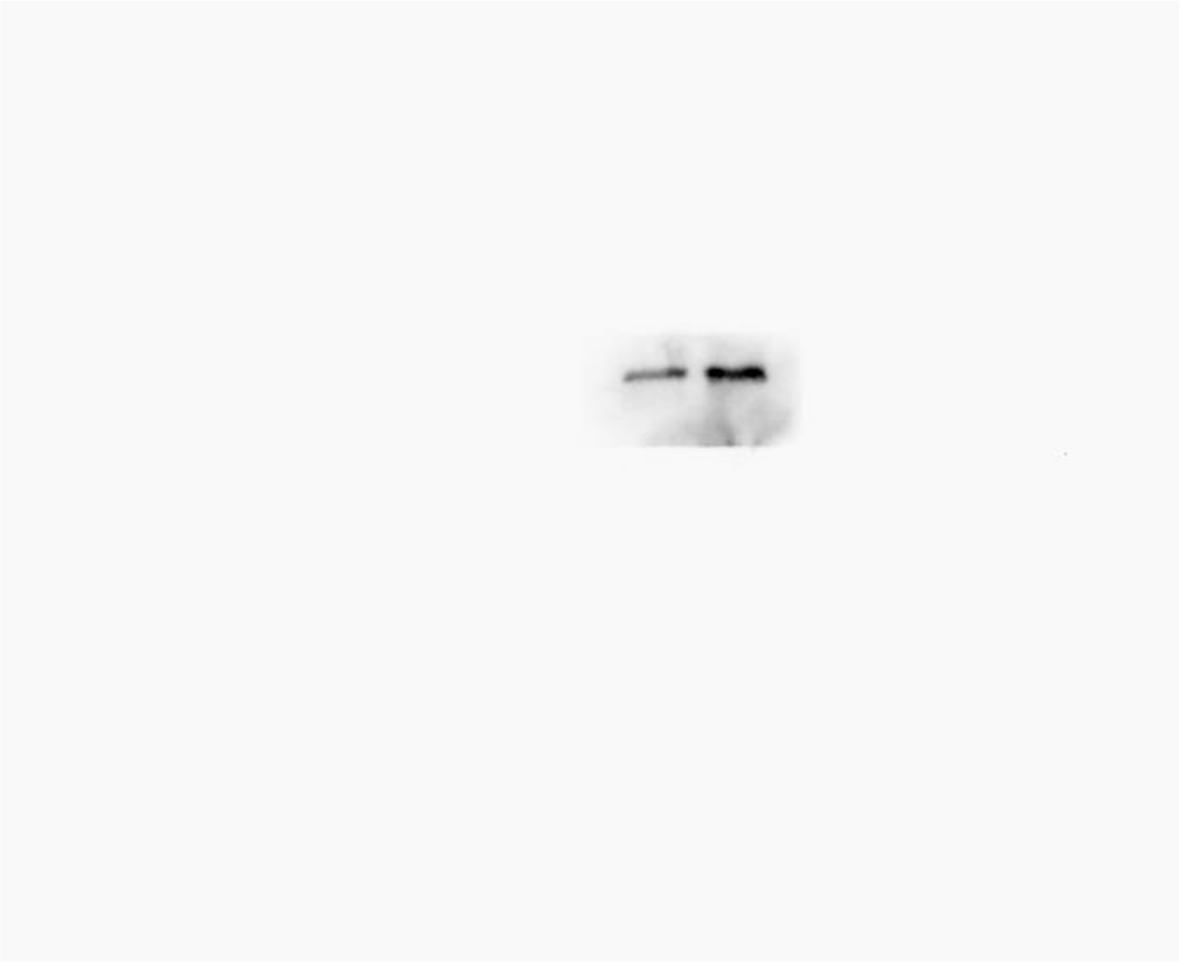

Fig 5H (SW480 cells)

IB: Ac-K

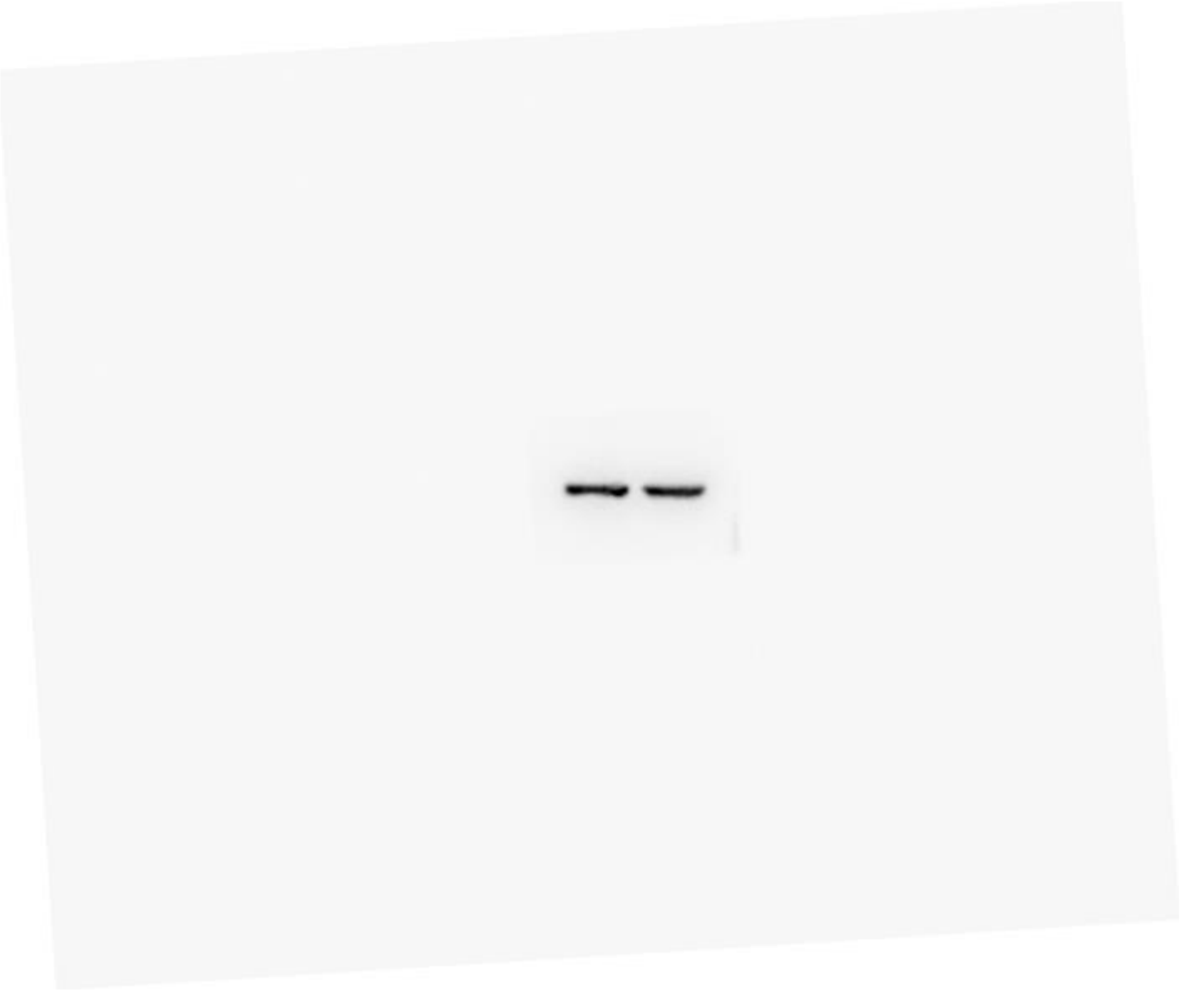

Fig 5H (SW480 cells)

IB: CPT2

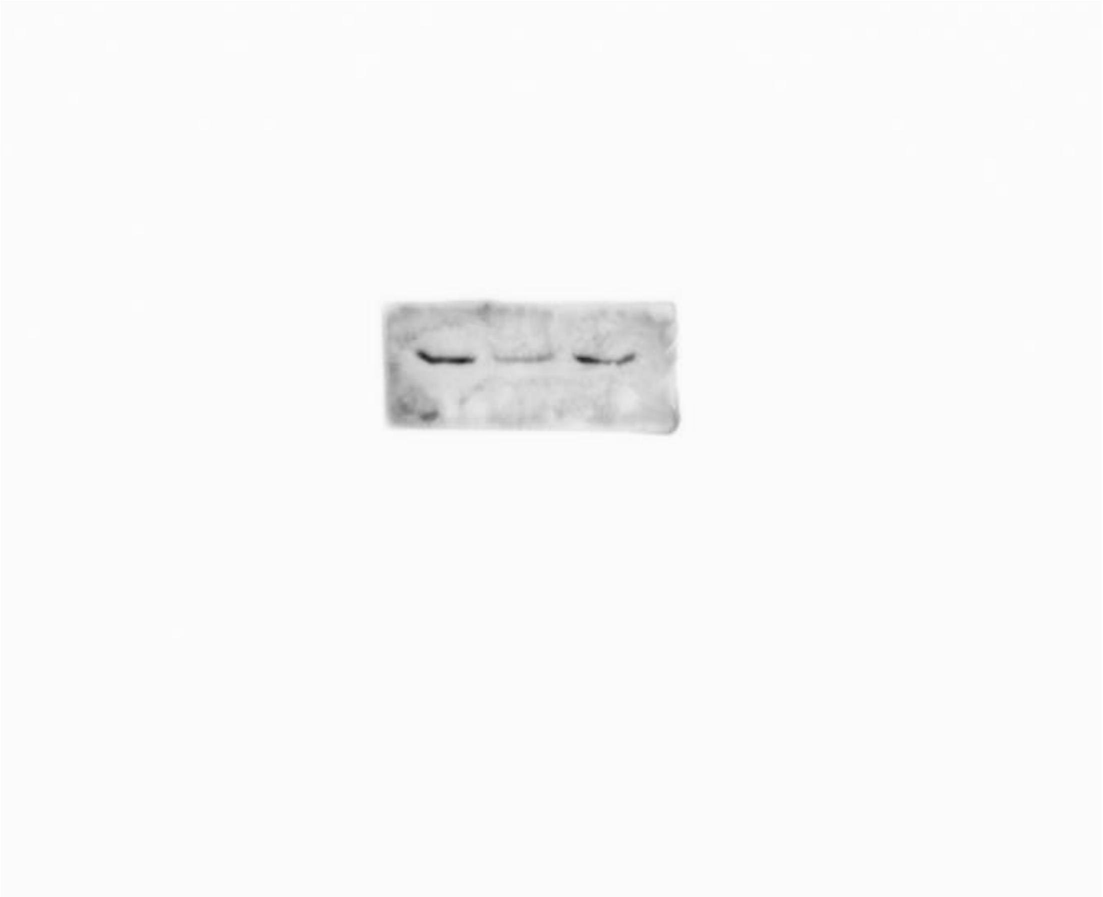

Fig 5I (HCT116 cells)

IB: Ac-K

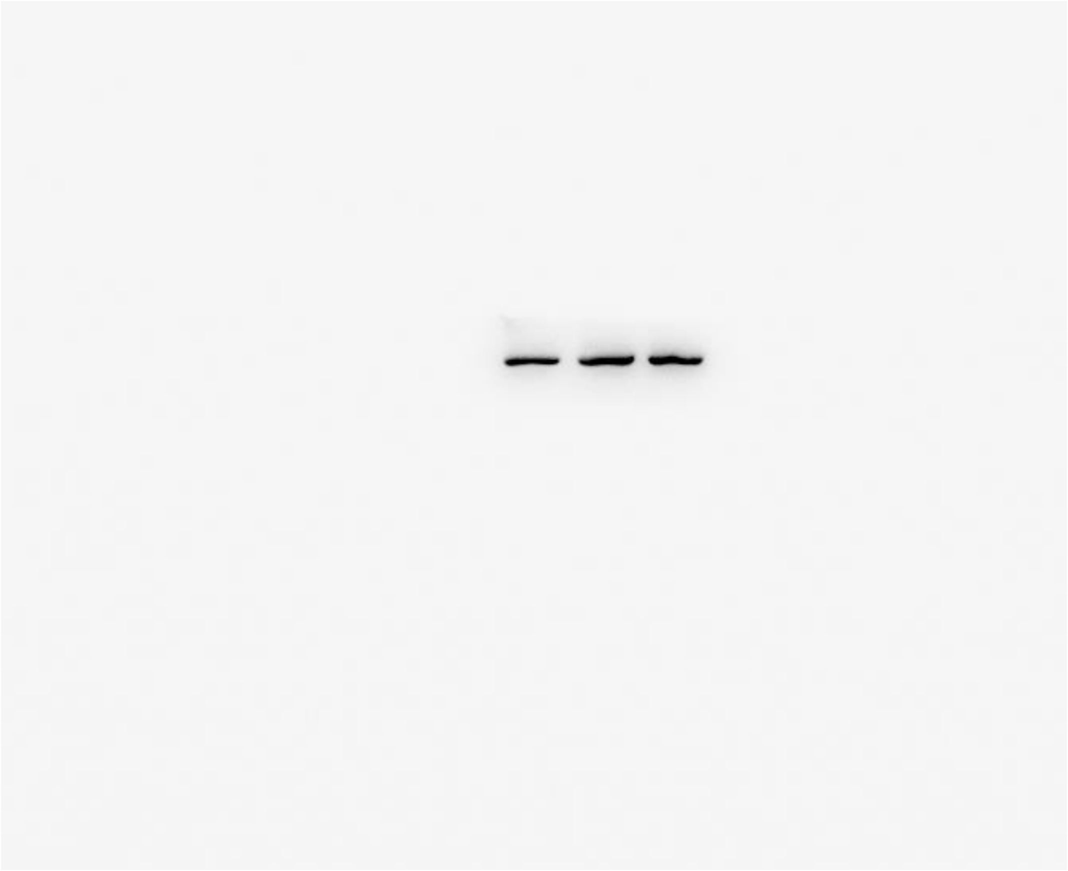

Fig 5I (HCT116 cells)

IB: CPT2

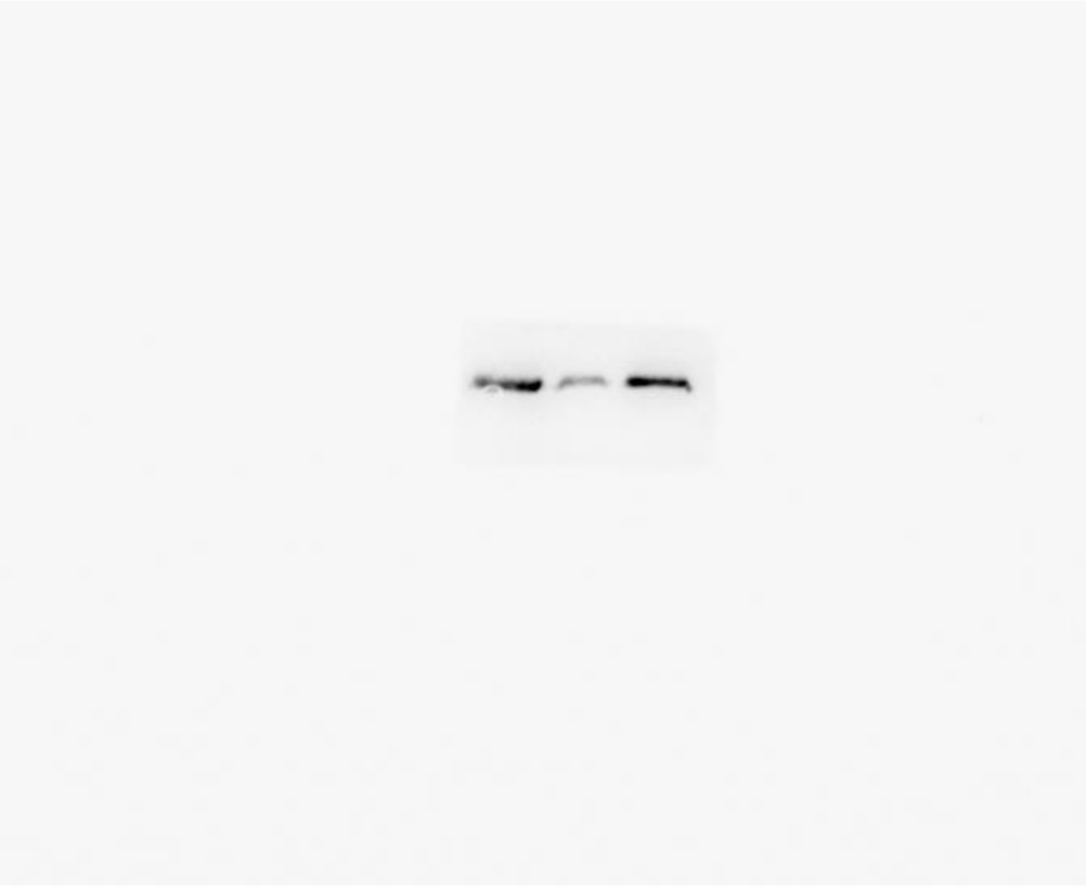

Fig 5I (LS174T cells)

IB: Ac-K

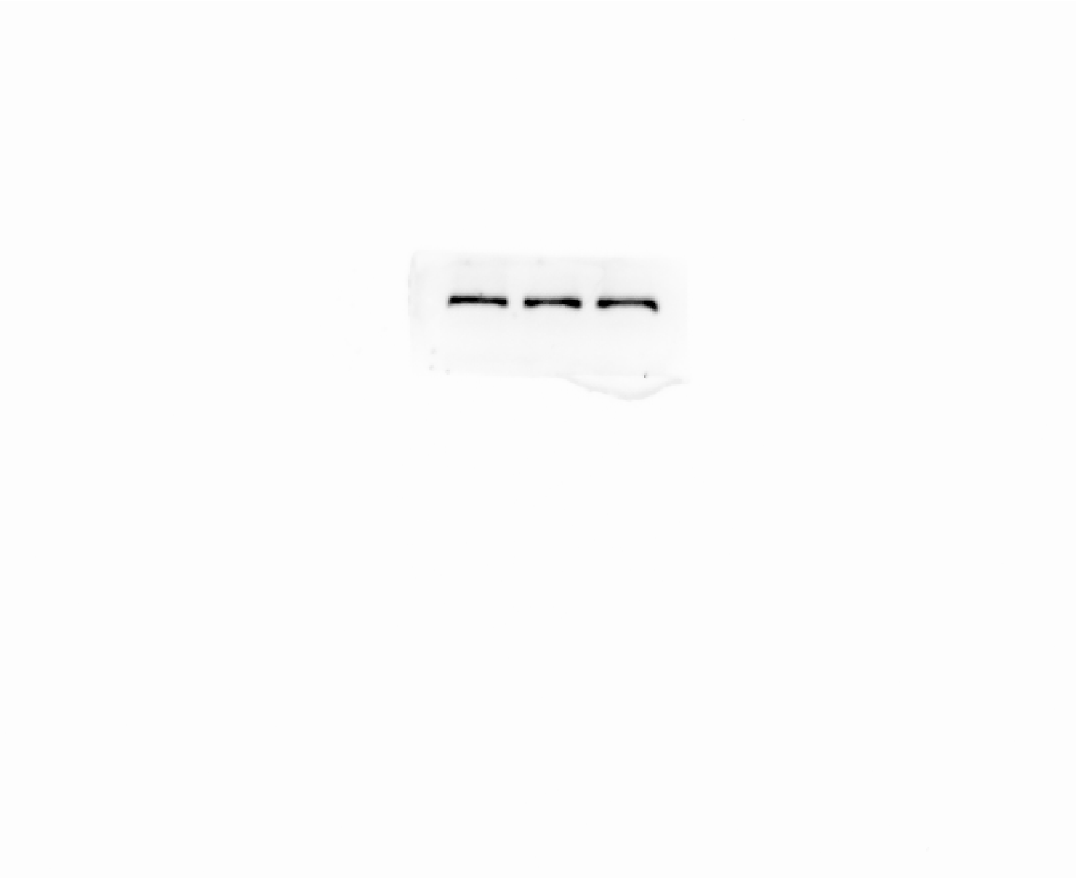

Fig 5I (LS174T cells)

IB: CPT2

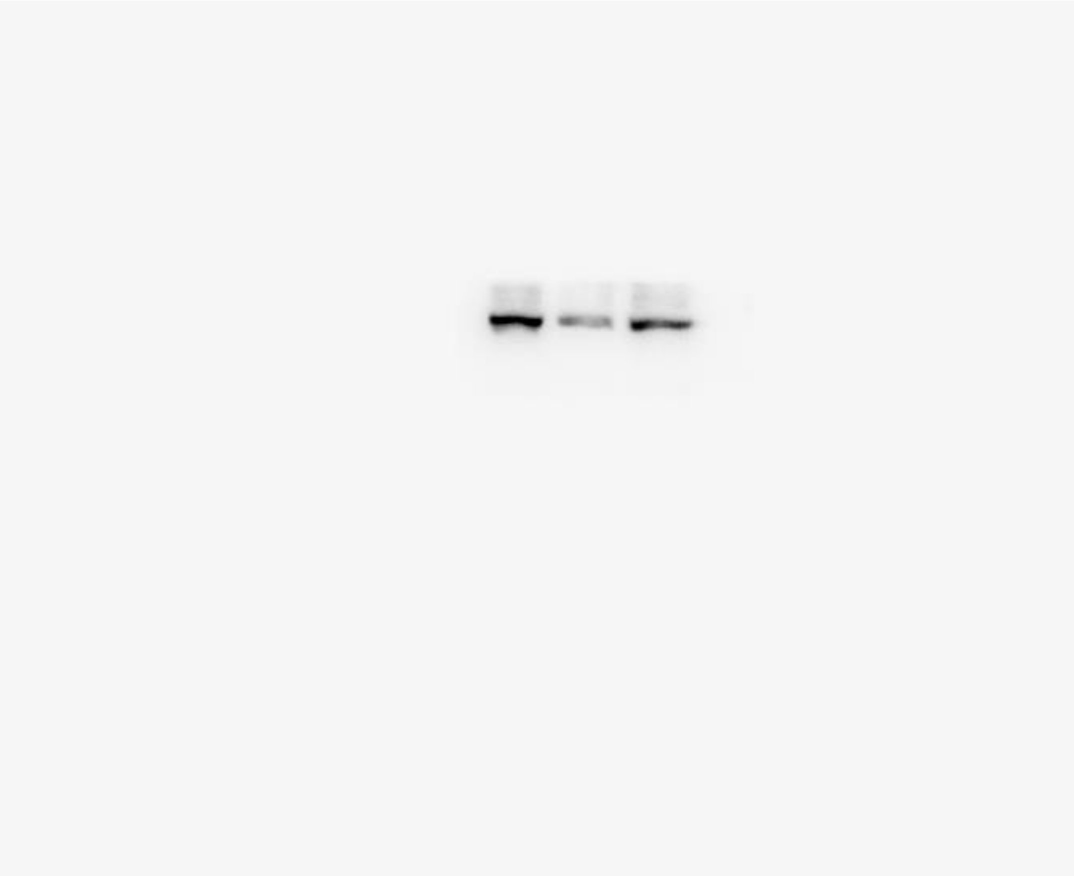

Fig 5J (HCT116 cells)

IB: CPT2

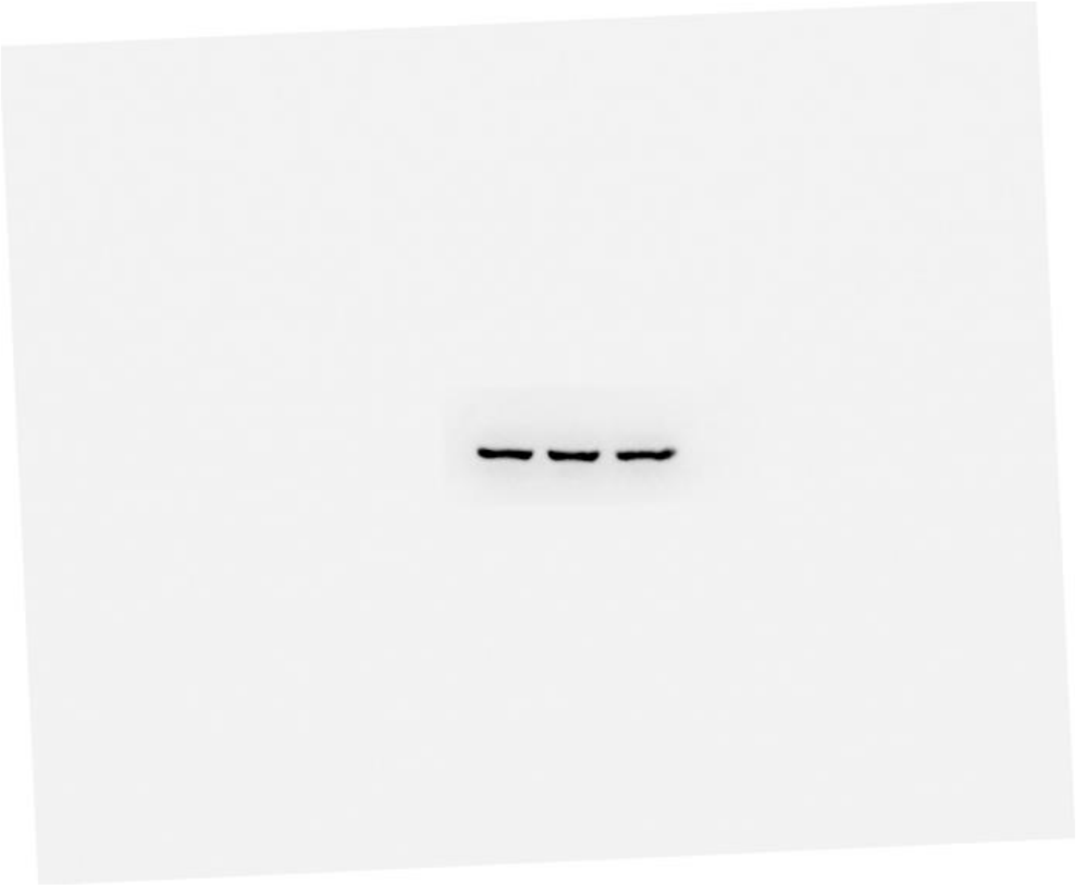

Fig 5J (HCT116 cells)

IB: β-actin

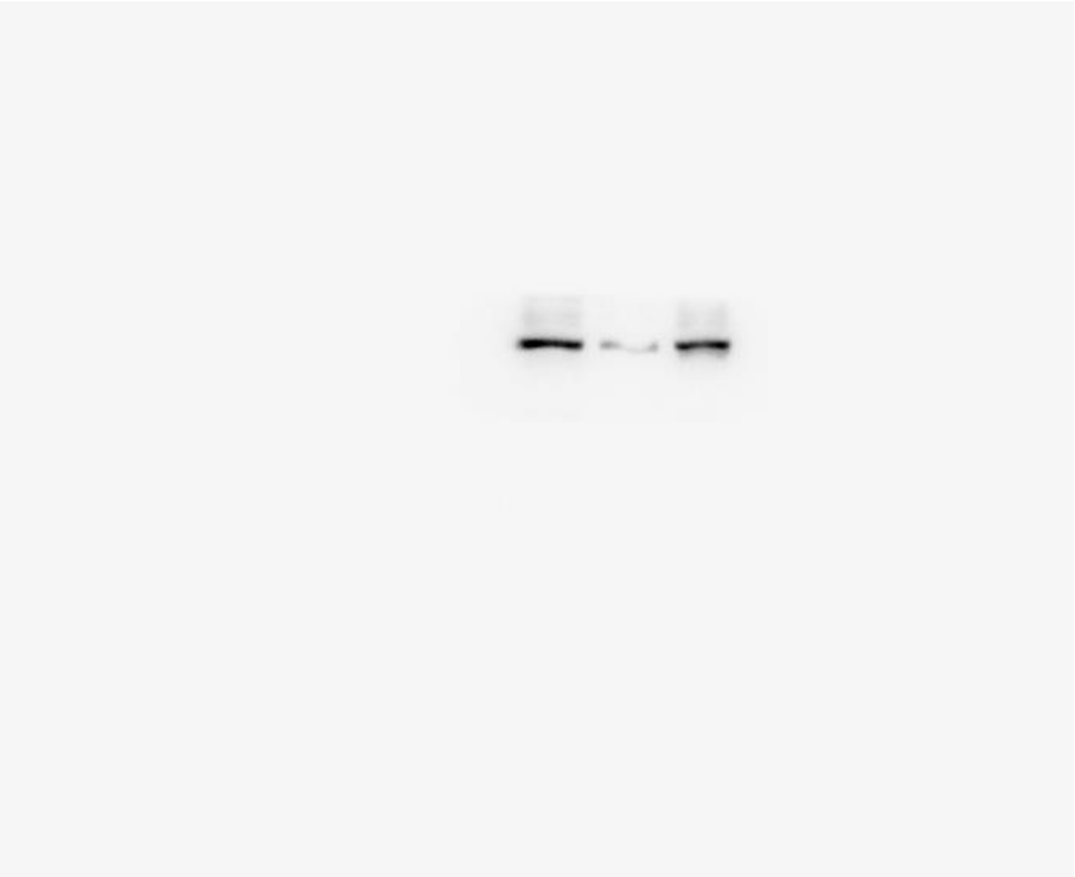

Fig 5J (LS174T cells)

IB: CPT2

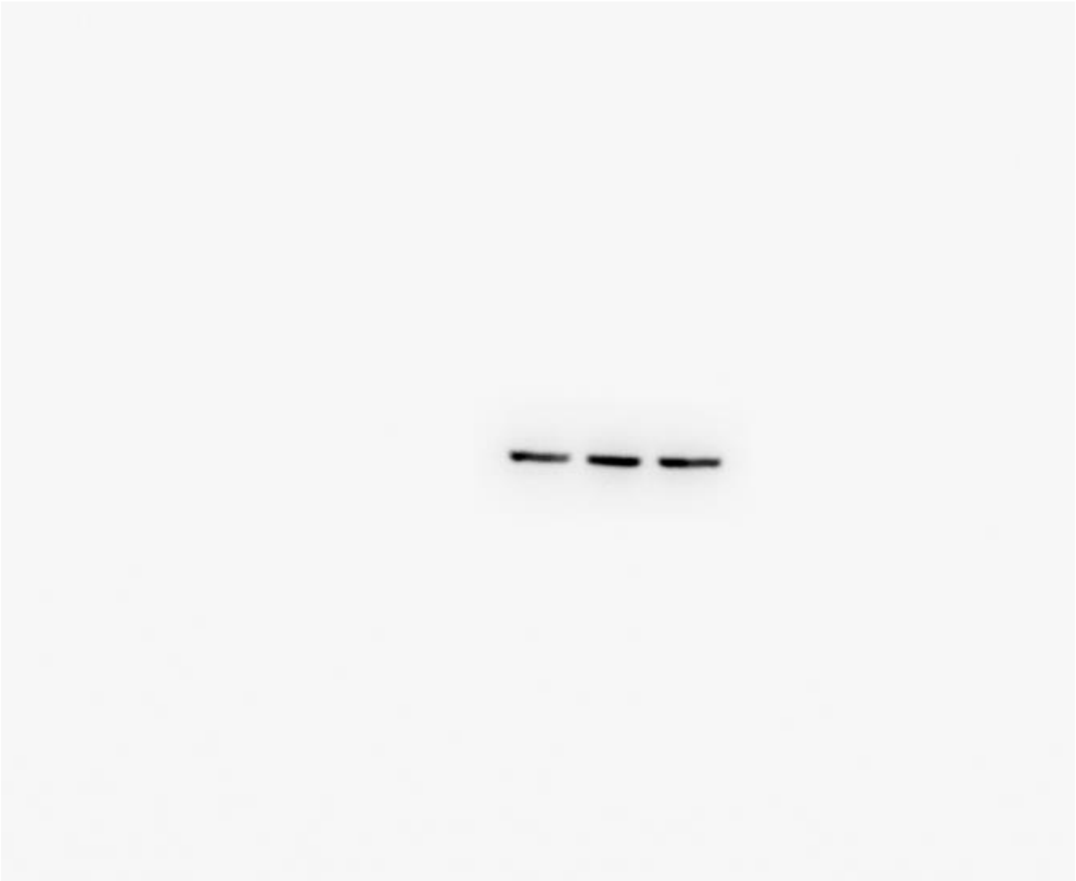

Fig 5J (LS174T cells)

IB: β-actin

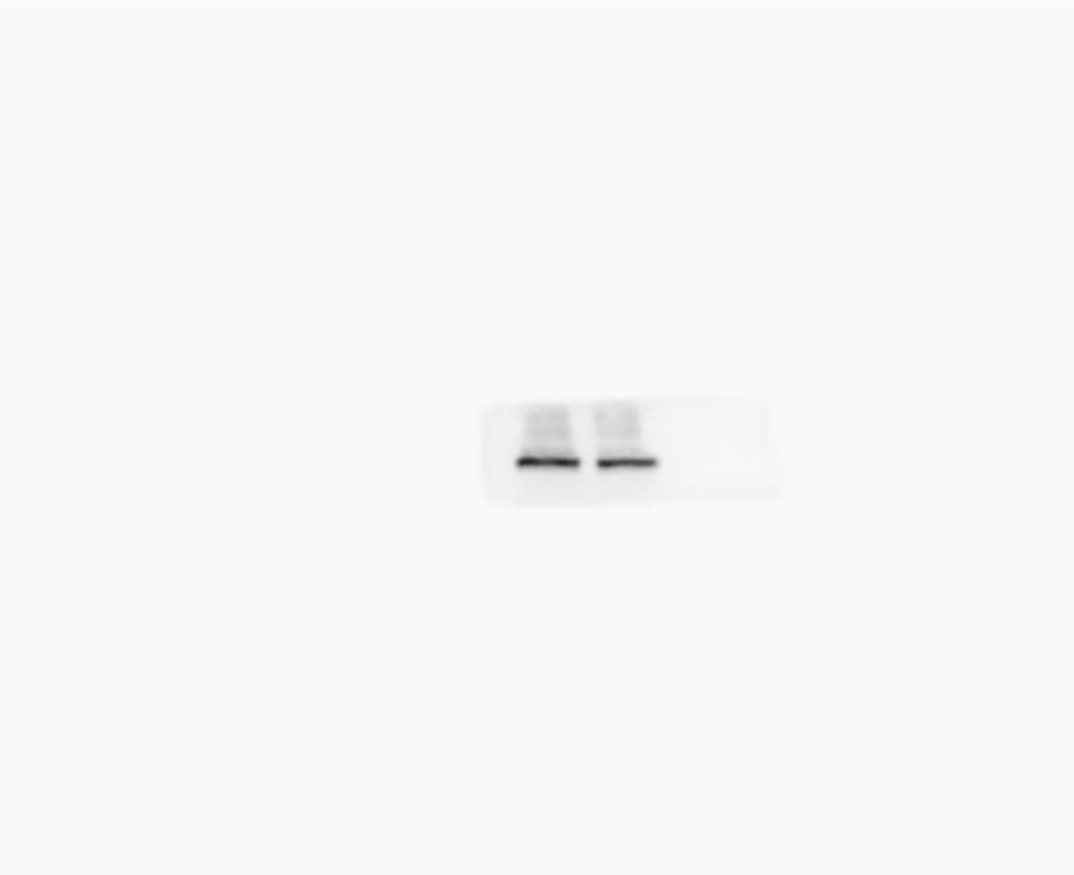

Fig 6A (HCT116 cells; EV, MARCH5)

IB: CPT2

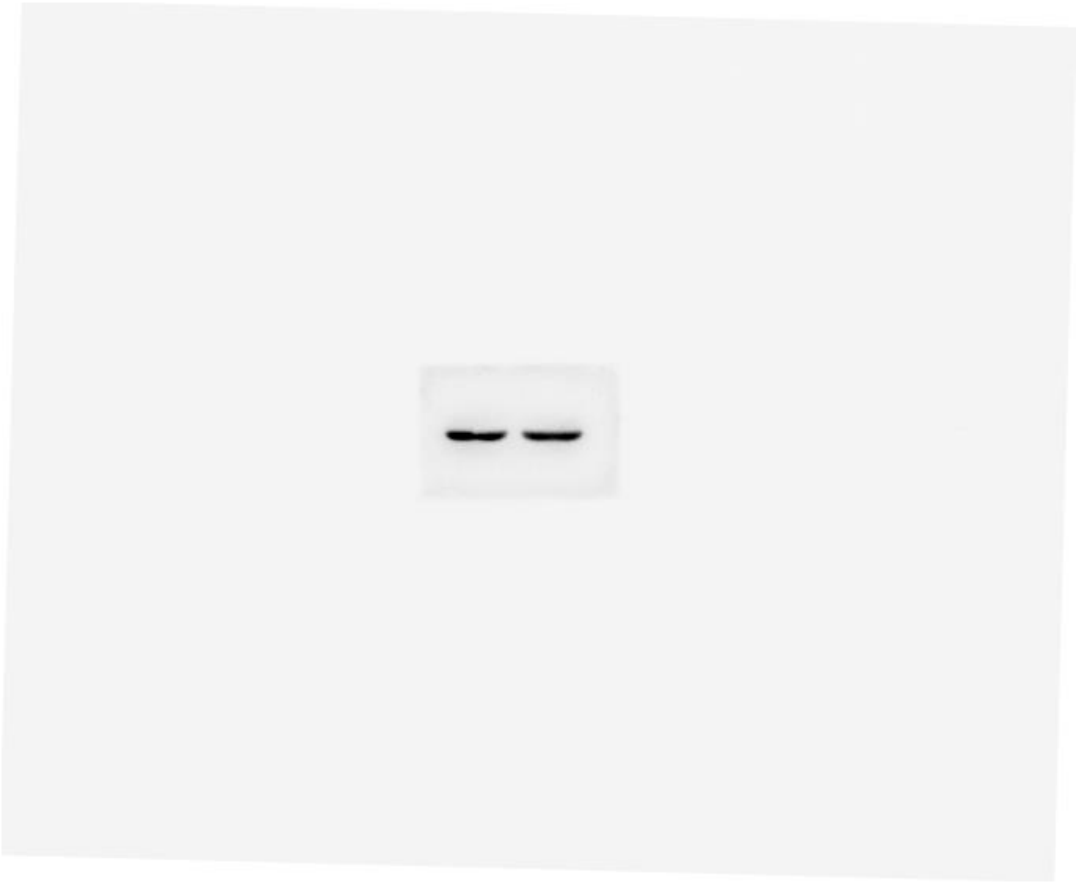

Fig 6A (HCT116 cells; EV, MARCH5)

IB: β-actin

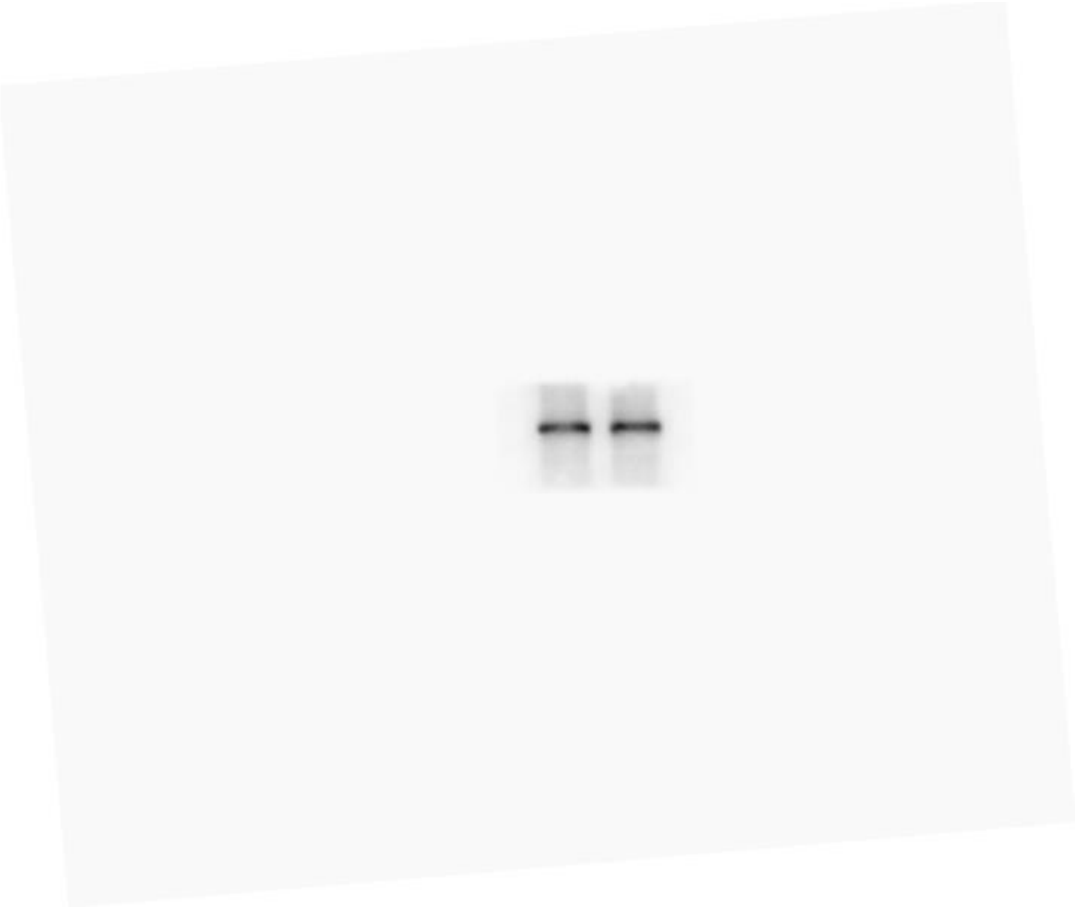

Fig 6A (HCT116 cells; EV, RNF185)

IB: CPT2

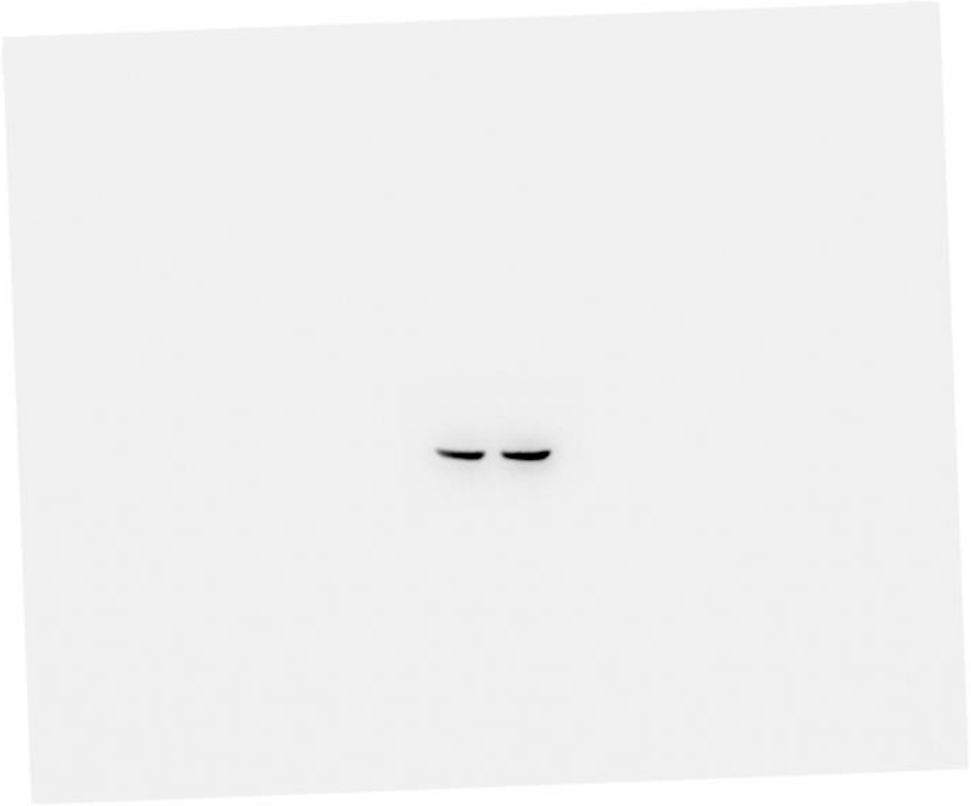

Fig 6A (HCT116 cells; EV, RNF185)

IB: β-actin

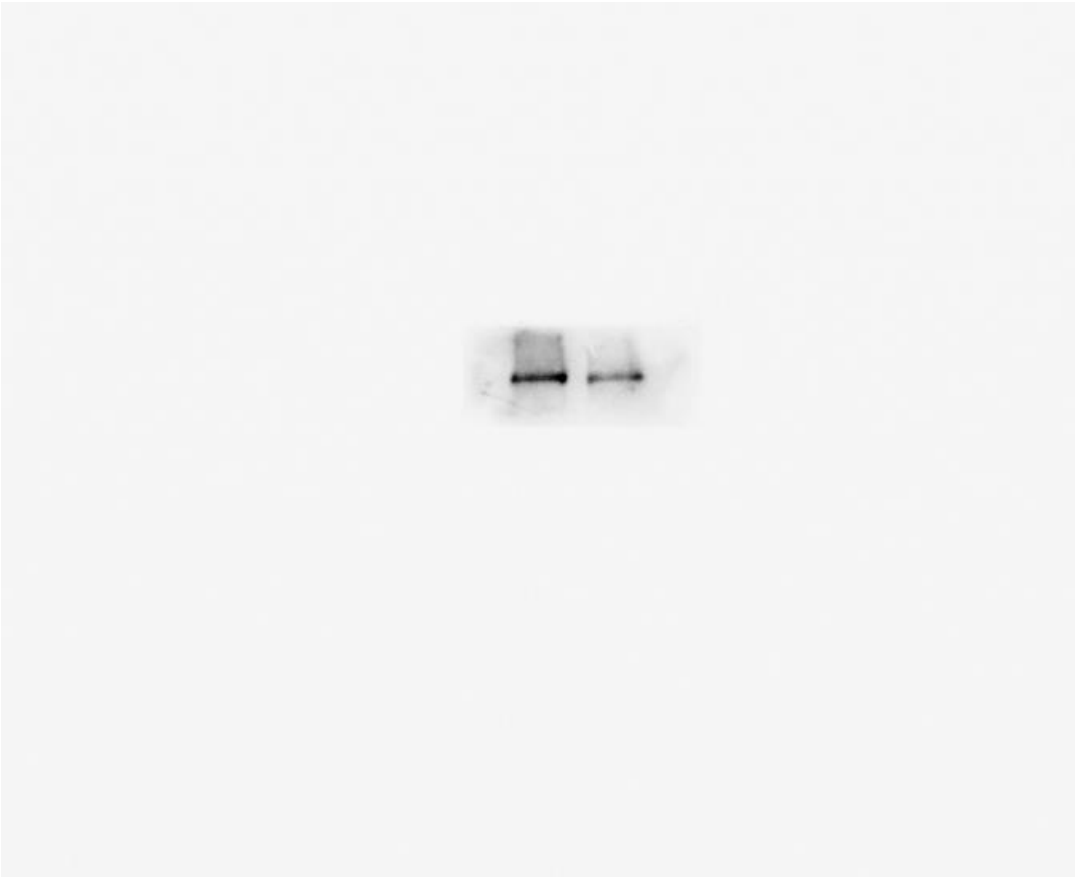

Fig 6A (HCT116 cells; EV, MUL1)

IB: CPT2

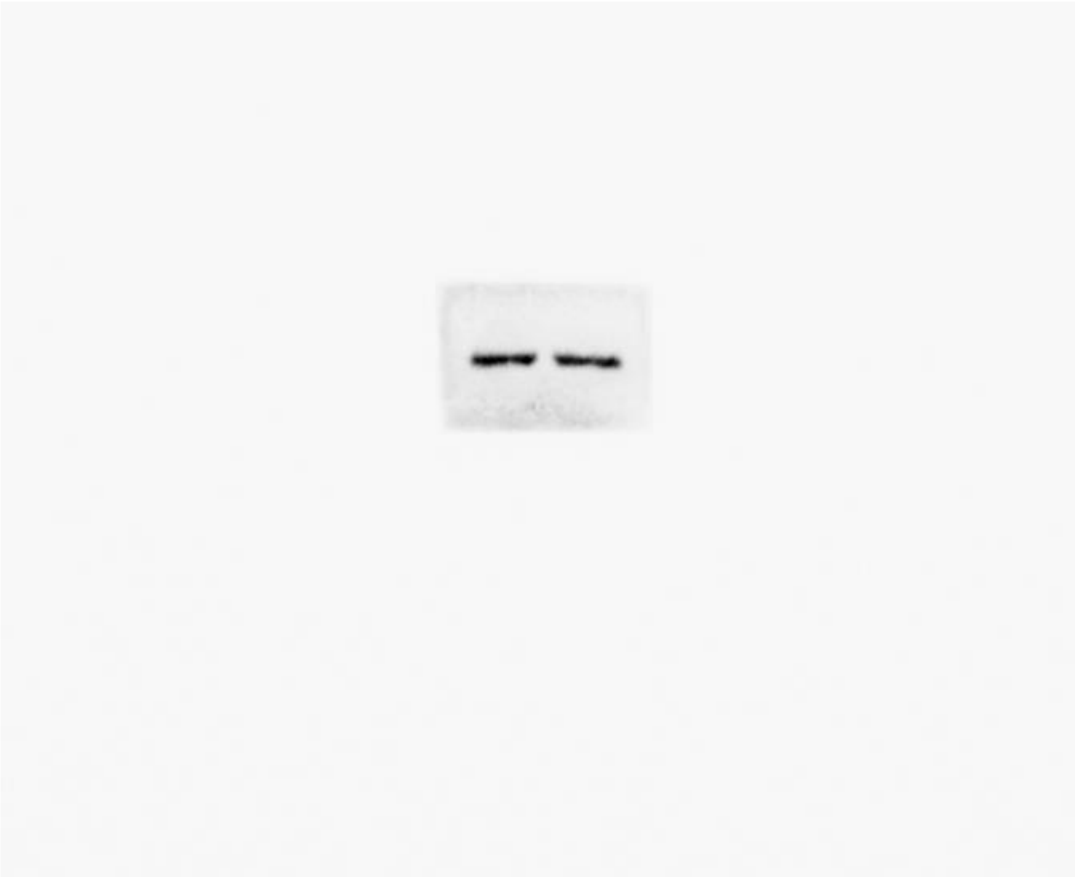

Fig 6A (HCT116 cells; EV, MUL1)

IB: β-actin

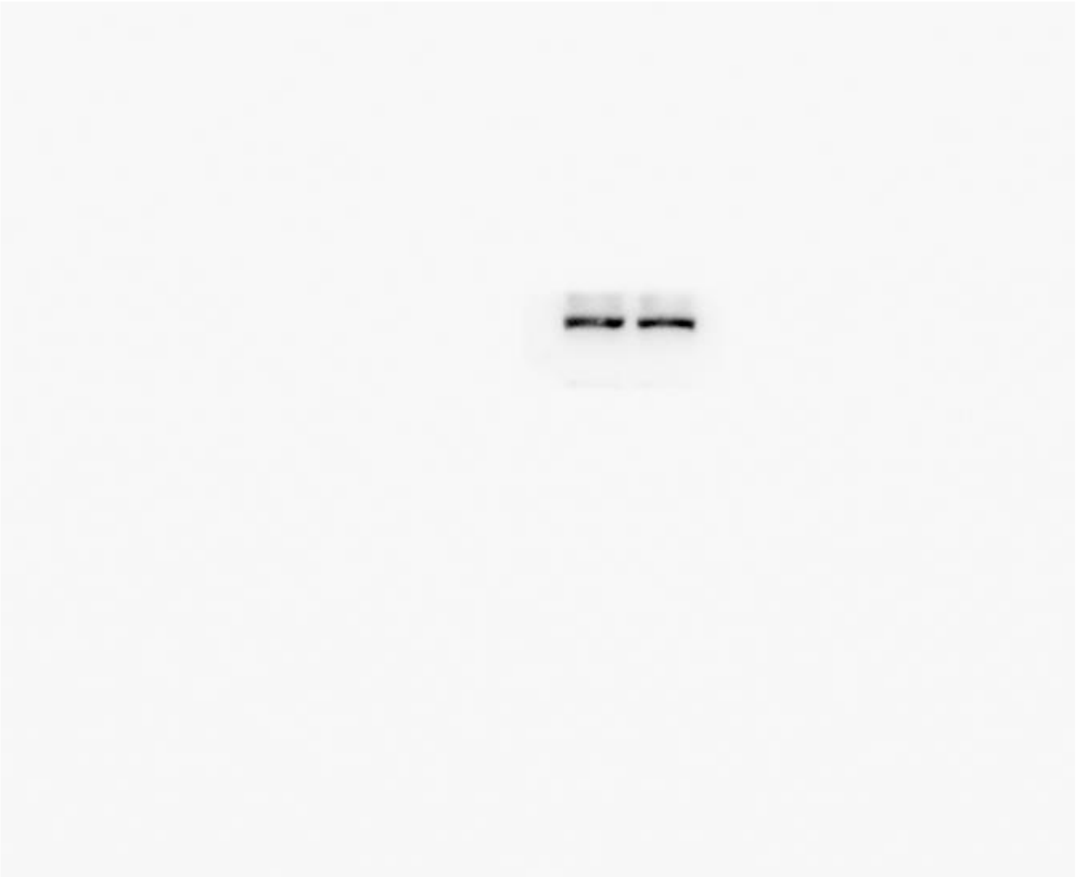

Fig 6A (LS174T cells; EV, MARCH5)

IB: CPT2

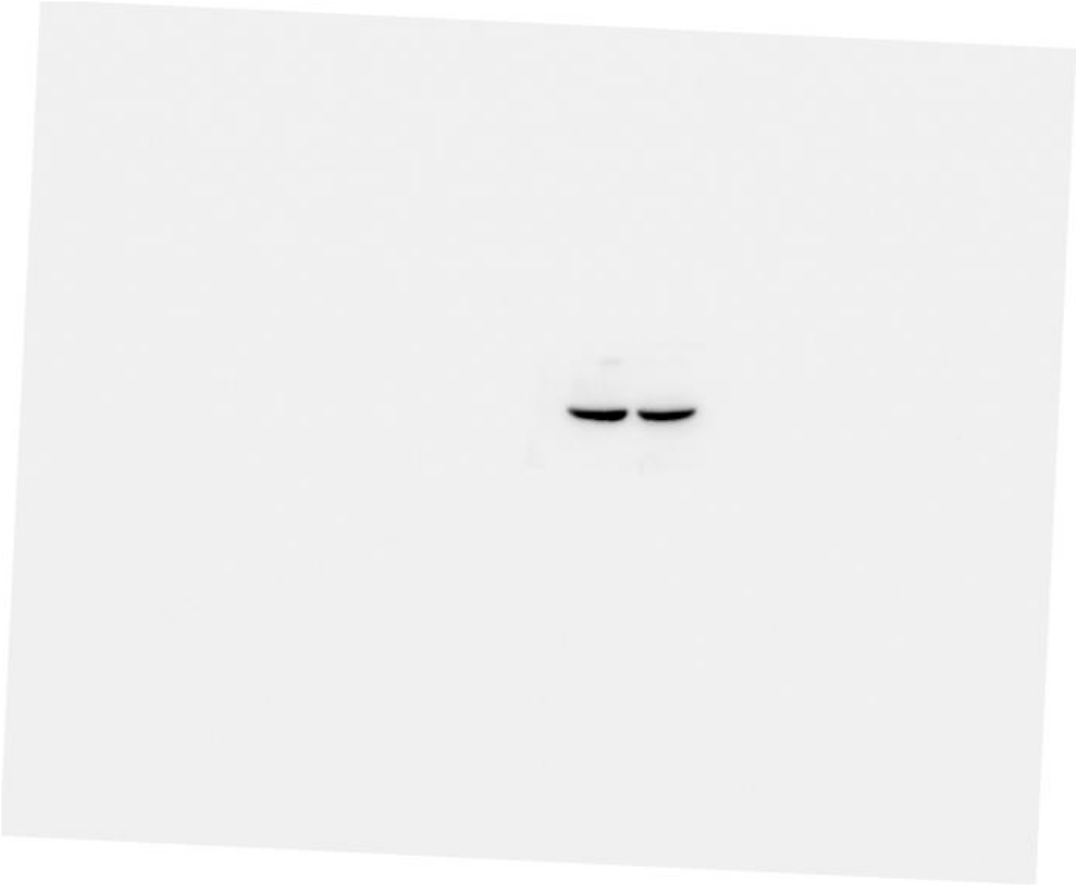

Fig 6A (LS174T cells; EV, MARCH5)

IB: β-actin

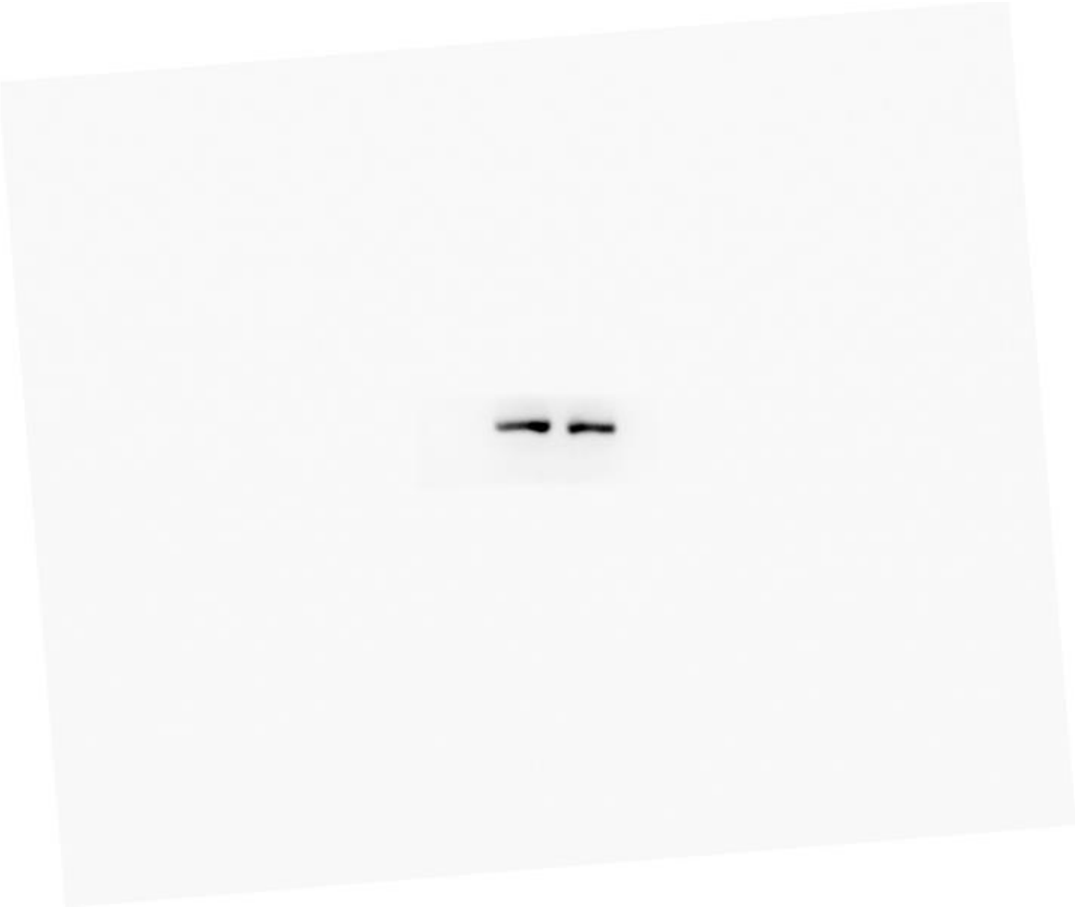

Fig 6A (LS174T cells; EV, RNF185)

IB: CPT2

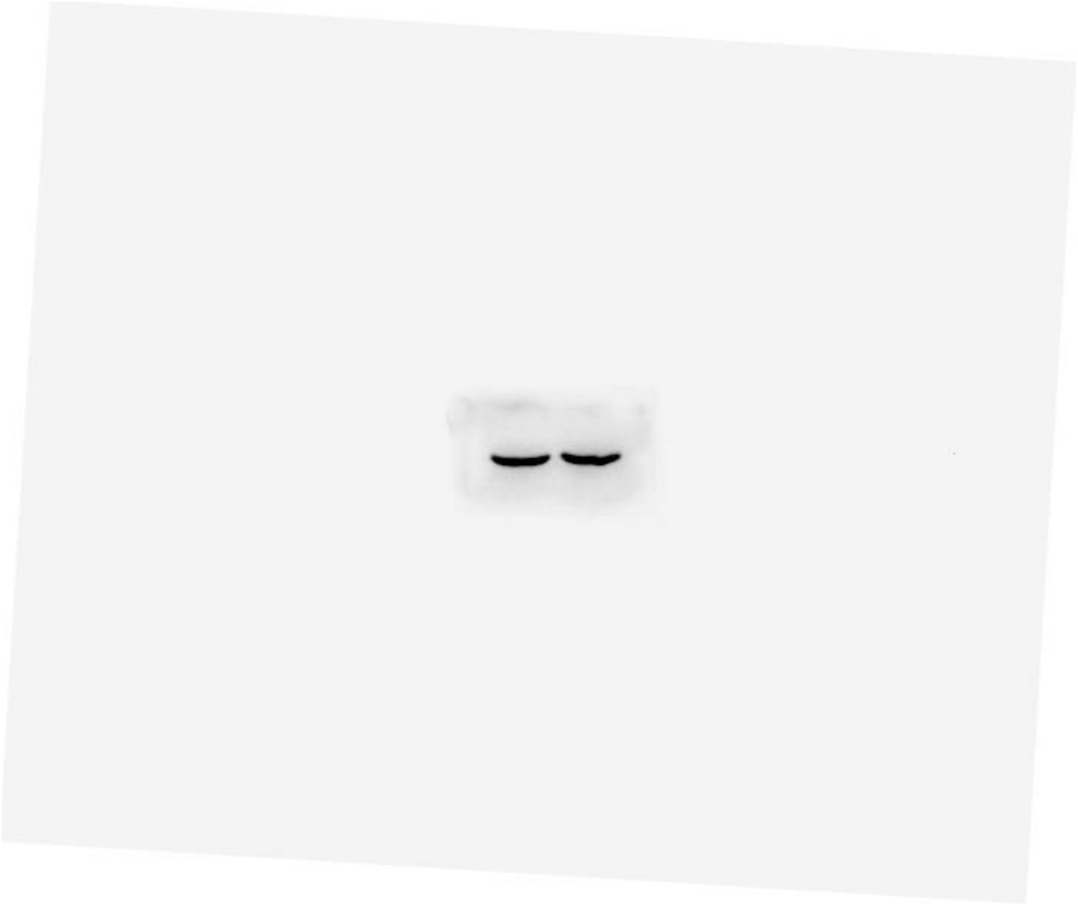

Fig 6A (LS174T cells; EV, RNF185)

IB: β-actin

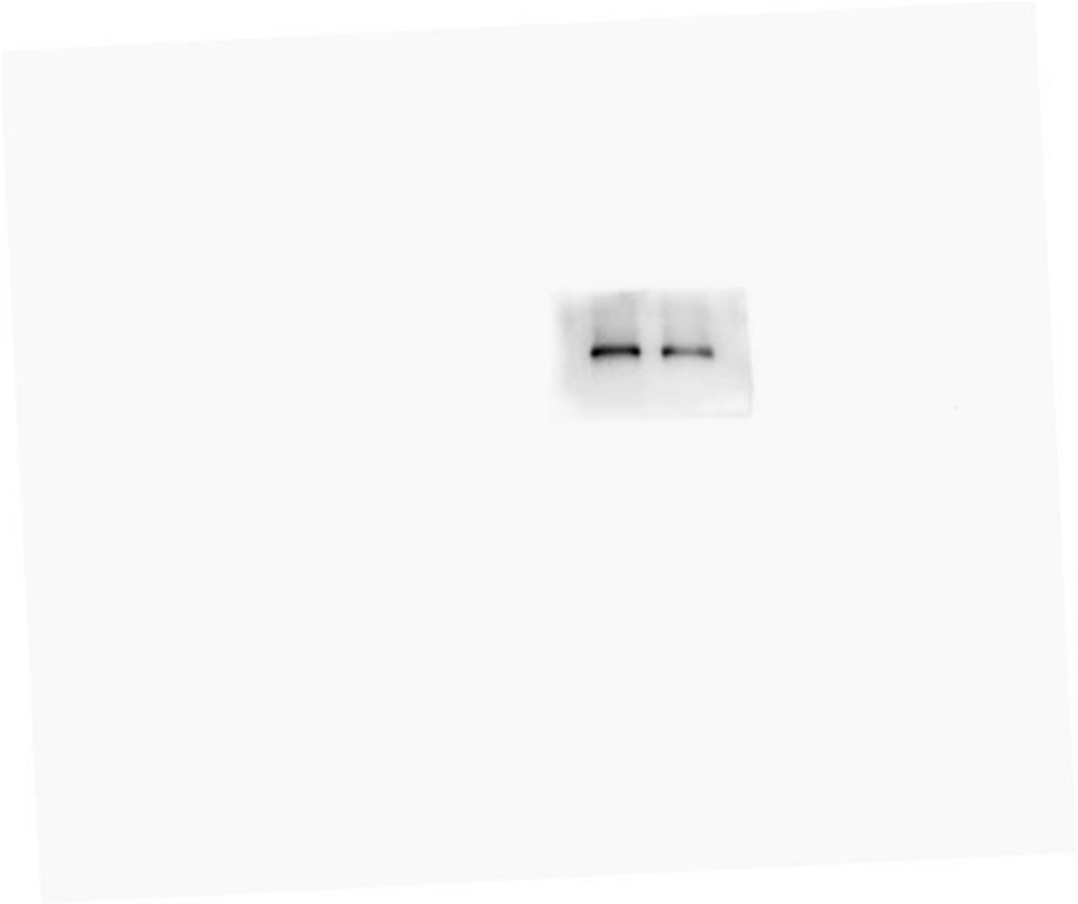

Fig 6A (LS174T cells; EV, MUL1)

IB: CPT2

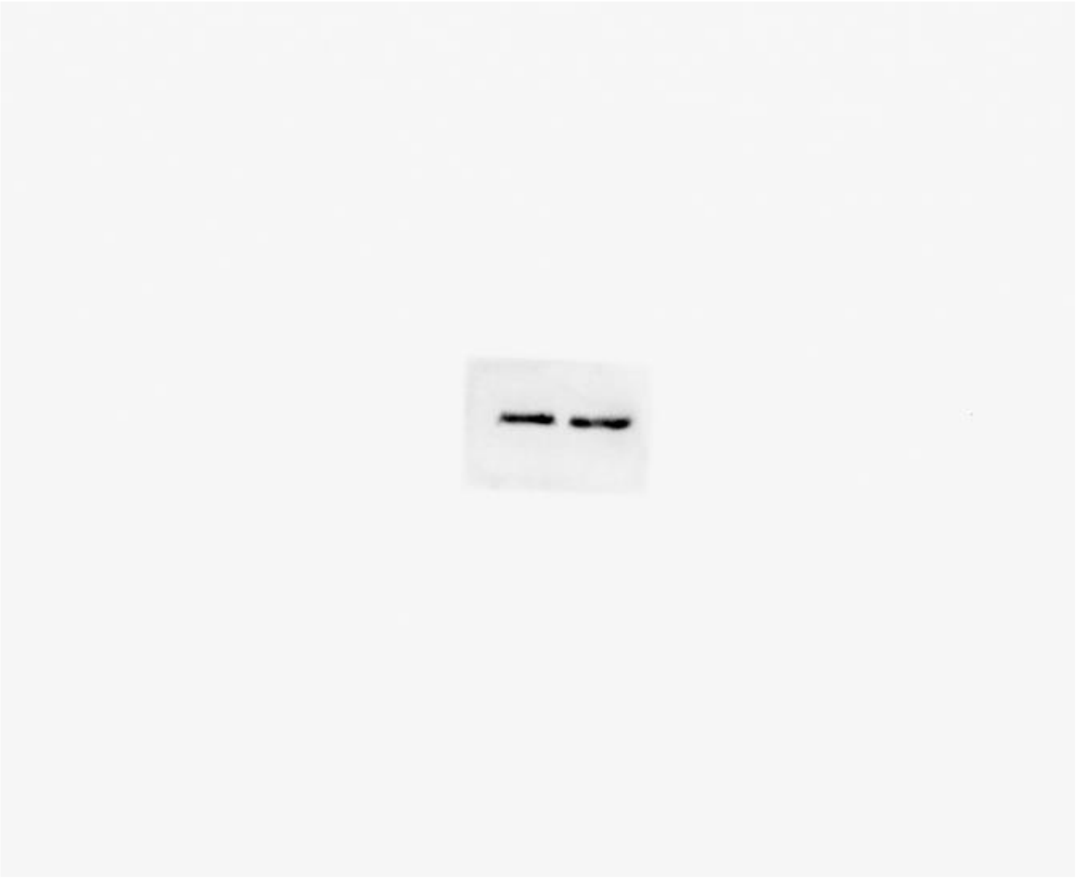

Fig 6A (LS174T cells; EV, MUL1)

IB: β-actin

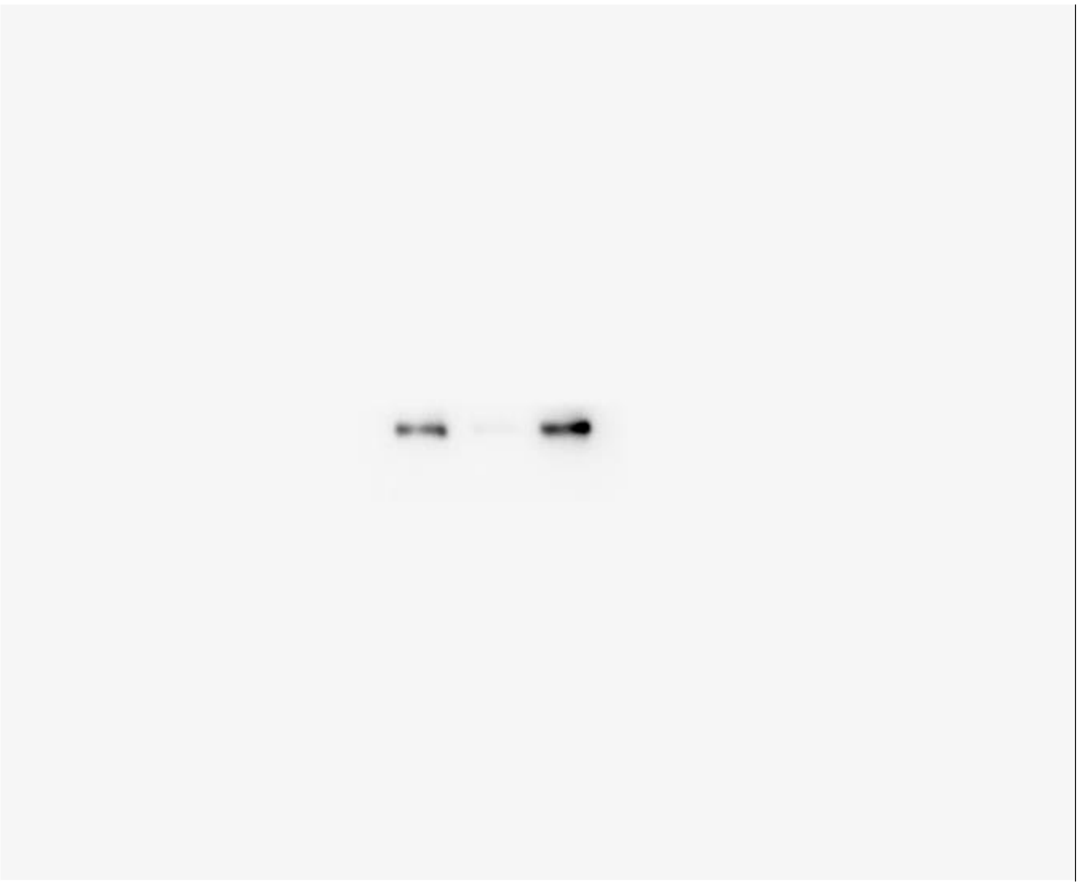

Fig 6B (HCT116 cells; IP: CPT2)

IB: CPT2

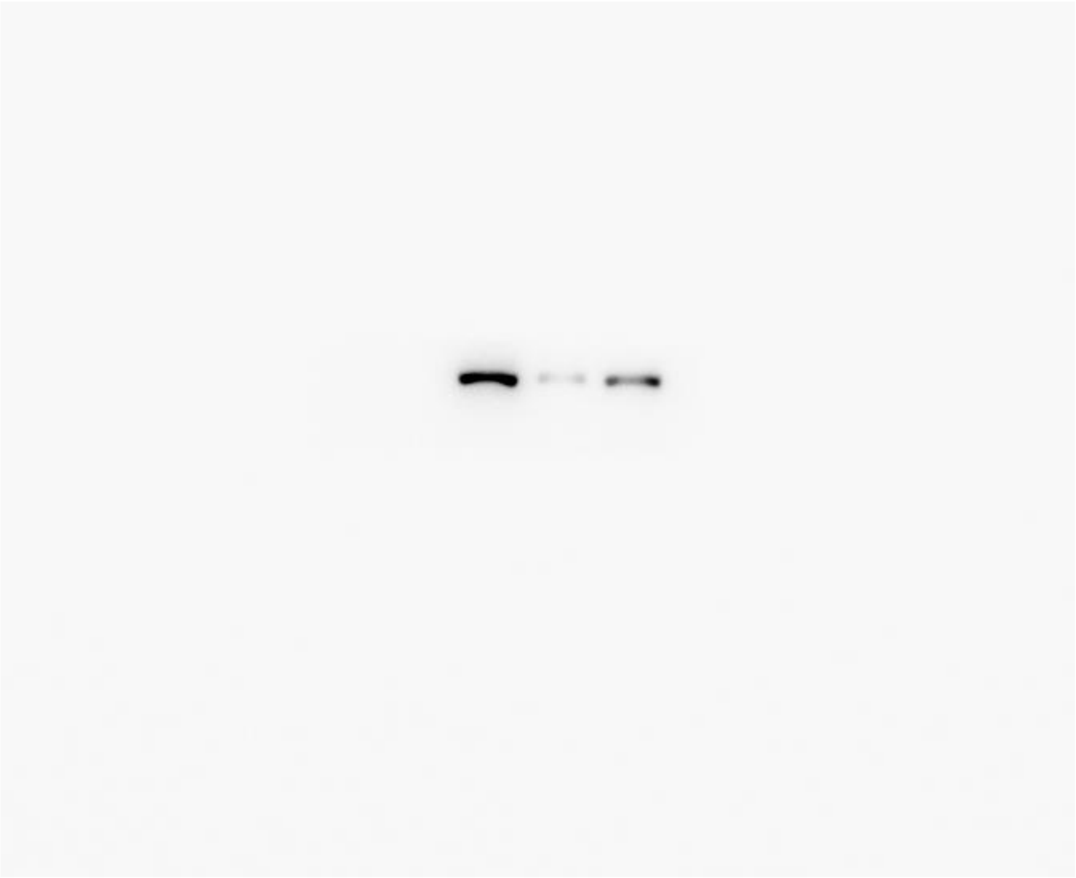

Fig 6B (HCT116 cells; IP: CPT2)

IB: MUL1

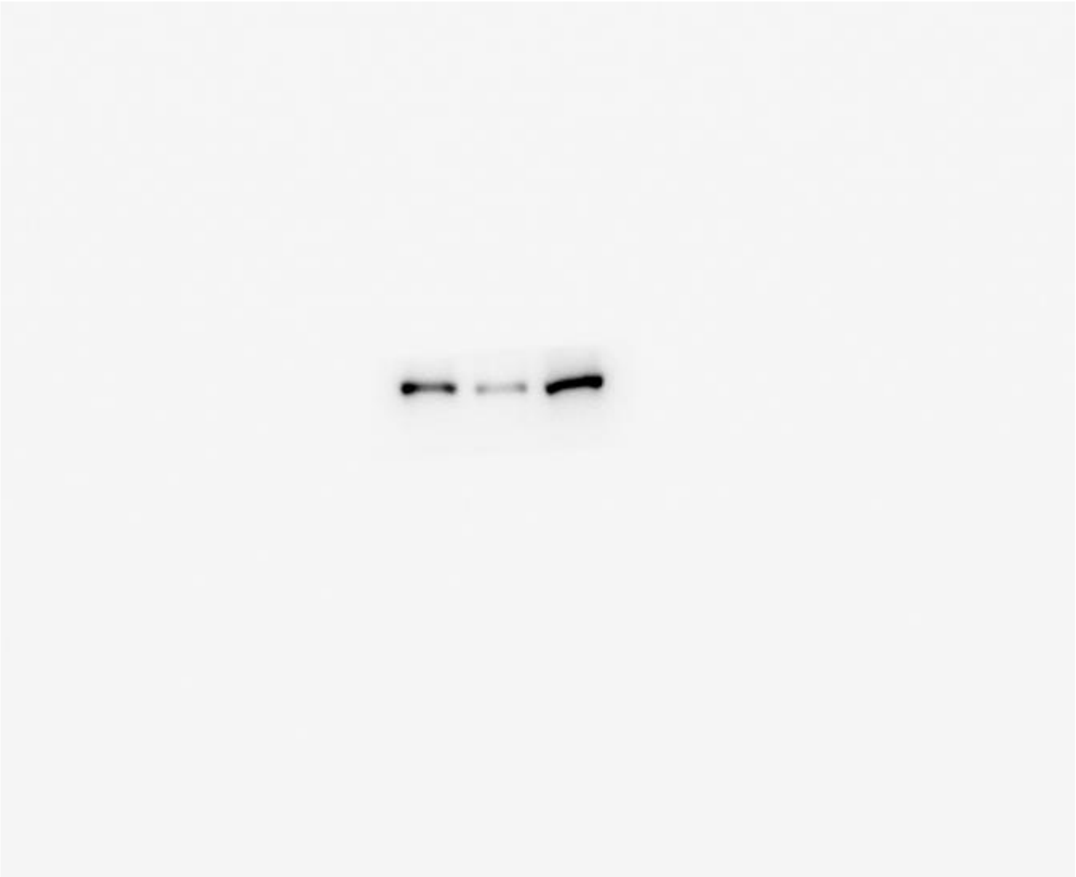

Fig 6B (HCT116 cells; IP: MUL1)

IB: MUL1

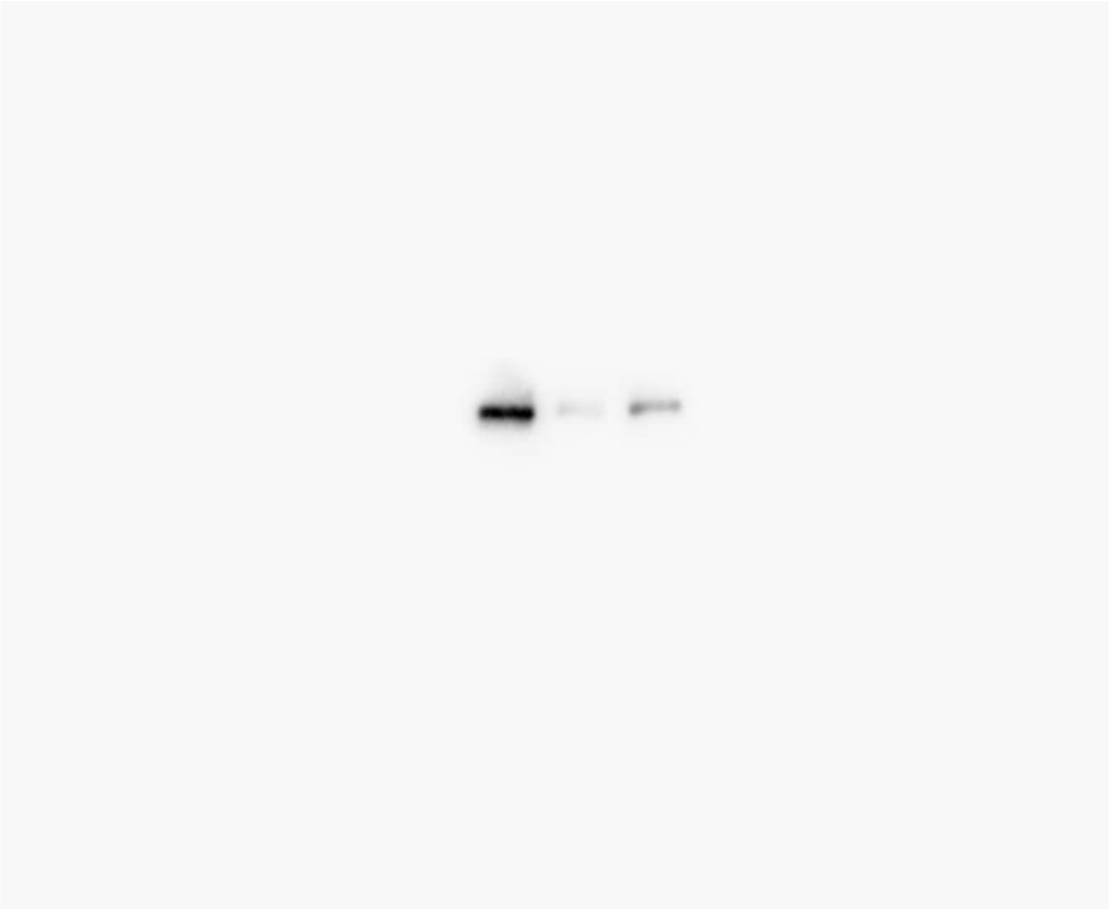

Fig 6B (HCT116 cells; IP: MUL1)

IB: CPT2

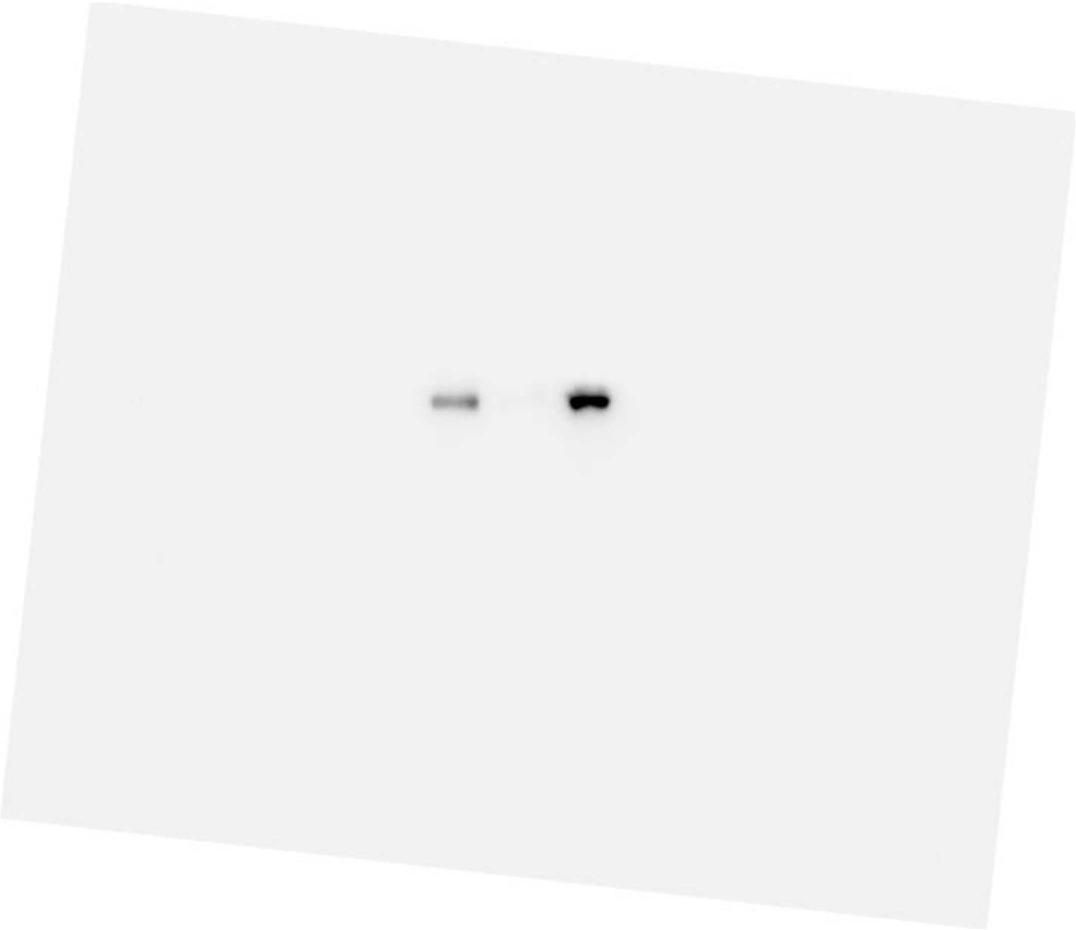

Fig 6B (LS174T cells; IP: CPT2)

IB: CPT2

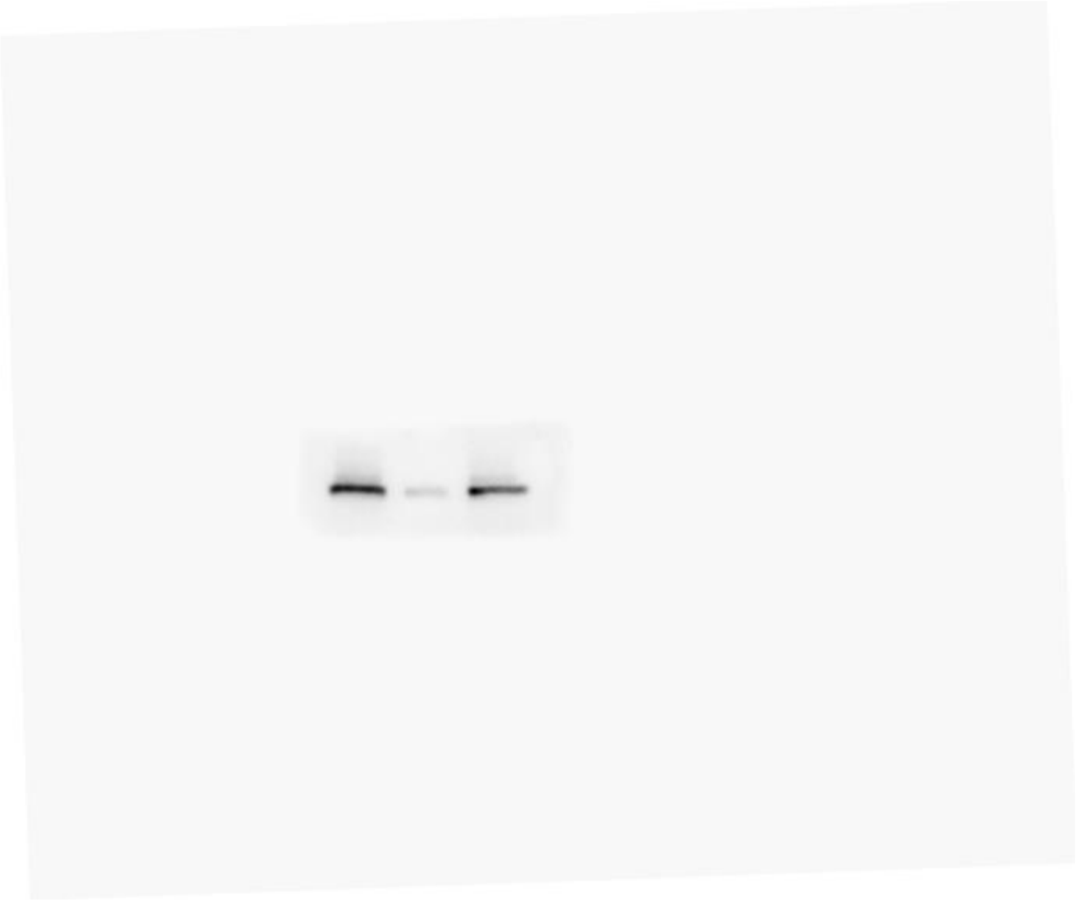

Fig 6B (LS174T cells; IP: CPT2)

IB: MUL1

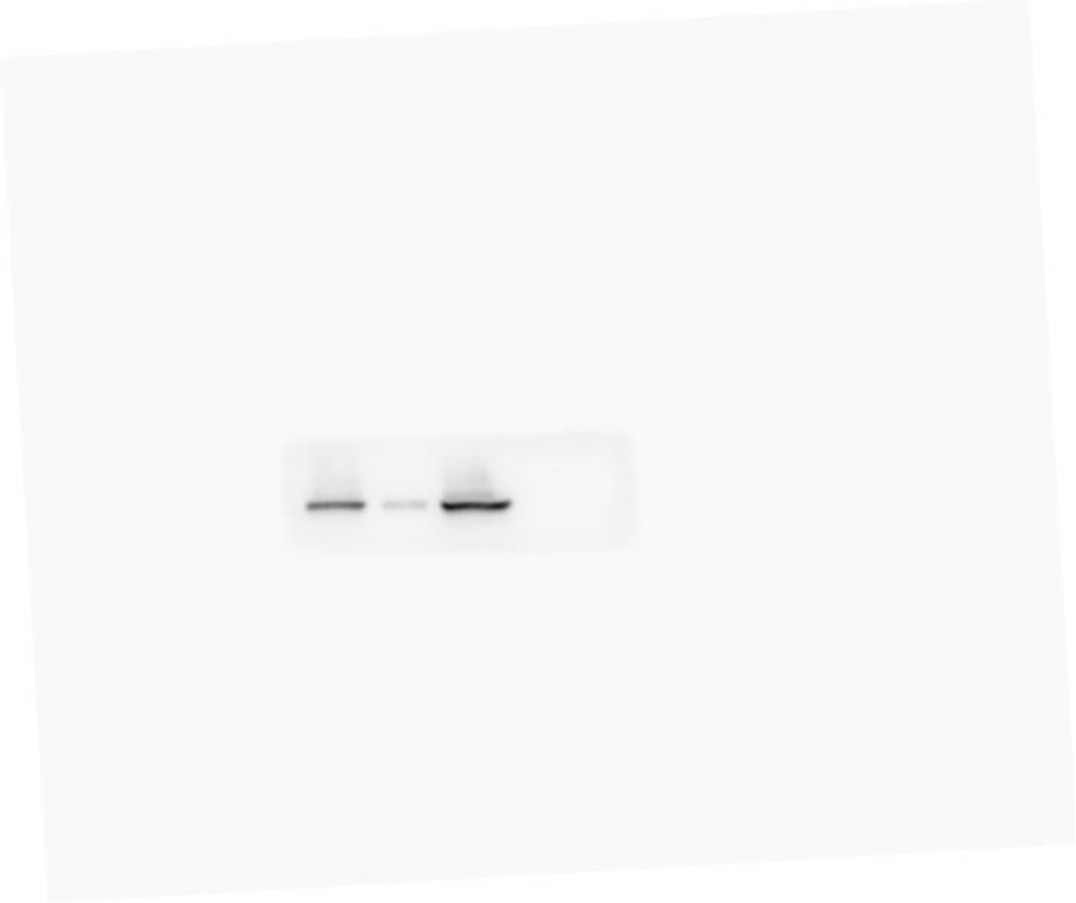

Fig 6B (LS174T cells; IP: MUL1)

IB: MUL1

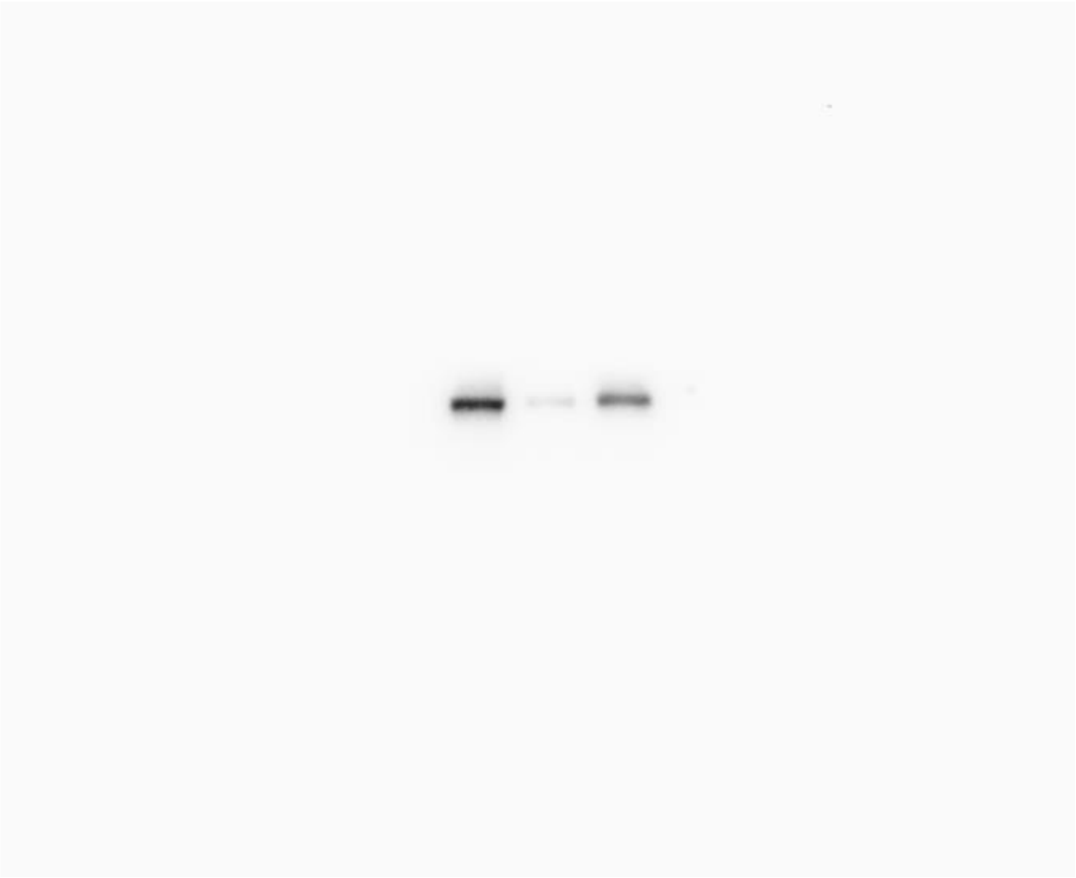

Fig 6B (LS174T cells; IP: MUL1)

IB: CPT2

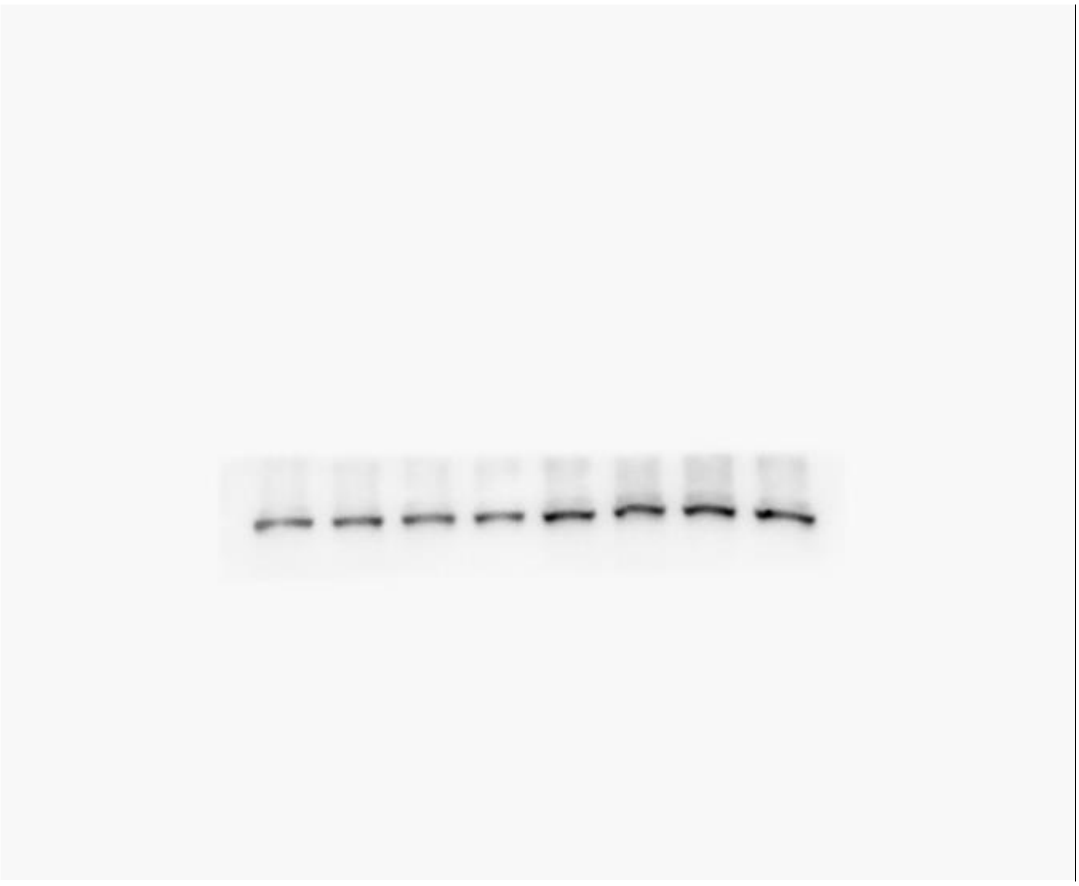

Fig 6D (HCT116 cells)

IB: MUL1

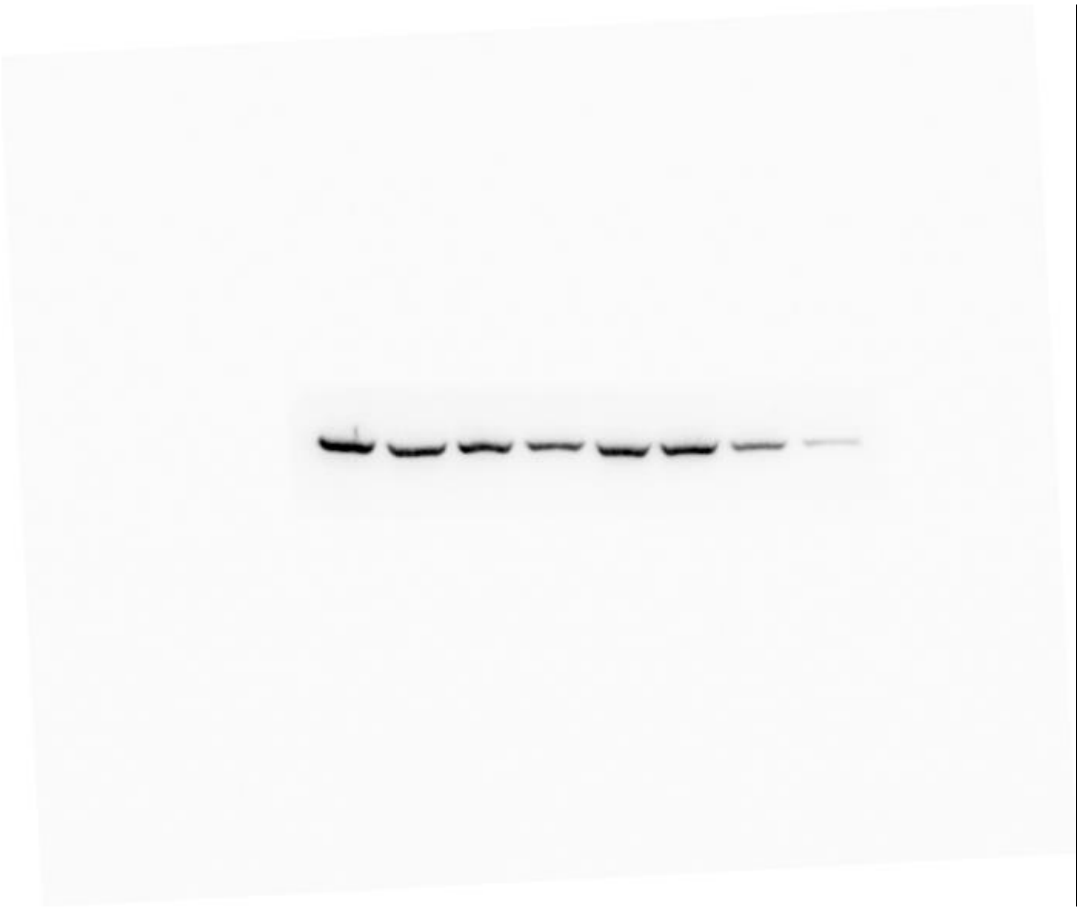

Fig 6D (HCT116 cells)

IB: CPT2

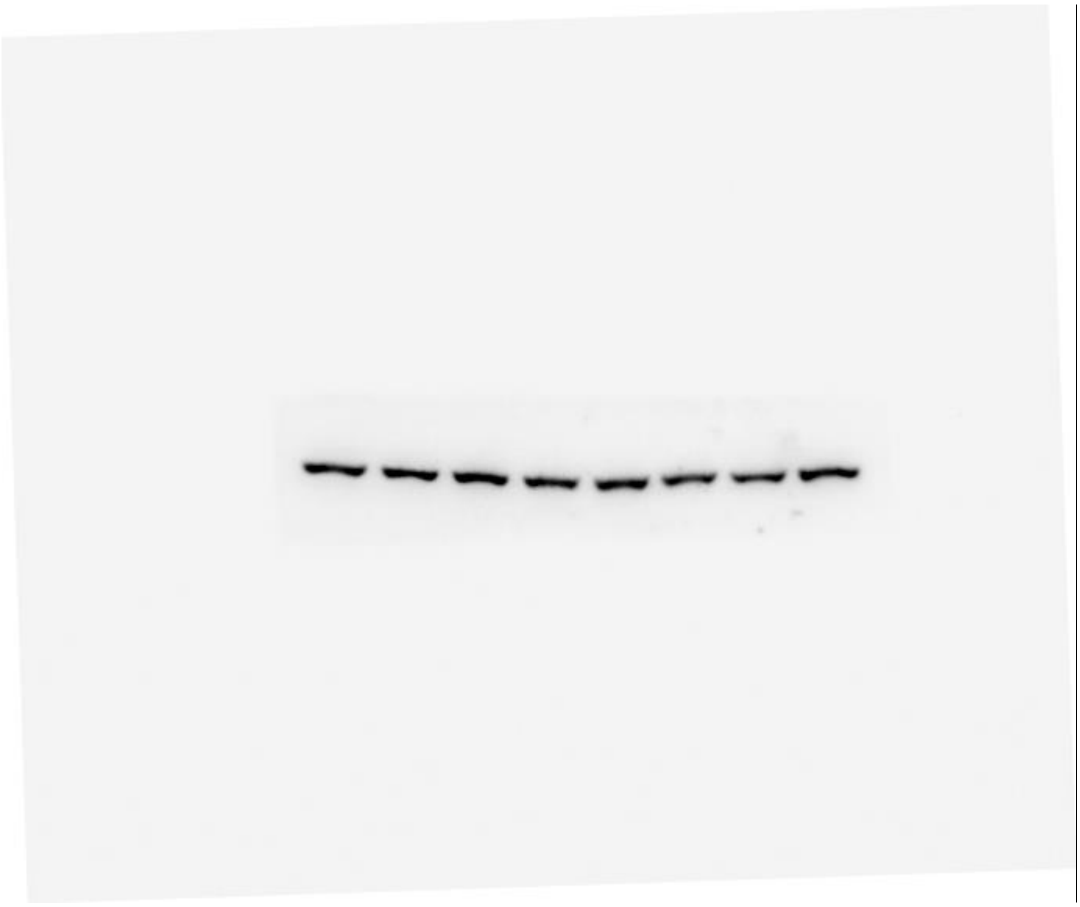

Fig 6D (HCT116 cells)

IB: β-actin

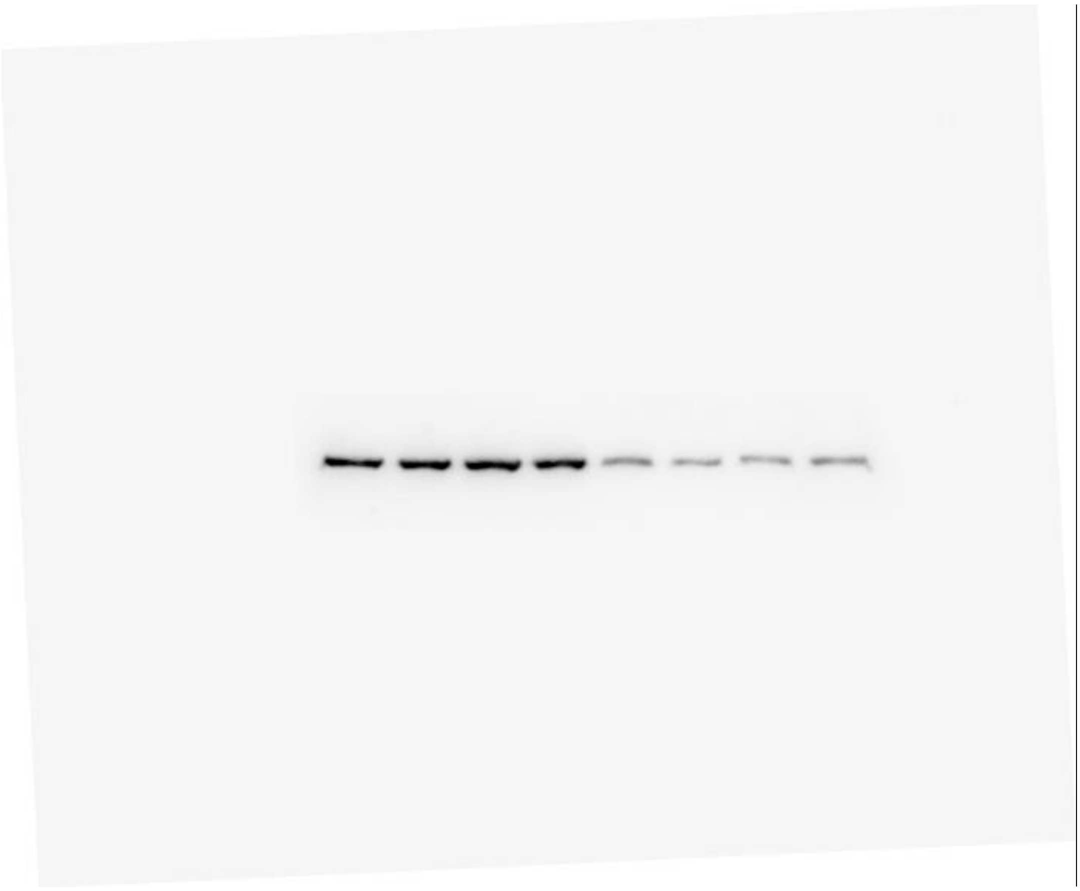

Fig 6D (LS174T cells)

IB: MUL1

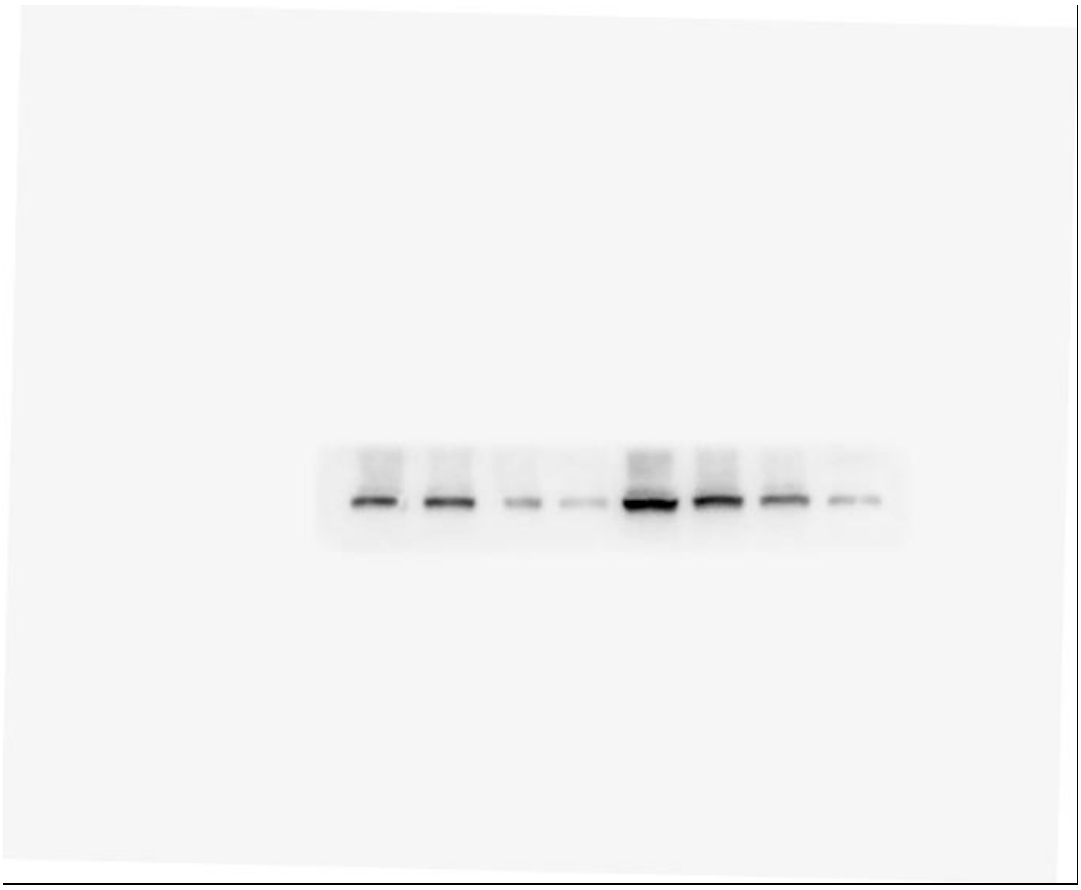

Fig 6D (LS174T cells)

IB:CPT2

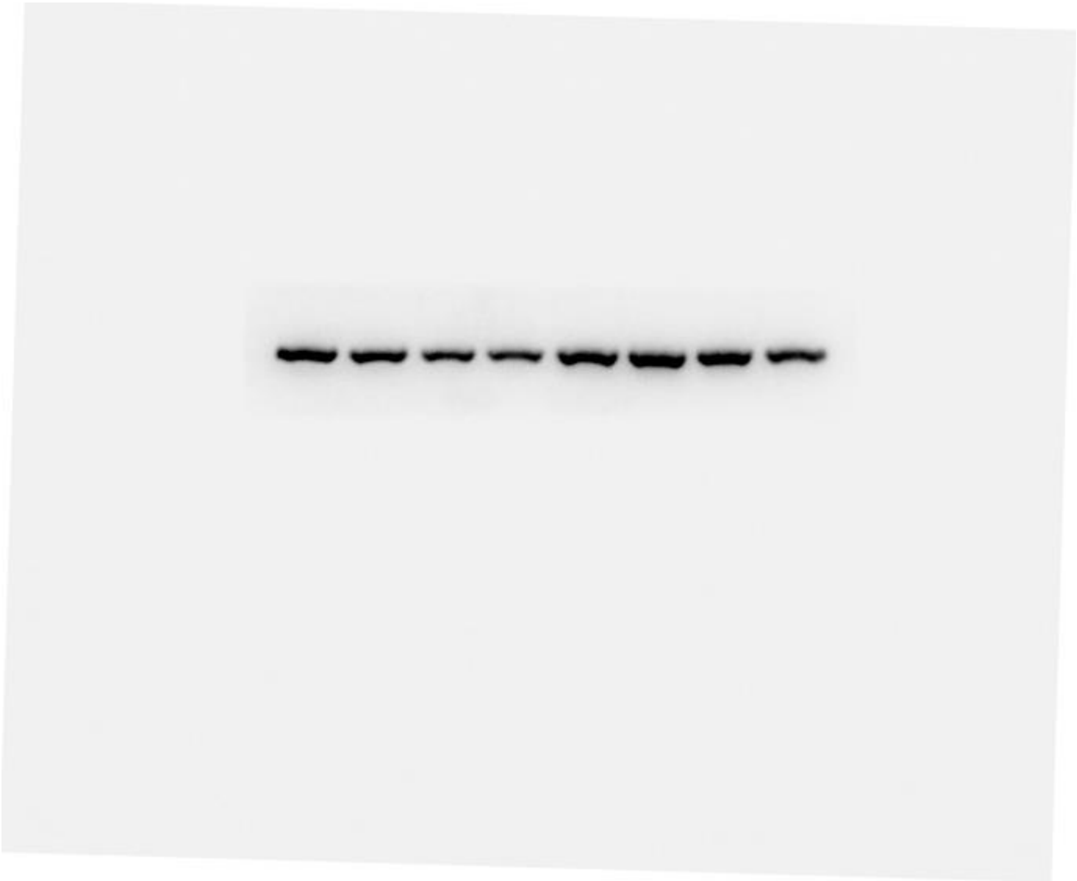

Fig 6D (LS174T cells)

IB: β-actin

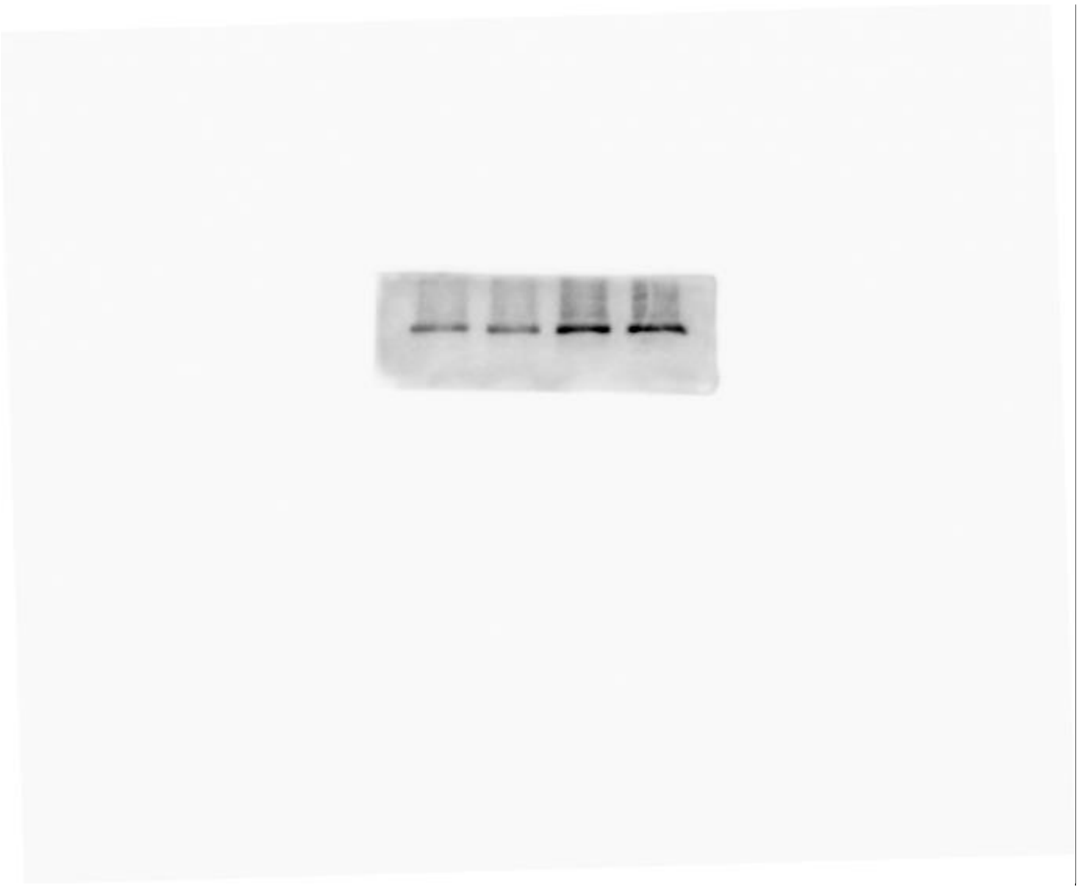

Fig 6E (HCT116 cells)

IB: MUL1

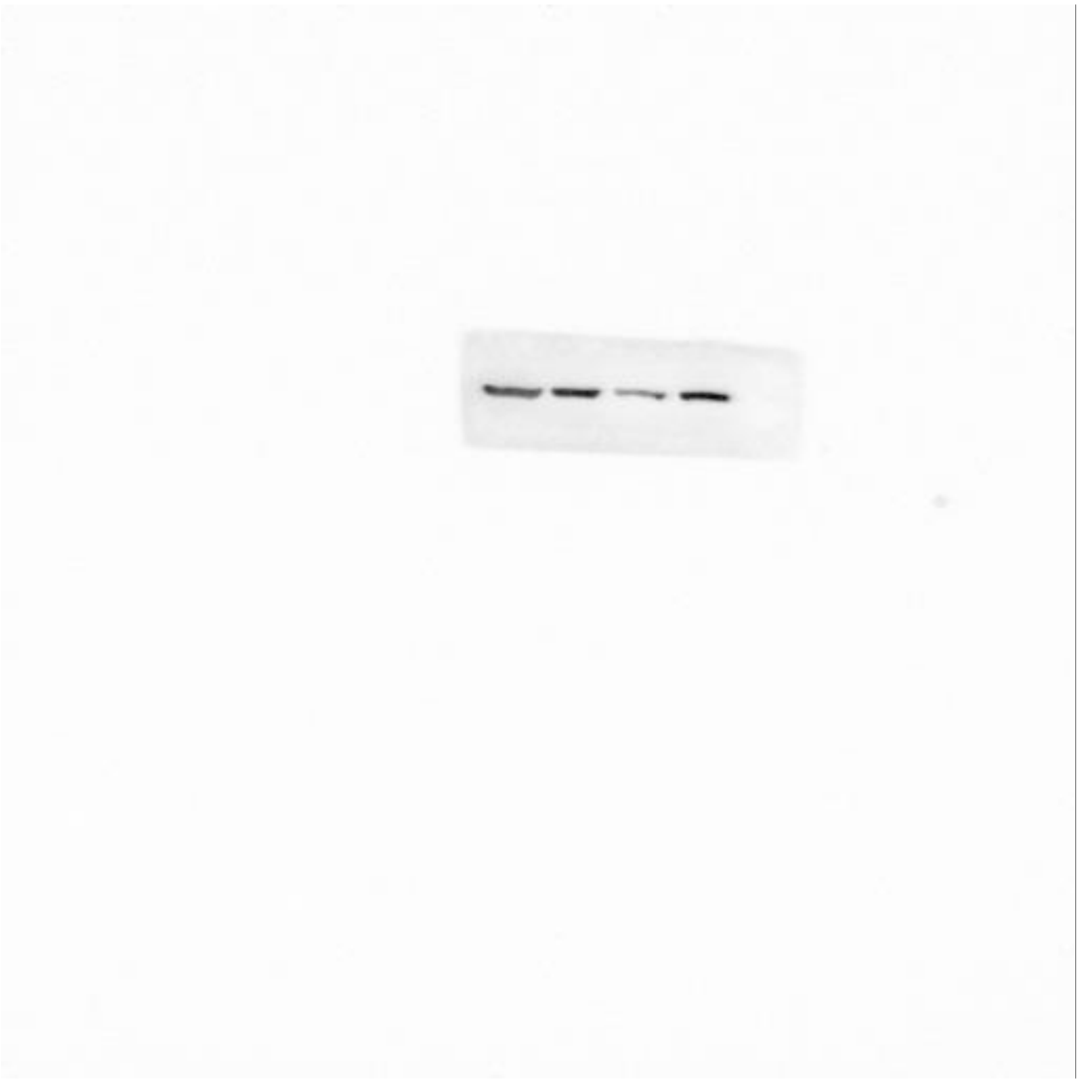

Fig 6E (HCT116 cells)

IB: CPT2

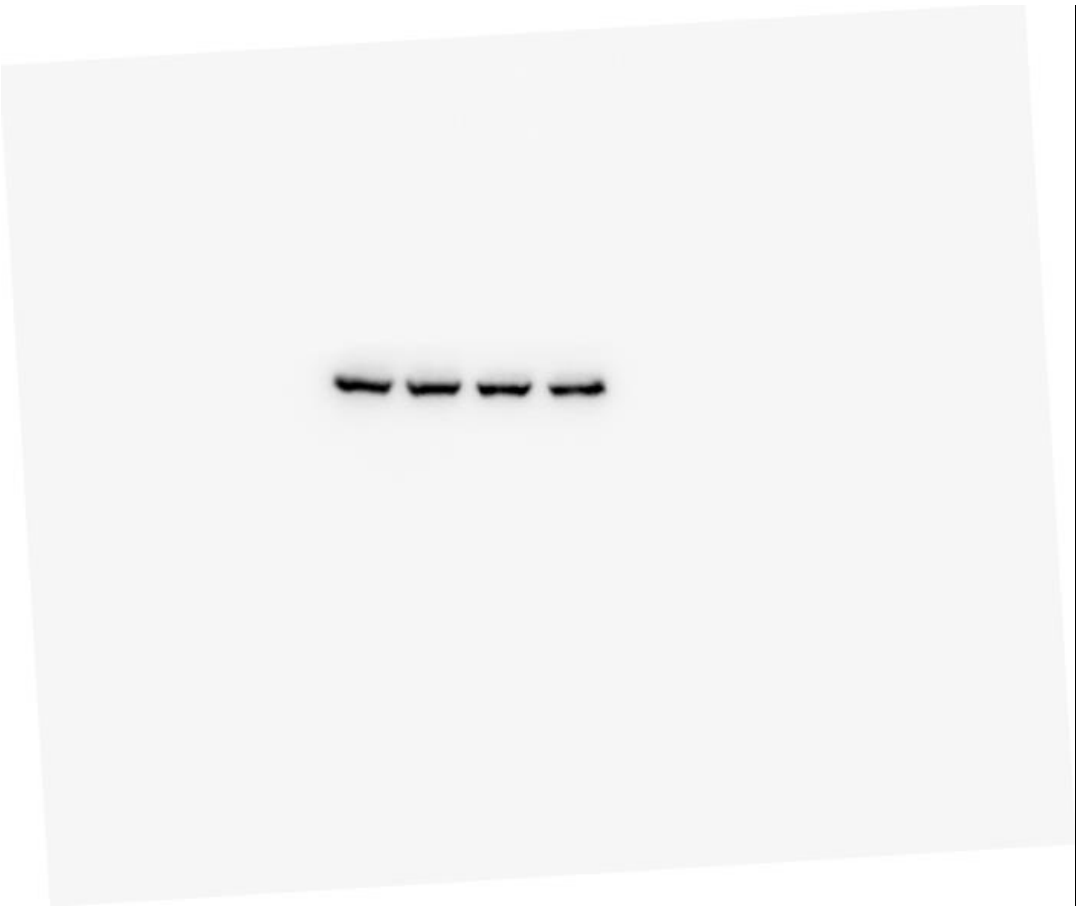

Fig 6E (HCT116 cells)

IB: β-actin

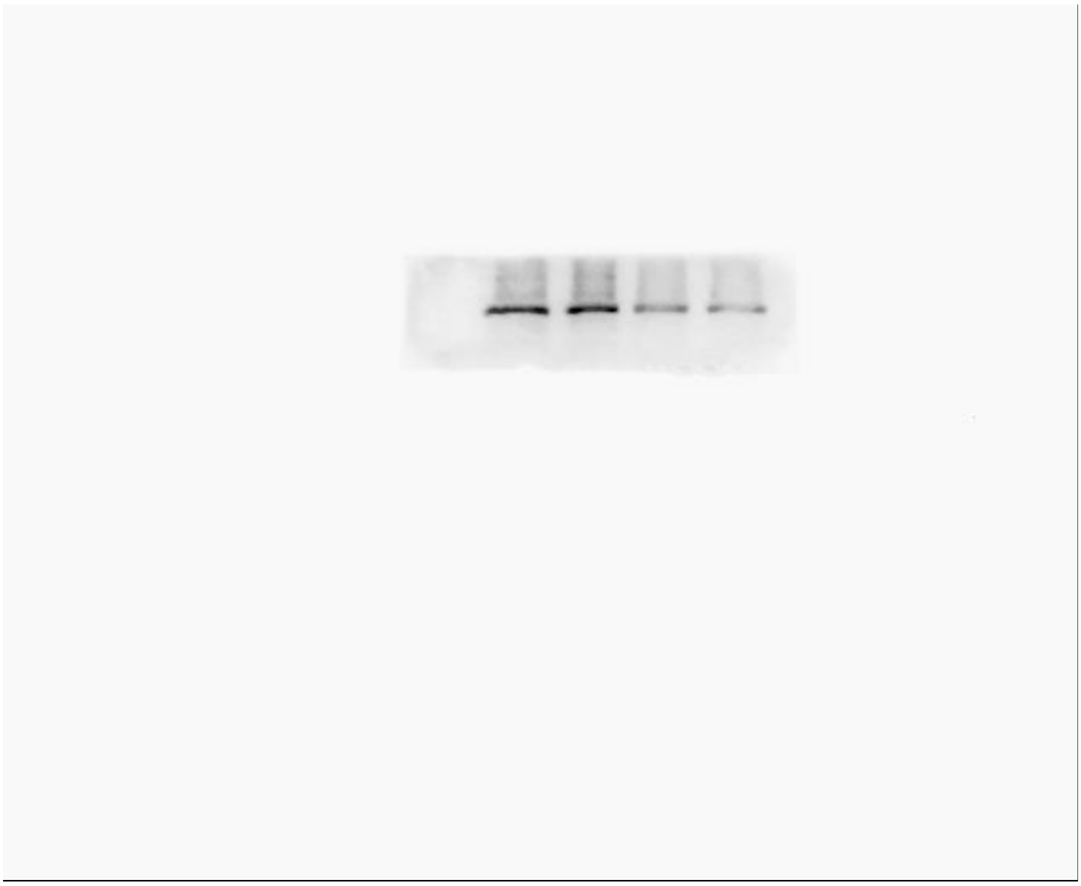

Fig 6E (LS174T cells)

IB: MUL1

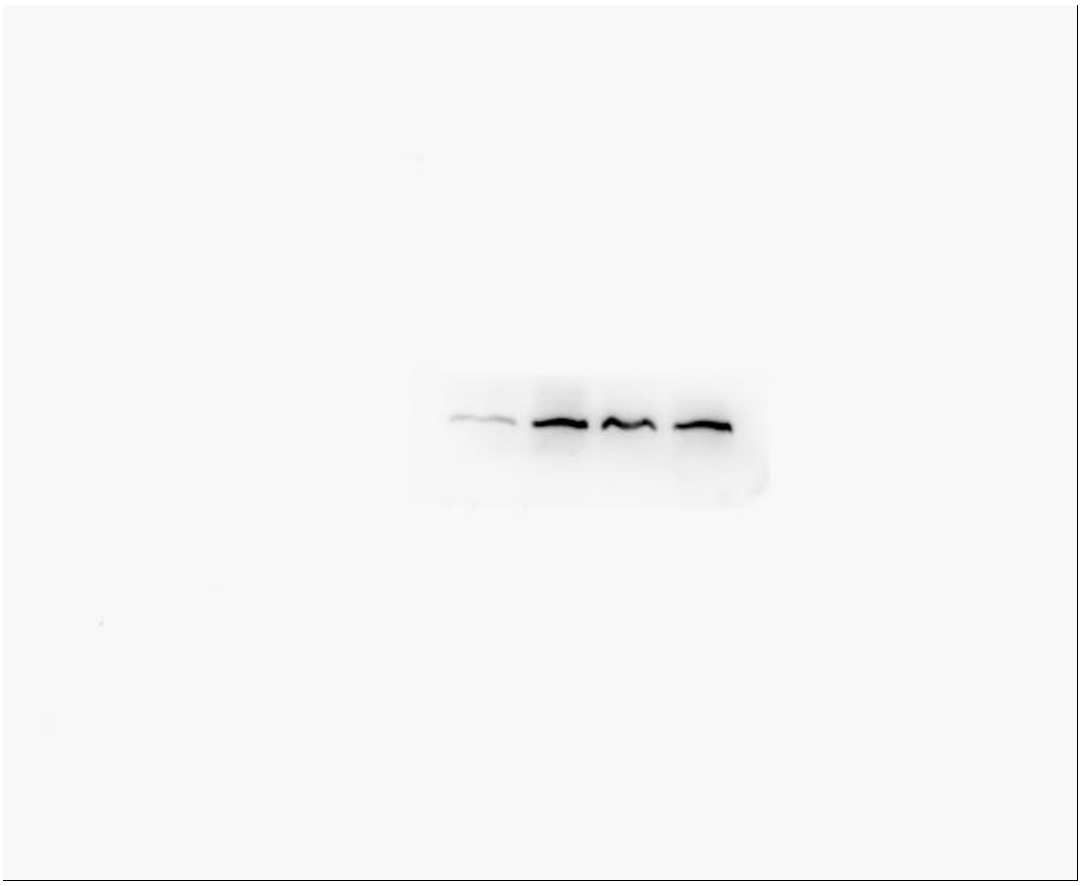

Fig 6E (LS174T cells)

IB: CPT2

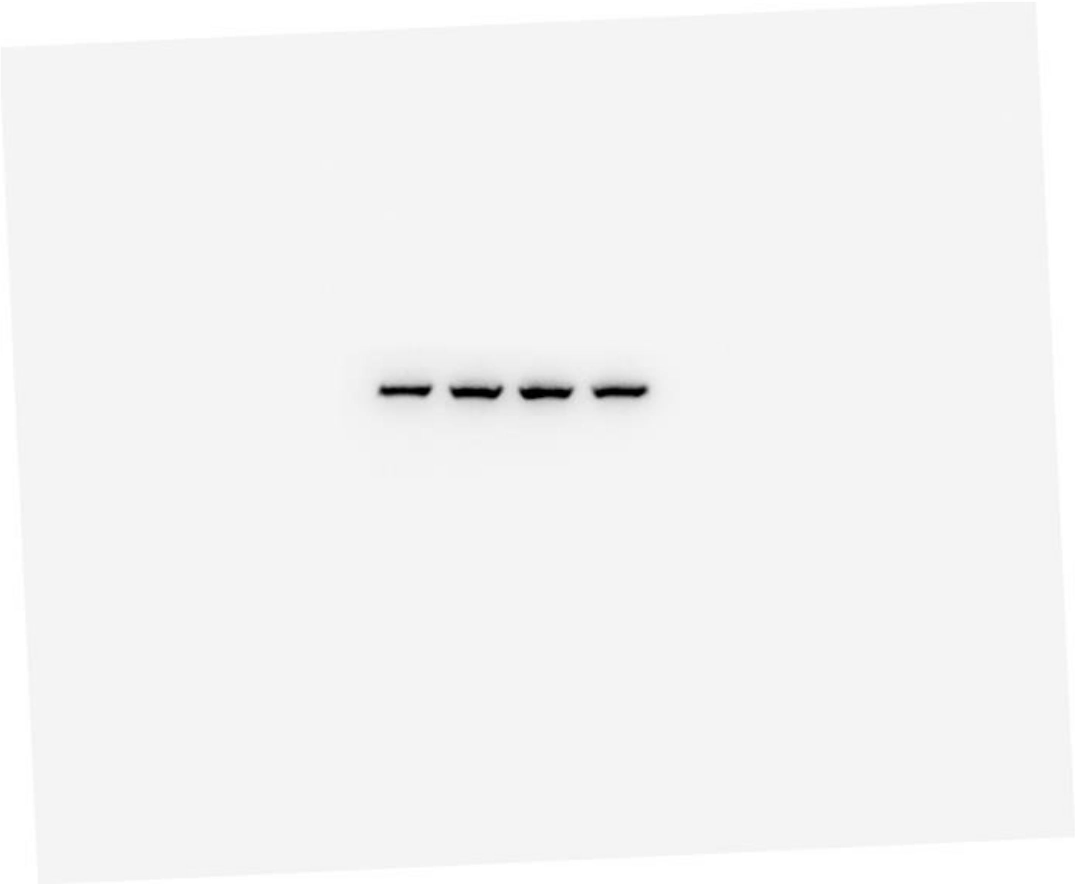

Fig 6E (LS174T cells)

IB: β-actin

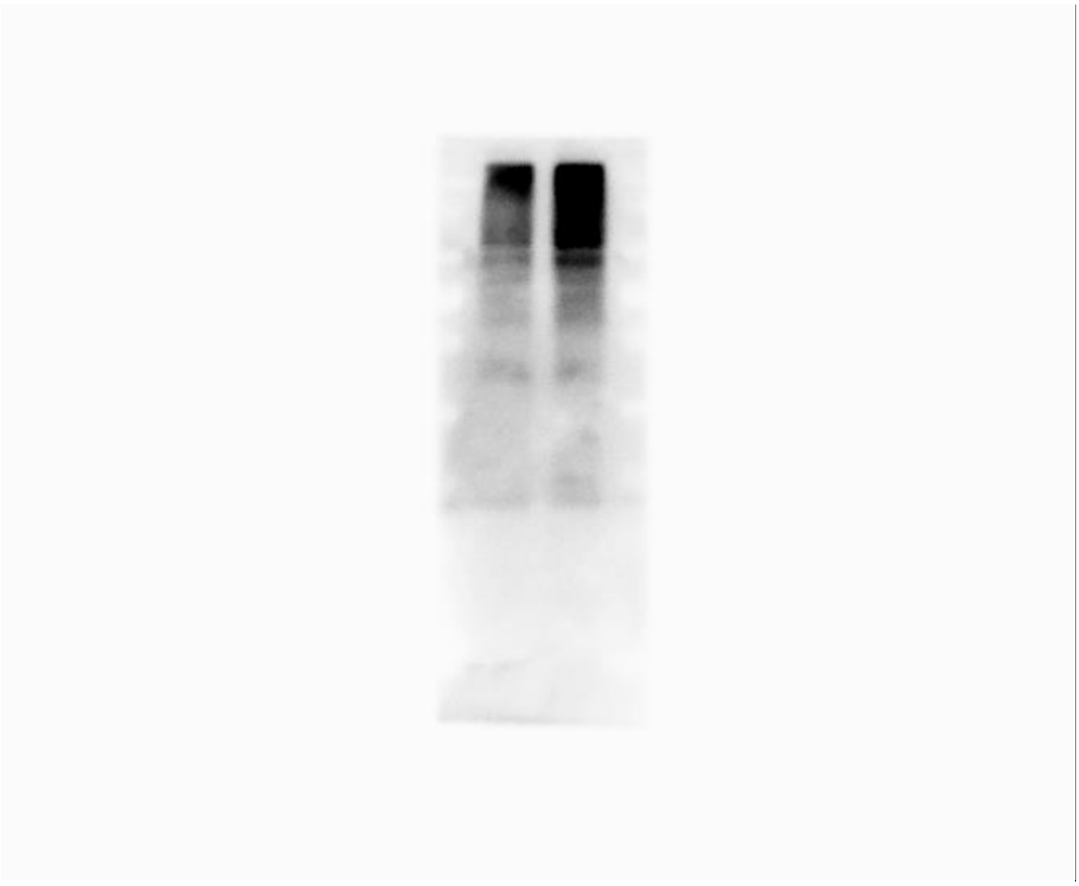

Fig 6F (HCT116 cells)

IB: UB

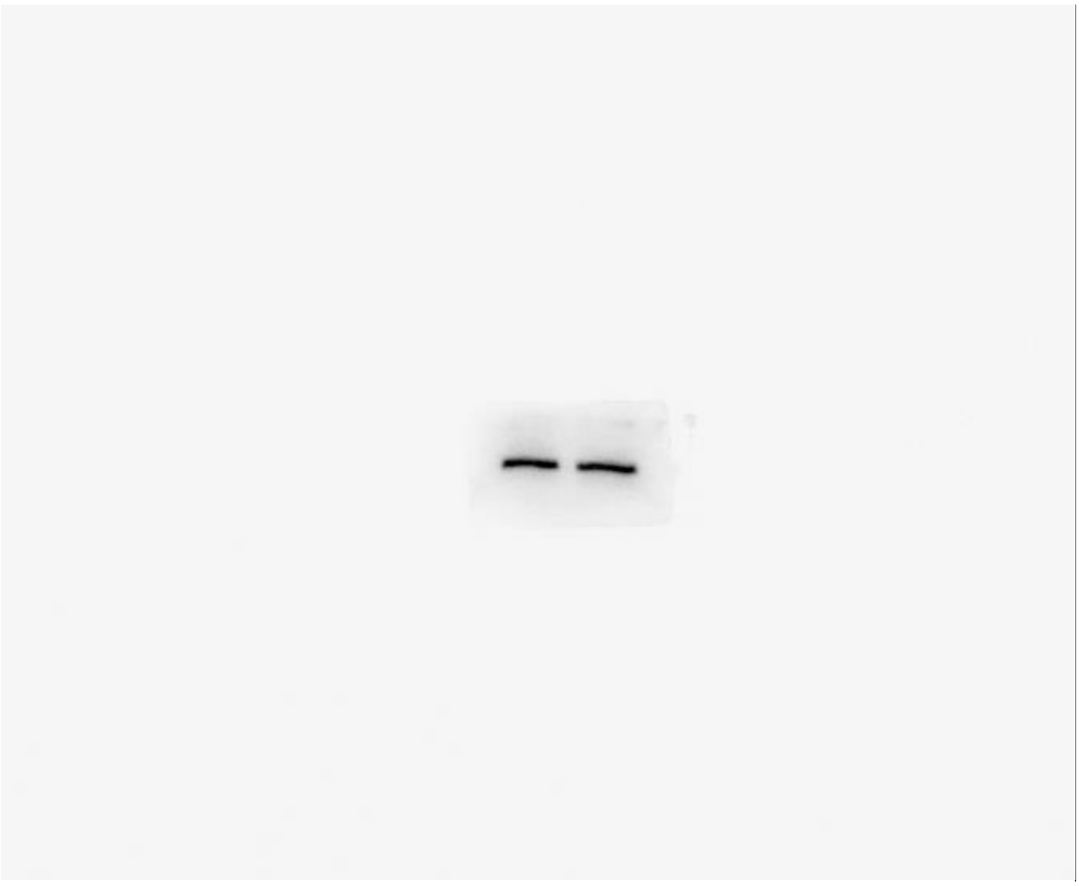

Fig 6F (HCT116 cells)

IB: CPT2

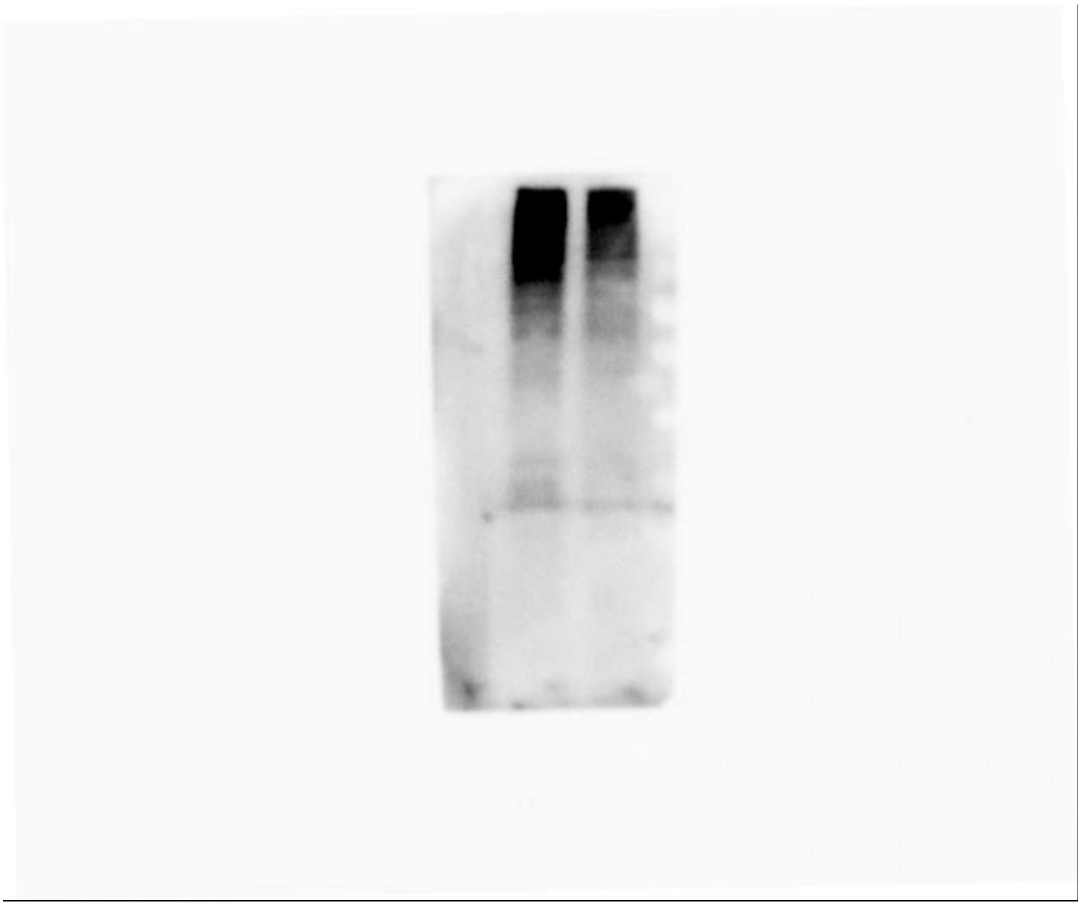

Fig 6F (LS174T cells)

IB: UB

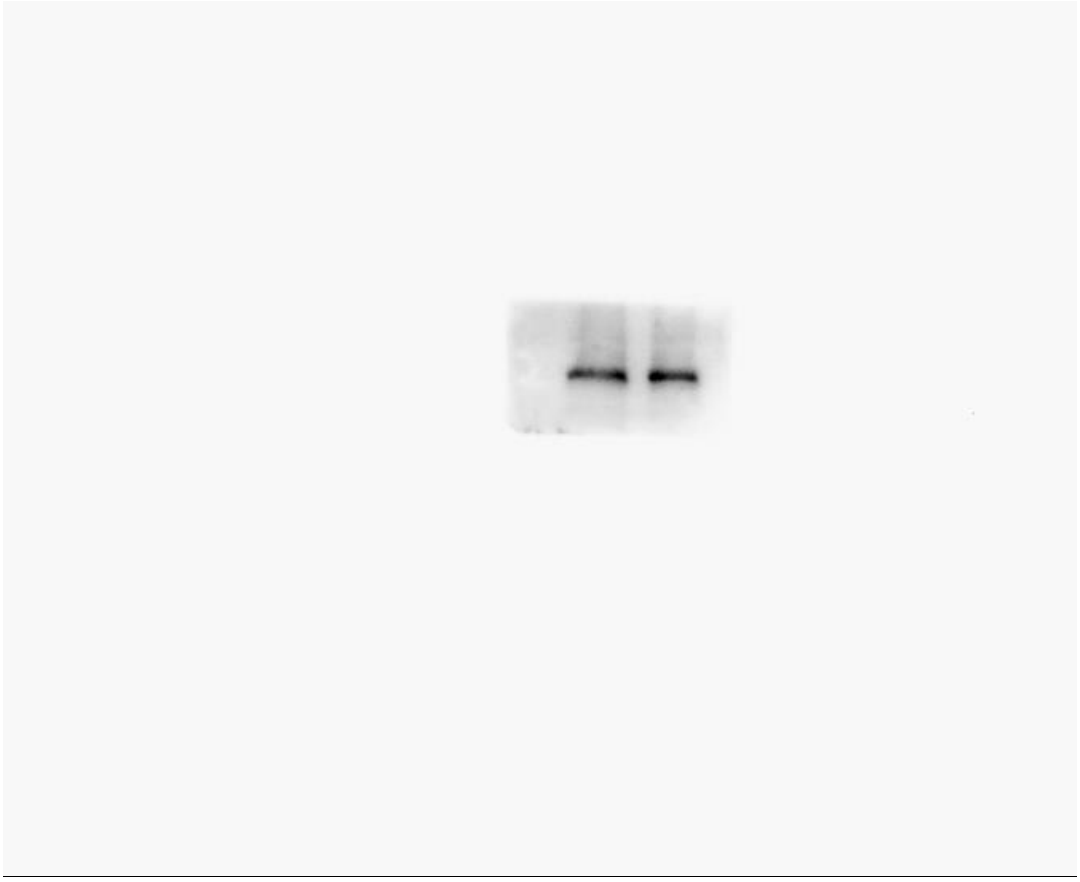

Fig 6F (LS174T cells)

IB: CPT2

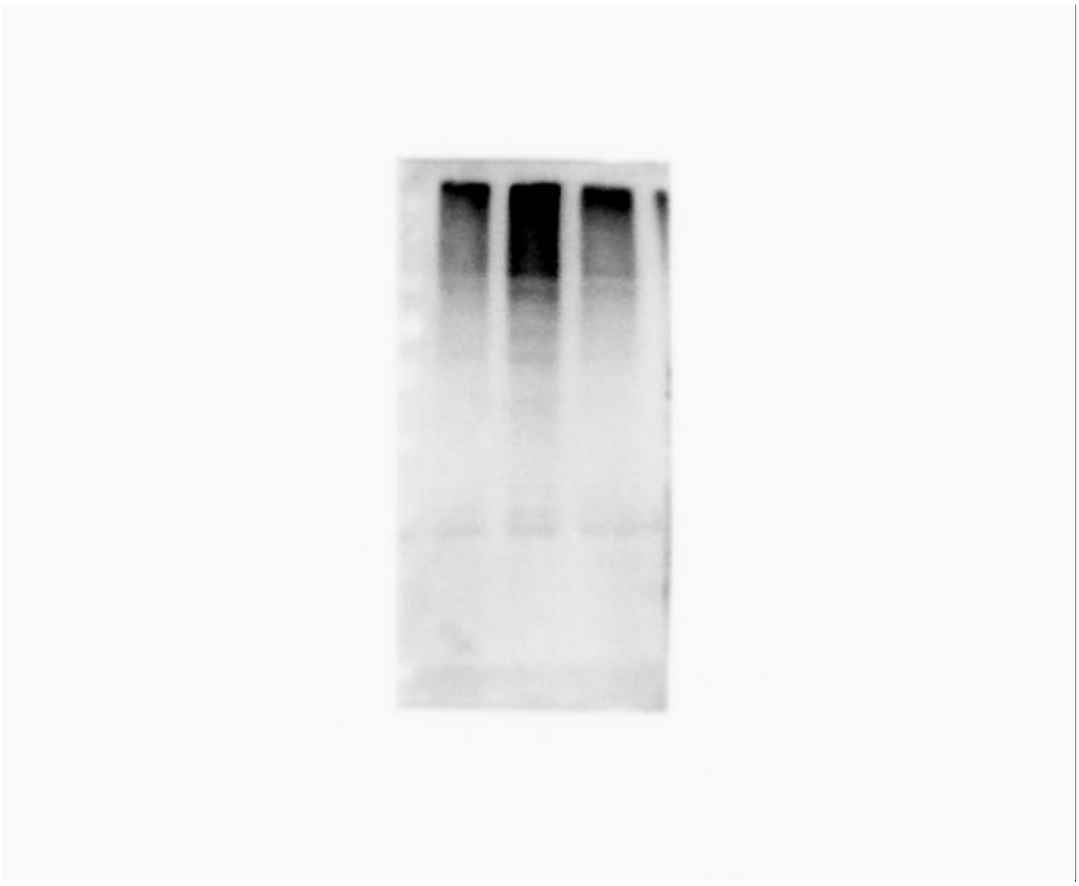

Fig 6G (HCT116 cells)

IB: UB

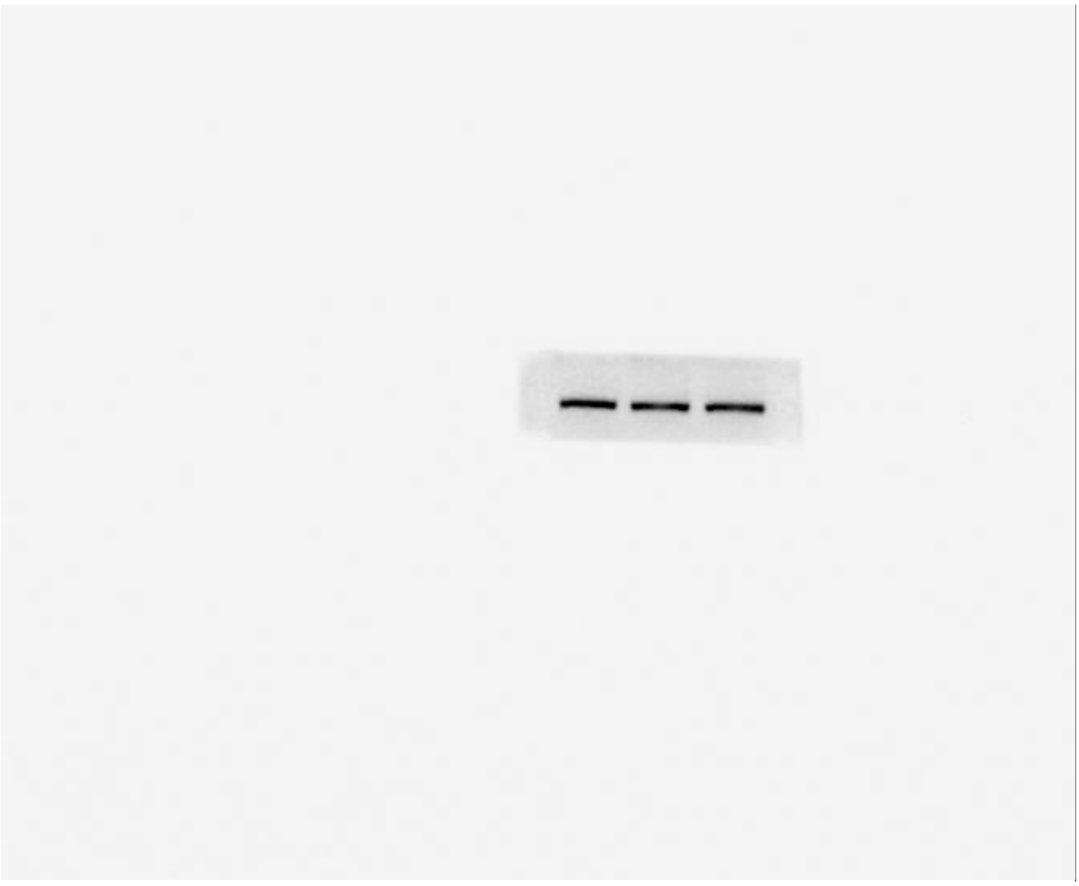

Fig 6G (HCT116 cells)

IB: CPT2

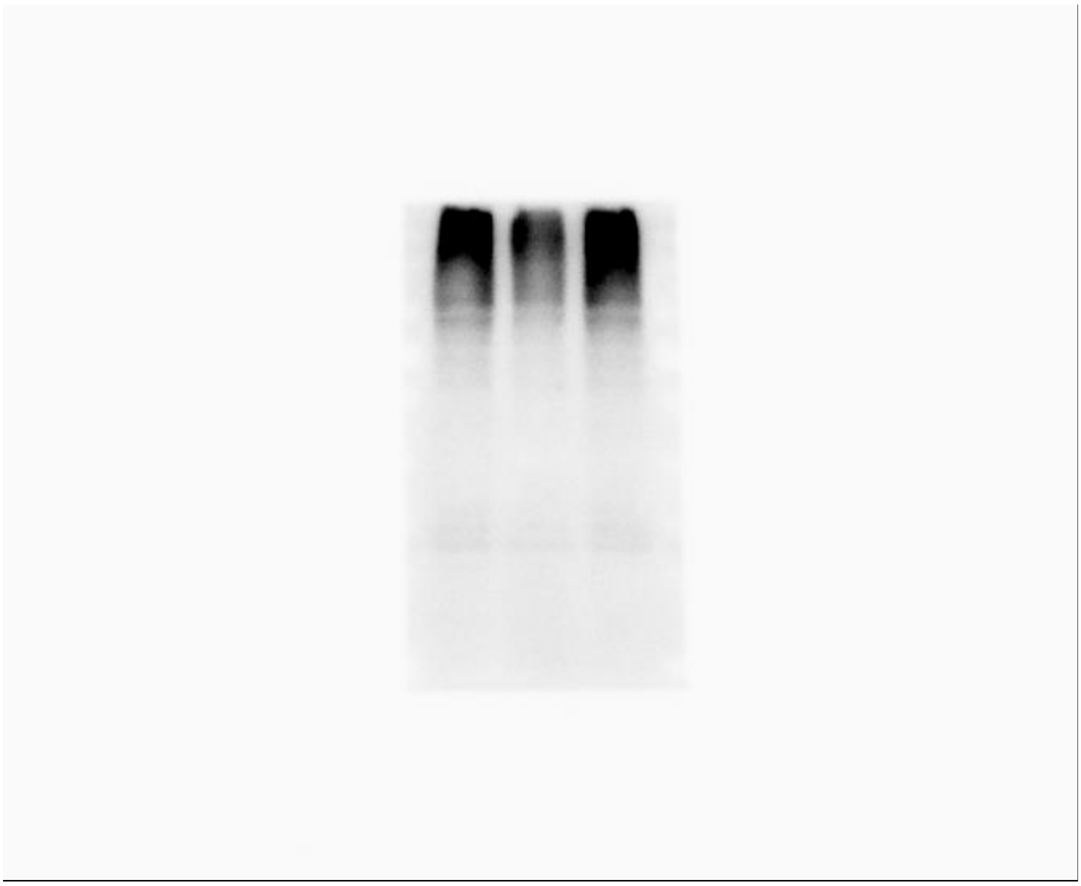

Fig 6G (LS174T cells)

IB: UB

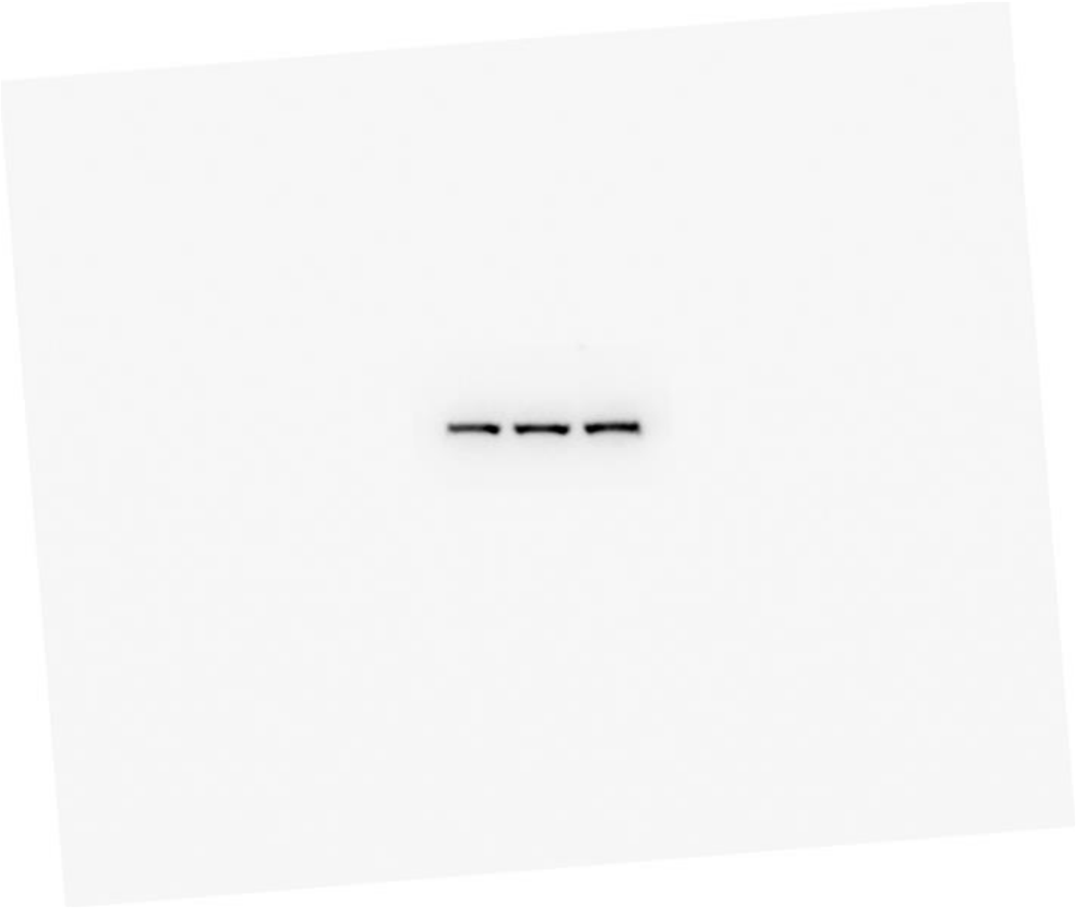

Fig 6G (LS174T cells)

IB: CPT2

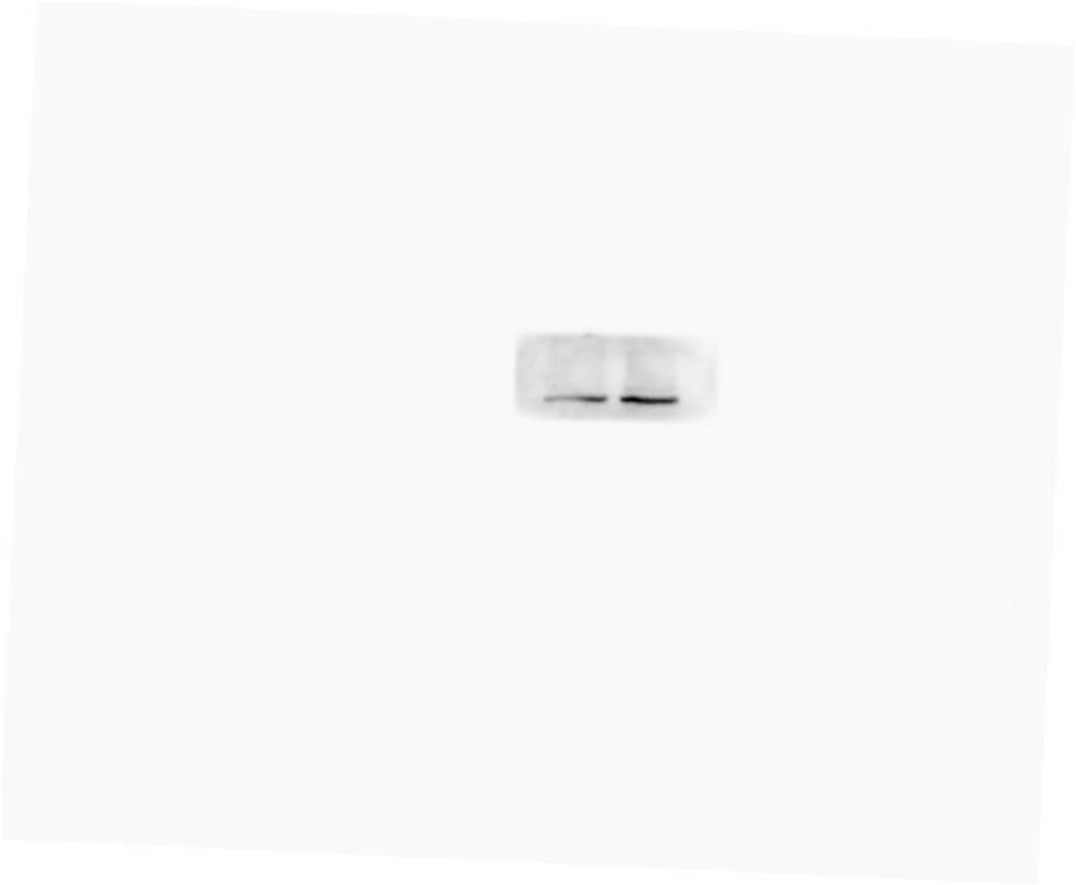

Fig 6H (HCT116 cells; IP: MUL1)

IB: CPT2

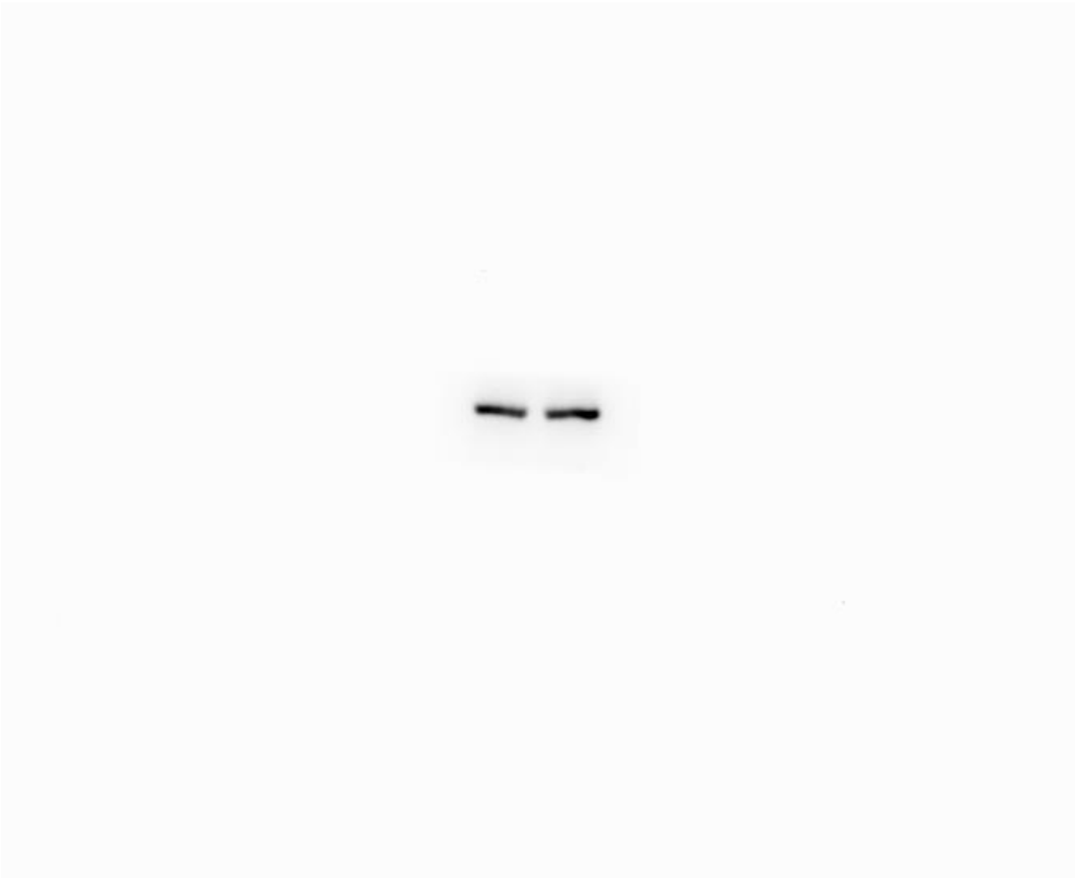

Fig 6H (HCT116 cells; IP: MUL1)

IB: MUL1

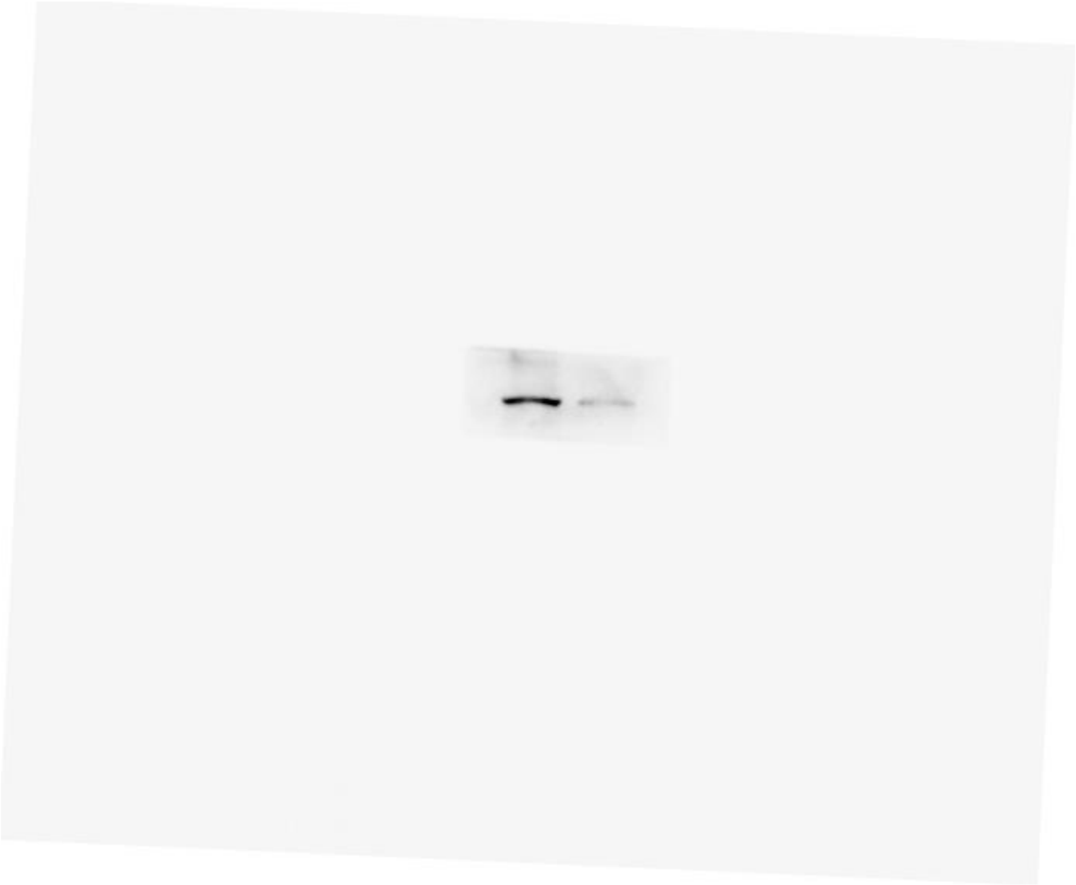

Fig 6H (HT29 cells; IP: MUL1)

IB: CPT2

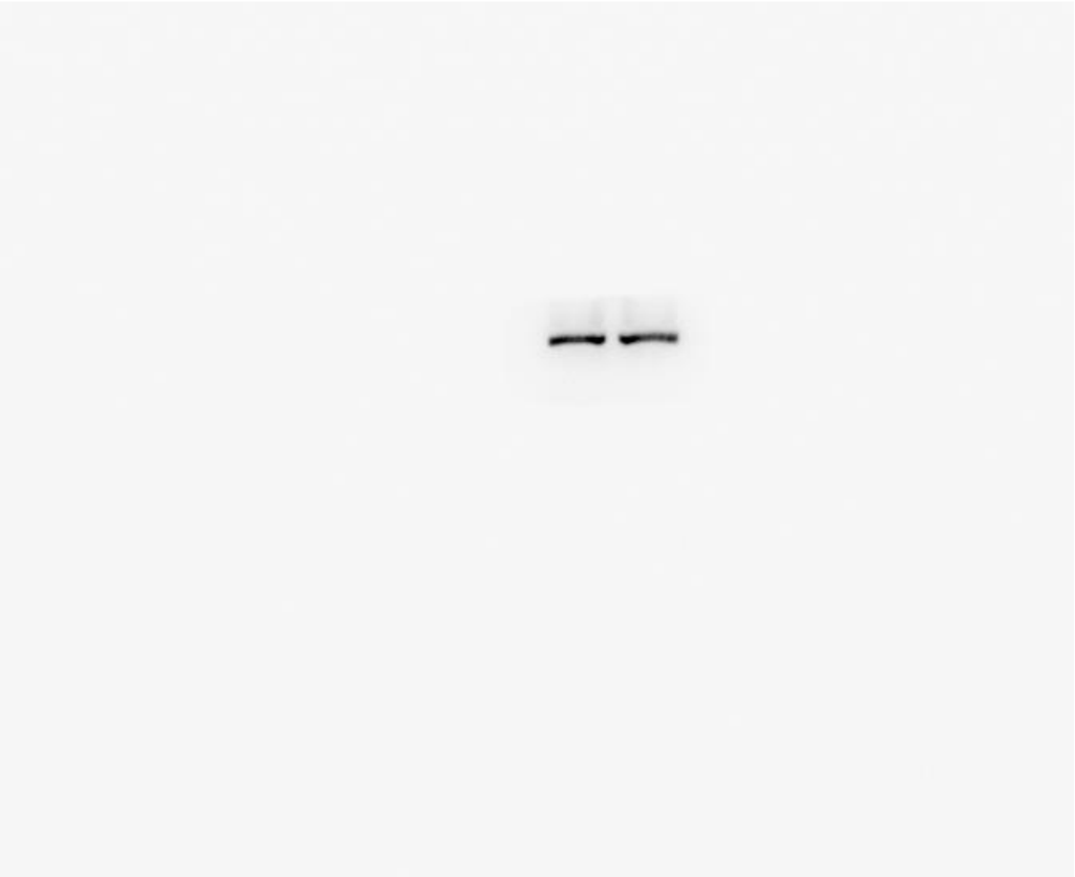

Fig 6H (HT29 cells; IP: MUL1)

IB: MUL1

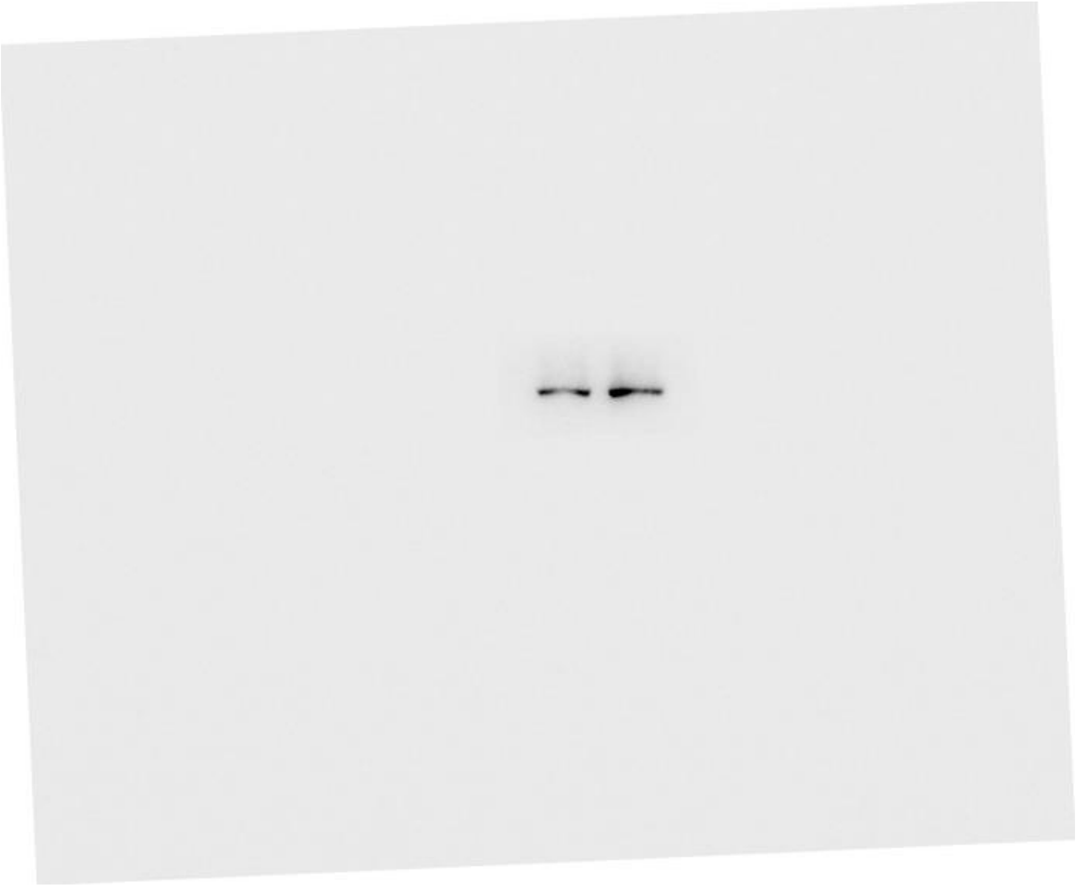

Fig 6H (HCT116 cells; IP:CPT2)

IB: MUL1

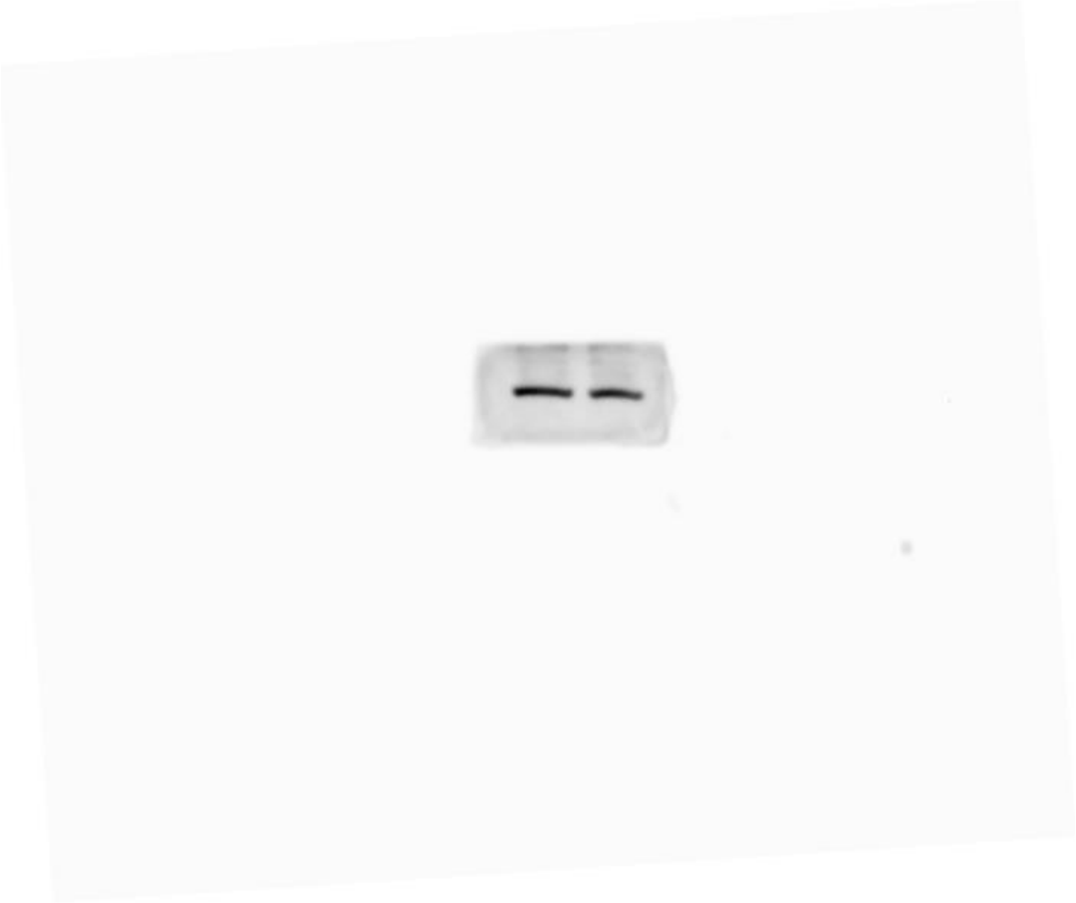

Fig 6H (HCT116 cells; IP:CPT2)

IB: CPT2

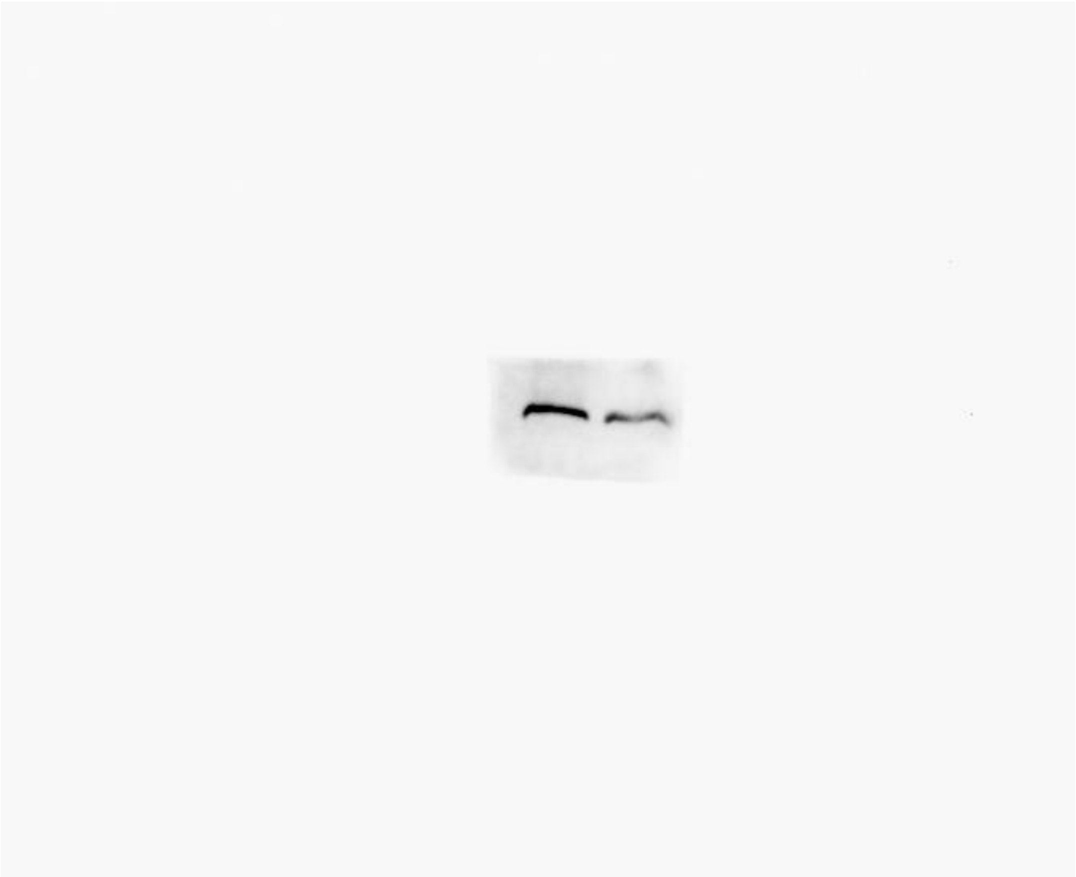

Fig 6H (HT29 cells; IP: CPT2)

IB: MUL1

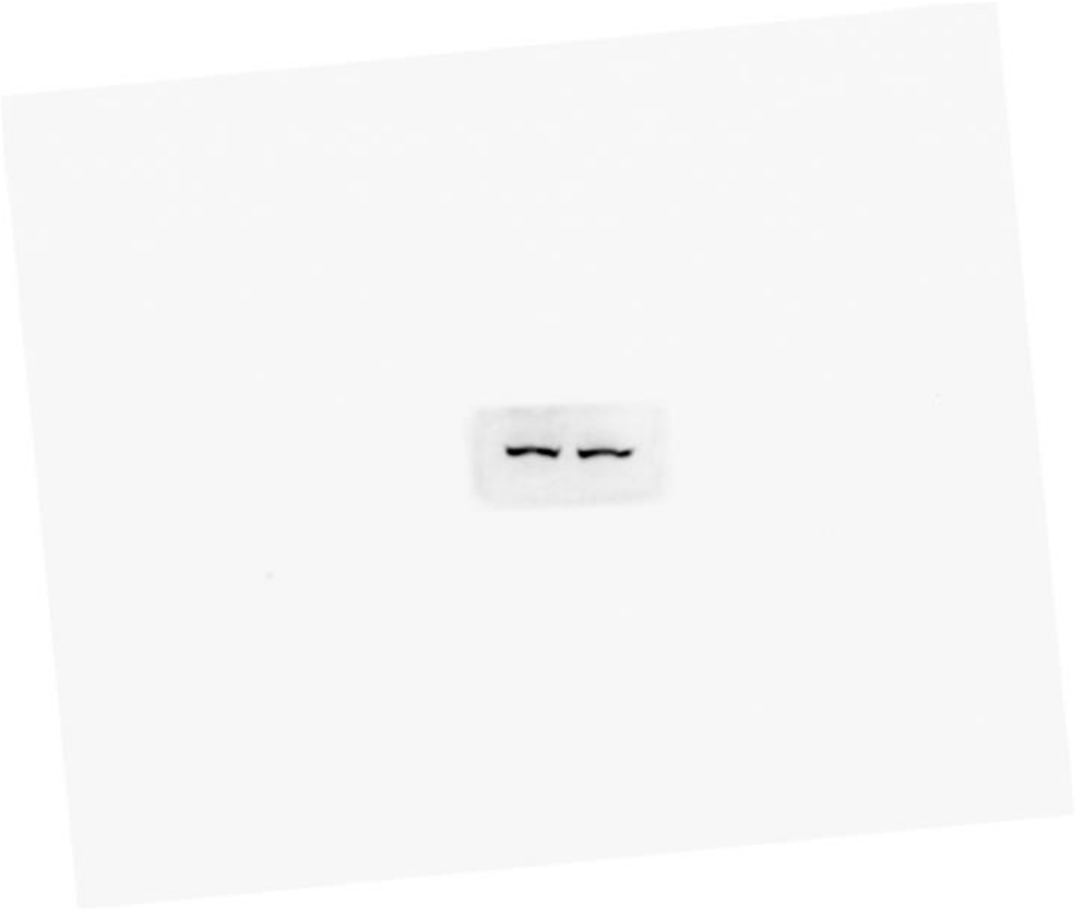

Fig 6H (HT29 cells; IP: CPT2)

IB: CPT2

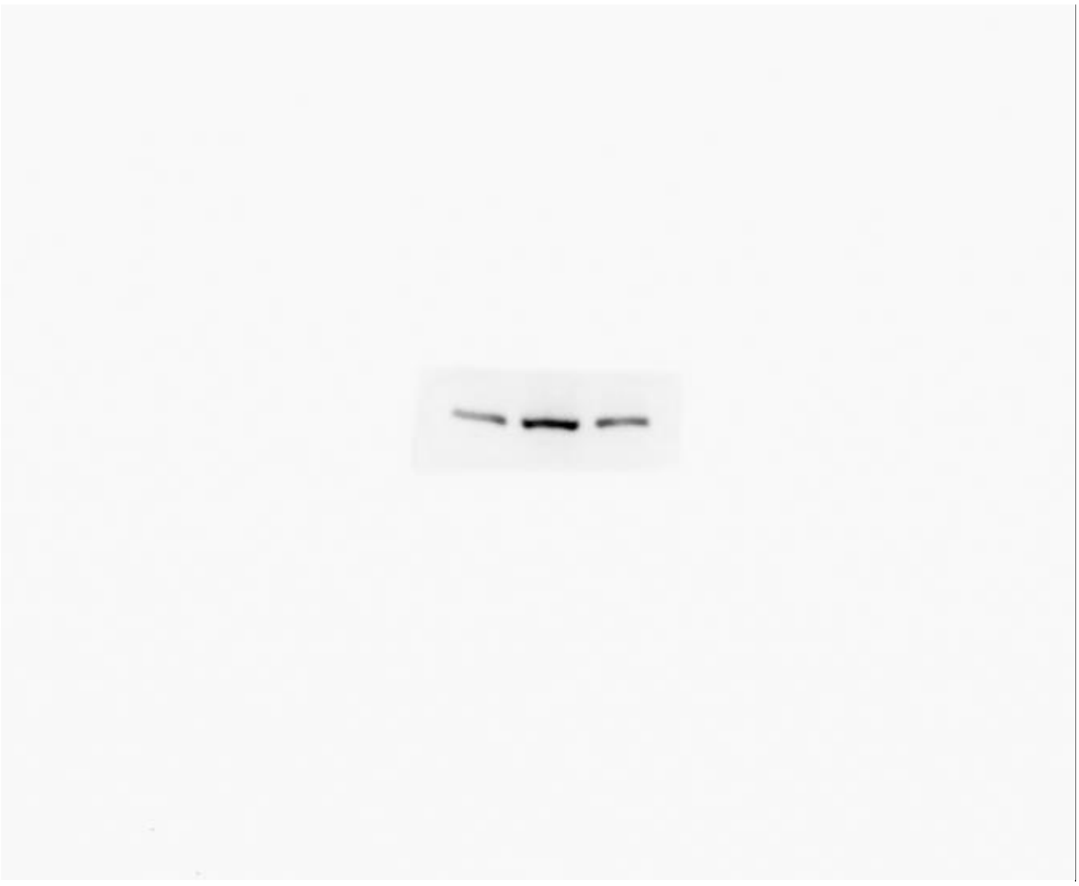

Fig 6I (HCT116 cells; IP: MUL1)

IB: CPT2

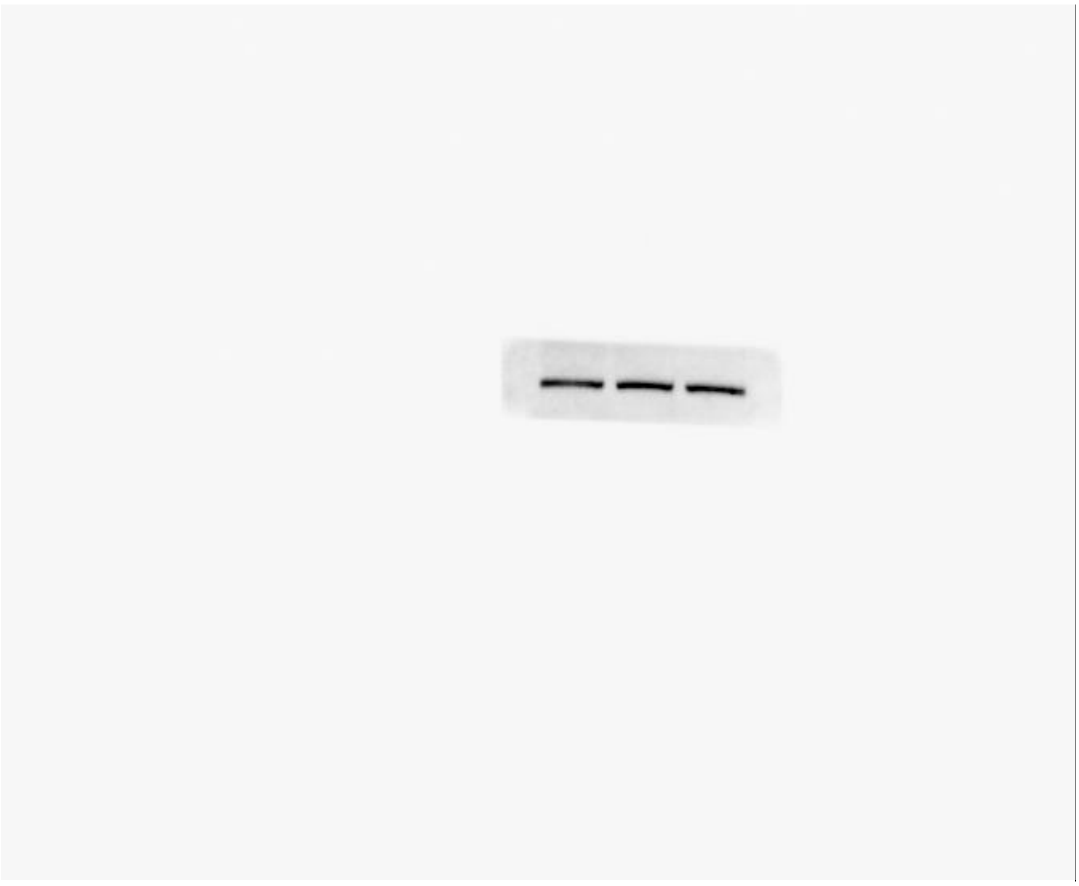

Fig 6I (HCT116 cells; IP: MUL1)

IB: MUL1

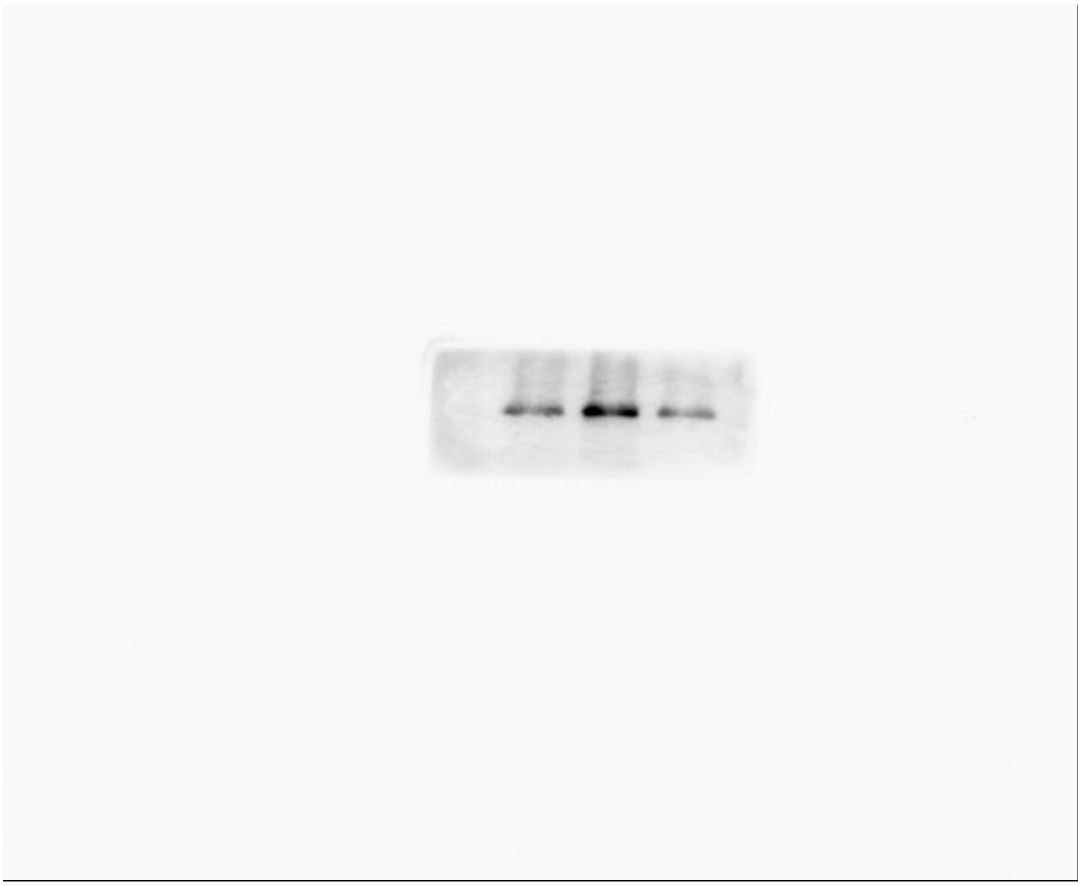

Fig 6I (LS174T cells; IP: MUL1)

IB: CPT2

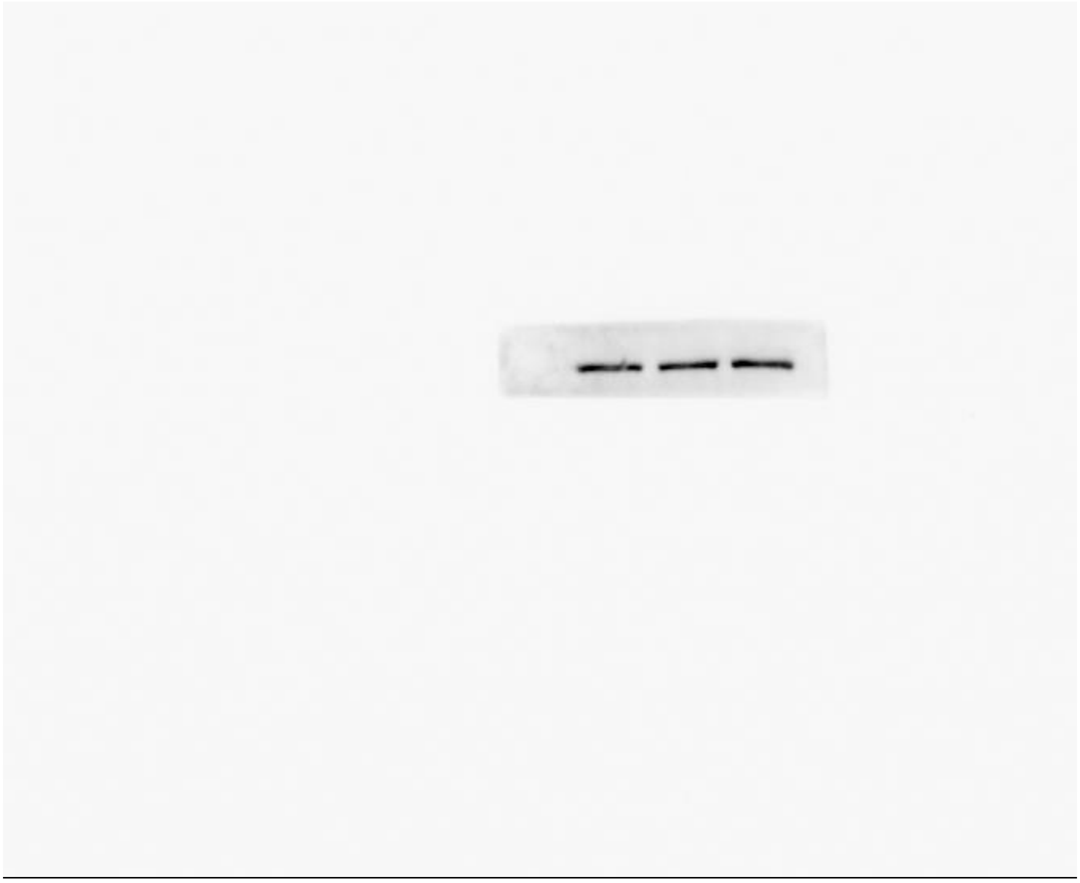

Fig 6I (LS174T cells; IP: MUL1)

IB: MUL1

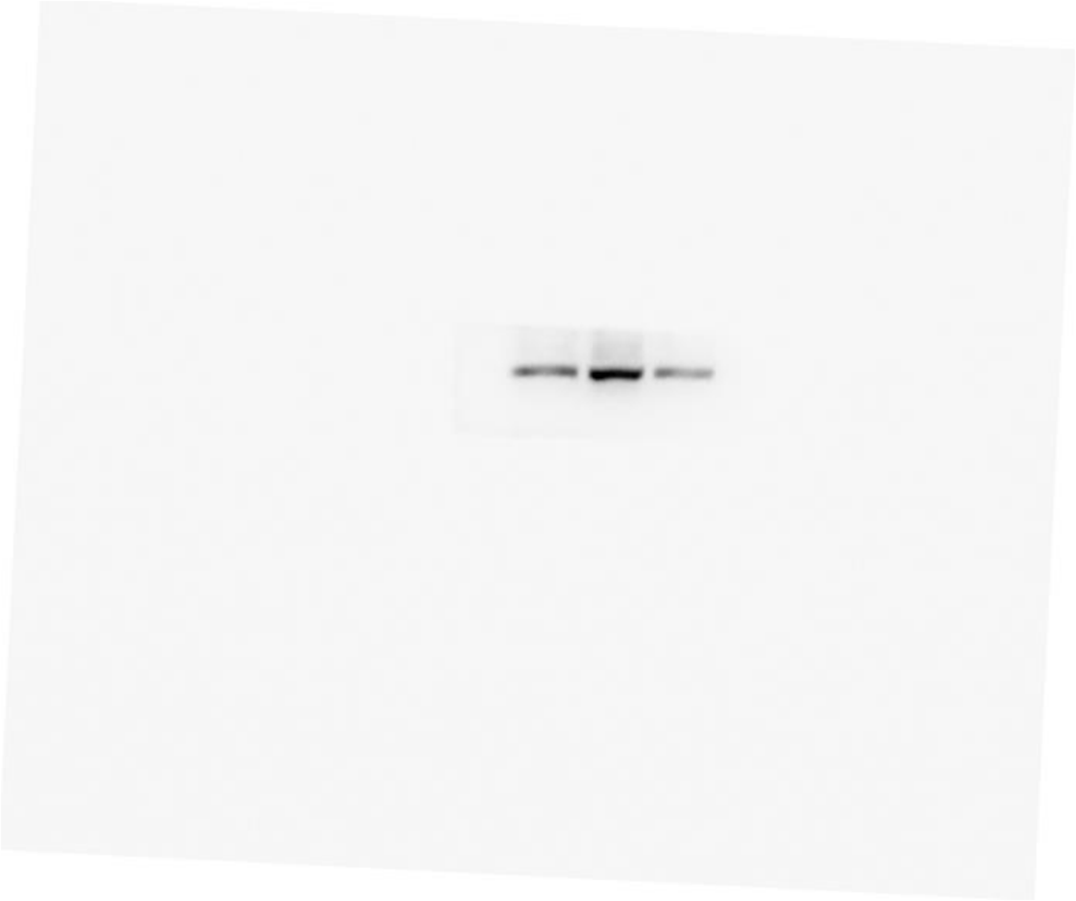

Fig 6I (HCT116 cells; IP: CPT2)

IB: MUL1

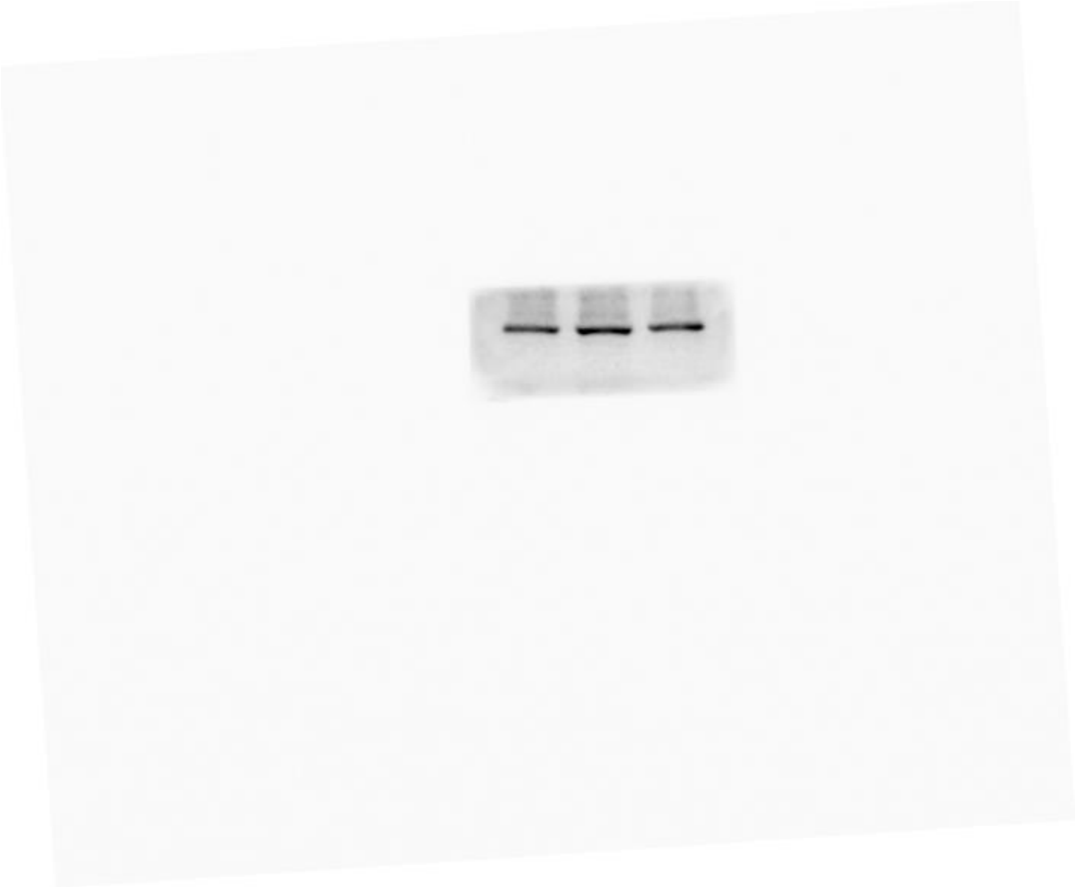

Fig 6I (HCT116 cells; IP: CPT2)

IB: CPT2

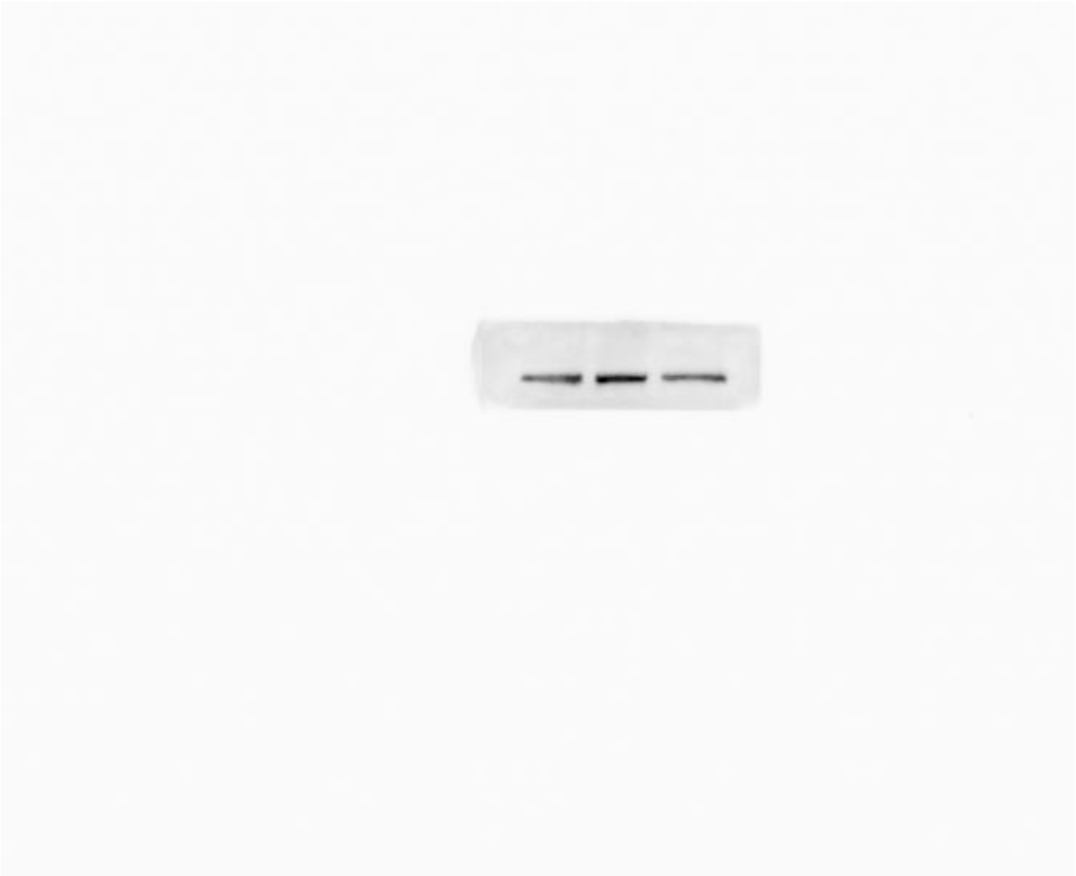

Fig 6I (HT29 cells; IP: CPT2)

IB: MUL1

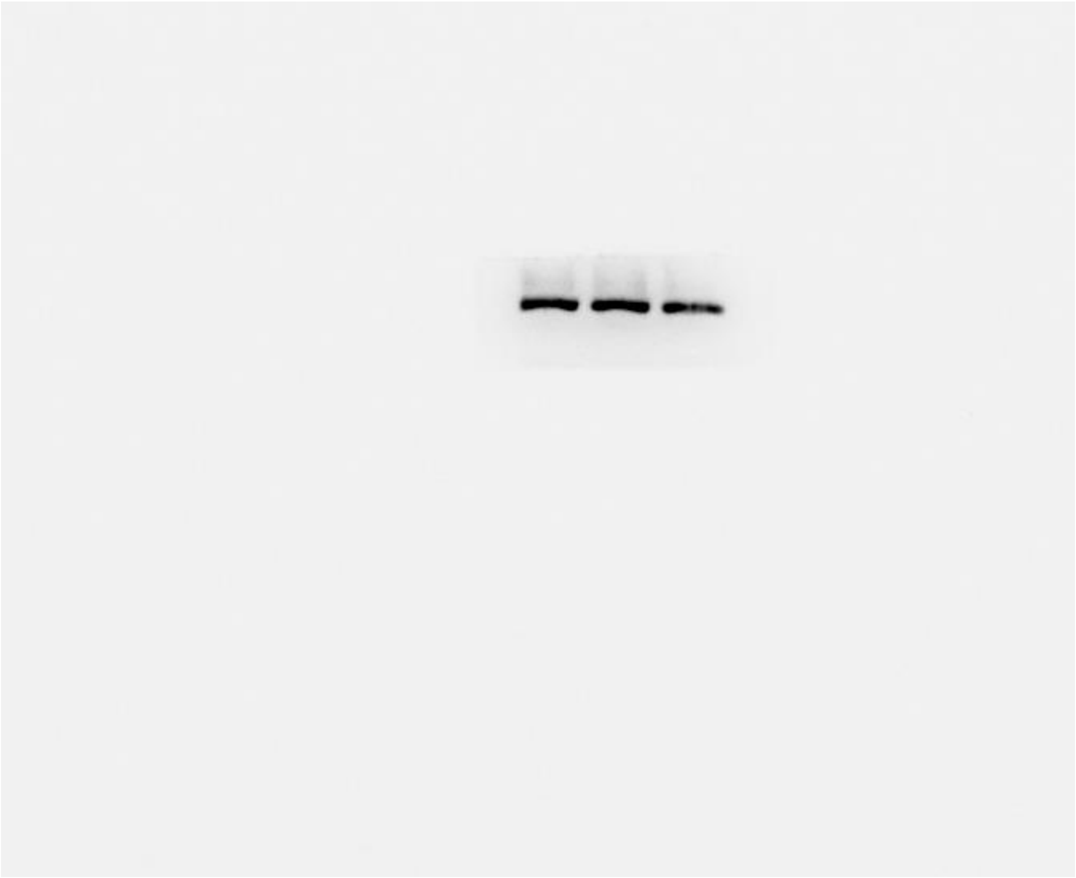

Fig 6I (HT29 cells; IP: CPT2)

IB: CPT2

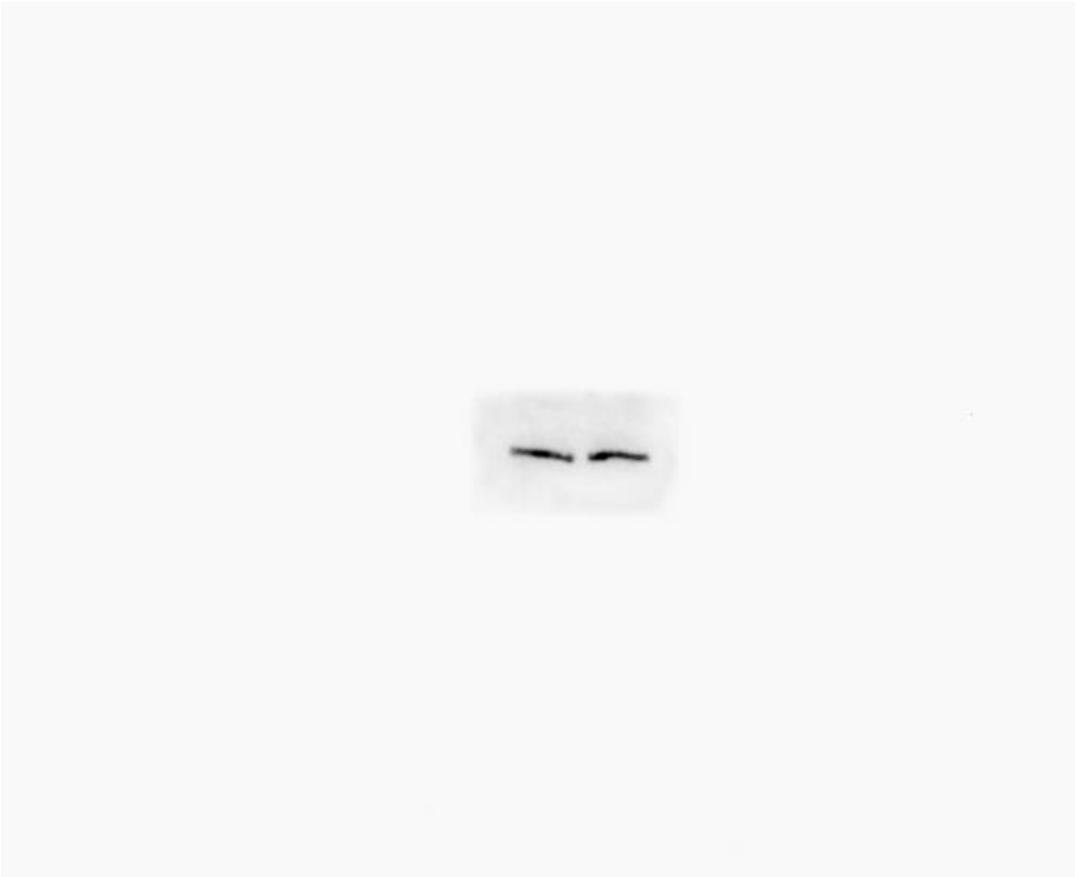

Fig 6J (HCT116 cells)

IB: MUL1

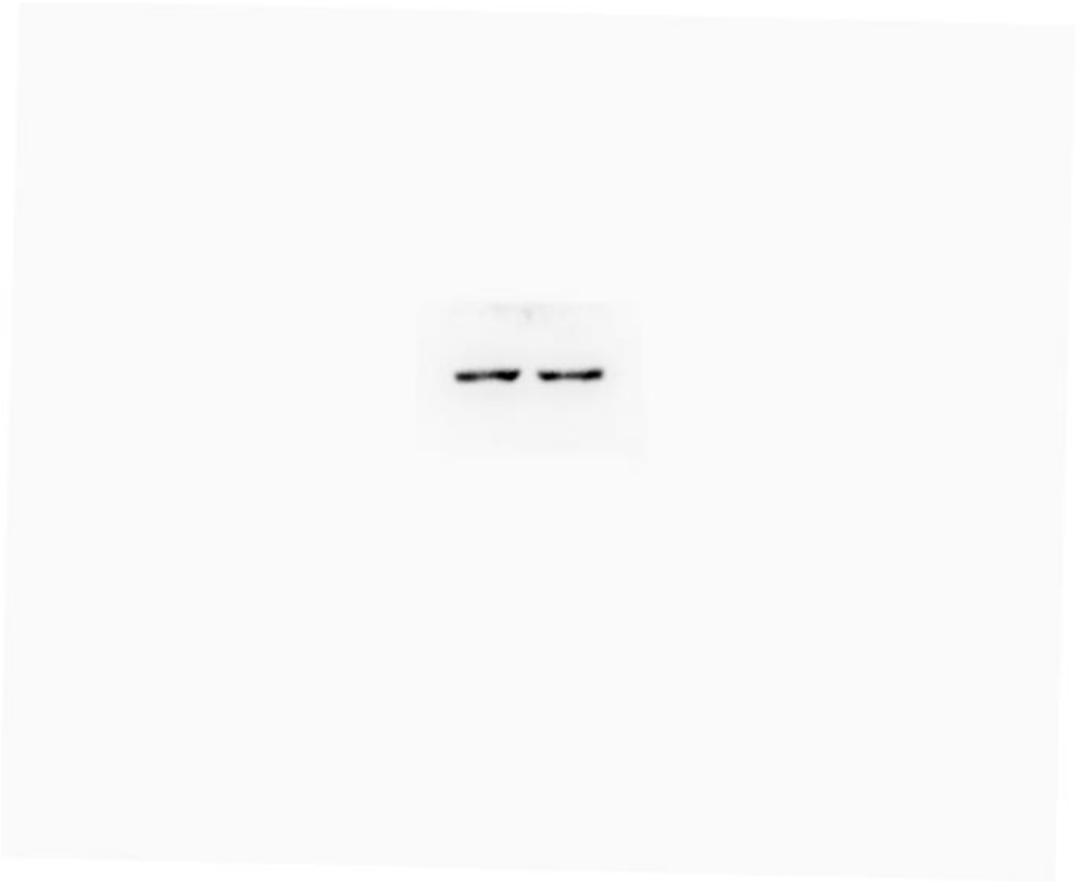

Fig 6J (HCT116 cells)

IB: β-actin

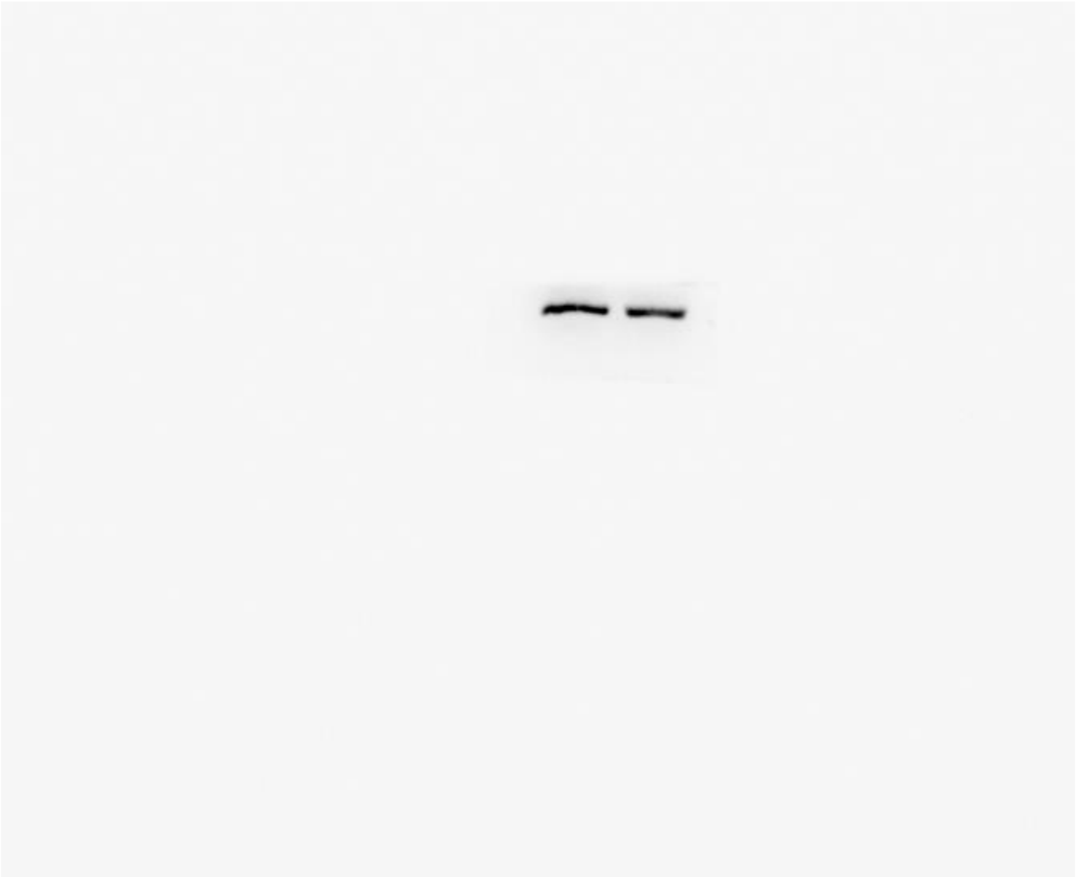

Fig 6J (Ht29 cells)

IB: MUL1

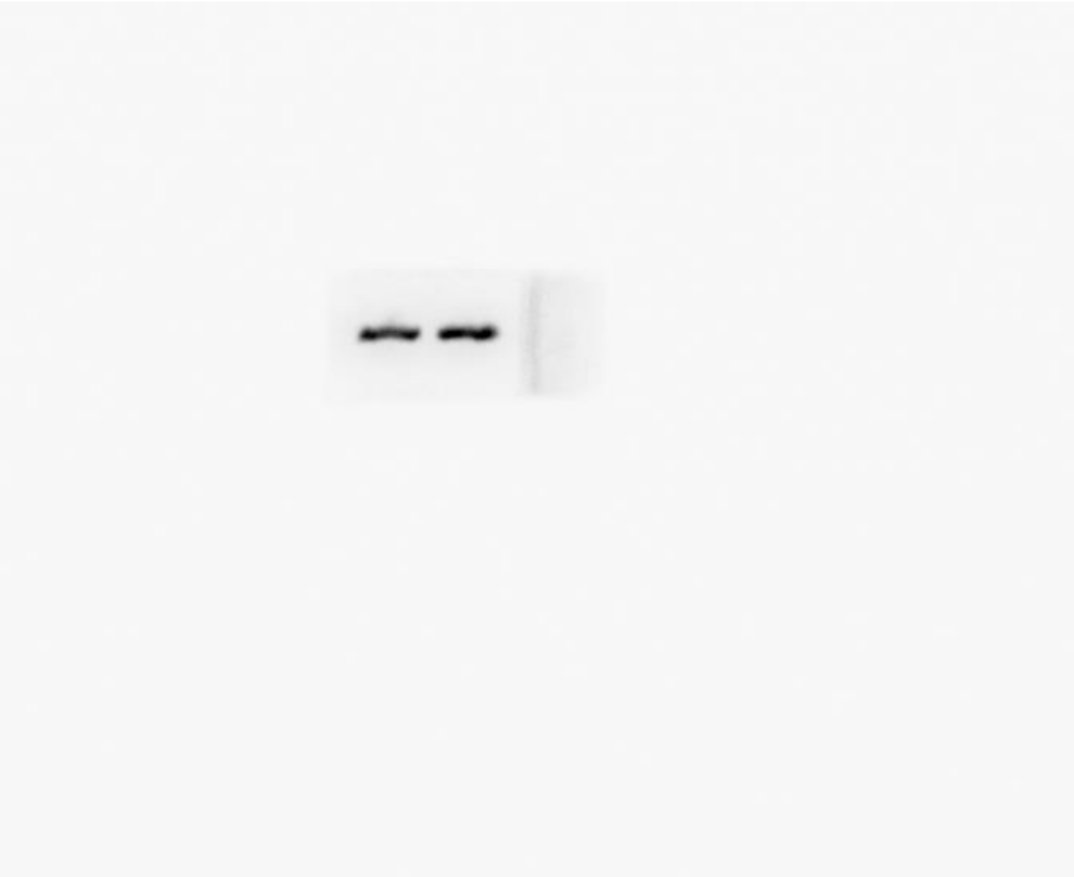

Fig 6J (Ht29 cells)

IB: β-actin
